# Palladium-catalyzed Heck-type reaction of secondary trifluoromethylated alkyl bromides

**DOI:** 10.3762/bjoc.13.258

**Published:** 2017-12-06

**Authors:** Tao Fan, Wei-Dong Meng, Xingang Zhang

**Affiliations:** 1 College of Chemistry, Chemical Engineering and Biotechnology, Donghua University, 2999 North Renmin Road, Shanghai 201620, China,; 2 Key Laboratory of Organofluorine Chemistry, Shanghai Institute of Organic Chemistry, Chinese Academy of Sciences, 345 Lingling Road, Shanghai 200032, China

**Keywords:** alkenes, cross-coupling, Heck-type reaction, palladium, secondary trifluoromethylated alkyl bromides

## Abstract

An efficient palladium-catalyzed Heck-type reaction of secondary trifluoromethylated alkyl bromides has been developed. The reaction proceeds under mild reaction conditions with high efficiency and excellent functional group tolerance, even towards formyl and hydroxy groups. Preliminary mechanistic studies reveal that a secondary trifluoromethylated alkyl radical is involved in the reaction.

## Introduction

With the increasing number of applications of fluorinated compounds in life and materials science, developing efficient and straightforward methods for the synthesis of fluorinated compounds has become more and more important. Although great achievements have been made in the introduction of fluorine atom(s) into organic molecules over the past decade, investigations mainly focused on direct fluorination or fluoroalkylation of aromatics [1–5]. Fluoroalkylated alkenes are a kind of important fluorinated structural motifs and have wide applications in medicinal chemistry and advanced functional materials [6–9]. However, developing efficient methods for the synthesis of such valuable structures has received less attention [10–15].

Commonly, fluoroalkylated alkenes can be prepared through fluoroalkyl radical addition of alkynes via an atom transfer pathway [16–17] or cross-coupling of alkenyl halides with fluoroalkyl metal species [18–24]. Recently, we reported a palladium-catalyzed Heck-type reaction of fluoroalkyl bromides, representing an efficient and straightforward alternative to access fluoroalkylated alkenes [25–27]. Preliminary mechanistic studies reveal that a fluoroalkyl radical via a single electron transition (SET) pathway is involved in the reaction. Inspired by this work, we question that whether secondary fluoroalkylated alkyl bromides are also suitable substrates for such a Heck-type reaction. To the best of our knowledge, the palladium-catalyzed Heck-type reaction of secondary fluoroalkylated alkyl bromides with alkenes remains a challenge and has not been reported so far [28–32] due to the sluggish oxidative addition [33] of alkyl halides to palladium and facile β-hydride elimination [34–35] of alkylpalladium species. Additionally, the resulting fluoroalkylated allylic compounds can serve as a versatile building block for the synthesis of complex fluorinated molecules [36–37]. Herein, we describe a palladium-catalyzed Heck-type reaction of secondary trifluoromethylated alkyl bromides. The reaction proceeds under mild reaction conditions with broad substrate scope and high efficiency. The reaction can also extend to secondary difluoroalkylated alkyl iodide. Preliminary mechanistic studies reveal that a secondary alkyl radical via a SET pathway is involved in the reaction.

## Results and Discussion

We began this study by choosing styrene (**1a**) and 2-bromo-1,1,1-trifluorohexane (**2a**) as model substrates (Table 1). Initially, a 27% yield of product **3a** along with small amount of hydrodebrominated byproduct **3a′** (2% yield) were obtained when the reaction of **1a** (0.2 mmol, 1.0 equiv) with **2a** (2.0 equiv) was carried out in the presence of PdCl_2_(PPh_3_)_2_ (5 mol %), Xantphos (10 mol %) and K_2_CO_3_ (2.0 equiv) in DCE for 16 h at 80 °C (Table 1, entry 1). After optimization of the reaction conditions (for details, see Supporting Information File 1), a dramatically improved yield of **3a** (83% yield upon isolation) was provided by using KOAc as a base (Table 1, entry 5). K_3_PO_4_ also led to a good yield of **3a** (Table 1, entry 6), but other bases, such as Na_2_CO_3_, Cs_2_CO_3_, NaOAc and KF were less effective (Table 1, entries 2–4 and 7). Among all the tested palladium salts (Table 1, entries 8–12), PdCl_2_(PPh_3_)_2_ was proved to be the most effective catalyst and provided **3a** in 84% yield (Table 1, entry 5). The use of Xantphos was crucial for the reaction efficiency (Table 1, entry 5). Other ligands either led to low yield or showed no reactivity (Table 1, entry 13, for details, see Supporting Information File 1). The beneficial effect of Xantphos is probably due to its large bit angle to promote the reaction [38–39]. However, the exact role of Xantphos remains elusive. Finally, the optimal reaction conditions were identified by decreasing the loading amount of Xantphos from 10 mol % to 7.5 mol % with a higher concentration of **1a** and **2a**, providing **3a** in 88% yield upon isolation (Table 1, entry 14).

**Table 1:** Representative results for the optimization of Pd-catalyzed cross-coupling between **1a** and 2-bromo-1,1,1-trifluorohexane (**2a**)^a^.

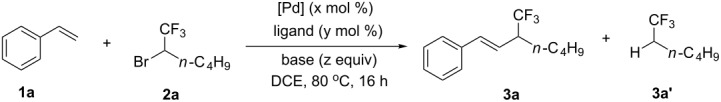				

entry	[Pd] (mol %)	ligand (mol %)	base (equiv)	**3a**, yield (%)^b^

1	PdCl_2_(PPh_3_)_2_ (5)	Xantphos (10)	K_2_CO_3_ (2)	27
2	PdCl_2_(PPh_3_)_2_ (5)	Xantphos (10)	Na_2_CO_3_ (2)	5
3	PdCl_2_(PPh_3_)_2_ (5)	Xantphos (10)	Cs_2_CO_3_ (2)	26
4	PdCl_2_(PPh_3_)_2_ (5)	Xantphos (10)	NaOAc (2)	42
5	PdCl_2_(PPh_3_)_2_ (5)	Xantphos (10)	KOAc (2)	84 (83)
6	PdCl_2_(PPh_3_)_2_ (5)	Xantphos (10)	K_3_PO_4_ (2)	75
7	PdCl_2_(PPh_3_)_2_ (5)	Xantphos (10)	KF (2)	37
8	PdCl_2_ (5)	Xantphos (10)	KOAc (2)	18
9	Pd(OAc)_2_ (5)	Xantphos (10)	KOAc (2)	51
10	PdCl_2_·dppp (5)	Xantphos (10)	KOAc (2)	Trace
11	PdCl_2_·dppf (5)	Xantphos (10)	KOAc (2)	69
12	Pd(PPh_3_)_4_ (5)	Xantphos (10)	KOAc (2)	64
13	PdCl_2_(PPh_3_)_2_ (5)	dprephos	KOAc (2)	45
14^c^	PdCl_2_(PPh_3_)_2_ (5)	Xantphos (7.5)	KOAc (2)	95 (88)

^a^Reaction conditions (unless otherwise specified): **1a** (0.2 mmol, 1.0 equiv), **2a** (2.0 equiv), base (2.0 equiv), DCE (3 mL), 16 h. ^b^Determined by ^19^F NMR using fluorobenzene as an internal standard (isolated yield in parentheses). ^c^**1a** (0.4 mmol, 1.0 equiv), **2a** (2.0 equiv) and DCE (3 mL) were used.

**Scheme 1:**
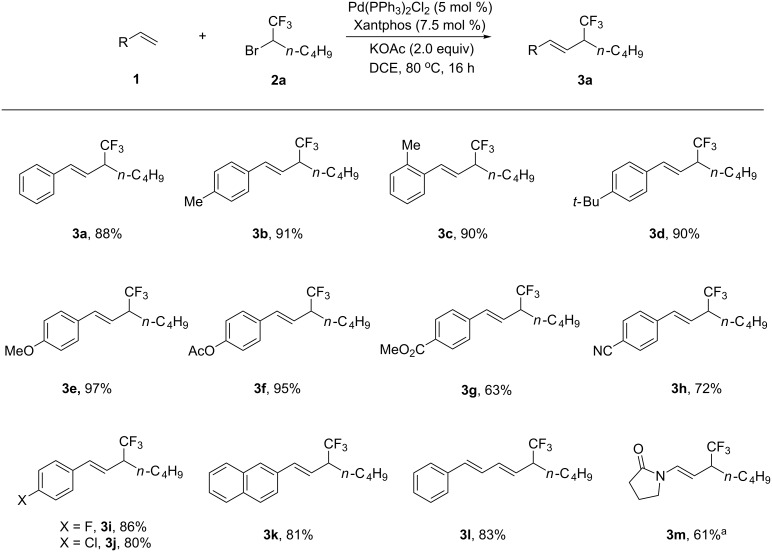
Palladium-catalyzed Heck-type reaction of 2-bromo-1,1,1-trifluorohexane (**2a**) with alkenes **1**. Reaction conditions (unless otherwise specified): **1** (0.4 mmol, 1.0 equiv), **2a** (2.0 equiv), DCE (3 mL), 80 °C, 16 h. All reported yields are those of isolated products. ^a^The reaction was conducted at 100 °C.

With the optimized reaction conditions in hand, a variety of alkenes were examined. As shown in Scheme 1, reactions of **2a** with a series of styrene derivatives **1** proceeded smoothly and provided **3** in moderate to excellent yields (Scheme 1). Generally, substrates bearing electron-donating groups afforded higher yields than that of alkenes bearing electron-withdrawing groups. Various versatile functional groups, such as ester, cyano, and chloride showed good tolerance to the reaction (**3f**–**j**). Vinylnaphthalene also furnished the corresponding product efficiently (**3k**). The conjugated alkene did not interfere with the reaction efficiency, providing **3l** in 83% yield. The reaction was not restricted to aromatic alkenes, enamide was also applicable to the reaction and afforded **3m** in 61% yield.

**Scheme 2:**
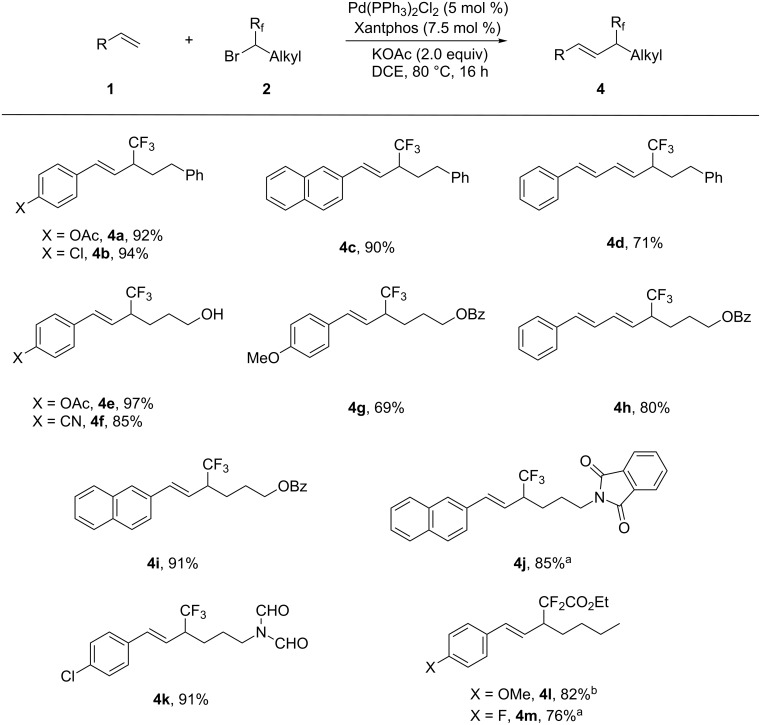
Palladium-catalyzed Heck-type reaction of fluorinated secondary bromides (iodides) **2** with alkenes **1**. Reaction conditions (unless otherwise specified): **1** (0.4 mmol, 1.0 equiv), **2** (2.0 equiv), DCE (3 mL), 80 °C, 16 h. All reported yields are those of isolated products. ^a^The reaction was carried out in 0.2 mmol scale. ^b^Ethyl 2,2-difluoro-2-iodoacetate was used.

To demonstrate the generality of this method further, reactions of alkenes with various secondary trifluoromethylated alkyl bromides were performed and provided the corresponding products **4** with high yield. As shown in Scheme 2, a variety of secondary trifluoromethylated alkyl bromides, including those substrates bearing base and nucleophile sensitive functional groups, such as hydroxy, aldehyde, and phthalimide (**4e**, **4f**, **4j** and **4k**), were all applicable to the reaction, thus offering good opportunities for downstream transformations and highlighting the utility of the current process further. (3-Bromo-4,4,4-trifluorobutyl)benzene and 4-bromo-5,5,5-trifluoropentyl benzoate were also successfully employed to couple with a conjugated alkene and afforded the corresponding products **4d** and **4h** in good yields. The current cross coupling was also applicable to the secondary difluoroalkylated alkyl halides as demonstrated by the representative reaction of secondary ethyl 2,2-difluoro-2-iodoacetate with *para*-methoxystyrene and *para*-fluorostyrene (**4l** and **4m**).

To probe whether a secondary trifluoromethylated alkyl radical is involved in the current reaction, radical inhibition experiments were performed (Table 2). When a reaction of **1a** with **2a** was carried out in the presence of PdCl_2_(PPh_3_)_2_ (5 mol %), Xantphos (7.5 mol %) and KOAc in DCE at 80 °C, the addition of an electron transfer scavenger 1,4-dinitrobenzene [25–27] dramatically diminished the yield of **3a** (Table 2, entries 2 and 3), and catalytic amount of radical inhibitor hydroquinone totally shut down the reaction (Table 2, entry 4). Thus, these results suggest that a secondary trifluoromethylated alkyl radical via a SET pathway is likely involved in the reaction.

**Table 2:** Radical inhibition experiments of Pd-catalyzed cross-coupling between **1a** and 2-bromo-1,1,1-trifluorohexane (**2a**)^a^.

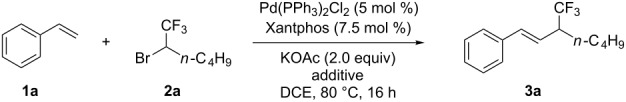		

entry	additive (equiv)	**3a**, yield (%)^b^

1	none	95 (88)
2	1,4-dinitrobenzene (0.2)	22
3	1,4-dinitrobenzene (1.0)	5
4	hydroquinone (0.2)	0

^a^Reaction conditions: **1** (0.4 mmol, 1.0 equiv), **2a** (2.0 equiv), KOAc (2.0 equiv), DCE (3 mL), 16 h. ^b^Determined by ^19^F NMR using fluorobenzene as an internal standard.

**Scheme 3:**
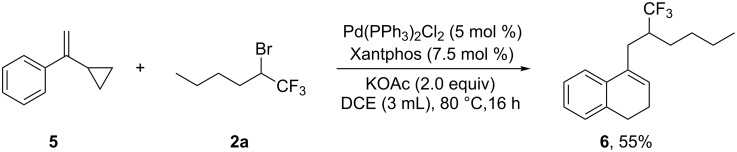
Radical clock experiment for mechanistic studies.

The existence of an alkyl radical species was further confirmed by the radical clock experiment. As illustrated in Scheme 3, when α-cyclopropylstyrene (**5**) [40] was subjected to the reaction, a ring-opened compound **6** was isolated in 55% yield, demonstrating that a secondary trifluromethylated alkyl radical existed in the reaction is reasonable.

**Scheme 4:**
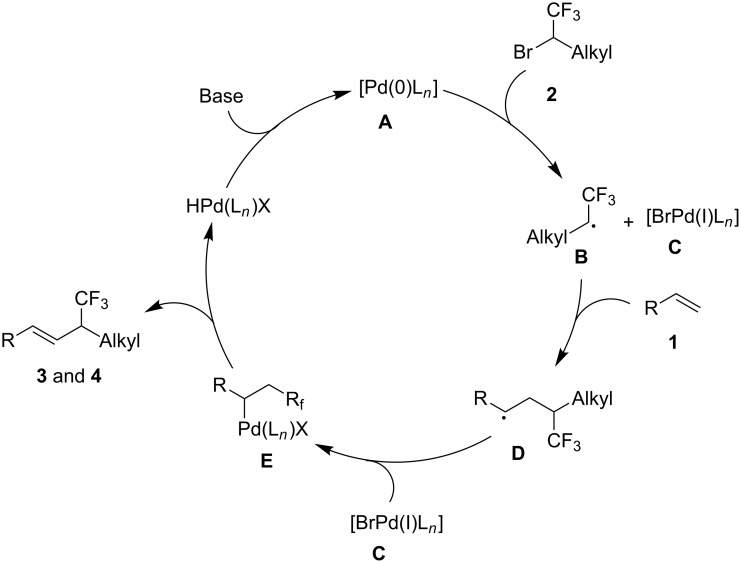
Proposed mechanism.

On the basis of these results and our previous reports [25–27], a plausible mechanism is proposed (Scheme 4). The reaction begins with the reaction of [PdL*_n_*(0)] with secondary trifluoromethylated **2** via a SET pathway to generate alkyl radical **B**. **B** subsequently reacts with alkene to produce new radical species **D**, which then recombines with [L*_n_*Pd(I)Br] **C** to give the key palladium-complex **E**. Finally, a β-hydride elimination delivers trifluoromethylated allylic products **3** and **4**.

## Conclusion

In conclusion, we have developed an efficient method for preparation of aliphatic alkenes branched with a trifluoromethyl group by palladium catalyzed Heck-type reaction of secondary trifluoromethylated alkyl bromides. The reaction proceeds under mild conditions and showed good functional group compatibility, even towards formyl and hydroxy groups, thus providing a facile route for applications in discovering biologically interesting molecules. Preliminary mechanistic studies reveal that a secondary trifluoromethylated alkyl radical is involved in the reaction.

## Supporting Information

File 1 General experimental information, experimental details on the synthesis of compounds **2**–**4** and **6**; full characterization data as well as ^1^H/ ^19^F/ ^13^C NMR spectra of all products.
