# Supplementary material for: Palladium-catalyzed Heck-type reaction of secondary trifluoromethylated alkyl bromides
Source: Beilstein J Org Chem. 2017 Dec 6;13:2610–6. doi: 10.3762/bjoc.13.258 (PMC5727777; doi:10.3762/bjoc.13.258)
Supplement: File 1 — General experimental information, experimental details on the synthesis of compounds 2–4 and 6; full characterization data as well as 1H/ 19F/ 13C NMR spectra of all products. [file Beilstein_J_Org_Chem-13-2610-s001.pdf]

Supporting Information

for

Palladium-catalyzed Heck-type reaction of secondary trifluoromethylated alkyl bromides

Tao Fan[1], Wei-Dong Meng[1] and Xingang Zhang*[2]

Address: [1]College of Chemistry, Chemical Engineering and Biotechnology, Donghua University, 2999 North Renmin Road, Shanghai 201620, China, and [2]Key Laboratory of Organofluorine Chemistry, Shanghai Institute of Organic Chemistry, Chinese Academy of Sciences, 345 Lingling Road, Shanghai 200032, China

Email: Xingang Zhang* - xgzhang@mail.sioc.ac.cn.

*Corresponding author

General experimental information, experimental details on the synthesis of compounds 2–4 and 6; full characterization data as well as ^{1}H, ^{19}F and ^{13}C NMR spectra of all products.

List of contents

1) General information……………………………………………………………………….S3

2) Screening of Pd-catalyzed cross-coupling of **2a** with styrene **1a**……………………….....…S3

3) Mechanistic studies…………………………………………………………..………….….S6

4) Preparation of secondary fluoroalkylated alkyl halides **2**…………………………S7

5) General procedure for Pd-catalyzed cross-coupling of secondary fluorinated alkyl halides with alkenes……………………………………………………………………………..S11

6) Data of compounds **3** and **4** ……………………………………………………….…..…S12

7) References……………………………………………………………………………….…S23

8) Copies of ^{1}H NMR, ^{19}F NMR and ^{13}C NMR spectra of compounds **3**，**4** and **6**..S24

General information

^{1}H NMR and ^{13}C NMR spectra were recorded on Bruker AM 400, Agilent MR 400 and Agilent MR 500 spectrometers. ^{19}F NMR was recorded on an Agilent MR 400 spectrometer ($CFCl_3$ as an external standard and low field is positive). Chemical shifts (δ) are reported in ppm, and coupling constants (*J*) are in Hertz (Hz). The following abbreviations were used to explain the multiplicities: s = singlet, d = doublet, t = triplet, q = quartet, m = multiplet, br = broad. NMR yield was determined by ^{19}F NMR using fluorobenzene as an internal standard before working up the reaction.

Materials: All reagents were used as received from commercial sources, unless specified otherwise, or prepared as described in the literature. All reagents were weighed and handled in air, and refilled with an inert atmosphere of N_2 at room temperature.

Screening of Pd-catalyzed cross-coupling of 2a with styrene 1a (Tables S1 – S4). To a 25 mL of Schlenk tube were added [Pd] (5 mol %), ligand (7.5–10 mol %) and base (2.0 equiv) under air. The mixture was then evacuated and backfilled with N_2 for three times. Styrene (**1a**, 0.2 mmol), 2-bromo-1,1,1-trifluorohexane (2a, 2.0 equiv) and solvent (3 mL) were then added. The reaction mixture was heated to 80 °C (oil bath). After stirring for 16 h, the reaction mixture was cooled to room temperature and diluted with ethyl acetate (2 mL). The yield was determined by ^{19}F NMR using fluorobenzene as an internal standard. If necessary, the reaction mixture was filtered with a pad of cellite. The filtrate was concentrated, and the residue was purified with silica gel chromatography to give product **3a**.

Table S1. Screening of bases.[a]

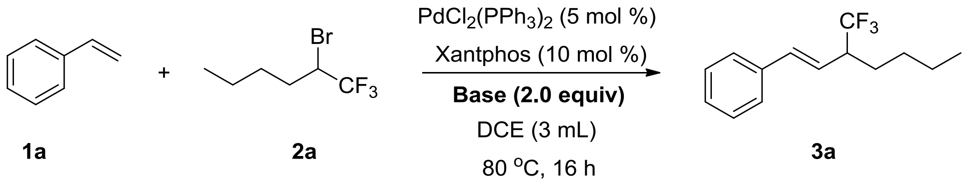

Entry	Base	Yield **3a** (%)[b]
1	Na_2CO_3	5
2	K_2CO_3	27
3	Cs_2CO_3	26
**4**	**KOAc**	**84 (83)**
5	NaOAc	42
6	K_3PO_4	75
7	KF	37
8	*t*-BuOLi	27

[a]Reaction conditions (unless otherwise specified): **1a** (0.2 mmol, 1.0 equiv), **2a** (2.0 equiv), DCE (3 mL). [b]Determined by ^{19}F NMR using fluorobenzene as an internal standard and number in parenthesis is isolated yield.

Table S2. Screening of solvents.[a]

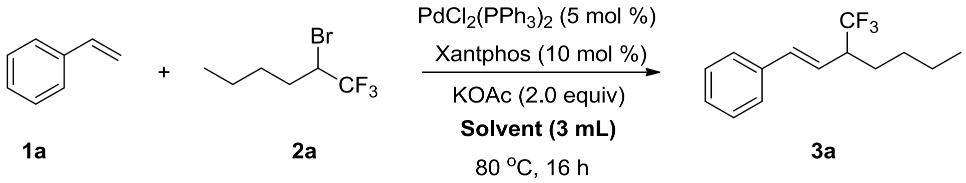

Entry	Solvent	Yield (%)[b]
1	1,4-Dioxane	26
2	DMSO	69
3	THF	35
4	DMA	49
5	MeCN	61
**6**	**DCE**	**84 (83)**
7	Toluene	74

[a]Reaction conditions (unless otherwise specified): **1a** (0.2 mmol, 1.0 equiv), **2a** (2.0 equiv), Solvent (3 mL). [b]Determined by ^{19}F NMR using fluorobenzene as an internal standard and number in parenthesis is isolated yield.

Table S3. Screening of palladium sources.[a]

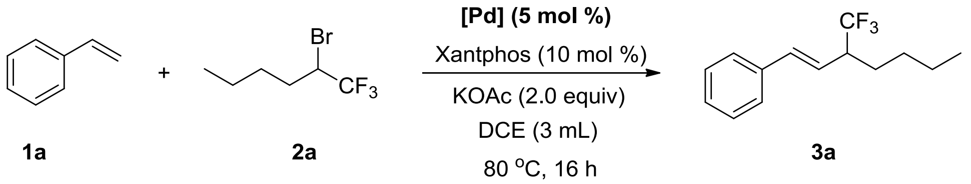

1a **2a** **3a**

Entry	[Pd]	Yield (%)[b]
1	$Pd(PPh_3)_4$	64
2	$PdCl_2$	18
3	$Pd(OAc)_2$	51
4	$PdCl_2$·dppp	trace
5	$PdCl_2$·dppf	69
**6**	**$PdCl_2(PPh_3)_2$**	**84 (83)**

[a]Reaction conditions (unless otherwise specified): **1a** (0.2 mmol, 1.0 equiv), **2a** (2.0 equiv), DCE (3 mL). [b]Determined by ^{19}F NMR using fluorobenzene as an internal standard and number in parenthesis is isolated yield.

Table S4. Screening of ligands.[a]

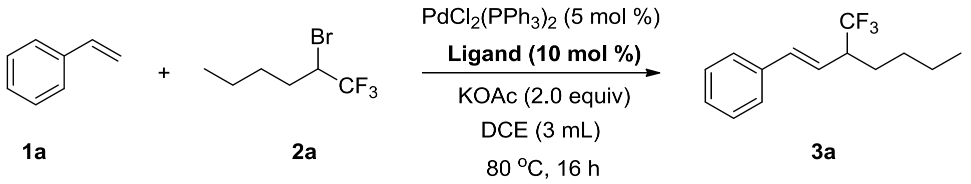

1a **2a** **3a**

Entry	Ligand (x)	Yield (%)[b]
**1**	**Xantphos (10)**	**84 (83)**
2	Dppe (10)	0
3	Dppp (10)	0
4	Dppb (10)	0
5	Dppf (10)	0
6	Sphos (10)	0
7	Ruphos (10)	0
8	Davephos (10)	0
9	Dpephos (10)	45
10	Binap (10)	0
**11**[c]	**Xantphos (7.5)**	**95 (88)**

[a]Reaction conditions (unless otherwise specified): **1a** (0.2 mmol, 1.0 equiv), **2a** (2.0 equiv), DCE (3 mL). [b]Determined by ^{19}F NMR using fluorobenzene as an internal standard and number in parenthesis is isolated yield. [c]**1a** (0.4 mmol, 1.0 equiv), **2a** (2.0 equiv), KOAc (2.0 equiv), DCE (3 mL) were used.

Mechanistic studies

Table S5. Radical inhibition experiments [a]

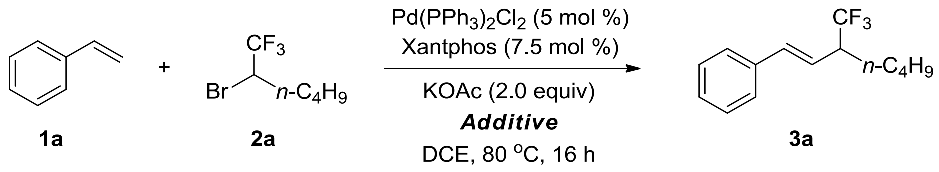

Entry	Additive (equiv)	**3a** Yield(%)[a]
1	none	95 (88)
2	1,4-dinitrobenzene (0.2)	22
3	1,4-dinitrobenzene (1.0)	5
4	Hydroquinone (0.2)	0

[a]Reaction conditions (unless otherwise specified): **1a** (0.4 mmol, 1.0 equiv), **2a** (2.0 equiv), DCE (3 mL). [b]Determined by ^{19}F NMR using fluorobenzene as an internal standard and number in parenthesis is isolated yield.

Procedure for radical inhibition experiments: To a 25 mL of Schlenk tube were added $PdCl_2(PPh_3)_2$ (5 mol%), Xantphos (7.5 mol %), KOAc (2.0 equiv) and additive (0–1.0 equiv) under air, the mixture was then evacuated and backfilled with N_2 for three times. **1a** (0.4 mmol, 1.0 equiv), **2a** (2.0 equiv) and DCE (3 mL) were added subsequently. The reaction mixture was heated to 80 °C (oil bath). After stirring for 16 h, the reaction mixture was cooled to room temperature and diluted with ethyl acetate (2 mL). The yield was determined by ^{19}F NMR using fluorobenzene as an internal standard and number in parenthesis is isolated yield.

Radical clock experiments

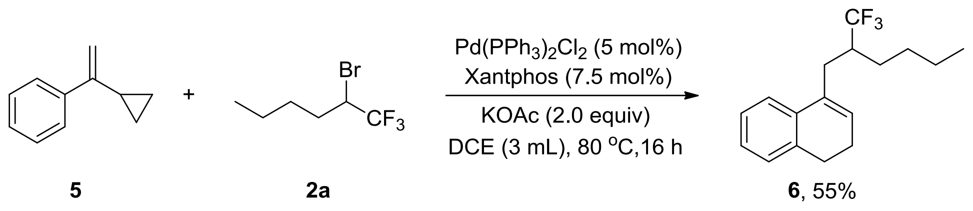

To a 25 mL of Schlenk tube were added $PdCl_2(PPh_3)_2$ (5 mol %), Xantphos (7.5 mol %) and KOAc (2.0 equiv) under air, the mixture was then evacuated and backfilled with N_2 for three times. **5** (0.4

mmol), **2a** (2.0 equiv) and DCE (3 mL) were added subsequently. The reaction mixture was heated to 80 °C (oil bath). After stirring for 16 h, the reaction mixture was cooled to room temperature and diluted with ethyl acetate (2 mL). The reaction mixture was filtered with a pad of cellite. The filtrate was concentrated, and the residue was purified with silica gel chromatography (Petroleum ether) to give product **6** (62 mg, 55%) as a colorless oil. ^{1}H NMR (400 MHz, $CDCl_3$) δ 7.25 – 7.15 (m, 4H), 5.94 (t, *J* = 4.4 Hz, 1H), 2.96 (d, *J* = 11.2 Hz, 1H), 2.75 (t, *J* = 7.8 Hz, 2H), 2.41 – 2.21 (m, 4H), 1.65 – 1.44 (m, 2H), 1.43 – 1.33 (m, 1H), 1.32 – 1.19 (m, 3H), 0.85 (t, *J* = 7.2 Hz, 3H). ^{19}F NMR (376 MHz, $CDCl_3$) δ -70.2 (d, *J* = 9.0 Hz). ^{13}C NMR (101 MHz, $CDCl_3$) δ 137.0, 133.8, 133.0, 128.7 (q, *J* = 282.0 Hz), 128.1, 127.8, 126.9, 126.5, 122.4, 40.8 (q, J = 24.5 Hz), 32.0 (q, *J* = 2.8 Hz), 28.9, 28.3, 27.4 (q, *J* = 1.9 Hz), 23.2, 22.7, 13.8. MS (EI): m/z (%) 282 (M^+), 129 (100). HRMS: Calculated for $C_{17}H_{21}F_3$: 282.1595; Found: 282.1594.

Preparation of secondary fluoroalkylated alkyl halides.

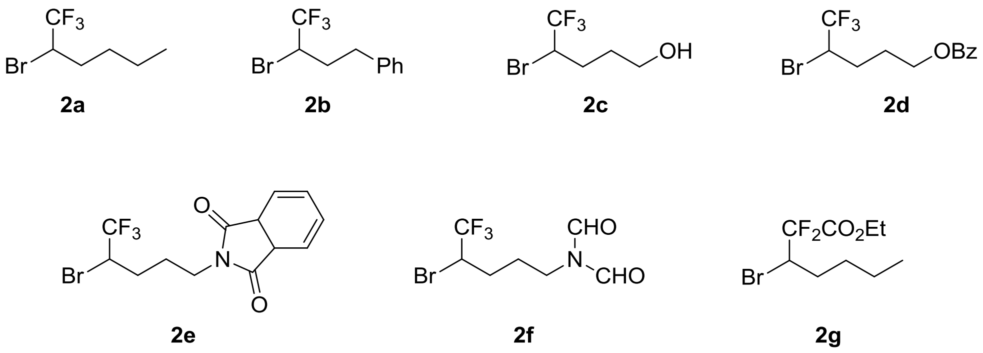

General procedure for the preparation of secondary fluoroalkylated alkyl bromides.

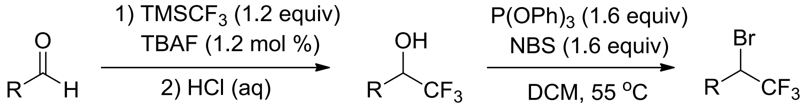

Trifluoromethylation of the aldehyde. [1] A solution of TBAF (1.0 M in THF, 1.8 mL, 1.8 mmol, 0.012 equiv) was added over 20 min to a solution of aldehyde (150 mmol) and trifluoromethyltrimethylsilane (26.6 mL, 180 mmol, 1.2 equiv) in anhydrous THF (400 mL) at 0 °C (Caution: very exothermic!). After stirring for 30 min at 0 °C, the reaction mixture was allowed to warm to room temperature, and stirred for 2 hours at room temperature. An aqueous solution of 1 N

HCl (200 mL) was added slowly, and the reaction mixture was allowed to stir at room temperature for another 2 hours. Then, the mixture was extracted with ethyl acetate (3 × 150 mL), and the combined organic layers were dried over anhydrous Na_2SO_4, filtered, and concentrated. The crude product was purified with flash chromatography on silica gel to give trifluoromethylated alcohol.

Bromination of the alcohol. [2] A solution of triphenylphosphite (74.5 g, 63.0 mL, 1.6 equiv) in DCM (50 mL) was added over 30 min to a solution of *N*-bromosuccinimide (42.7 g, 240 mmol, 1.6 equiv) in DCM (300 mL) at 0 °C. Then a solution of the prepared alcohol (150 mmol) in DCM (100 mL) was added over 20 min to the mixture at 0 °C. The reaction mixture was stirred at 0 °C for 20 min, and then allowed to heat to 55 °C and stirred for 12 h. The reaction mixture was then cooled to room temperature and concentrated. The residue was purified by flash chromatography on silica gel to give product **2**. For compound **2a**, the product was purified by reduced pressure distillation of the reaction mixture.

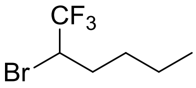

2a

2-Bromo-1,1,1-trifluorohexane (2a). The product as a colorless oil (13.1 g, 40% yield, 2 steps) was purified by reduced pressure distillation. ^{1}H NMR (400 MHz, $CDCl_3$) δ 4.12 – 3.99 (m, 1H), 2.12 – 1.97 (m, 1H), 1.92 – 1.79 (m, 1H), 1.71 – 1.57 (m, 1H), 1.48 – 1.27 (m, 3H), 0.94 (t, *J* = 7.2 Hz, 3H). ^{19}F NMR (376 MHz, $CDCl_3$) δ -72.4 (d, *J* = 7.2 Hz). ^{13}C NMR (126 MHz, $CDCl_3$) δ 124.1 (q, *J* = 277.9 Hz), 47.7 (q, *J* = 32.5 Hz), 31.1 (q, *J* = 1.4 Hz), 28.9, 21.8, 13.7. MS (EI): m/z (%) 218 (M^+), 203 (100). HRMS: Calculated for $C_6H_{10}BrF_3$: 217.9918; Found: 217.9927.

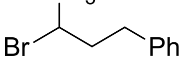

2b

(3-Bromo-4,4,4-trifluorobutyl)benzene (2b). The product as a light yellow oil (20.4 g, 51% yield, 2 steps) was purified with silica gel chromatography (petroleum ether). This compound is known.[2] ^{1}H NMR (400 MHz, $CDCl_3$) δ 7.31 (t, *J* = 7.2 Hz, 2H), 7.25 – 7.20 (m, 3H), 4.05 – 3.89 (m, 1H), 3.07 – 2.92 (m, 1H), 2.81 – 2.67 (m, 1H), 2.40 – 2.27 (m, 1H), 2.25 – 2.10 (m, 1H). ^{19}F NMR (376 MHz,

CDCl3) δ -72.2 (d, *J* = 7.1 Hz). ^{13}C NMR (101 MHz, CDCl3) δ 139.2, 128.8, 128.5, 126.7, δ 124.1 (q, *J* = 278.7 Hz), 46.7 (q, *J* = 32.8 Hz), 32.9, 32.5.

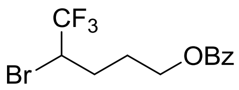

2d

4-Bromo-5,5,5-trifluoropentyl benzoate (2d). The product as a light yellow oil (17.5 g, 30% yield, 2 steps) was synthesized according to the literature.[1] ^{1}H NMR (400 MHz, CDCl3) δ 8.04 (d, *J* = 7.6 Hz, 2H), 7.57 (t, *J* = 7.4 Hz, 1H), 7.45 (t, *J* = 7.6 Hz, 2H), 4.47 – 4.31 (m, 2H), 4.25 – 4.13 (m, 1H), 2.36 – 2.21 (m, 1H), 2.16 – 2.10 (m, 1H), 2.08 – 1.88 (m, 2H). ^{19}F NMR (376 MHz, CDCl3) δ -72.3 (d, *J* = 7.1 Hz). ^{13}C NMR (101 MHz, CDCl3) δ 166.4, 133.1, 129.9, 129.5, 128.4, 123.9 (q, *J* = 278.8 Hz), 63.5, 46.9 (q, *J* = 32.9 Hz), 28.4 (q, *J* = 1.5 Hz), 26.2.

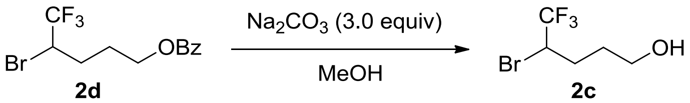

4-Bromo-5,5,5-trifluoropentan-1-ol (2c). The product as a light yellow oil (12.1 g, 88% yield) was synthesized according to the literature.[1] ^{1}H NMR (400 MHz, CDCl3) δ 4.21 – 4.10 (m, 1H), 3.75 – 3.63 (m, 2H), 2.30 – 2.13 (m, 2H), 2.00 – 1.82 (m, 2H), 1.75 – 1.59 (m, 1H). ^{19}F NMR (376 MHz, CDCl3) δ -72.4 (d, *J* = 6.9 Hz). ^{13}C NMR (101 MHz, CDCl3) δ 124.0 (q, *J* = 278.7 Hz), 61.5, 47.5 (q, *J* = 32.8 Hz), 29.6, 28.2 (d, *J* = 1.6 Hz).

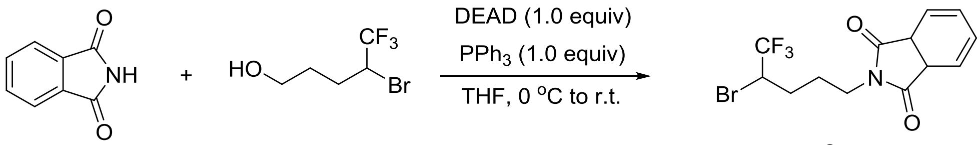

2-(4-Bromo-5,5,5-trifluoropentyl)-3a,7a-dihydro-1*H*-isoindole-1,3(2*H*)-dione (2e). The product as a yellow oil (882 mg, 84% yield) was synthesized according to the literature.[1] ^{1}H NMR (400 MHz, CDCl3) δ 7.84 – 7.73 (m, 2H), 7.72 – 7.63 (m, 2H), 4.28 – 4.08 (m, 1H), 3.68 (t, *J* = 6.2 Hz, 2H), 2.17 – 1.93 (m, 2H), 1.93 – 1.69 (m, 2H). ^{19}F NMR (376 MHz, CDCl3) δ -72.3 (d, *J* = 7.1 Hz). ^{13}C NMR (126 MHz, CDCl3) δ 168.3, 134.1, 131.9, 123.8 (q, *J* = 278.2 Hz), 123.3, 46.6 (q, *J* = 32.8 Hz), 36.5, 28.6, 26.0.

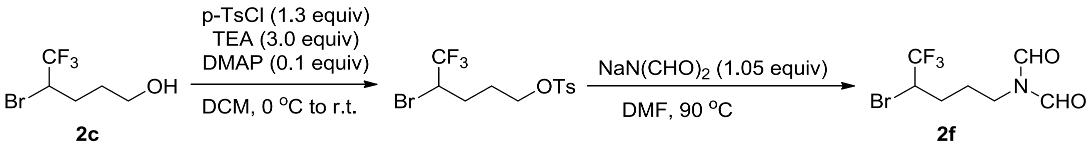

***N*-(4-Bromo-5,5,5-trifluoropentyl)-N-formylformamide (2f)**[3] *p*-TsCl (1.49 g, 7.8 equiv) was slowly added to a solution of **2c** (1.33 g, 6.0 mmol), DMAP (74 mg, 0.1 equiv) and TEA (2.5 mL, 18 mmol, 3.0 equiv) in DCM (15 mL) at 0 °C. After stirring at 0 °C for 1 hour, the reaction mixture was allowed to warm to room temperature. After stirring at room temperature for another 5 hours, the reaction mixture was washed with water (2 × 10 mL), and the aqueous layers were extracted with DCM (15 mL). The combined organic layers were dried over anhydrous Na_2SO_4, filtered, and concentrated. The residue was purified with flash chromatography on silica gel (petroleum ether / ethyl acetate 10:1) to give a light yellow oil. The crude product and diformylimide sodium salt (599 mg, 1.05 equiv) were dissolved in DMF (5 mL), then the reaction mixture was allowed to heated to 90 °C and stirred for 8 h. The reaction mixture was then cooled to room temperature and the solvent was evaporated to give a solid residue. The solid residue was washed with $CHCl_3$ (3 × 10 mL), and the combined organic layers were dried over anhydrous Na_2SO_4, filtered, and concentrated. The residue was purified with flash chromatography on silica gel (petroleum ether/ethyl acetate 5:1) to give **2f** as a light yellow oil (1.07 g, 65% yield). ^{1}H NMR (400 MHz, $CDCl_3$) δ 8.87 (s, 2H), 4.19 – 4.08 (m, 1H), 3.70 (t, *J* = 6.4 Hz, 2H), 2.07 – 1.81 (m, 3H), 1.80 – 1.69 (m, 1H). ^{19}F NMR (376 MHz, $CDCl_3$) δ -72.3 (d, *J* = 7.7 Hz). ^{13}C NMR (101 MHz, $CDCl_3$) δ 164.1, 123.8 (q, *J* = 278.9 Hz), 46.4 (q, *J* = 33.0 Hz), 37.3, 28.5, 24.9. MS (EI): m/z (%) 275 (M^+), 58 (100). HRMS: Calculated for $C_7H_9BrF_3NO_2$: 274.9769; Found: 274.9774.

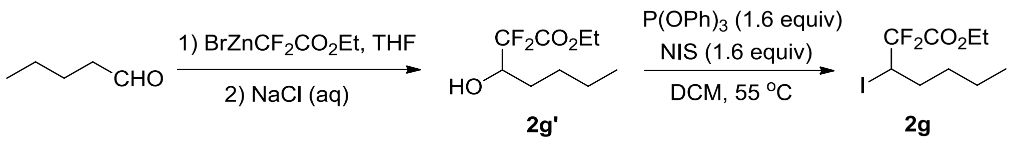

Ethyl 2, 2-difluoro-3-iodoheptanoate (2g)[2] Under the atmosphere of argon, preactivated zinc powder (3.33 g, 51.0 mmol, 3.0 equiv) was added into anhydrous THF (10 mL) at room temperature, followed by 2, 2, 2-trifluoroacetic acid (0.19 mL, 2.55 mmol, 0.15 equiv). After the mixture was stirred for 10 min at room temperature, anhydrous THF (15 mL) was added and then ethyl 2-bromo-2, 2 -difluoroacetate (3.27 mL, 25.5 mmol, 1.5 equiv) was added by dropwise. After stirring

for 15 min, another anhydrous THF (20 mL) was added and the reaction was then stirred for another 3 hours. The supernatant of prepared zinc reagents was transferred into a solution of valeraldehyde (1.81 mL, 17 mmol, 1.0 equiv) in anhydrous THF (10 mL). After stirring for 12 hours at room temperature, the reaction was quenched by saturated NaCl solution (100 mL) and extracted with ethyl acetate (3 × 50 mL). The combined organic layers were dried over anhydrous Na_2SO_4, filtered, and concentrated. The residue was purified with flash chromatography on silica gel (petroleum ether / ethyl acetate = 10/1) to give compound **2g′**, as a light yellow oil (1.72 g, 48% yield).

The preparation of compound **2g** from alcohol **2g′** is according to the procedure of the preparation of compound **2b**. The product as a light yellow oil (1.3 g, 50 % yield) was purified with silica gel chromatography (petroleum ether/ethyl acetate 10:1). *N*-iodosuccinimide was used instead of *N*-bromosuccinimide. ^{1}H NMR (400 MHz, $CDCl_3$) δ 4.37 (q, *J* = 7.2 Hz, 2H), 4.33 – 4.23 (m, 1H), 1.90 – 1.74 (m, 2H), 1.68 – 1.57 (m, 1H), 1.47 – 1.27 (m, 6H), 0.93 (t, *J* = 6.6 Hz, 3H). ^{19}F NMR (376 MHz, $CDCl_3$) δ -103.1 (dd, *J* = 252.3 Hz, 12.2 Hz), -106.65 (dd, *J* = 252.3 Hz, 12.0 Hz). ^{13}C NMR (101 MHz, $CDCl_3$) δ 162.0 (t, *J* = 32.4 Hz), 114.1 (t, *J* = 255.6 Hz), 63.3, 31.2, 31.1, 28.8 (t, *J* = 25.0 Hz), 21.6, 13.9, 13.8. MS (EI): m/z (%) 320 (M^+), 85 (100). HRMS: Calculated for $C_9H_{15}F_2IO_2$: 320.0085; Found: 320.0094.

General Procedure for Pd-Catalyzed cross-coupling of secondary fluorinated alkyl halides with alkenes.

To a 25 mL of Schlenk tube were added $PdCl_2(PPh_3)_2$ (5 mol %), Xantphos (7.5 mol %) and KOAc (2.0 equiv) under air. The mixture was then evacuated and backfilled with N_2 for three times. Alkene **1** (0.4 mmol), secondary fluoroalkylated alkyl halide **2** (0.8 mmol, 2.0 equiv) and DCE (3 mL) were added subsequently. The reaction mixture was heated to 80 °C (oil bath). After stirring for 16 h, the reaction mixture was cooled to room temperature, diluted with EtOAc and filtered with a pad of cellite. The filtrate was concentrated, and the residue was purified with silica gel chromatography to give product.

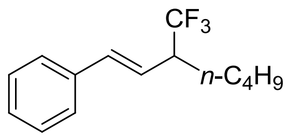

(*E*)-(3-(Trifluoromethyl)hept-1-en-1-yl)benzene (3a). The product (86 mg, 88% yield) as a colorless oil was purified by silica gel chromatography (petroleum ether). ^{1}H NMR (500 MHz, $CDCl_3$) δ 7.42 (d, *J* = 7.5 Hz, 2H), 7.36 (t, *J* = 7.5 Hz, 2H), 7.29 (t, *J* = 7.3 Hz, 1H), 6.58 (d, *J* = 16.0 Hz, 1H), 5.99 (dd, *J* = 16.0 Hz, 9.5 Hz, 1H), 2.94 – 2.78 (m, 1H), 1.90 – 1.80 (m, 1H), 1.63 – 1.53 (m, 1H), 1.48 – 1.24 (m, 4H), 0.93 (t, *J* = 7.0 Hz, 3H). ^{19}F NMR (376 MHz, $CDCl_3$) δ -70.9 (d, *J* = 8.6 Hz). ^{13}C NMR (126 MHz, $CDCl_3$) δ 136.3, 135.6, 128.6, 128.0, 127.0 (q, *J* = 280.4 Hz), 126.5, 123.1 (q, *J* = 2.6 Hz), 47.91 (q, *J* = 26.5 Hz), 28.7, 27.6 (q, *J* = 2.0 Hz), 22.4, 13.8. MS (EI): m/z (%) 242 (M^+), 242 (100). HRMS: Calculated for $C_{14}H_{17}F_3$: 242.1282; Found: 242.1285.

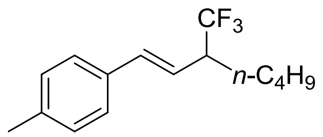

(*E*)-1-Methyl-4-(3-(trifluoromethyl)hept-1-en-1-yl)benzene (3b). The product (94 mg, 91% yield) as colorless oil was purified by silica gel chromatography (petroleum ether). ^{1}H NMR (400 MHz, $CDCl_3$) δ 7.34 (d, *J* = 8.0 Hz, 2H), 7.18 (d, *J* = 8.0 Hz, 2H), 6.56 (d, *J* = 15.6 Hz, 1H), 5.95 (dd, *J* = 16.0 Hz, 9.2 Hz, 1H), 3.01 – 2.68 (m, 1H), 2.39 (s, 3H), 1.98 – 1.75 (m, 1H), 1.63 – 1.53 (m, 1H), 1.48 – 1.21 (m, 4H), 0.94 (t, *J* = 6.8 Hz, 3H). ^{19}F NMR (376 MHz, $CDCl_3$) δ -70.9 (d, *J* = 9.0 Hz). ^{13}C NMR (126 MHz, $CDCl_3$) δ 137.9, 135.5, 133.6, 129.3, 127.1 (q, *J* = 280.4 Hz), 126.4, 122.08 (q, *J* = 2.6 Hz), 47.9 (q, *J* = 26.5 Hz), 28.7, 27.7 (q, *J* = 2.0 Hz), 22.4, 21.2, 13.8. MS (EI): m/z (%) 256 (M^+), 105 (100). HRMS: Calculated for $C_{15}H_{19}F_3$: 256.1439; Found: 256.1433.

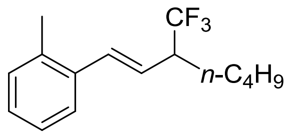

(*E*)-1-Methyl-2-(3-(trifluoromethyl)hept-1-en-1-yl)benzene (3c). The product (93 mg, 90% yield) as a colorless oil was purified by silica gel chromatography (petroleum ether). ^{1}H NMR (400 MHz, $CDCl_3$) δ 7.51 – 7.46 (m, 1H), 7.25 – 7.19 (m, 3H), 6.82 (d, *J* = 15.6 Hz, 1H), 5.87 (dd, *J* = 15.8 Hz, 9.4 Hz, 1H), 3.01 – 2.78 (m, 1H), 2.40 (s, 3H), 1.92 – 1.83 (m, 1H), 1.67 – 1.55 (m, 1H), 1.51 – 1.29

(m, 4H), 0.96 (t, *J* = 6.8 Hz, 3H). ^{19}F NMR (376 MHz, $CDCl_3$) δ -70.9 (d, *J* = 9.0 Hz). ^{13}C NMR (101 MHz, $CDCl_3$) δ 135.7, 135.4, 133.7, 130.3, 127.9, 127.0 (q, *J* = 280.0 Hz), 126.2, 125.9, 124.7 (q, *J* = 2.5 Hz), 48.1 (q, *J* = 26.4 Hz), 28.7, 27.5 (q, *J* = 2.0 Hz), 22.3, 19.7, 13.8. MS (EI): m/z (%) 256 (M^+), 129 (100). HRMS: Calculated for $C_{15}H_{19}F_3$: 256.1439; Found: 256.1428.

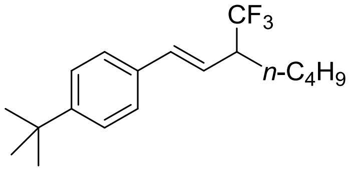

(*E*)-1-(*tert*-Butyl)-4-(3-(trifluoromethyl)hept-1-en-1-yl)benzene (3d). The product (108 mg, 90% yield) as a colorless oil was purified with silica gel chromatography (petroleum ether). ^{1}H NMR (400 MHz, $CDCl_3$) δ 7.42 – 7.34 (m, 4H), 6.55 (d, *J* = 16.0 Hz, 1H), 5.95 (dd, *J* = 16.0 Hz, 9.2 Hz, 1H), 2.96 – 2.72 (m, 1H), 1.87 – 1.76 (m, 1H), 1.65 – 1.48 (m, 1H), 1.48 – 1.22 (m, 13H), 0.91 (t, *J* = 6.8 Hz, 3H). ^{19}F NMR (376 MHz, $CDCl_3$) δ -70.8 (d, *J* = 8.6 Hz). ^{13}C NMR (101 MHz, $CDCl_3$) δ 151.2, 135.4, 133.6, 127.1 (q, *J* = 280.0 Hz), 126.2, 125.6, 122.31 (q, *J* = 3.1 Hz), 48.0 (q, *J* = 26.6 Hz), 34.6, 31.2, 28.7, 27.7 (q, *J* = 1.9 Hz), 22.4, 13.8. MS (EI): m/z (%) 298 (M^+), 283 (100). HRMS: Calculated for $C_{18}H_{25}F_3$: 298.1908; Found: 298.1902.

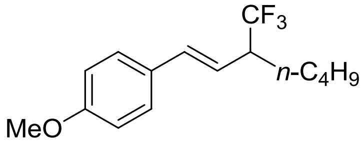

(*E*)-1-Methoxy-4-(3-(trifluoromethyl)hept-1-en-1-yl)benzene (3e). The product (106 mg, 97% yield) as a colorless oil was purified with silica gel chromatography (petroleum ether/ethyl acetate 20/:1). ^{1}H NMR (400 MHz, $CDCl_3$) δ 7.37 (d, *J* = 8.8 Hz, 2H), 6.90 (d, *J* = 8.8 Hz, 2H), 6.53 (d, *J* = 16.0 Hz, 1H), 5.86 (dd, *J* = 15.6 Hz, 9.6 Hz, 1H), 3.84 (s, 3H), 2.90 – 2.74 (m, 1H), 1.92 – 1.77 (m, 1H), 1.62 – 1.52 (m, 1H), 1.49 – 1.25 (m, 4H), 0.94 (t, *J* = 6.9 Hz, 3H). ^{19}F NMR (376 MHz, $CDCl_3$) δ -70.9 (d, *J* = 9.0 Hz). ^{13}C NMR (101 MHz, $CDCl_3$) δ 159.5, 135.0, 129.1, 127.1 (q, *J* = 280.0 Hz), 127.1, 120.8 (q, *J* = 2.6 Hz), 114.0, 55.2, 47.9 (q, *J* = 26.5 Hz), 28.7, 27.7 (q, *J* = 2.0 Hz), 22.4 13.8. MS (EI): m/z (%) 272 (M^+), 121 (100). HRMS: Calculated for $C_{15}H_{19}F_3O$: 272.1388; Found: 272.1393.

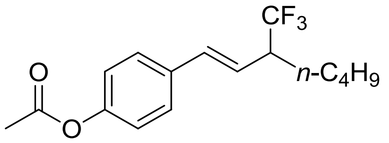

(*E*)-4-(3-(Trifluoromethyl)hept-1-en-1-yl)phenyl acetate (3f). The product (114 mg, 95% yield) as a pale yellow oil was purified with silica gel chromatography (petroleum ether/dichloromethane 2:1). ^{1}H NMR (500 MHz, $CDCl_3$) δ 7.42 (d, *J* = 8.5 Hz, 2H), 7.08 (d, *J* = 8.6 Hz, 2H), 6.56 (d, *J* = 15.8 Hz, 1H), 5.94 (dd, *J* = 15.8 Hz, 9.2 Hz, 1H), 2.92 – 2.74 (m, 1H), 2.31 (s, 3H), 1.96 – 1.69 (m, 1H), 1.60 – 1.53 (m, 1H), 1.48 – 1.22 (m, 4H), 0.93 (t, *J* = 7.0 Hz, 3H). ^{19}F NMR (376 MHz, $CDCl_3$) δ -70.9 (d, *J* = 8.6 Hz). ^{13}C NMR (126 MHz, $CDCl_3$) δ 169.3, 150.3, 134.6, 134.1, 127.4, 126.9 (q, *J* = 280.3 Hz), 123.3 (q, *J* = 2.5 Hz), 121.7, 47.8 (q, *J* = 26.5 Hz), 28.6, 27.5 (q, *J* = 1.8 Hz), 22.3, 21.0, 13.7. MS (EI): m/z (%) 300 (M^+), 258 (100). HRMS: Calculated for $C_{16}H_{19}F_3O_2$: 300.1337; Found: 300.1338.

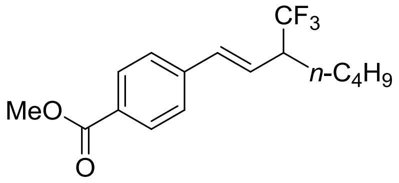

Methyl (*E*)-4-(3-(trifluoromethyl)hept-1-en-1-yl)benzoate (3g). The product (76 mg, 63% yield) as a colorless oil was purified with silica gel chromatography (petroleum ether/ethyl acetate 10:1). ^{1}H NMR (400 MHz, $CDCl_3$) δ 8.01 (d, *J* = 8.4 Hz, 2H), 7.45 (d, *J* = 8.0 Hz, 2H), 6.59 (d, *J* = 16.0 Hz, 1H), 6.09 (dd, *J* = 16.0 Hz, 9.4 Hz, 1H), 3.91 (s, 3H), 2.92 – 2.77 (m, 1H), 1.91 – 1.76 (m, 1H), 1.62 – 1.51 (m, 1H), 1.45 – 1.24 (m, 4H), 0.90 (t, *J* = 7.0 Hz, 3H). ^{19}F NMR (376 MHz, $CDCl_3$) δ -70.7 (d, *J* = 8.6 Hz). ^{13}C NMR (101 MHz, $CDCl_3$) δ 166.7, 140.6, 134.7, 129.9, 129.4, 126.8 (q, *J* = 281.1 Hz), 126.3, 125.9 (q, *J* = 2.5 Hz), 52.1, 47.9 (q, *J* = 26.6 Hz), 28.7, 27.5 (q, *J* = 1.9 Hz), 22.3, 13.8. MS (EI): m/z (%) 300 (M^+), 191 (100). HRMS: Calculated for $C_{16}H_{19}F_3O_2$: 300.1337; Found: 300.1333.

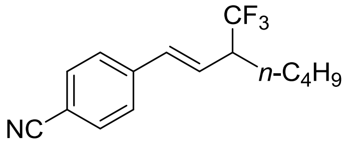

(*E*)-4-(3-(Trifluoromethyl)hept-1-en-1-yl)benzonitrile (3h). The product (77 mg, 72% yield) as a

colorless oil was purified with silica gel chromatography (petroleum ether/ethyl acetate 5:1). ^{1}H NMR (400 MHz, $CDCl_3$) ^{1}H NMR (400 MHz, $CDCl_3$) δ 7.61 (d, *J* = 8.0 Hz, 2H), 7.47 (d, *J* = 8.0 Hz, 2H), 6.57 (d, *J* = 15.8 Hz, 1H), 6.10 (dd, *J* = 15.8 Hz, 9.4 Hz, 1H), 2.97 – 2.74 (m, 1H), 1.92 – 1.74 (m, 1H), 1.61 – 1.52 (m, 1H), 1.43 – 1.22 (m, 4H), 0.90 (t, *J* = 6.4 Hz, 3H). ^{19}F NMR (376 MHz, $CDCl_3$) δ -70.6 (d, *J* = 8.7 Hz). ^{13}C NMR (101 MHz, $CDCl_3$) δ 140.6, 134.0, 132.4, 127.2 (q, *J* = 2.5 Hz), 126.9, 126.7 (q, *J* = 281.2 Hz), 118.7, 111.3, 47.9 (q, *J* = 26.8 Hz), 28.7, 27.4 (q, *J* = 2.0 Hz), 22.3, 13.7. MS (EI): m/z (%) 267 (M^+), 129 (100). HRMS: Calculated for $C_{15}H_{16}F_3N$: 267.1235; Found: 267.1241.

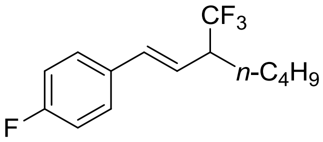

(*E*)-1-Fluoro-4-(3-(trifluoromethyl)hept-1-en-1-yl)benzene (3i). The product (90 mg, 86% yield) as a colorless oil was purified with silica gel chromatography (petroleum ether). ^{1}H NMR (400 MHz, $CDCl_3$) δ 7.46 – 7.32 (m, 2H), 7.03 (t, *J* = 8.6 Hz, 2H), 6.53 (d, *J* = 15.8 Hz, 1H), 5.90 (dd, *J* = 15.8 Hz, 9.3 Hz, 1H), 2.92 – 2.71 (m, 1H), 1.93 – 1.76 (m, 1H), 1.62 – 1.50 (m, 1H), 1.49 – 1.20 (m, 4H), 0.92 (t, *J* = 6.8 Hz, 3H). ^{19}F NMR (376 MHz, $CDCl_3$) δ -70.9 (d, *J* = 9.0 Hz, 3F), -113.8 – -113.9 (m, 1F). ^{13}C NMR (126 MHz, $CDCl_3$) δ 162.6 (d, *J* = 248.0 Hz), 134.4, 132.5 (d, *J* = 3.4 Hz), 128.0 (d, *J* = 8.0 Hz) 127.0 (q, *J* = 280.4 Hz), 123.0-122.8 (m), 115.5 (d, *J* = 21.6 Hz), 47.9 (q, *J* = 26.6 Hz), 28.7, 27.6 (q, *J* = 2.0 Hz), 22.4, 13.8. MS (EI): m/z (%) 260 (M^+), 122 (100). HRMS: Calculated for $C_{14}H_{16}$F4: 260.1188; Found: 260.1184.

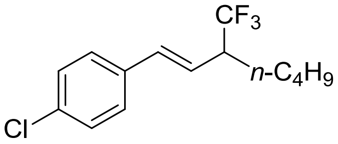

(*E*)-1-Chloro-4-(3-(trifluoromethyl)hept-1-en-1-yl)benzene (3j). The product (89 mg, 80% yield) as a white solid was purified with silica gel chromatography (petroleum ether). m.p. 48~50 °C. ^{1}H NMR (500 MHz, $CDCl_3$) δ 7.34 – 7.28 (m, 4H), 6.51 (d, *J* = 16.0 Hz, 1H), 5.94 (dd, *J* = 15.8 Hz, 9.2 Hz, 1H), 2.91 – 2.74 (m, 1H), 1.86 – 1.77 (m, 1H), 1.61 – 1.50 (m, 1H), 1.44 – 1.22 (m, 4H), 0.91 (t, *J* = 7.0 Hz, 3H). ^{19}F NMR (376 MHz, $CDCl_3$) δ -70.8 (d, J = 9.0 Hz). ^{13}C NMR (126 MHz, $CDCl_3$) δ

134.8, 134.4, 133.7, 128.8, 127.7, 126.9 (q, *J* = 280.4 Hz), 123.9 (q, *J* = 2.6 Hz), 47.9 (q, *J* = 26.6 Hz), 28.7, 27.6 (q, *J* = 2.0 Hz), 22.4, 13.8. MS (EI): m/z (%) 276 (M^+), 138 (100). HRMS: Calculated for $C_{14}H_{16}ClF_3$: 276.0893; Found: 276.0890.

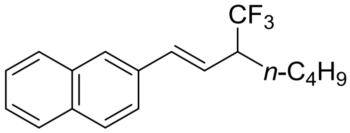

(*E*)-2-(3-(Trifluoromethyl)hept-1-en-1-yl)naphthalene (3k). The product (95 mg, 81% yield) as a white solid was purified with silica gel chromatography (petroleum ether). m.p. 86~88 ^oC. ^1H NMR (400 MHz, $CDCl_3$) δ 7.85 – 7.80 (m, 3H), 7.76 (s, 1H), 7.62 (d, *J* = 8.0 Hz, 1H), 7.53 – 7.44 (m, 2H), 6.73 (d, *J* = 15.6 Hz, 1H), 6.11 (dd, *J* = 15.6 Hz, 9.2 Hz, 1H), 3.00 – 2.81 (m, 1H), 1.99 – 1.79 (m, 1H), 1.73 – 1.55 (m, 1H), 1.51 – 1.21 (m, 4H), 0.93 (t, *J* = 6.8 Hz, 3H). ^{19}F NMR (376 MHz, $CDCl_3$) δ -70.8 (d, *J* = 8.6 Hz). ^{13}C NMR (126 MHz, $CDCl_3$) δ 135.7, 133.8, 133.5, 133.1, 128.3, 128.0, 127.7, 127.1 (q, *J* = 280.4 Hz), 126.5, 126.4, 126.1, 123.5 (q, *J* = 2.5 Hz), 123.4, 48.0 (q, *J* = 26.5 Hz), 28.7, 27.7 (q, *J* = 1.9 Hz), 22.4, 13.8. MS (EI): m/z (%) 292 (M^+), 199 (100). HRMS: Calculated for $C_{18}H_{19}F_3$: 292.1439; Found: 292.1444.

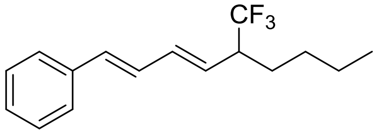

((1*E*,3*E*)-5-(Trifluoromethyl)nona-1,3-dien-1-yl)benzene (3l). The product (90 mg, 83% yield) as a white semi-solid was purified with silica gel chromatography (petroleum ether). ^1H NMR (400 MHz, $CDCl_3$) δ 7.44 (d, *J* = 7.6 Hz, 2H), 7.36 (t, *J* = 7.6 Hz, 2H), 7.28 (t, *J* = 7.2 Hz, 1H), 6.81 (dd, *J* = 15.6 Hz, 10.4 Hz, 1H), 6.60 (d, *J* = 15.6 Hz, 1H), 6.40 (dd, *J* = 15.2 Hz, 10.4 Hz, 1H), 5.60 (dd, *J* = 15.2 Hz, 9.2 Hz, 1H), 2.90 – 2.66 (m, 1H), 1.92 – 1.75 (m, 1H), 1.60 – 1.48 (m, 1H), 1.45 – 1.21 (m, 4H), 0.95 (t, *J* = 6.8 Hz, 3H). ^{19}F NMR (376 MHz, $CDCl_3$) δ -70.8 (d, *J* = 9.0 Hz). ^{13}C NMR (101 MHz, $CDCl_3$) δ 136.9, 135.9, 133.2, 128.6, 127.9, 127.8, 126.97 (q, *J* = 281.1 Hz), 126.95 (q, *J* = 2.4 Hz), 126.4, 47.6 (q, *J* = 26.4 Hz), 28.7, 27.6 (q, *J* = 1.9 Hz), 22.4, 13.8. MS (EI): m/z (%) 268 (M^+), 104 (100). HRMS: Calculated for $C_{16}H_{19}F_3$: 268.1439; Found: 268.1432.

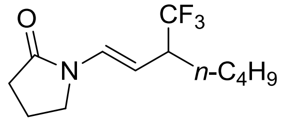

(*E*)-1-(3-(Trifluoromethyl)hept-1-en-1-yl)pyrrolidin-2-one (3m). The reaction was conducted at 100 °C. The product (61 mg, 61% yield) as a pale yellow solid was purified with silica gel chromatography (petroleum ether/ethyl acetate 2:1). m.p. 66~69 °C. ^{1}H NMR (400 MHz, $CDCl_3$) δ 7.00 (d, *J* = 14.4 Hz, 1H), 4.62 (dd, *J* = 14.4 Hz, 9.6 Hz, 1H), 3.51 (t, *J* = 7.2 Hz, 2H), 2.76 – 2.55 (m, 1H), 2.48 (t, *J* = 8.2 Hz, 2H), 2.19 – 2.03 (m, 2H), 1.80 – 1.66 (m, 1H), 1.48 – 1.14 (m, 5H), 0.86 (t, *J* = 6.8 Hz, 3H). ^{19}F NMR (376 MHz, $CDCl_3$) δ -71.6 (d, *J* = 8.6 Hz). ^{13}C NMR (101 MHz, $CDCl_3$) δ 173.3, 128.2, 127.0 (q, *J* = 280.9 Hz), 104.6 (q, *J* = 2.8 Hz), 45.6 (q, *J* = 27.0 Hz), 45.0, 31.0, 28.6, 27.8 (q, *J* = 1.9 Hz), 22.2, 17.3, 13.8. MS (EI): m/z (%) 249 (M^+), 86 (100). HRMS: Calculated for $C_{12}H_{18}F_3NO$: 249.1340; Found: 249.1332.

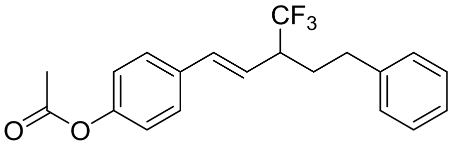

(*E*)-4-(5-phenyl-3-(trifluoromethyl)pent-1-en-1-yl)phenyl acetate (4a). The product (129 mg, 92% yield) as a colorless oil was purified with silica gel chromatography (petroleum ether/ethyl acetate 10:1). ^{1}H NMR (400 MHz, $CDCl_3$) δ 7.48 (d, *J* = 8.4 Hz, 2H), 7.37 (t, *J* = 7.2 Hz, 2H), 7.28 (t, *J* = 7.2 Hz, 1H), 7.24 (d, *J* = 7.2 Hz, 2H), 7.14 (d, *J* = 8.4 Hz, 2H), 6.61 (d, *J* = 15.8 Hz, 1H), 6.03 (dd, *J* = 15.8 Hz, 9.2 Hz, 1H), 3.00 – 2.79 (m, 2H), 2.69 – 2.58 (m, 1H), 2.37 (s, 3H), 2.30 – 2.17 (m, 1H), 2.02 – 1.89 (m, 1H). ^{19}F NMR (376 MHz, $CDCl_3$) δ -70.7 (d, *J* = 8.6 Hz). ^{13}C NMR (101 MHz, $CDCl_3$) δ 169.4, 150.5, 140.6, 135.3, 134.0, 128.5, 128.4, 127.5, 126.8 (q, *J* = 280.6 Hz), 126.2, 122.8 (q, *J* = 2.4 Hz), 121.8, 47.1 (q, *J* = 26.8 Hz), 32.4, 29.3, 21.1. MS (EI): m/z (%) 348 (M^+), 107 (100). HRMS: Calculated for $C_{20}H_{19}F_3O_2$: 348.1337; Found: 348.1344.

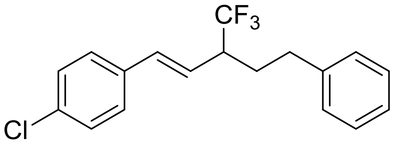

(*E*)-1-Chloro-4-(5-phenyl-3-(trifluoromethyl)pent-1-en-1-yl)benzene (4b). The product (123 mg, 94% yield) as a colorless oil was purified with silica gel chromatography (petroleum ether/ethyl

acetate 10:1). ^{1}H NMR (400 MHz, $CDCl_3$) δ 7.38 – 7.29 (m, 6H), 7.27 – 7.21 (m, 1H), 7.19 (d, *J* = 7.6 Hz, 2H), 6.53 (d, *J* = 16.0 Hz, 1H), 6.00 (dd, *J* = 15.8 Hz, 9.4 Hz, 1H), 2.97 – 2.73 (m, 2H), 2.69 – 2.52 (m, 1H), 2.26 – 2.13 (m, 1H), 2.02 – 1.84 (m, 1H). ^{19}F NMR (376 MHz, $CDCl_3$) δ -70.6 (d, *J* = 8.6 Hz). ^{13}C NMR (101 MHz, $CDCl_3$) δ 140.5, 135.1, 134.7, 133.9, 128.8, 128.6, 128.4, 127.7, 126.8 (q, *J* = 280.7 Hz), 126.3, 123.3 (q, *J* = 2.4 Hz), 47.1 (q, *J* = 26.9 Hz), 32.4, 29.3. MS (EI): m/z (%) 324 (M^+), 91 (100). HRMS: Calculated for $C_{18}H_{16}ClF_3$: 324.0893; Found: 324.0883.

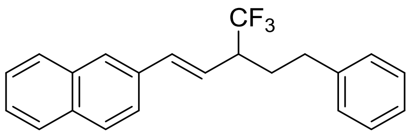

(*E*)-2-(5-Phenyl-3-(trifluoromethyl)pent-1-en-1-yl)naphthalene (4c). The product (123 mg, 90% yield) as a white semi-solid was purified by silica gel chromatography (petroleum ether). ^{1}H NMR (400 MHz, $CDCl_3$) δ 7.84 (d, *J* = 8.8 Hz, 3H), 7.78 (s, 1H), 7.64 (d, *J* = 8.8 Hz, 1H), 7.56 – 7.44 (m, 2H), 7.33 (t, *J* = 7.6 Hz, 2H), 7.25 (d, *J* = 7.2 Hz, 1H), 7.22 (d, *J* = 7.6 Hz, 2H), 6.74 (d, *J* = 15.8 Hz, 1H), 6.15 (dd, *J* = 15.8 Hz, 9.2 Hz, 1H), 3.04 – 2.78 (m, 2H), 2.72 – 2.57 (m, 1H), 2.32 – 2.16 (m, 1H), 2.06 – 1.89 (m, 1H). ^{19}F NMR (376 MHz, $CDCl_3$) δ -70.6 (d, *J* = 9.0 Hz). ^{13}C NMR (101 MHz, $CDCl_3$) δ 140.7, 136.4, 133.6, 133.5, 133.2, 128.6, 128.4, 128.3, 128.0, 127.7, 126.9 (q, *J* = 280.8 Hz), 126.7, 126.4, 126.2, 126.1, 123.4, 122.9 (q, *J* = 2.5 Hz), 47.3 (q, *J* = 26.8 Hz), 32.5, 29.4. MS (EI): m/z (%) 340 (M^+), 141 (100). HRMS: Calculated for $C_{22}H_{19}F_3$: 340.1439; Found: 340.1441.

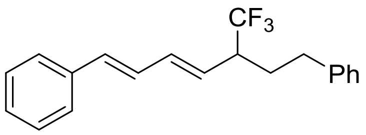

((1*E*, 3*E*)-5-(Trifluoromethyl)hepta-1,3-diene-1,7-diyl)dibenzene (4d). The product (90 mg, 71% yield) as a colorless oil was purified with silica gel chromatography (petroleum ether). ^{1}H NMR (400 MHz, $CDCl_3$) δ 7.47 (d, *J* = 7.4 Hz, 2H), 7.42 – 7.33 (m, 4H), 7.33 – 7.26 (m, 2H), 7.23 (d, *J* = 7.1 Hz, 2H), 6.85 (dd, *J* = 15.6 Hz, 10.4 Hz, 1H), 6.64 (d, *J* = 15.7 Hz, 1H), 6.42 (dd, *J* = 15.2 Hz, 10.4 Hz, 1H), 5.66 (dd, *J* = 15.2 Hz, 9.4 Hz, 1H), 2.94 – 2.76 (m, 2H), 2.65 – 2.57 (m, 1H), 2.24 – 2.12 (m, 1H), 1.95 – 1.84 (m, 1H). ^{19}F NMR (376 MHz, $CDCl_3$) δ -70.6 (d, *J* = 8.6 Hz). ^{13}C NMR (101 MHz, $CDCl_3$) δ 140.7, 136.8, 136.6, 133.5, 128.6, 128.5, 128.4, 127.8, 127.7, 126.8 (q, *J* = 281.1

Hz), 126.5, 126.3 (q, J = 2.5 Hz), 126.2, 46.9 (q, J = 26.8 Hz), 32.4, 29.4 (q, J = 1.7 Hz). MS (EI): m/z (%) 316 (M^+), 212 (100). HRMS: Calculated for $C_{20}H_{19}F_3$: 316.1439; Found: 316.1442.

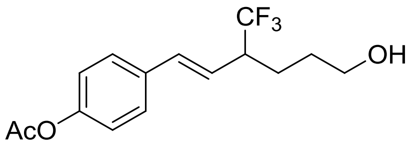

(*E*)-4-(6-Hydroxy-3-(trifluoromethyl)hex-1-en-1-yl)phenyl acetate (4e). The product (118 mg, 97% yield) as a yellow oil was purified with silica gel chromatography (petroleum ether/ethyl acetate 5:1). ^{1}H NMR (400 MHz, $CDCl_3$) δ 7.39 (d, J = 8.4 Hz, 2H), 7.05 (d, J = 8.4 Hz, 2H), 6.55 (d, J = 16.0 Hz, 1H), 5.92 (dd, J = 16.0 Hz, 9.2 Hz, 1H), 3.69 – 3.62 (m, 2H), 2.94 – 2.78 (m, 1H), 2.30 (s, 3H), 1.99 – 1.86 (m, 1H), 1.75 – 1.48 (m, 4H). ^{19}F NMR (376 MHz, $CDCl_3$) δ -70.9 (d, J = 8.6 Hz). ^{13}C NMR (101 MHz, $CDCl_3$) δ 169.4, 150.4, 135.0, 133.9, 127.4, 126.8 (q, J = 280.8 Hz), 123.0 (q, J = 2.5 Hz), 121.8, 62.2, 47.7 (q, J = 26.8 Hz), 29.5, 24.4 (q, J = 1.8 Hz), 21.0. MS (EI): m/z (%) 302 (M^+), 107 (100). HRMS: Calculated for $C_{15}H_{17}F_3O_3$: 302.1130; Found: 302.1126.

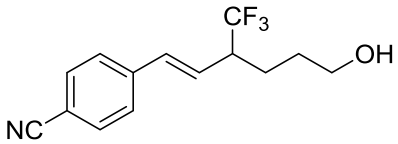

(*E*)-4-(6-hydroxy-3-(trifluoromethyl)hex-1-en-1-yl)benzonitrile (4f). The product (92 mg, 85% yield) as a yellow oil was purified with silica gel chromatography (petroleum ether/ethyl acetate 5:1). ^{1}H NMR (400 MHz, $CDCl_3$) δ7.62 (d, J = 8.4 Hz, 2H), 7.47 (d, J = 8.3 Hz, 2H) 6.60 (d, J = 16.0 Hz, 1H), 6.11 (dd, J = 16.0 Hz, 9.6 Hz, 1H), 3.69 (t, J = 6.2 Hz, 2H), 3.07 – 2.82 (m, 1H), 2.01 – 1.91 (m, 1H), 1.74 – 1.51 (m, 4H). ^{19}F NMR (376 MHz, $CDCl_3$) δ -70.6 (d, J = 8.6 Hz). ^{13}C NMR (101 MHz, $CDCl_3$) δ 140.5, 134.3, 132.4, 126.9, 126.8 (q, J = 2.5 Hz), 126.6 (q, J = 280.9 Hz), 118.7, 111.3, 62.1, 47.7 (q, J = 27.0 Hz), 29.4, 24.4 (q, J = 2.0 Hz). MS (EI): m/z (%) 269 (M^+), 154 (100). HRMS: Calculated for $C_{14}H_{14}F_3NO$: 269.1027; Found: 269.1033.

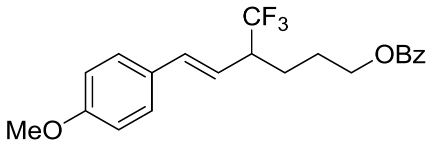

(*E*)-6-(4-Methoxyphenyl)-4-(trifluoromethyl)hex-5-en-1-yl benzoate (4g). The product (105 mg,

69% yield) as a pale yellow oil was purified with silica gel chromatography (petroleum ether/acetone 20:1). ^{1}H NMR (400 MHz, $CDCl_3$) δ 8.05 (d, *J* = 7.2 Hz, 2H), 7.57 (t, *J* = 7.4 Hz, 1H), 7.45 (t, *J* = 7.6 Hz, 2H), 7.33 (d, *J* = 8.8 Hz, 2H), 6.87 (d, *J* = 8.78 Hz, 2H), 6.54 (d, *J* = 16.0 Hz, 1H), 5.85 (dd, *J* = 15.8 Hz, 9.4 Hz, 1H), 4.35 (t, *J* = 6.2 Hz, 2H), 3.82 (s, 3H), 2.96 – 2.82 (m, 1H), 2.06 – 1.87 (m, 2H), 1.82 – 1.62 (m, 2H). ^{19}F NMR (376 MHz, $CDCl_3$) δ -70.9 (d, *J* = 8.6 Hz). ^{13}C NMR (126 MHz, $CDCl_3$) δ 166.5, 159.6, 135.7, 132.9, 130.2, 129.5, 128.8, 128.4, 127.7, 126.8 (q, *J* = 280.4 Hz), 119.9 (q, *J* = 2.5 Hz), 114.0, 64.2, 55.3, 47.6 (q, *J* = 26.8 Hz), 25.9, 24.7 (q, *J* = 2.0 Hz). MS (EI): m/z (%) 378 (M^+), 105 (100). HRMS: Calculated for $C_{21}H_{21}F_3O_3$: 378.1443; Found: 378.1436.

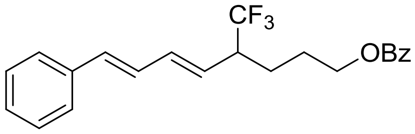

(5*E*,7*E*)-8-Phenyl-4-(trifluoromethyl)octa-5,7-dien-1-yl benzoate (4h). The product (120 mg, 80% yield) as a pale yellow oil was purified with silica gel chromatography (petroleum ether/acetone 20:1). ^{1}H NMR (400 MHz, $CDCl_3$) δ 8.05 (d, *J* = 7.2 Hz, 2H), 7.56 (t, *J* = 7.4 Hz, 1H), 7.44 (t, *J* = 7.6 Hz, 2H), 7.39 (d, *J* = 7.6 Hz, 2H), 7.32 (t, *J* = 7.6 Hz, 2H), 7.24 (t, *J* = 7.2 Hz, 1H), 6.76 (dd, *J* = 15.6 Hz, 10.4 Hz, 1H), 6.55 (d, *J* = 15.6 Hz, 1H), 6.39 (dd, *J* = 15.1 Hz, 10. Hz, 1H), 5.58 (dd, *J* = 15.2 Hz, 9.4 Hz, 1H), 4.34 (t, *J* = 6.2 Hz, 2H), 2.92 – 2.77 (m, 1H), 2.02 – 1.85 (m, 2H), 1.80 – 1.63 (m, 2H). ^{19}F NMR (376 MHz, $CDCl_3$) δ -70.8 (d, *J* = 8.6 Hz). ^{13}C NMR (126 MHz, $CDCl_3$) δ 166.5, 136.8, 136.5, 133.7, 133.0, 130.2, 129.5, 128.6, 128.4, 127.9, 127.5, 126.7 (q, *J* = 280.6 Hz), 126.5, 125.9 (q, *J* = 2.4 Hz), 64.2, 47.4 (q, *J* = 26.9 Hz), 25.9, 24.7 (q, *J* = 2.0 Hz). MS (EI): m/z (%) 374 (M^+), 105 (100). HRMS: Calculated for $C_{22}H_{21}F_3O_2$: 374.1494; Found: 374.1505.

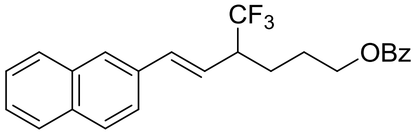

(*E*)-6-(Naphthalen-2-yl)-4-(trifluoromethyl)hex-5-en-1-yl benzoate (4i). The product (145 mg, 91% yield) as a pale yellow solid was purified with silica gel chromatography (petroleum ether/ethyl acetate 30:1). m.p. 79~82 ^{o}C. ^{1}H NMR (400 MHz, $CDCl_3$) δ 8.07 (d, *J* = 7.2 Hz, 2H), 7.85 – 7.80 (m, 3H), 7.75 (s, 1H), 7.65 – 7.55 (m, 2H), 7.52 – 7.42 (m, 4H), 6.78 (d, *J* = 15.6 Hz, 1H), 6.14 (dd, *J* =

15.8 Hz, 9.2 Hz, 1H), 4.39 (t, *J* = 6.0 Hz, 2H), 3.08 – 2.93 (m, 1H), 2.10 – 2.02 (m, 1H), 2.01 – 1.91 (m, 1H), 1.89 – 1.75 (m, 2H). ^{19}F NMR (376 MHz, $CDCl_3$) δ -70.7 (d, *J* = 8.6 Hz). ^{13}C NMR (101 MHz, $CDCl_3$) δ 166.5, 136.4, 133.42, 133.39, 133.2, 132.9, 130.1, 129.5, 128.4, 128.3, 128.0, 127.6, 126.9 (q, *J* = 281.3 Hz), 126.7, 126.4, 126.1, 123.3, 122.5 (q, *J* = 2.4 Hz), 64.2, 47.7 (q, *J* = 27.0 Hz), 25.9, 24.7 (q, *J* = 2.0 Hz). MS (EI): m/z (%) 398 (M^+), 105 (100). HRMS: Calculated for $C_{24}H_{21}F_3O_2$: 398.1494; Found: 398.1491.

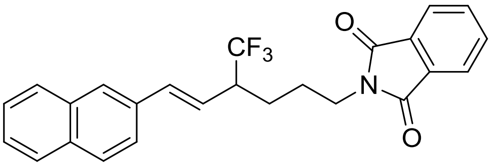

(*E*)-2-(6-(Naphthalen-2-yl)-4-(trifluoromethyl)hex-5-en-1-yl)isoindoline-1,3-dione (4j). The reaction was carried out in the presence of 2-vinylnaphthalene (0.2 mmol), **1e** (0.4 mmol, 2.0 equiv), $PdCl_2(PPh_3)_2$ (5 mol %), Xantphos (7.5 mol %), and KOAc (2.0 equiv) in DCE (3 mL). The product (72 mg, 85% yield) as a white solid was purified with silica gel chromatography (petroleum ether/ethyl acetate 10:1). m.p. 156~159 ^{o}C. ^{1}H NMR (400 MHz, $CDCl_3$) δ 7.88 – 7.75 (m, 5H), 7.75 – 7.64 (m, 3H), 7.58 (d, *J* = 8.4 Hz, 1H), 7.51 – 7.41 (m, 2H), 6.74 (d, *J* = 16.0 Hz, 1H), 6.07 (dd, *J* = 15.8 Hz, 9.4 Hz, 1H), 3.73 (t, *J* = 6.6 Hz, 2H), 3.07 – 2.91 (m, 1H), 1.94 – 1.80 (m, 2H), 1.78 – 1.63 (m, 2H). ^{19}F NMR (376 MHz, $CDCl_3$) δ -70.7 (d, *J* = 8.6 Hz). ^{13}C NMR (126 MHz, $CDCl_3$) δ 168.3, 136.4, 133.9, 133.42, 133.41, 133.2, 132.0, 128.3, 128.0, 127.6, 126.7 (q, *J* = 280.6 Hz), 126.69, 126.3, 126.1, 123.4, 123.2, 122.5 (q, *J* = 2.3 Hz), 47.6 (q, *J* = 26.9 Hz), 37.3, 25.7, 25.2 (q, *J* = 1.6 Hz). MS (EI): m/z (%) 423 (M^+), 165 (100). HRMS: Calculated for $C_{25}H_{20}F_3NO_2$: 423.1446; Found: 423.1447.

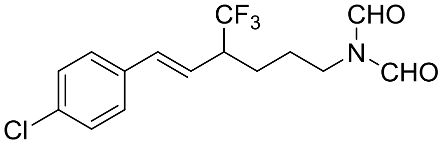

(*E*)-*N*-(6-(4-chlorophenyl)-4-(trifluoromethyl)hex-5-en-1-yl)-*N*-formylformamide (4k). The product (122 mg, 91% yield) as a white solid was purified with silica gel chromatography (petroleum ether/ethyl acetate 5:1). m.p. 57~60 °C. ^{1}H NMR (400 MHz, $CDCl_3$) δ 8.83 (s, 2H), 7.36 – 7.27 (m, 4H), 6.54 (d, *J* = 16.0 Hz, 1H), 5.90 (dd, *J* = 15.8 Hz, 9.4 Hz, 1H), 3.67 (t, *J* = 6.6 Hz, 2H), 2.94 –

2.80 (m, 1H), 1.85 – 1.52 (m, 4H). ^{19}F NMR (376 MHz, $CDCl_3$) δ -70.8 (d, *J* = 8.6 Hz). ^{13}C NMR (126 MHz, $CDCl_3$) δ 163.9, 135.2, 134.4, 133.9, 128.8, 127.7, 126.6 (q, *J* = 280.7 Hz), 122.6, 47.4 (q, *J* = 27.1 Hz), 38.1, 25.0, 24.6. MS (EI): m/z (%) 333 (M^+), 313 (100). HRMS: Calculated for $C_{15}H_{15}ClF_3NO_2$: 333.0743; Found: 333.0746.

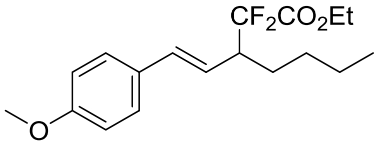

Ethyl (*E*)-2,2-difluoro-3-(4-methoxystyryl)heptanoate (4l). The product (107 mg, 82% yield) as a colorless oil was purified with silica gel chromatography (petroleum ether/ethyl acetate 30:1). ^{1}H NMR (400 MHz, $CDCl_3$) δ 7.30 (d, *J* = 8.8 Hz, 2H), 6.85 (d, *J* = 8.8 Hz, 2H), 6.43 (d, *J* = 16.0 Hz, 1H), 5.77 (dd, *J* = 15.8 Hz, 9.6 Hz, 1H), 4.27 (q, *J* = 7.1 Hz, 2H), 3.81 (s, 3H), 3.05 – 2.73 (m, 1H), 1.81 – 1.69 (m, 1H), 1.41 – 1.20 (m, 8H), 0.89 (t, *J* = 6.8 Hz, 3H). ^{19}F NMR (376 MHz, $CDCl_3$) δ -109.3 (dd, *J* = 251.9 Hz, 11.1 Hz, 1F), -114.46 (dd, *J* = 251.2 Hz, 19.2 Hz, 1F). ^{13}C NMR (101 MHz, $CDCl_3$) δ 164.2 (t, *J* = 33.0 Hz), 159.4, 134.9, 129.2, 127.6, 121.6 (dd, *J* = 6.2 Hz, 1.4 Hz), 116.7 (t, *J* = 255.7 Hz), 113.9, 62.5, 55.2, 48.2 (t, *J* = 22.1 Hz), 28.9, 26.5, 22.4, 14.0, 13.8. MS (EI): m/z (%) 326 (M^+), 203 (100). HRMS: Calculated for $C_{18}H_{24}F_2O_3$: 326.1694; Found: 326.1696.

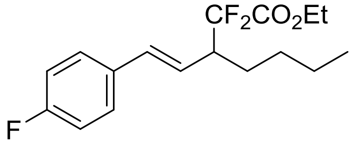

Ethyl (*E*)-2,2-difluoro-3-(4-fluorostyryl)heptanoate (4m). The product (96 mg, 76% yield) as a colorless oil was purified with silica gel chromatography (petroleum ether/acetone 150:1). ^{1}H NMR (400 MHz, $CDCl_3$) δ 7.37 – 7.29 (m, 2H), 7.00 (t, *J* = 8.2 Hz, 2H), 6.46 (d, *J* = 16.0 Hz, 1H), 5.84 (dd, *J* = 15.5 Hz, 10.0 Hz, 1H), 4.28 (q, *J* = 7.0 Hz, 2H), 2.96 – 2.79 (m, 1H), 1.81 – 1.67 (m, 1H), 1.61 – 1.47 (m, 1H), 1.42 – 1.20 (m, 7H), 0.89 (t, *J* = 6.4 Hz, 3H). ^{19}F NMR (376 MHz, $CDCl_3$) δ -109.7 (dd, *J* = 252.5 Hz, 11.5 Hz, 1F), -113.13 – -114.26 (m, 2F). ^{13}C NMR (101 MHz, $CDCl_3$) δ 164.2 (t, *J* = 33.0 Hz), 162.5 (d, *J* = 248.2 Hz), 134.3, 132.6 (d, *J* = 3.1 Hz), 128.0 (d, *J* = 8.0 Hz), 123.7 (d, *J* = 6.2 Hz), 116.6 (t, *J* = 255.8 Hz) 115.5 (d, *J* = 21.7 Hz), 62.6, 48.2 (t, *J* = 22.3 Hz), 28.9, 26.5 (t, *J* = 2.4 Hz), 22.4, 14.0, 13.8. MS (EI): m/z (%) 314 (M^+), 135 (100). HRMS: Calculated for

$C_{17}H_{21}F_3O_2$: 314.1494; Found: 314.1504.

REFERENCES.

1. Li, X. -F.; Feng, Z.; Jiang, Z.; Zhang. X. *Org. Lett.* **2015**, *17*, 5570.

2. Liang, Y.; Fu, G. C. *J. Am. Chem. Soc.* **2015**, *137*, 9523.

3. Han, Y.-L, Hu, H, *Synthesis* **1990**, *2*, 122.

2-Bromo-1,1,1-trifluorohexane (2a).

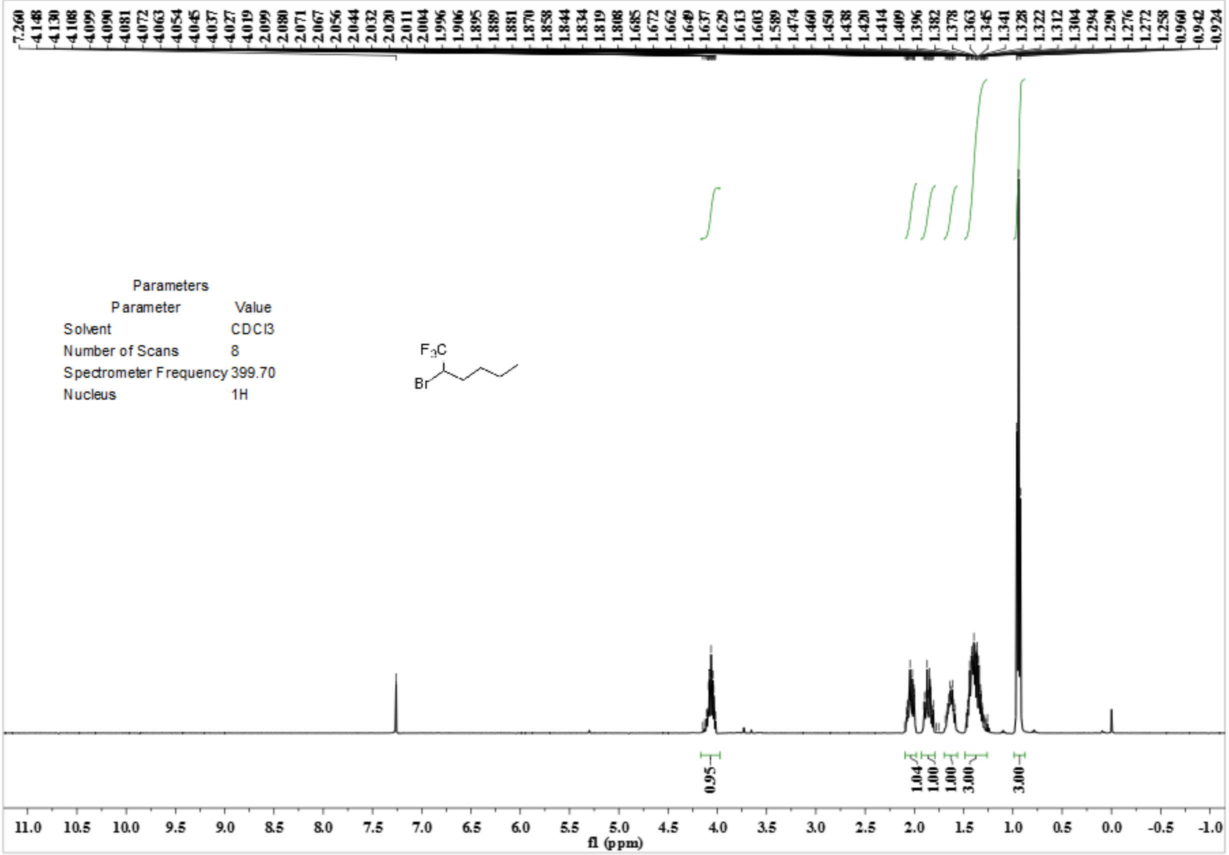

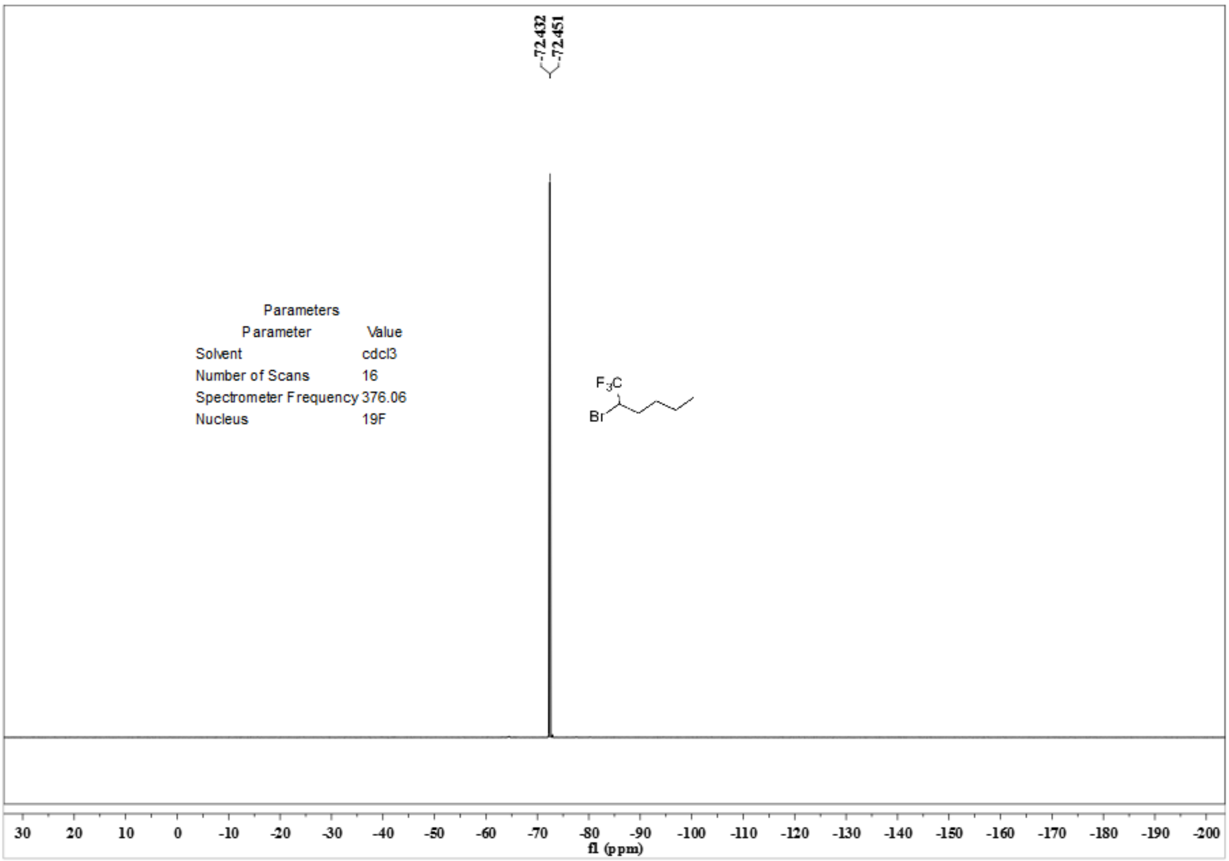

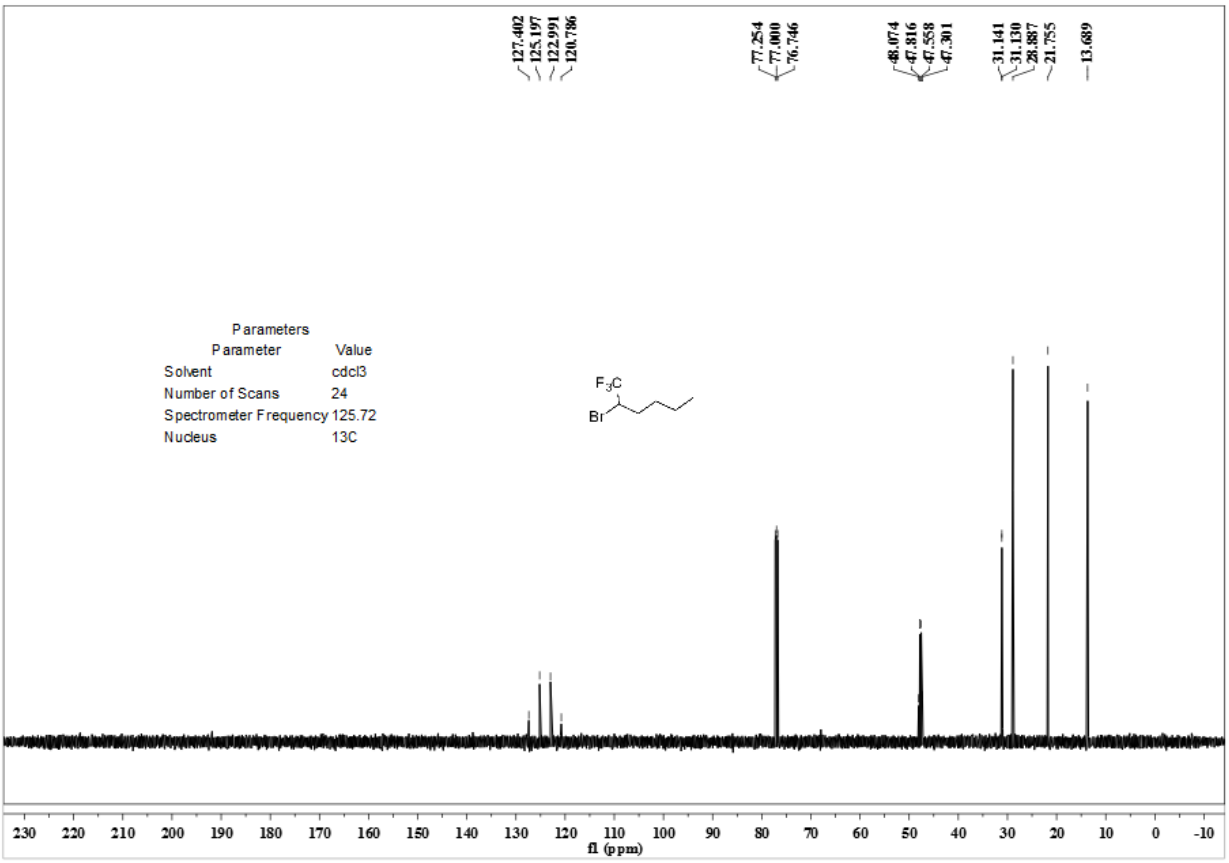

(3-Bromo-4,4,4-trifluorobutyl)benzene (2b).

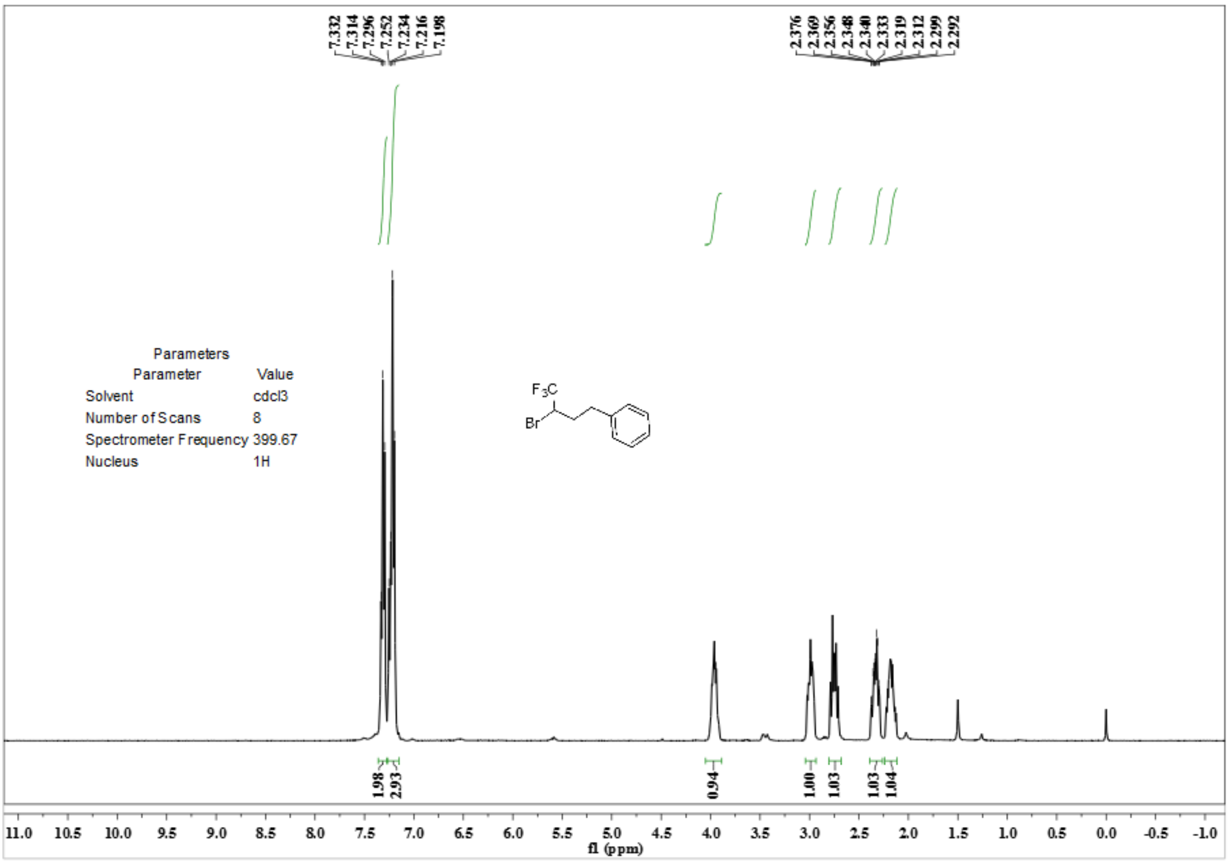

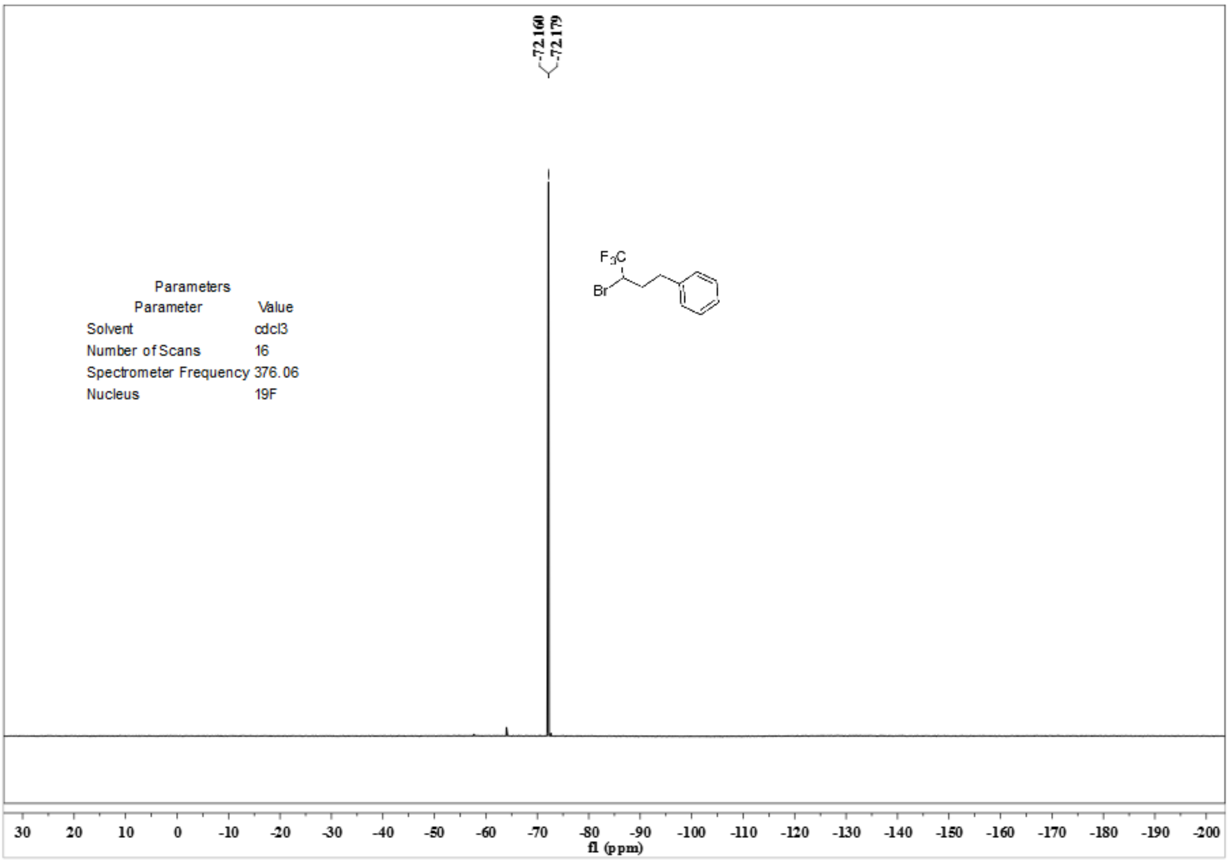
-72.160
-72.179
F3C
Br
Parameters
Parameter Value
Solvent cdcl3
Number of Scans 16
Spectrometer Frequency 376.06
Nucleus 19F
fl (ppm)

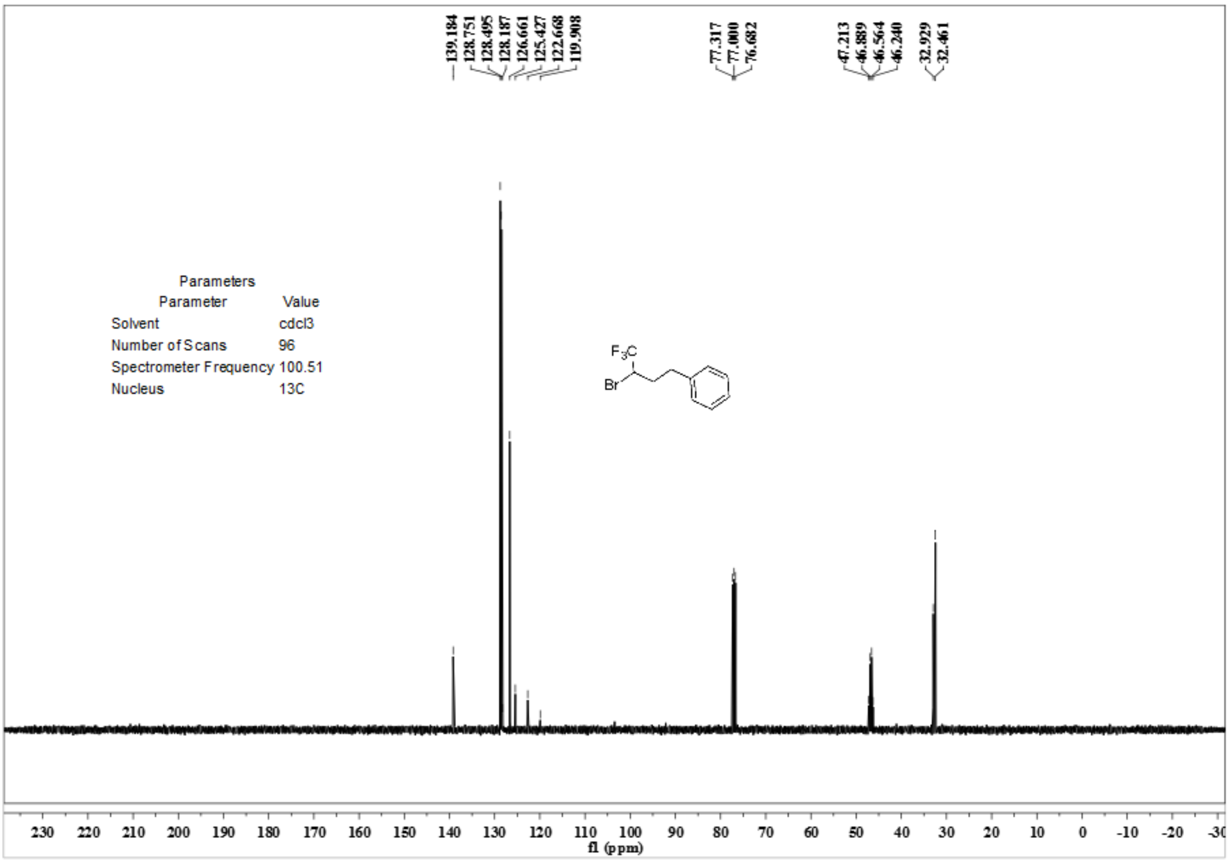
139.184
128.751
128.495
128.187
126.661
125.427
122.668
119.908
77.317
77.000
76.682
47.213
46.889
46.564
46.240
32.929
32.461
Parameters
Parameter Value
Solvent cdcl3
Number of Scans 96
Spectrometer Frequency 100.51
Nucleus 13C
F3C
Br
fl (ppm)

4-Bromo-5,5,5-trifluoropentan-1-ol (2c).

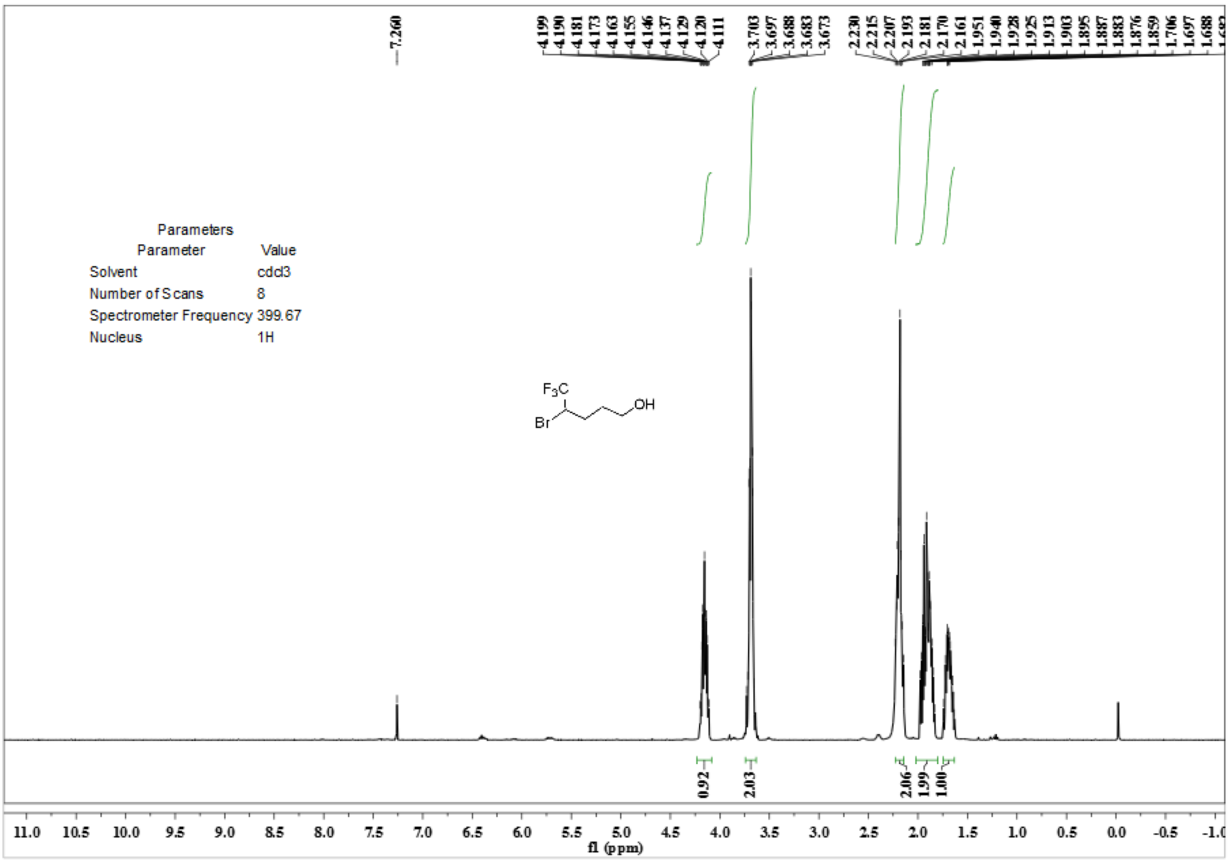

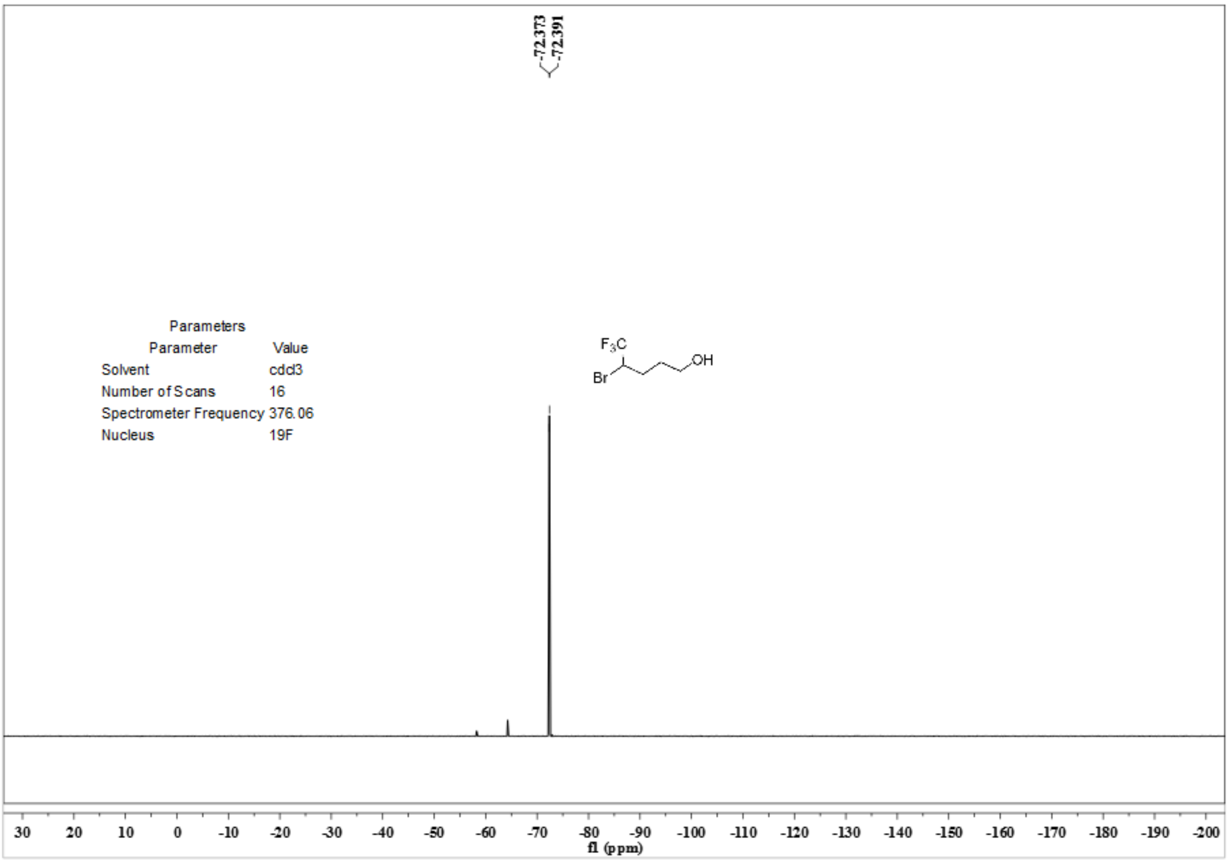

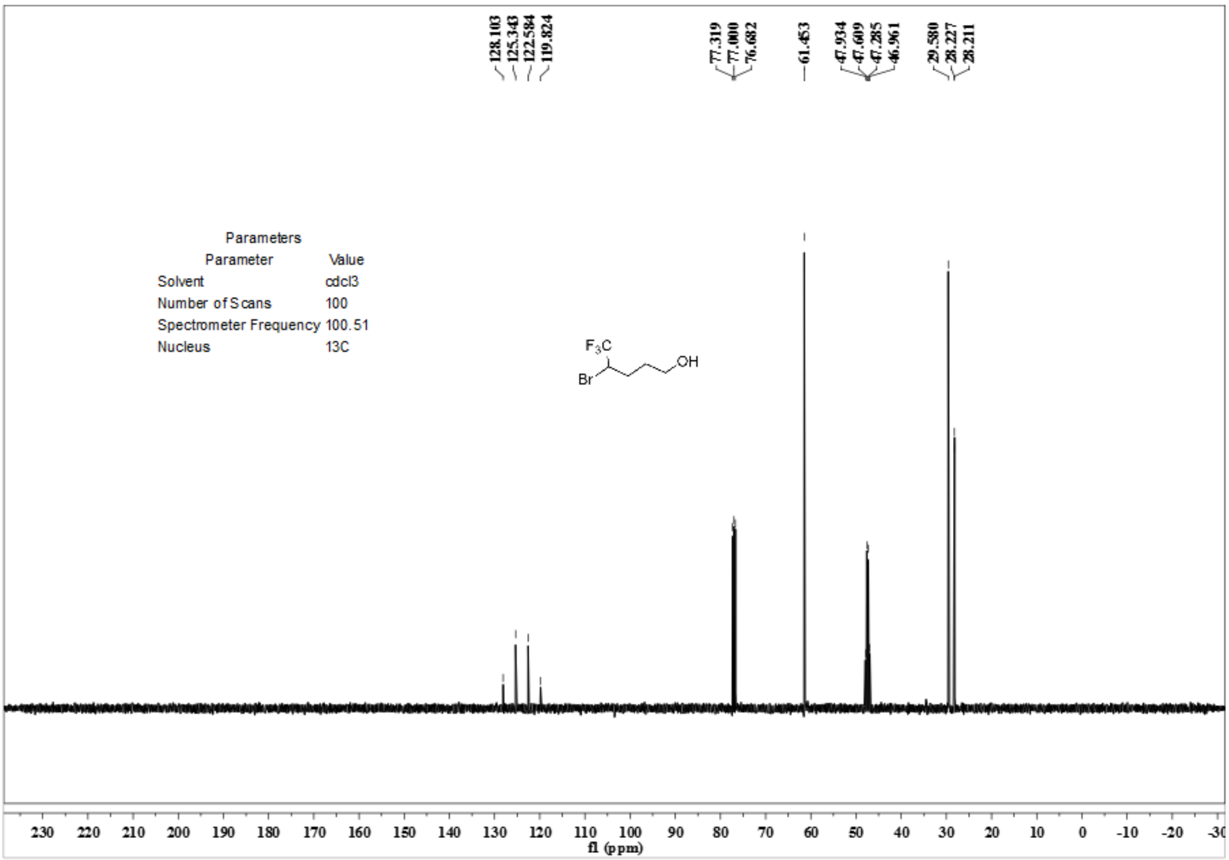

4-Bromo-5,5,5-trifluoropentyl benzoate (2d).

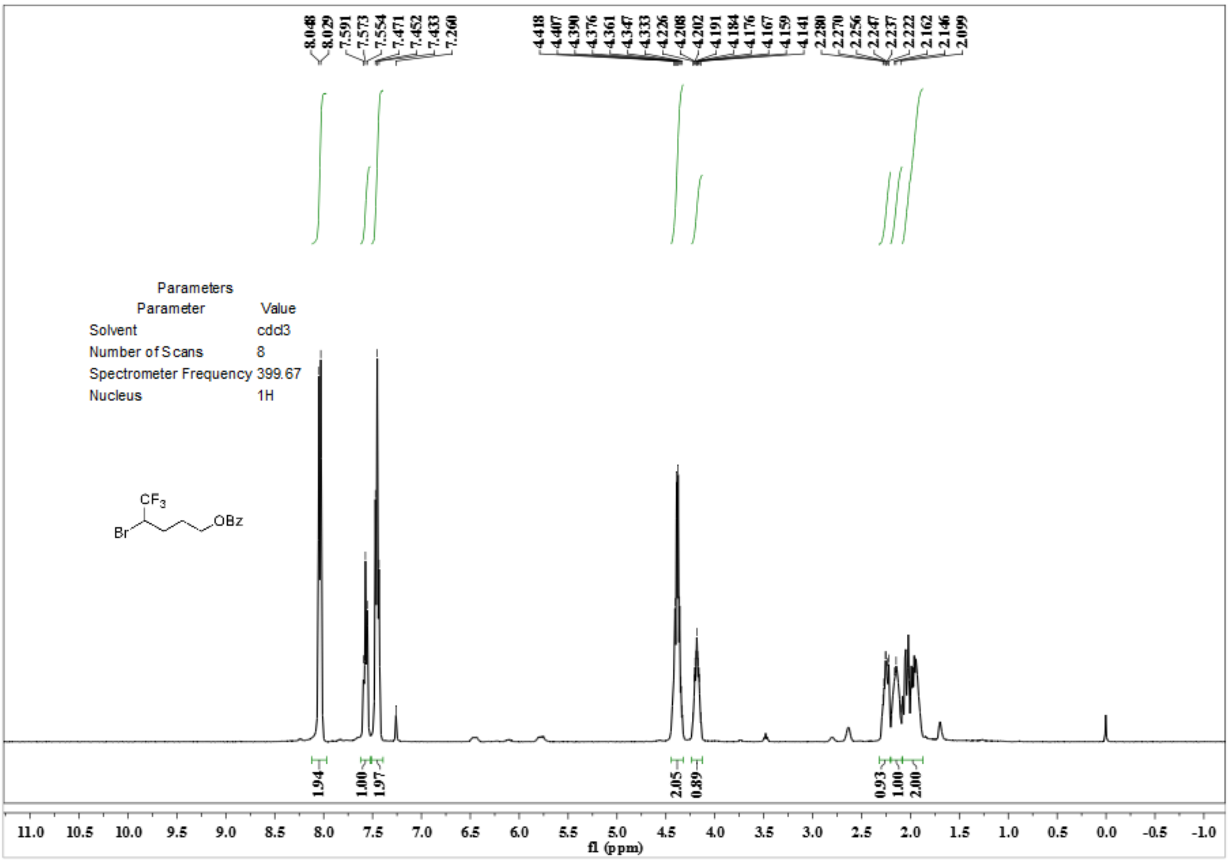

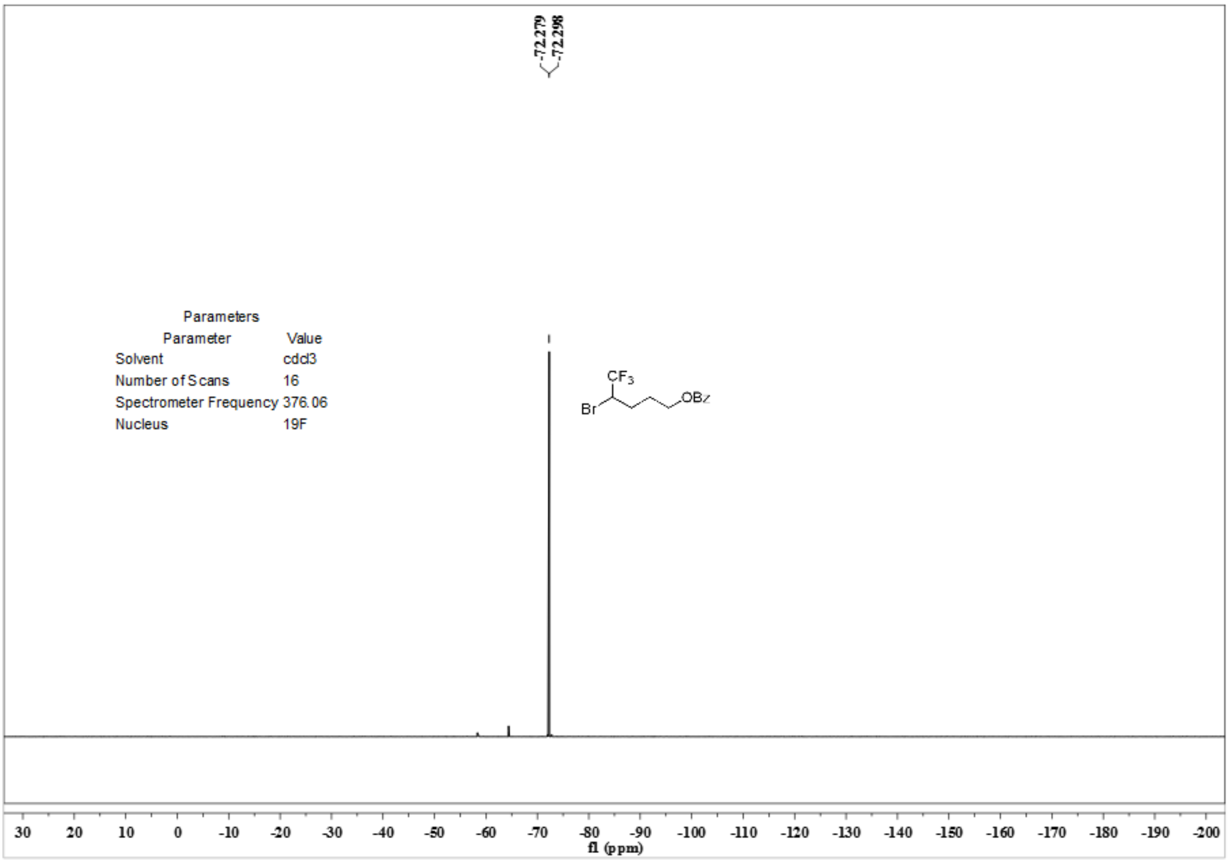
-72.279
-72.298
Parameters
Parameter Value
Solvent cdcl3
Number of Scans 16
Spectrometer Frequency 376.06
Nucleus 19F
CF3
Br
OBz
fl (ppm)

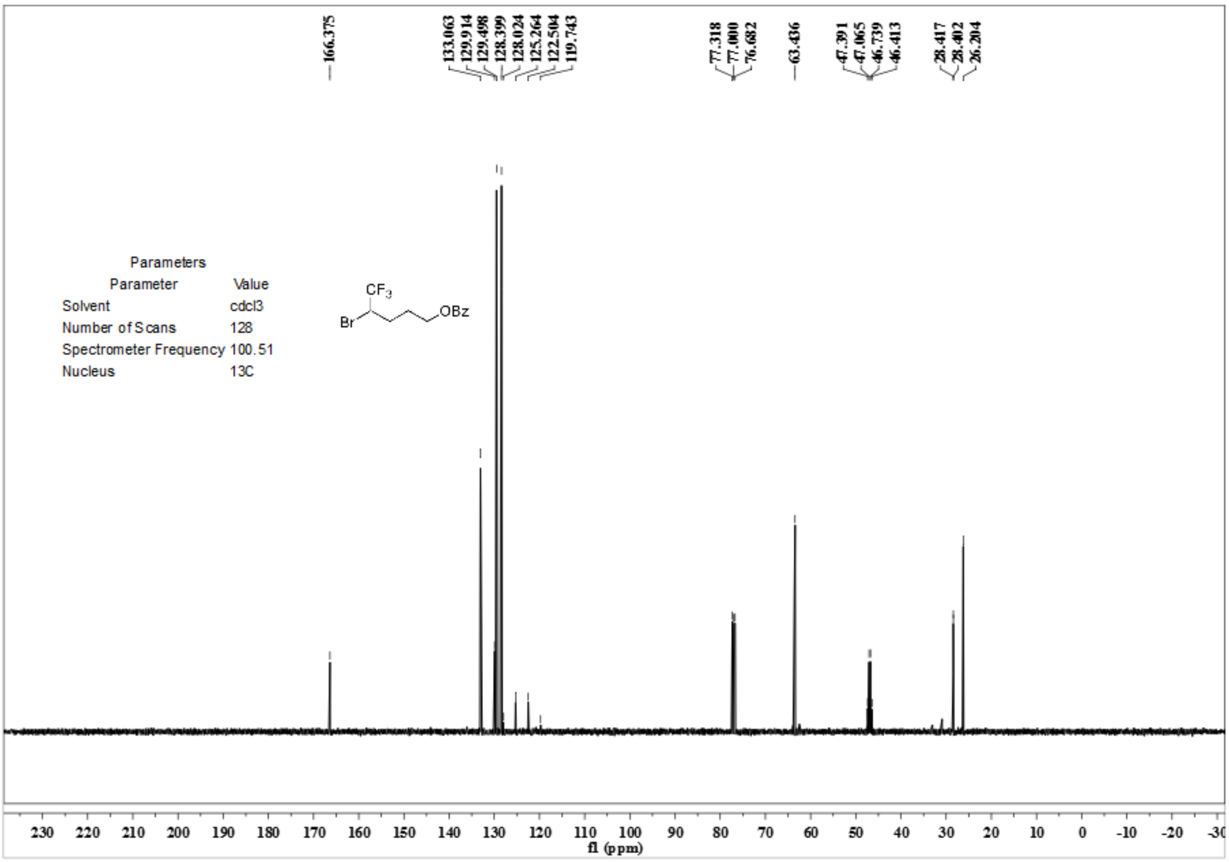
166.375
133.063
129.914
129.498
128.399
128.024
125.264
122.504
119.743
77.318
77.000
76.682
63.436
47.391
47.065
46.739
46.413
28.417
28.402
26.204
Parameters
Parameter Value
Solvent cdcl3
Number of Scans 128
Spectrometer Frequency 100.51
Nucleus 13C
CF3
Br
OBz
fl (ppm)

2-(4-Bromo-5,5,5-trifluoropentyl)-3a,7a-dihydro-1*H*-isoindole-1,3(2*H*)-dione (2e).

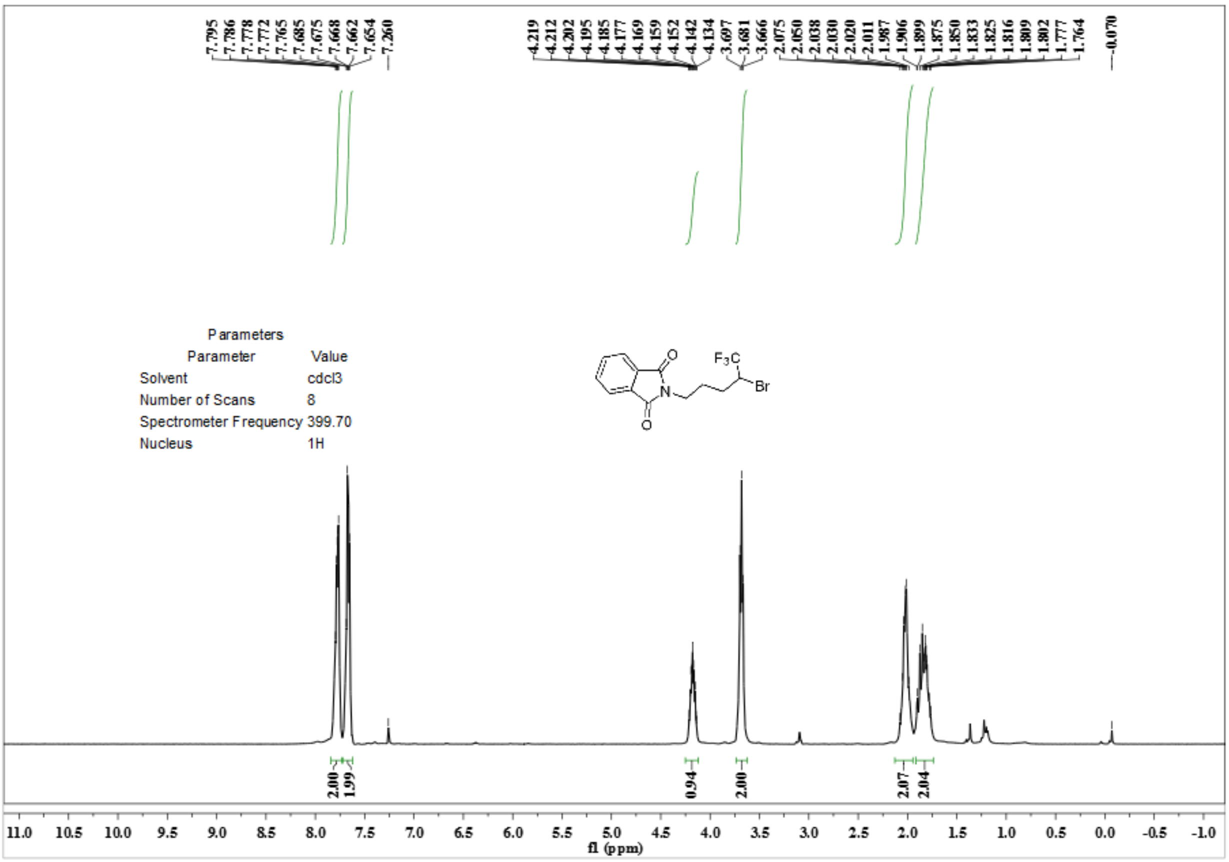

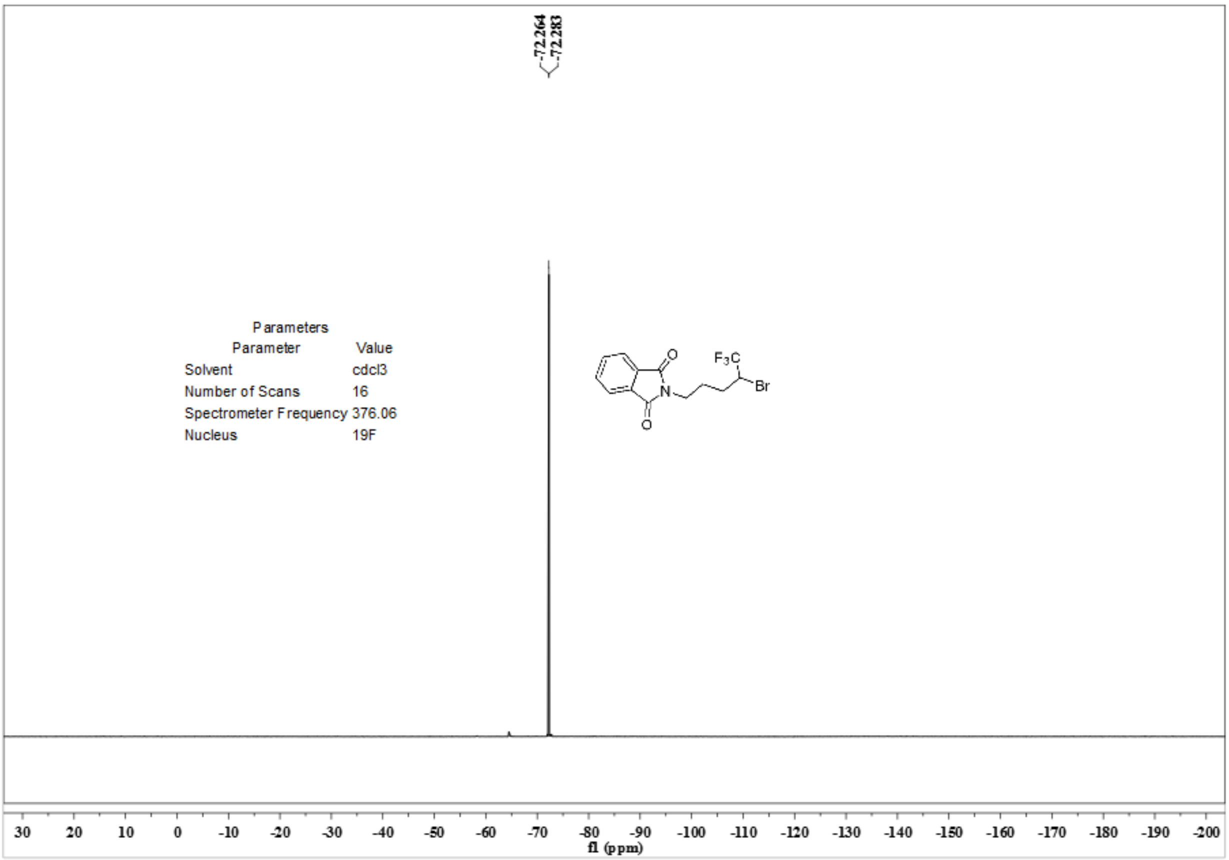

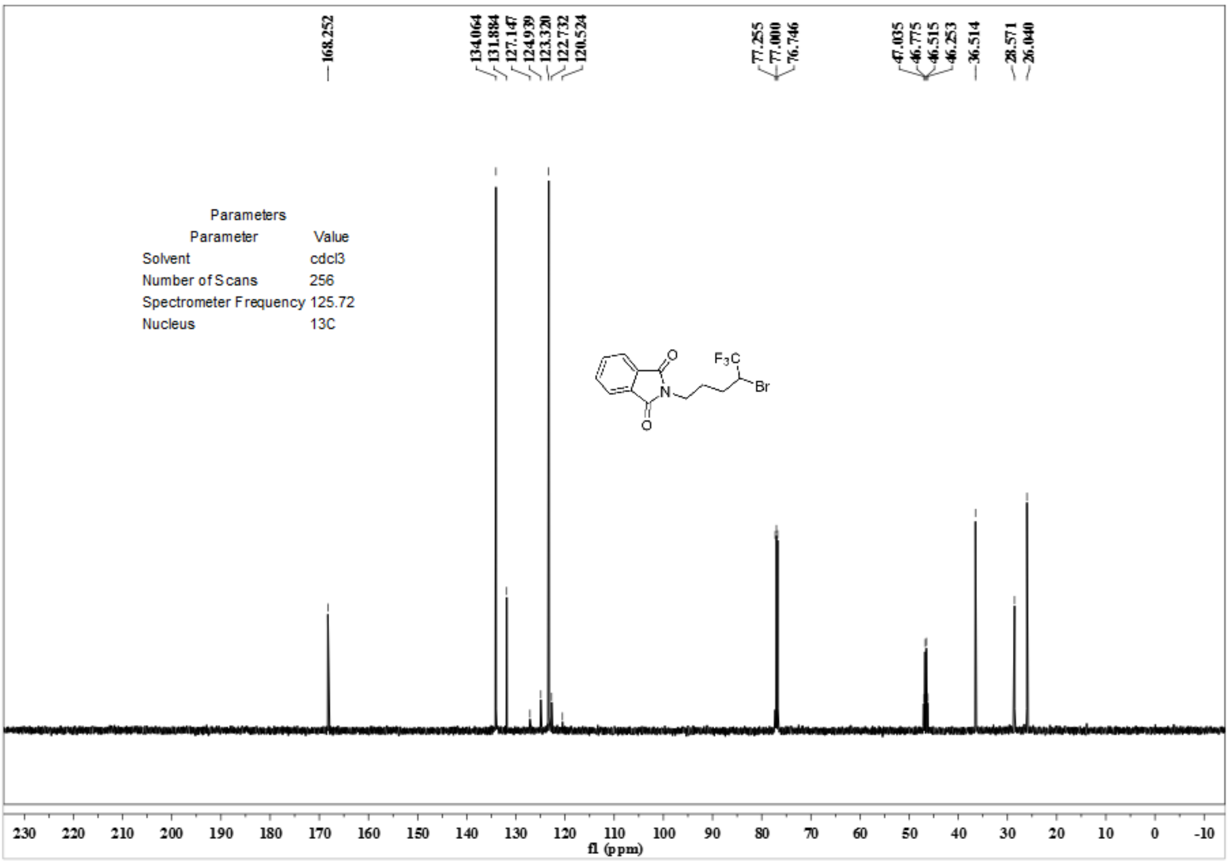

N-(4-Bromo-5, 5, 5-trifluoropentyl)-N-formylformamide (2f)

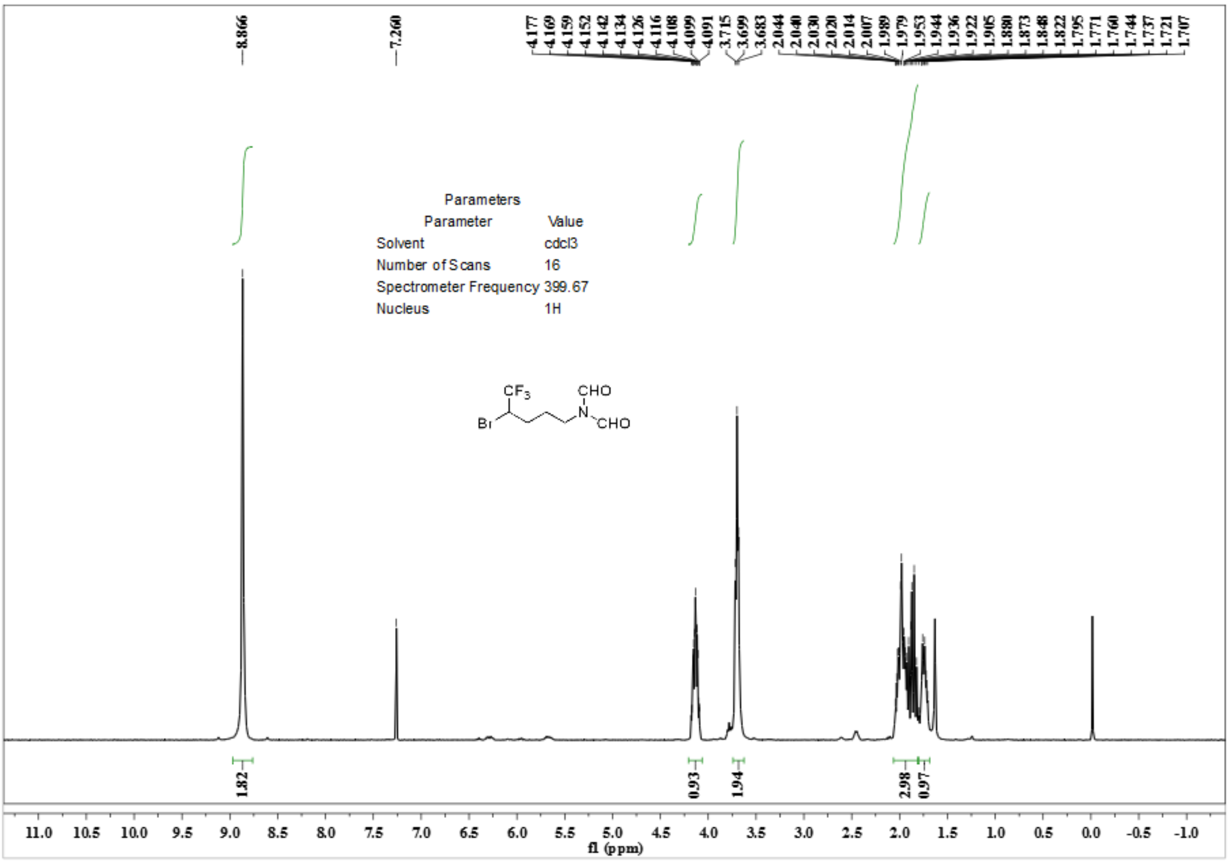

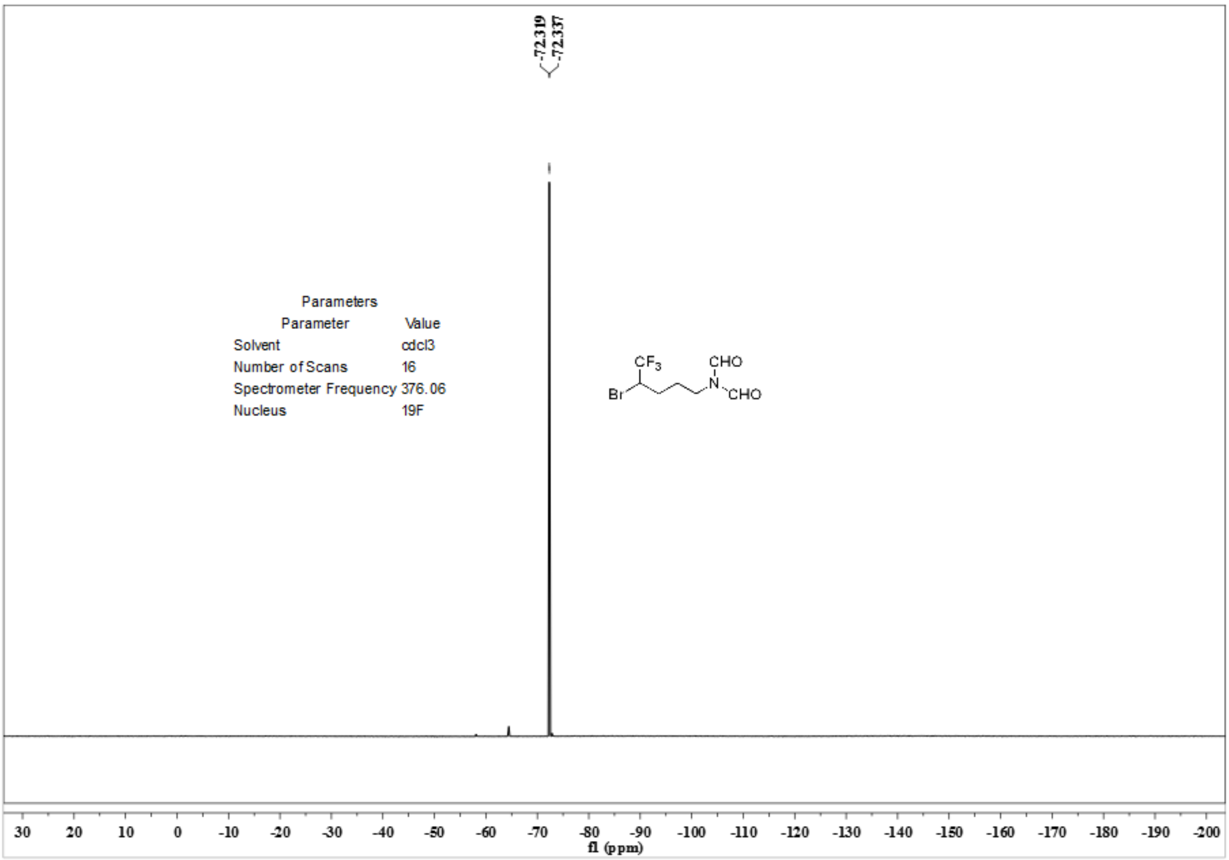

-72.319
-72.337
Parameters
Parameter Value
Solvent cdcl3
Number of Scans 16
Spectrometer Frequency 376.06
Nucleus 19F
CF3
CHO
Br
N
CHO
30 20 10 0 -10 -20 -30 -40 -50 -60 -70 -80 -90 -100 -110 -120 -130 -140 -150 -160 -170 -180 -190 -200
f1 (ppm)

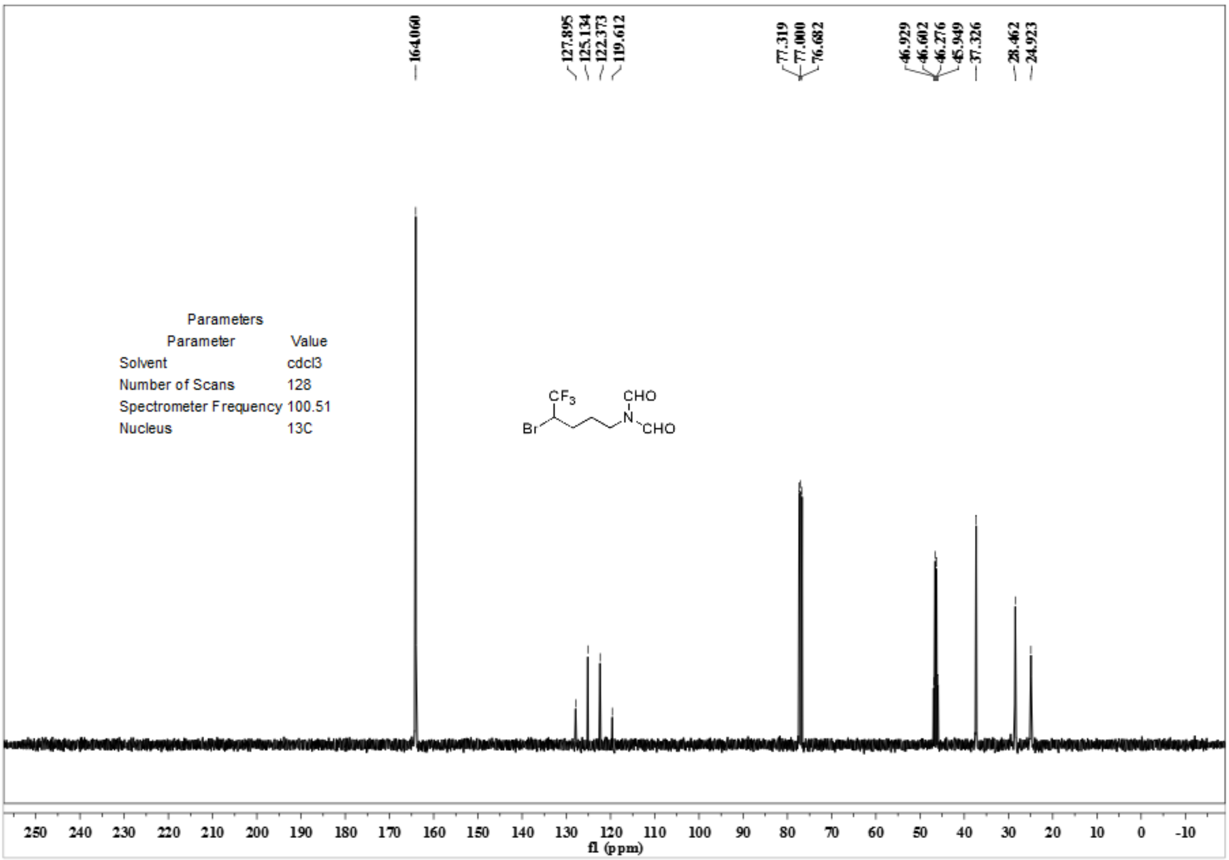

164.060
127.895
125.134
122.373
119.612
77.319
77.000
76.682
46.929
46.602
46.276
45.949
37.326
28.462
24.923
Parameters
Parameter Value
Solvent cdcl3
Number of Scans 128
Spectrometer Frequency 100.51
Nucleus 13C
CF3
CHO
Br
N
CHO
250 240 230 220 210 200 190 180 170 160 150 140 130 120 110 100 90 80 70 60 50 40 30 20 10 0 -10
f1 (ppm)

Ethyl 2, 2-difluoro-3-iodoheptanoate (2g).

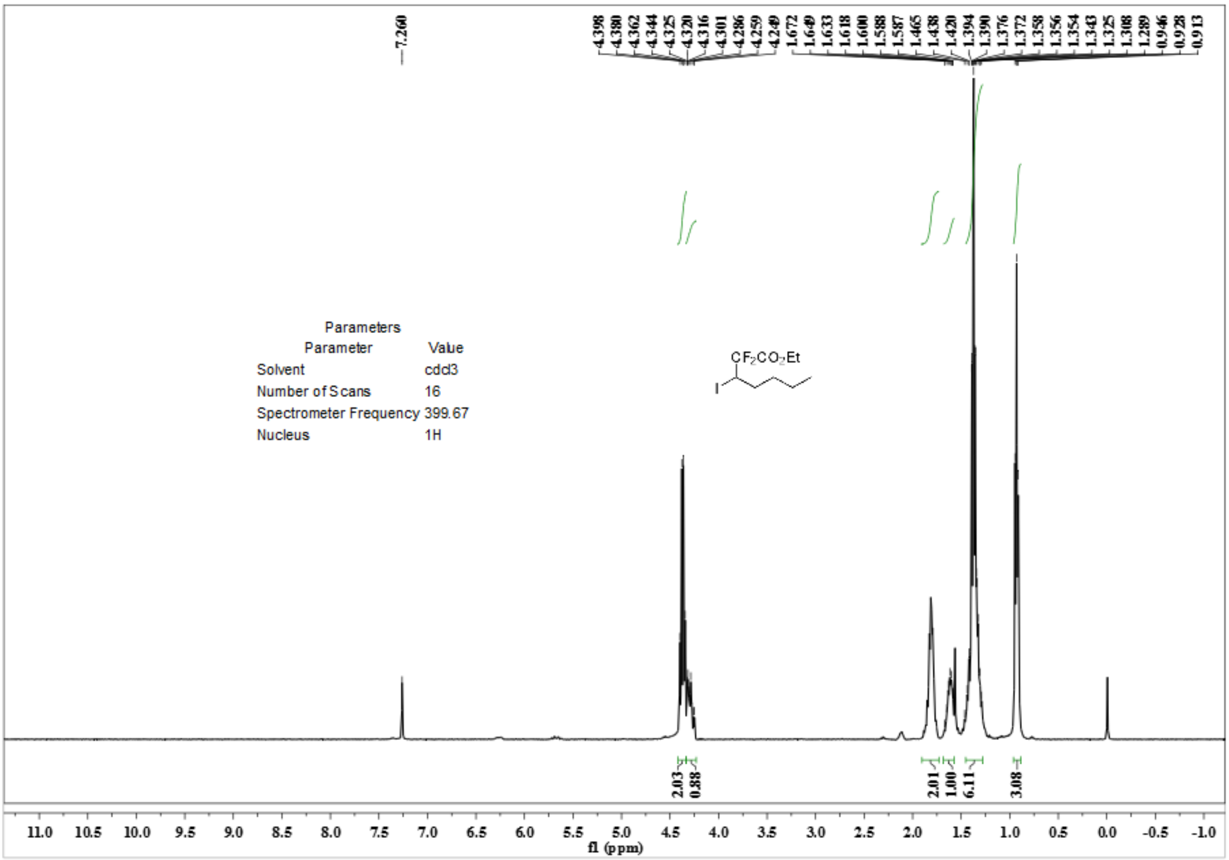

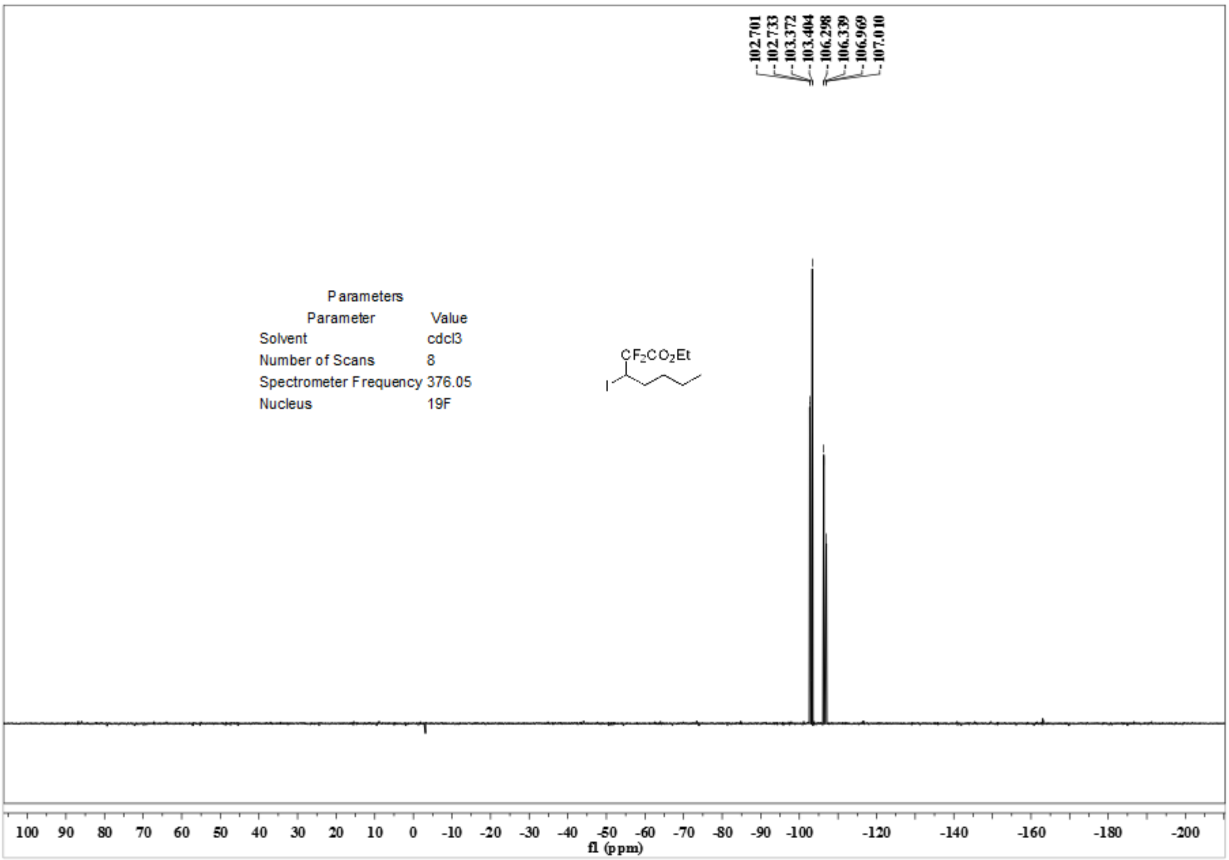

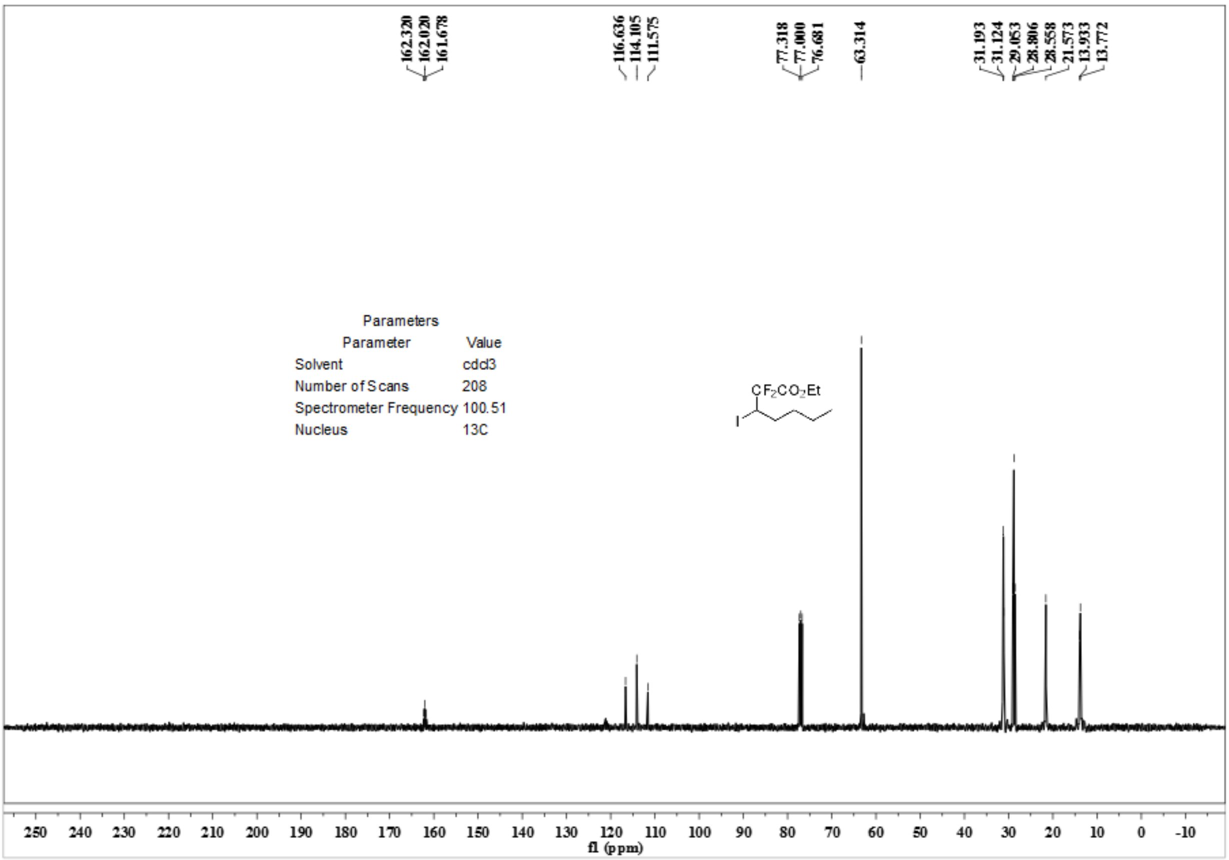

(*E*)-(3-(Trifluoromethyl)hept-1-en-1-yl)benzene (3a).

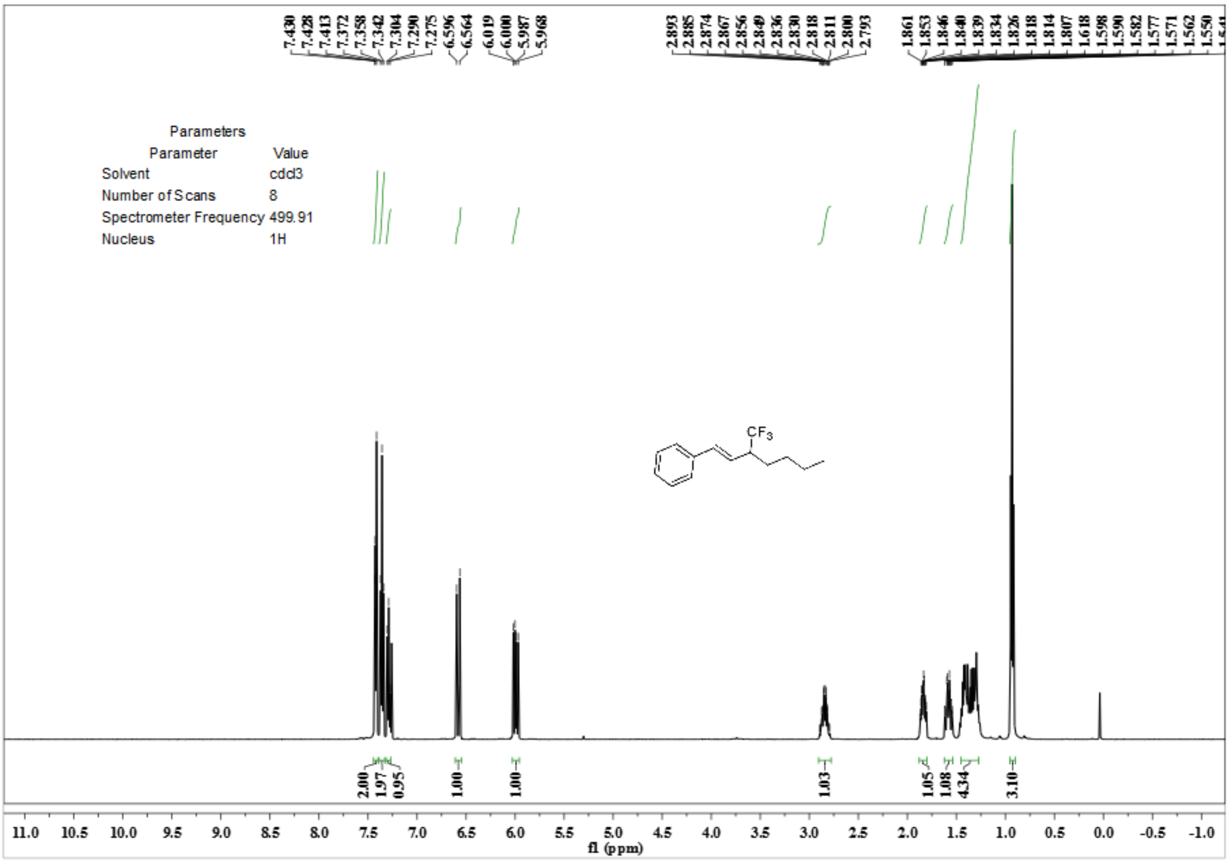

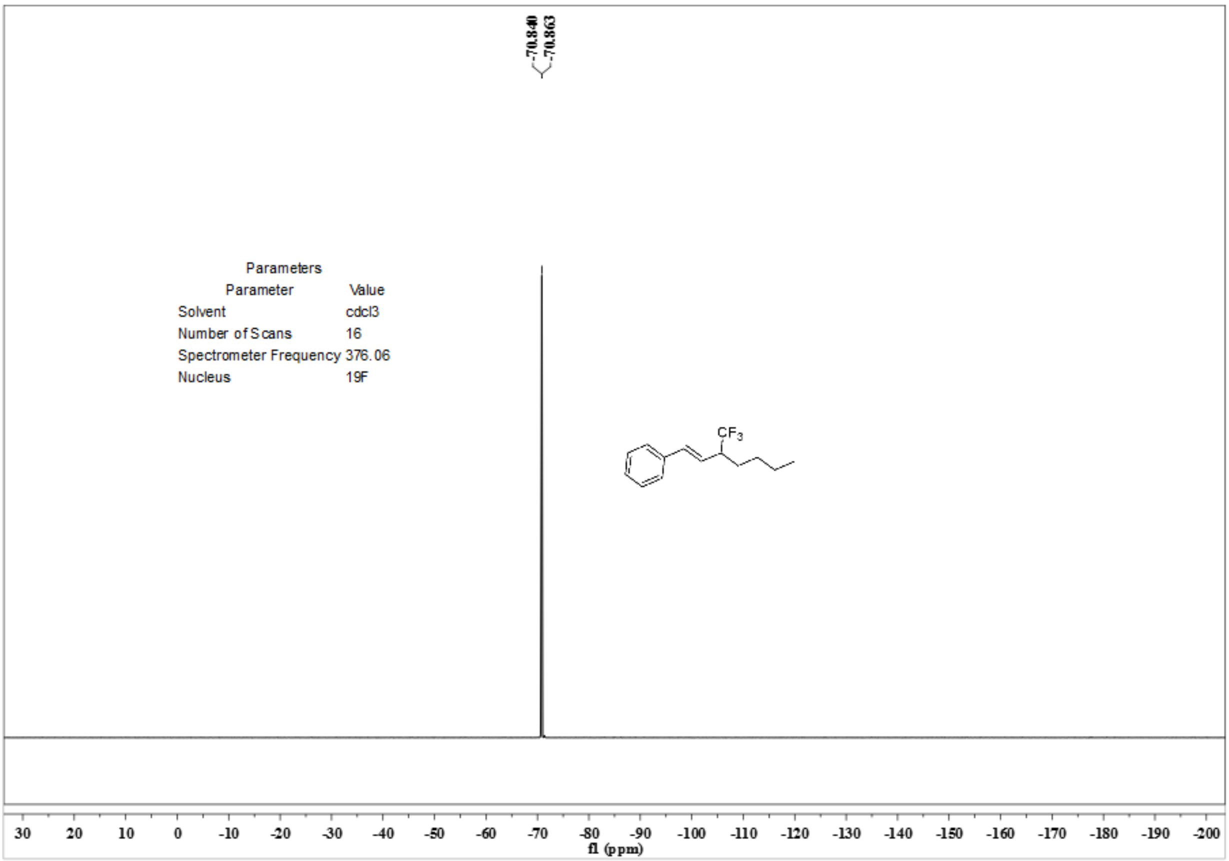
-70.840
-70.863
Parameters
Parameter Value
Solvent cdcl3
Number of Scans 16
Spectrometer Frequency 376.06
Nucleus 19F
CF3
fl (ppm)

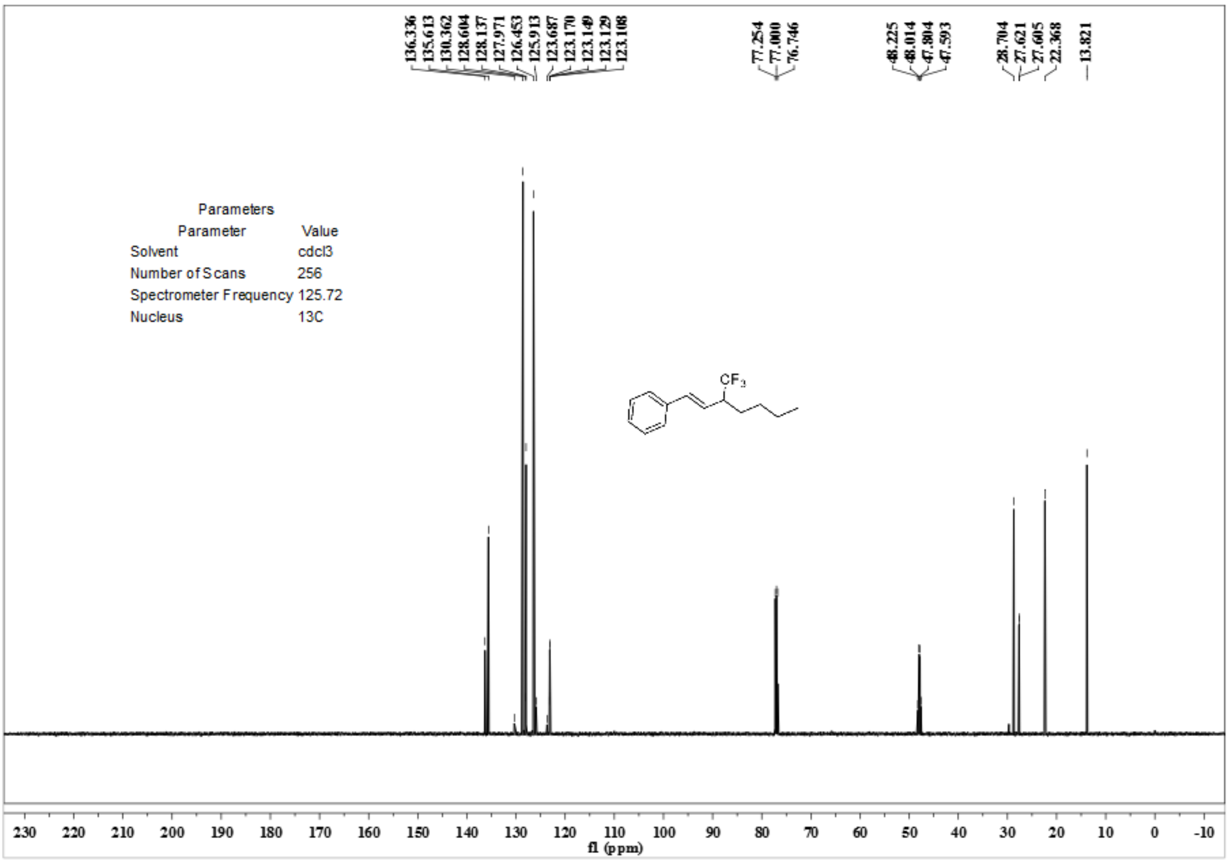
136.336
135.613
130.362
128.604
128.137
127.971
126.453
125.913
123.687
123.170
123.149
123.129
123.108
77.254
77.000
76.746
48.225
48.014
47.804
47.593
28.704
27.621
27.605
22.368
13.821
Parameters
Parameter Value
Solvent cdcl3
Number of Scans 256
Spectrometer Frequency 125.72
Nucleus 13C
CF3
fl (ppm)

(*E*)-1-Methyl-4-(3-(trifluoromethyl)hept-1-en-1-yl)benzene (3b).

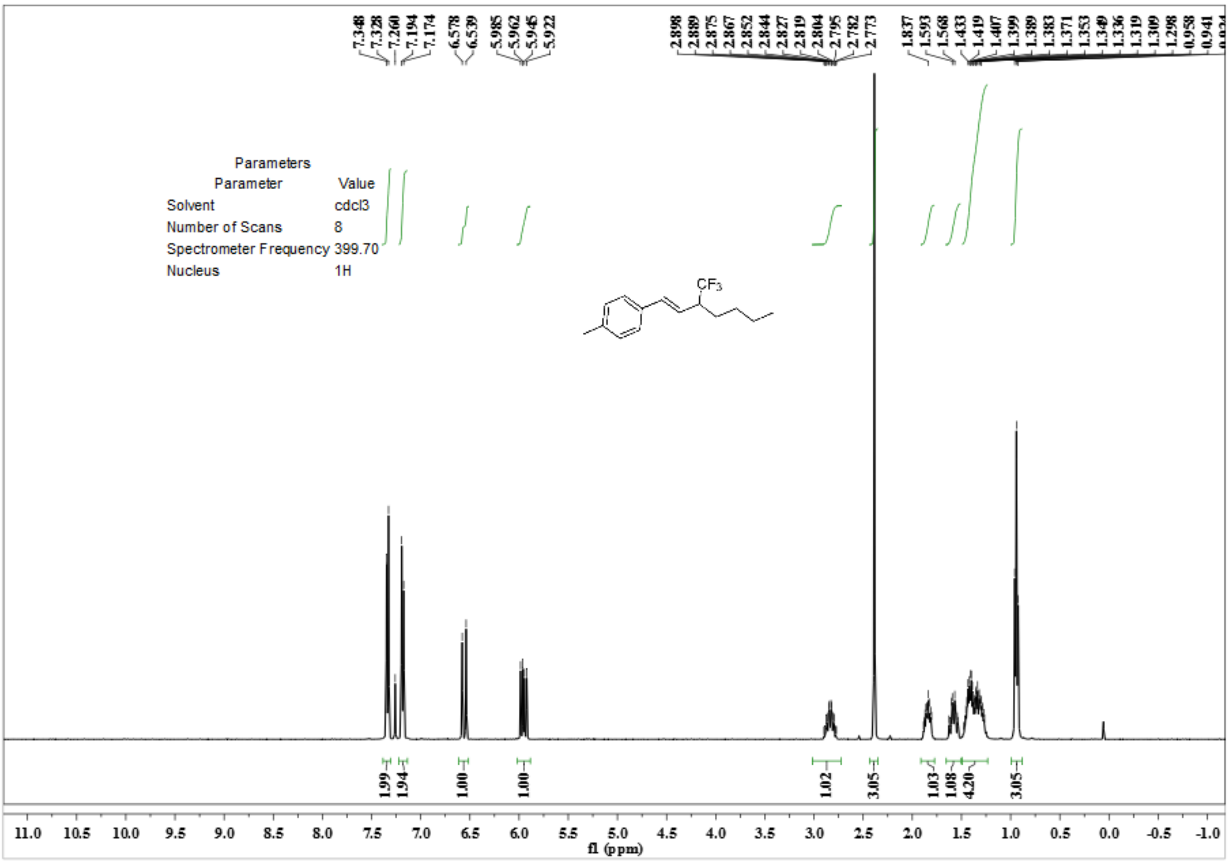

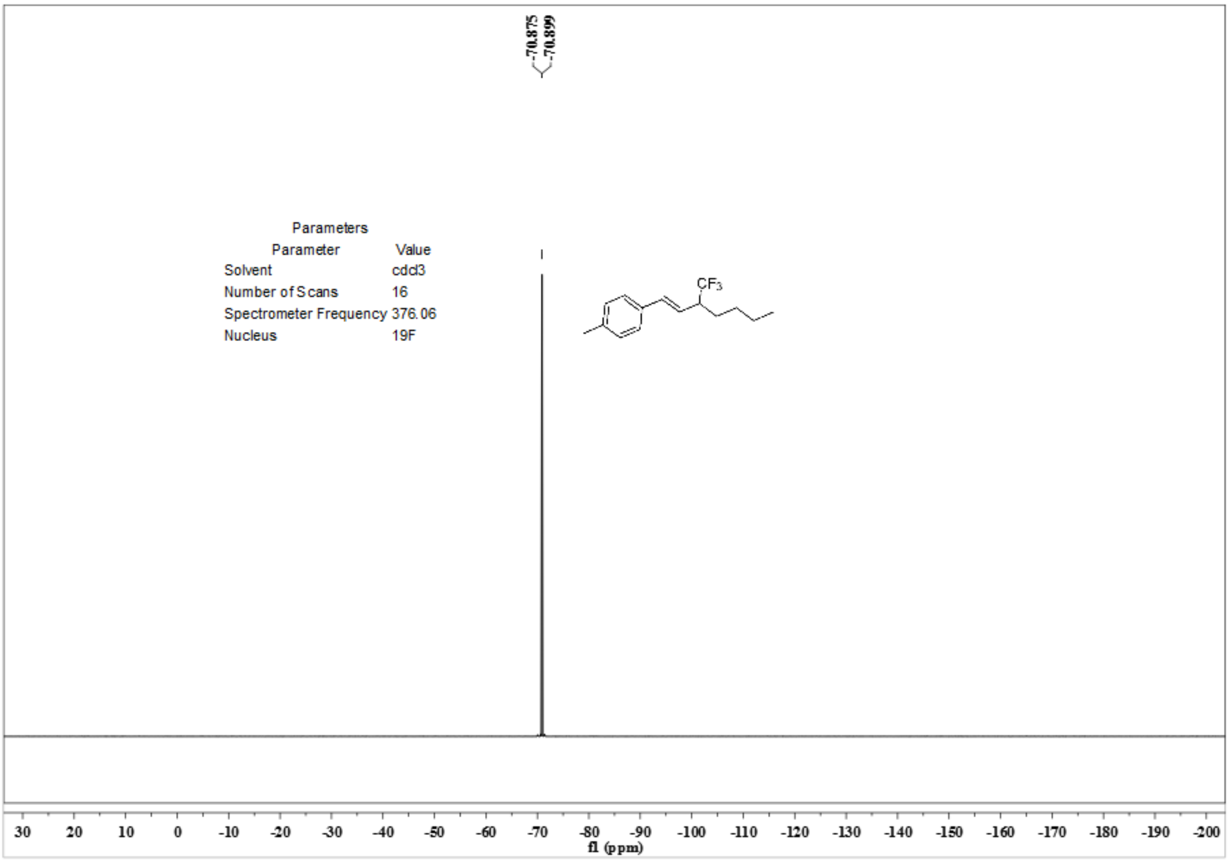

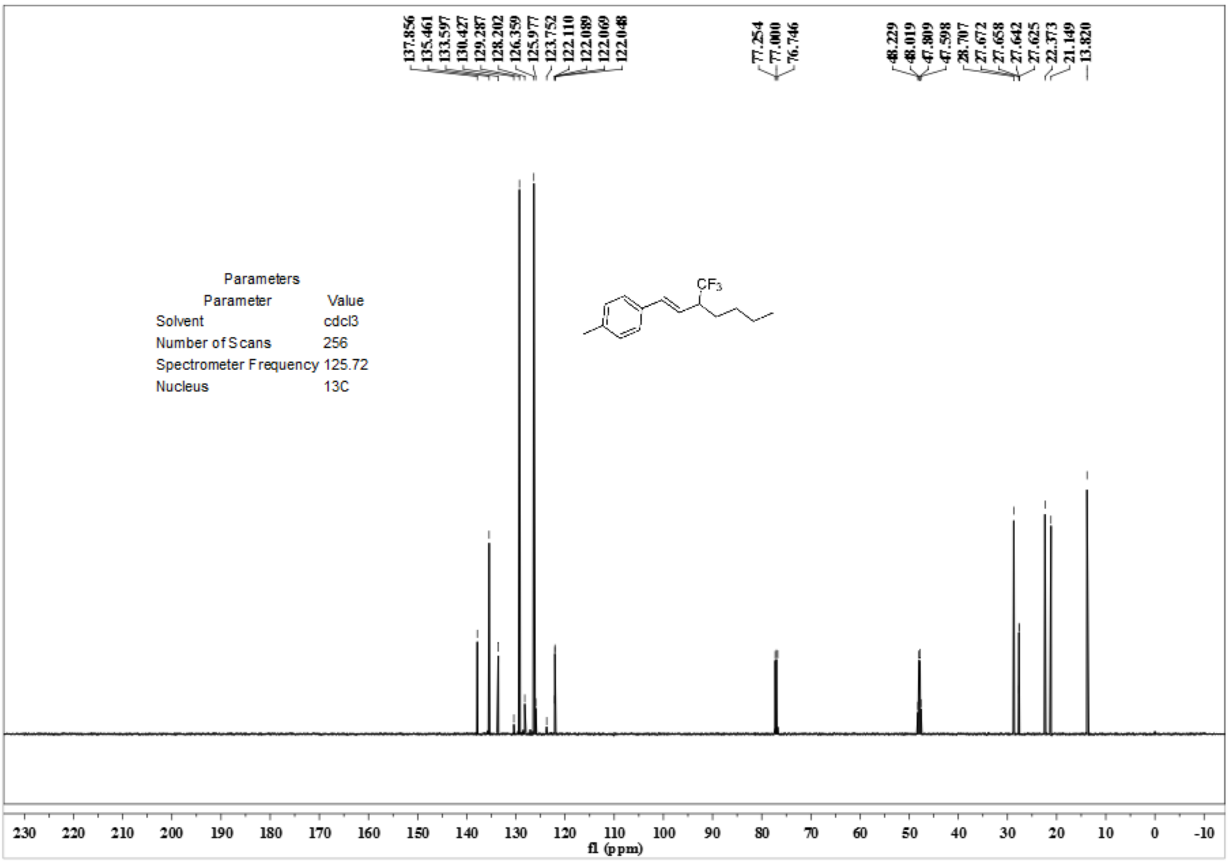

(*E*)-1-Methyl-2-(3-(trifluoromethyl)hept-1-en-1-yl)benzene (3c).

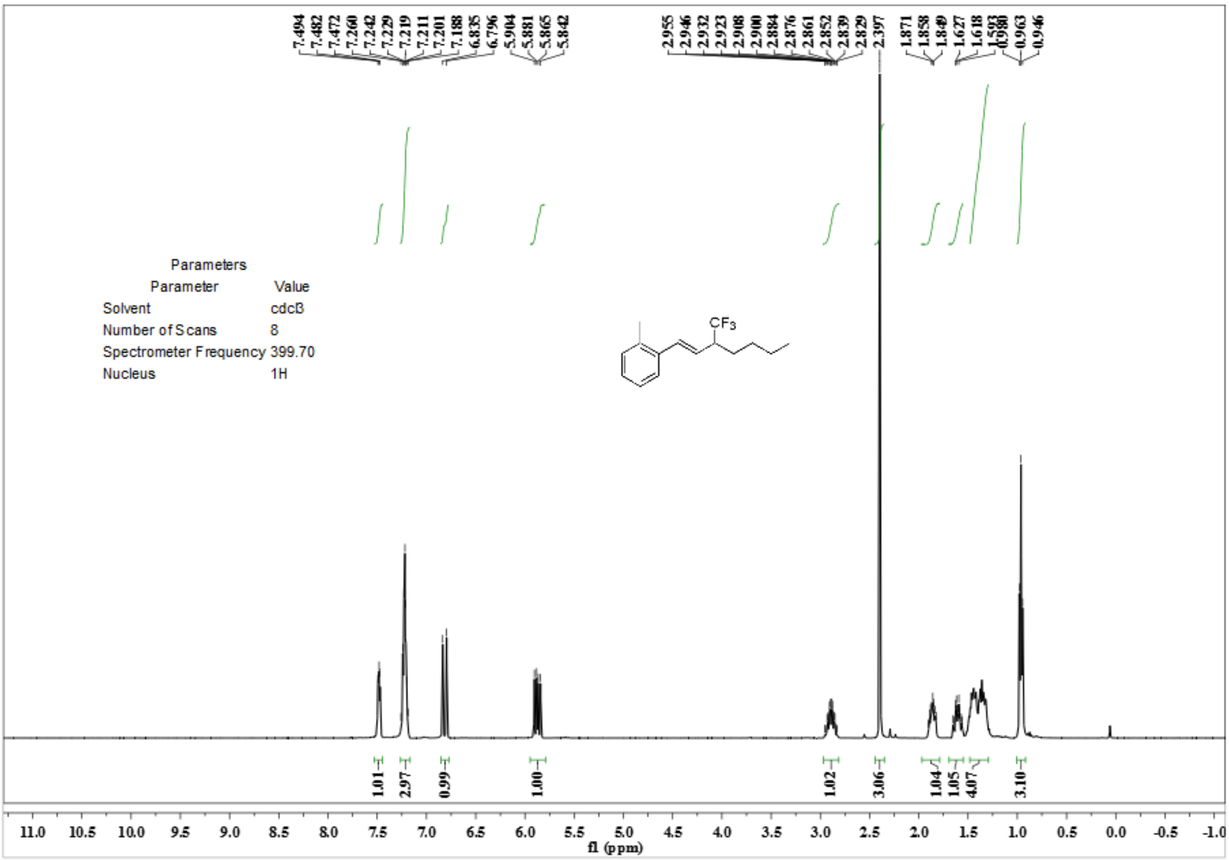

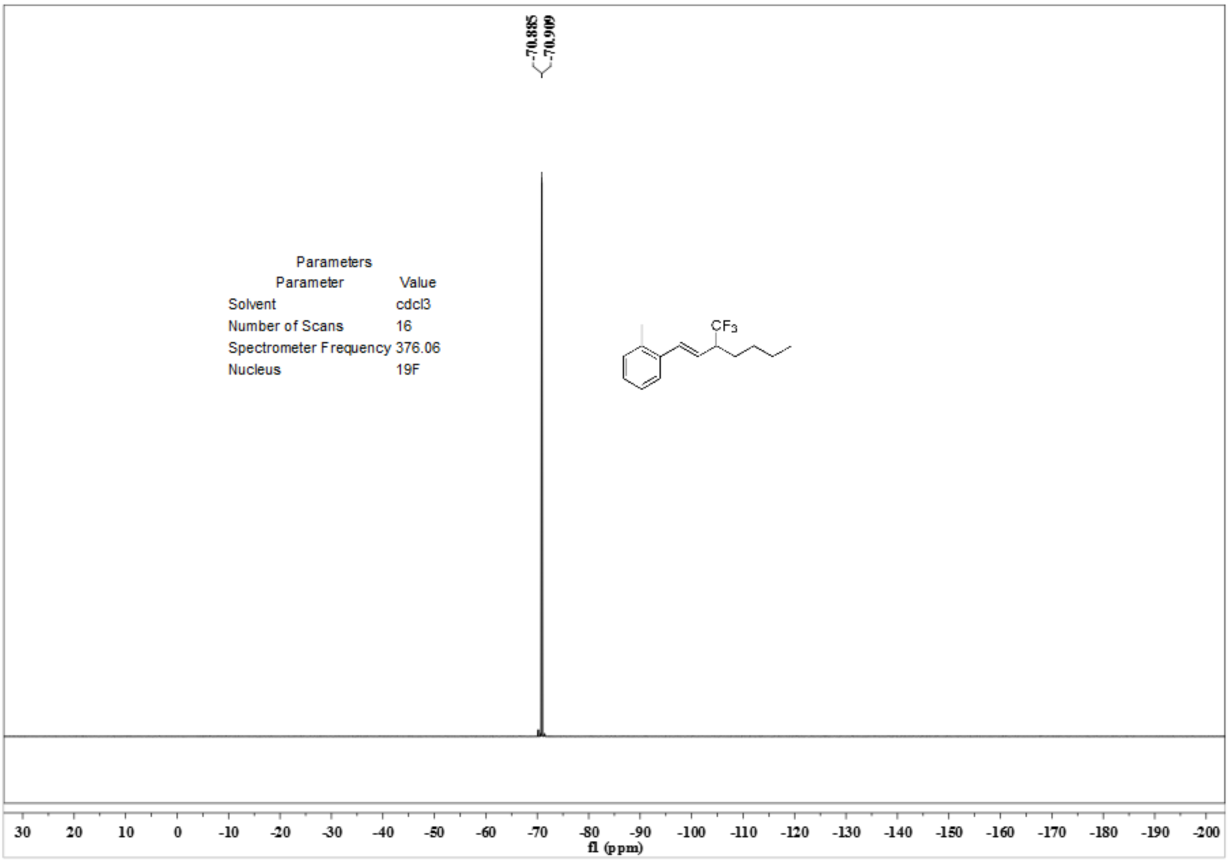

-70.885
-70.909
Parameters
Parameter Value
Solvent cdcl3
Number of Scans 16
Spectrometer Frequency 376.06
Nucleus 19F
CF3
fl (ppm)

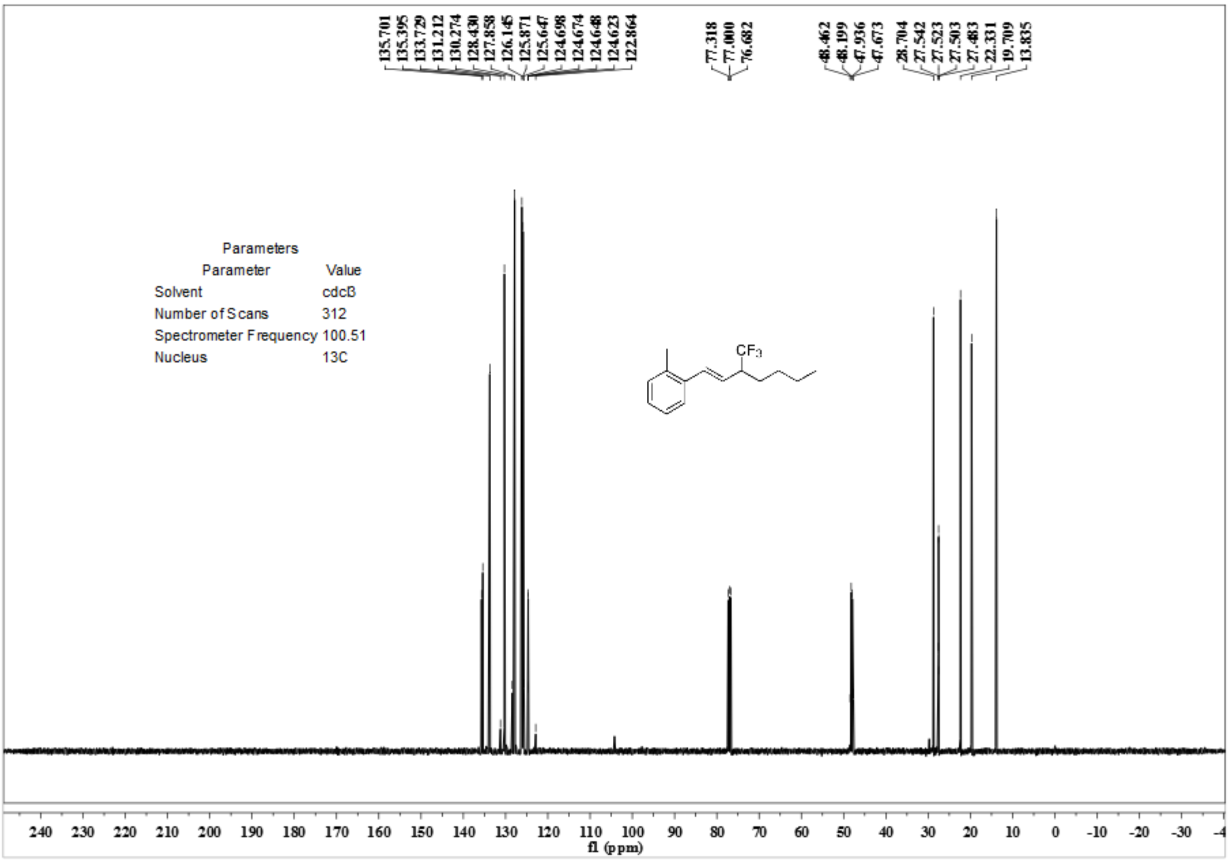

135.701
135.395
133.729
131.212
130.274
128.430
127.858
126.145
125.871
125.647
124.698
124.674
124.648
124.623
122.864
77.318
77.000
76.682
48.462
48.199
47.936
47.673
28.704
27.542
27.523
27.503
27.483
22.331
19.709
13.835
Parameters
Parameter Value
Solvent cdcl3
Number of Scans 312
Spectrometer Frequency 100.51
Nucleus 13C
CF3
fl (ppm)

(*E*)-1-(*tert*-Butyl)-4-(3-(trifluoromethyl)hept-1-en-1-yl)benzene (3d).

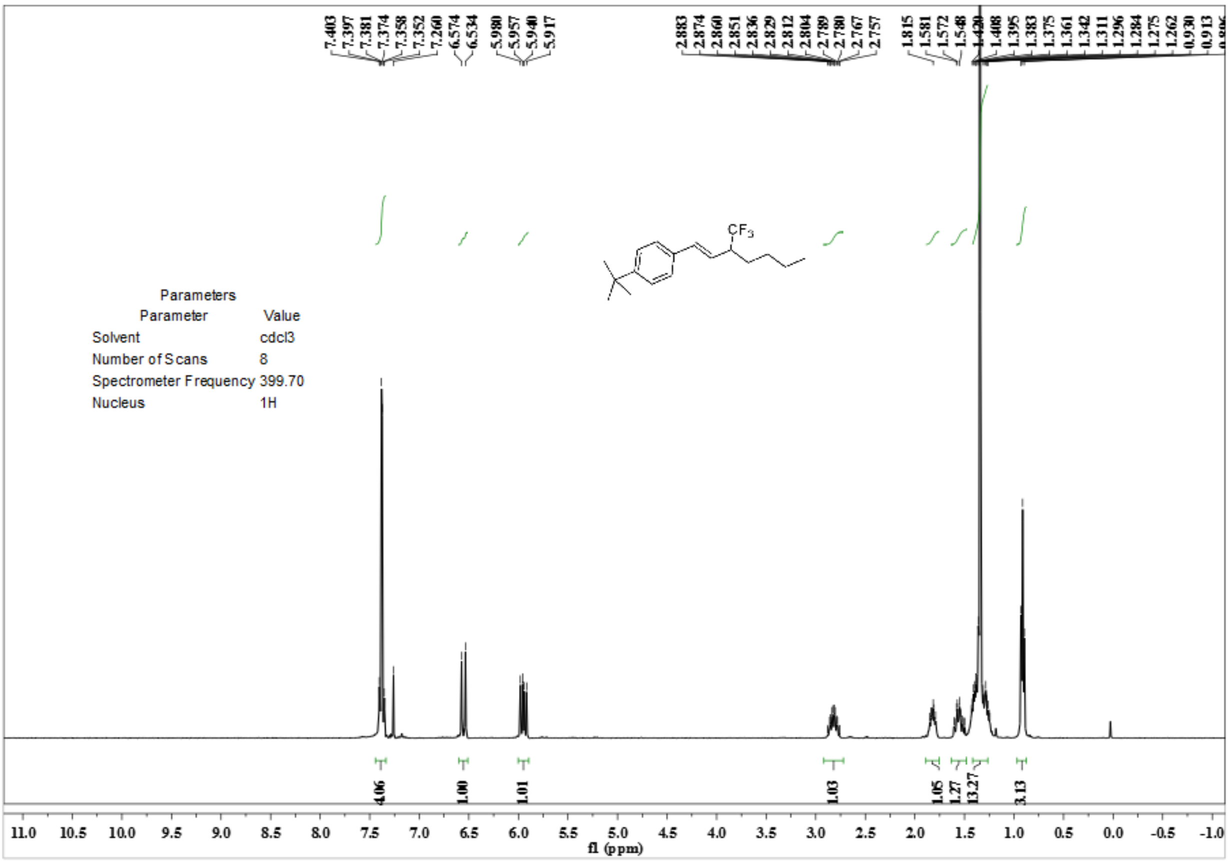

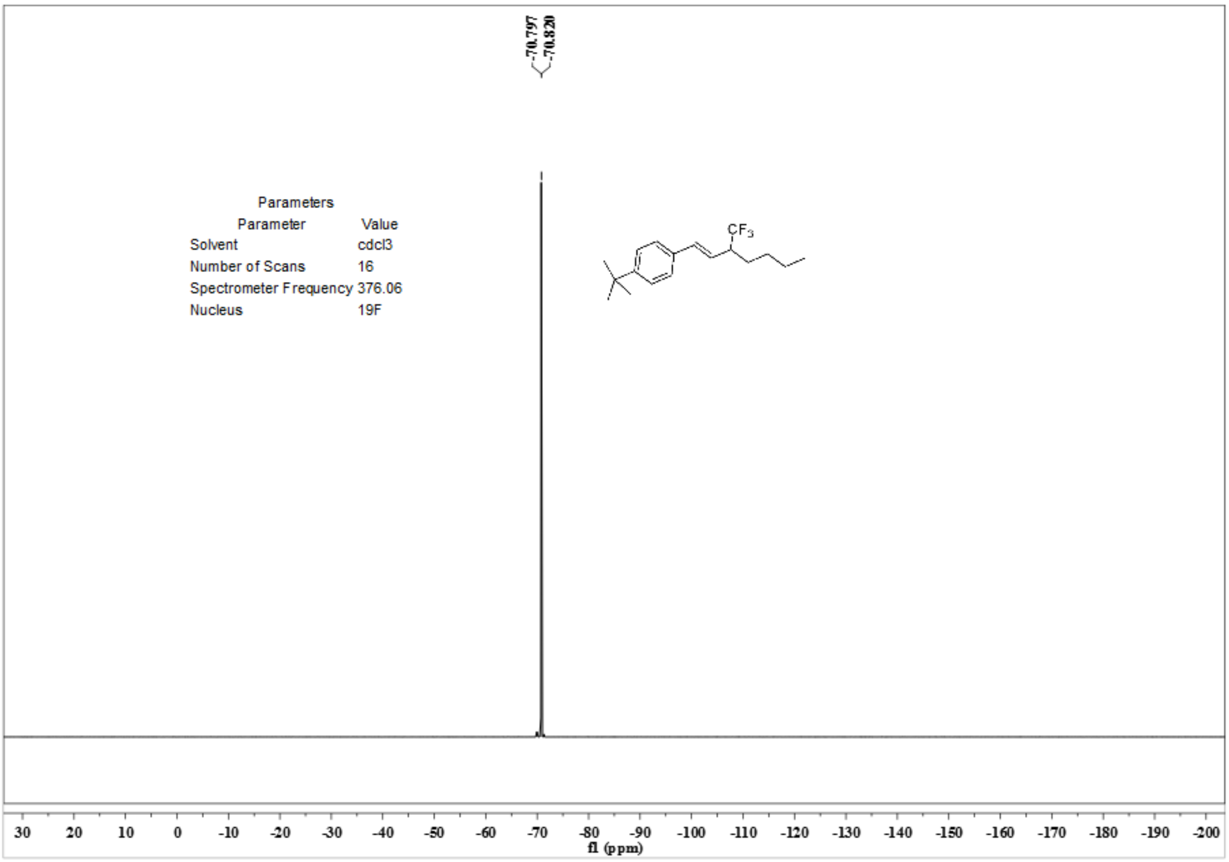

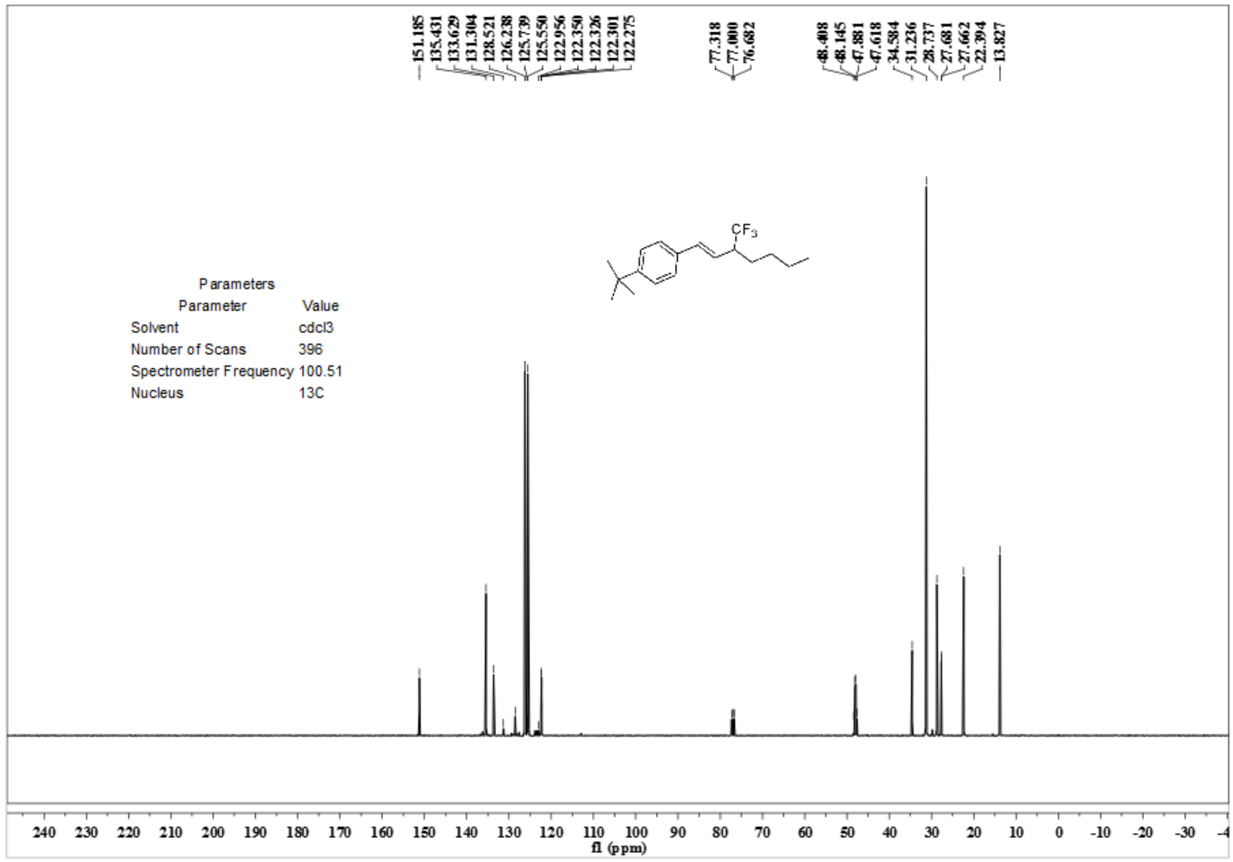

(*E*)-1-Methoxy-4-(3-(trifluoromethyl)hept-1-en-1-yl)benzene (3e).

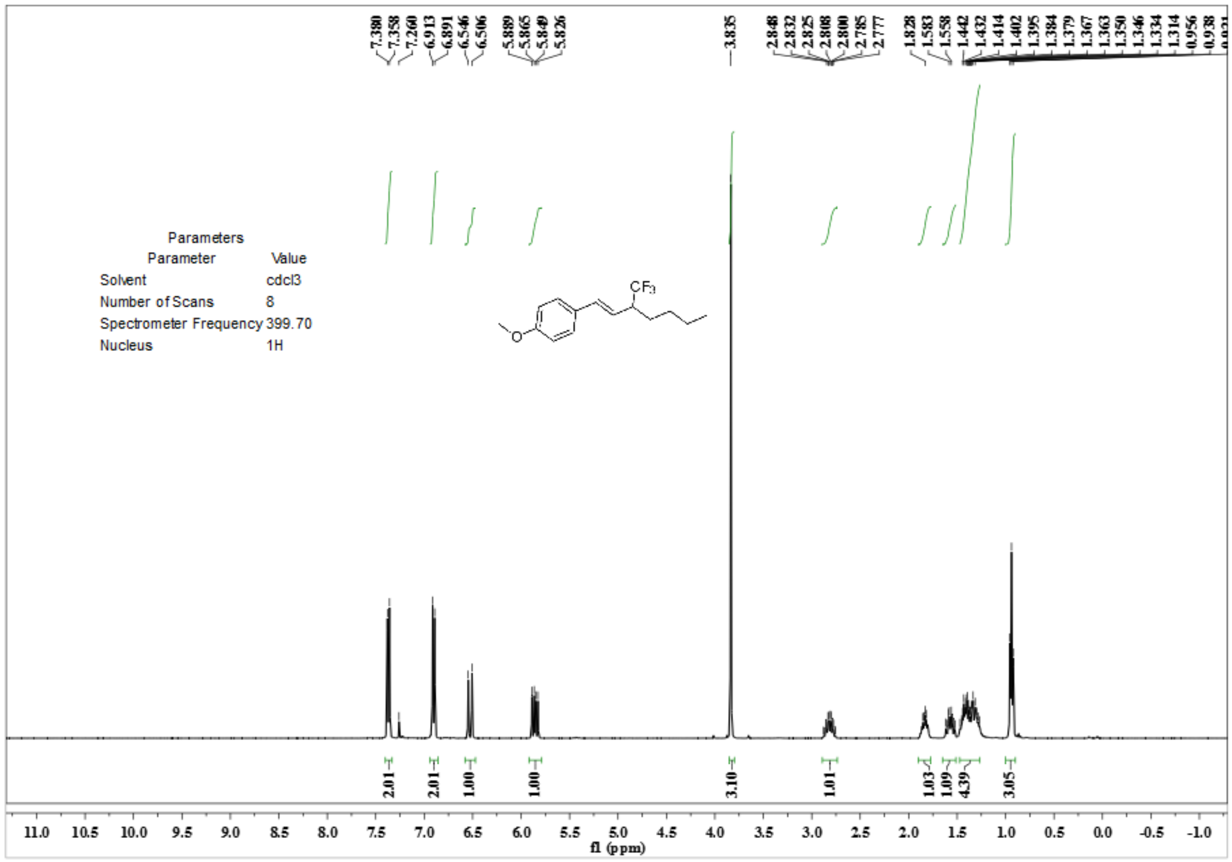

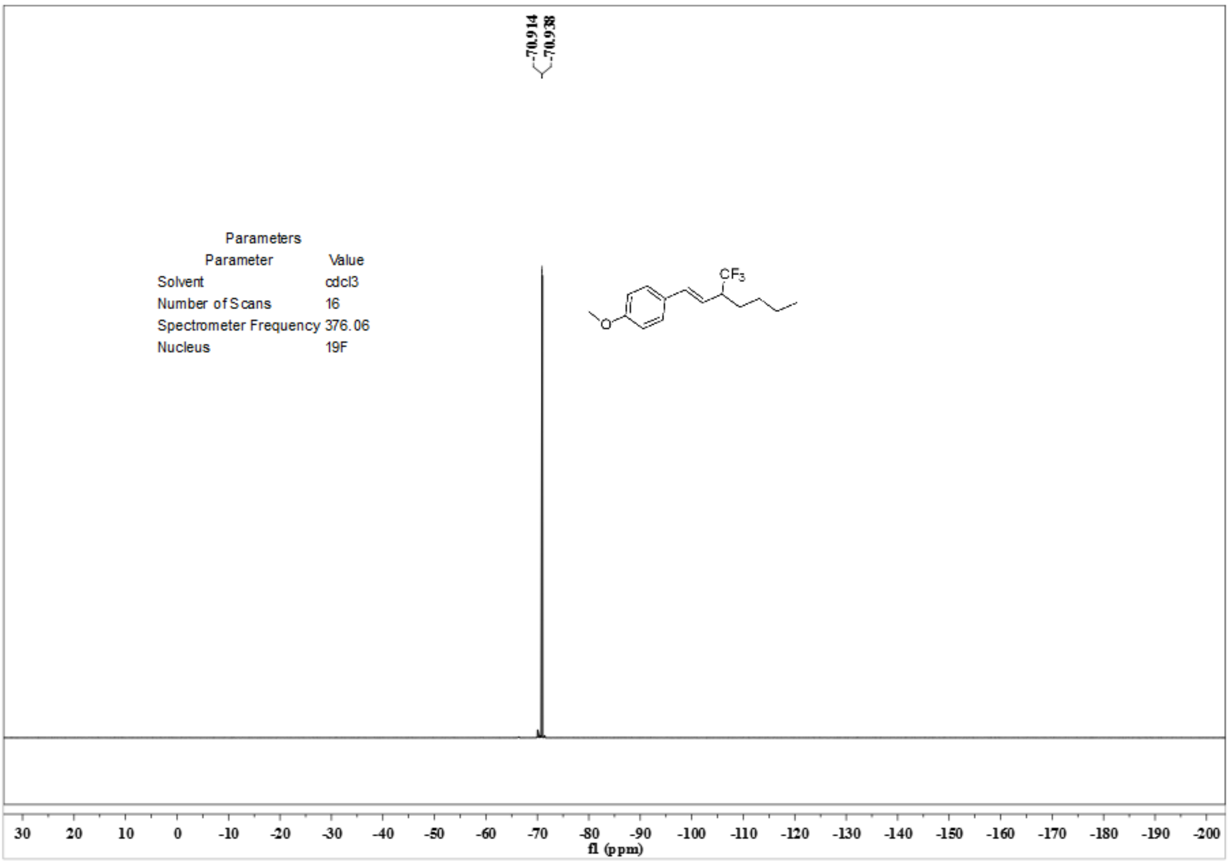

-70.914
-70.938
Parameters
Parameter Value
Solvent cdcl3
Number of Scans 16
Spectrometer Frequency 376.06
Nucleus 19F
CF3
O
30 20 10 0 -10 -20 -30 -40 -50 -60 -70 -80 -90 -100 -110 -120 -130 -140 -150 -160 -170 -180 -190 -200
f1 (ppm)

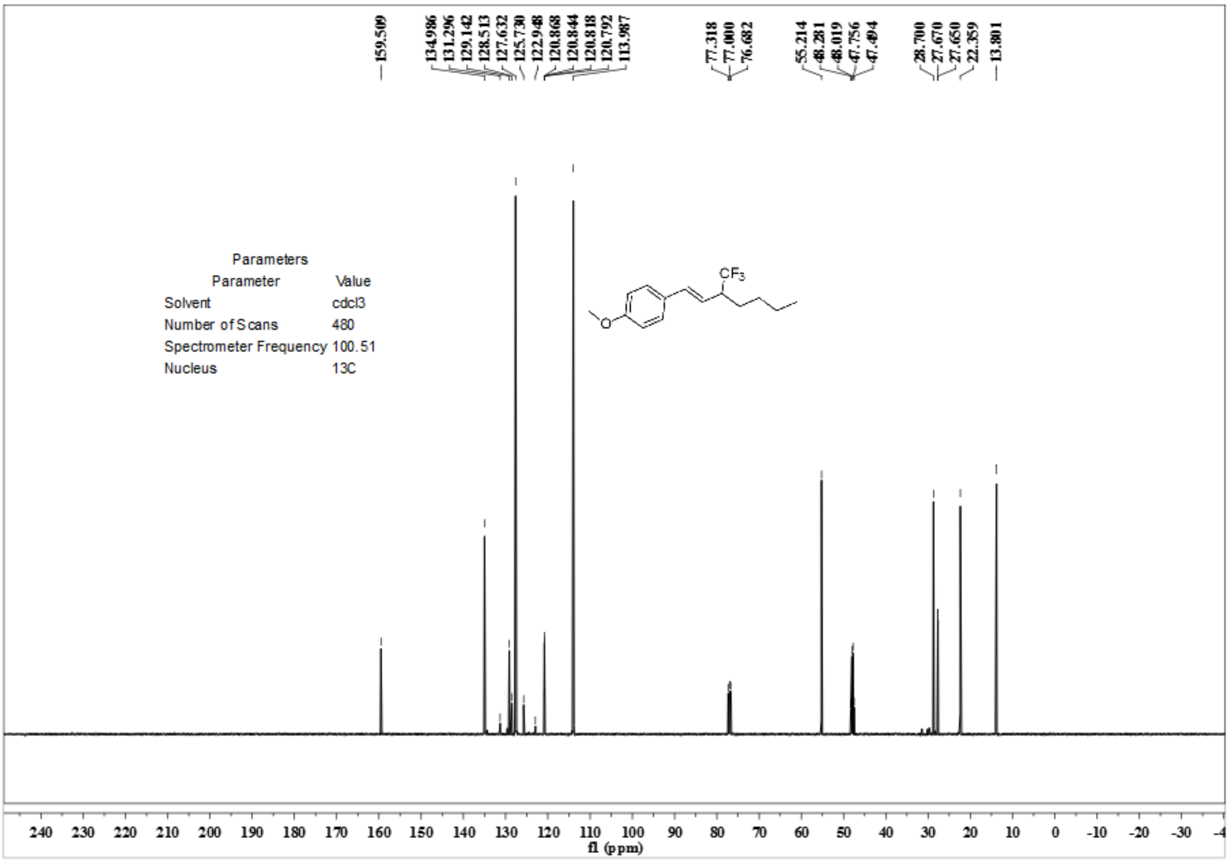

159.509
134.986
131.296
129.142
128.513
127.632
125.730
122.948
120.868
120.844
120.818
120.792
113.987
77.318
77.000
76.682
55.214
48.281
48.019
47.756
47.494
28.700
27.670
27.650
22.359
13.801
Parameters
Parameter Value
Solvent cdcl3
Number of Scans 480
Spectrometer Frequency 100.51
Nucleus 13C
CF3
O
240 230 220 210 200 190 180 170 160 150 140 130 120 110 100 90 80 70 60 50 40 30 20 10 0 -10 -20 -30
f1 (ppm)

(*E*)-4-(3-(Trifluoromethyl)hept-1-en-1-yl)phenyl acetate (3f).

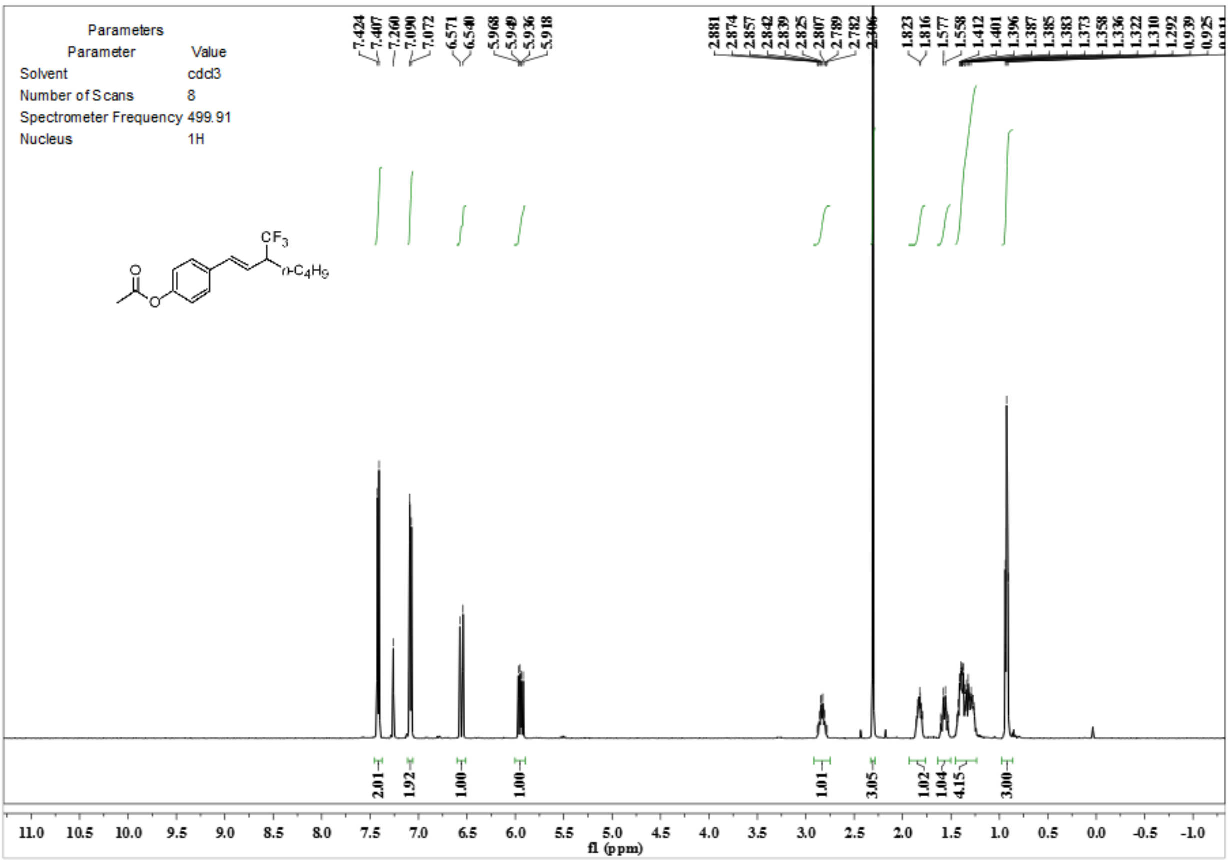

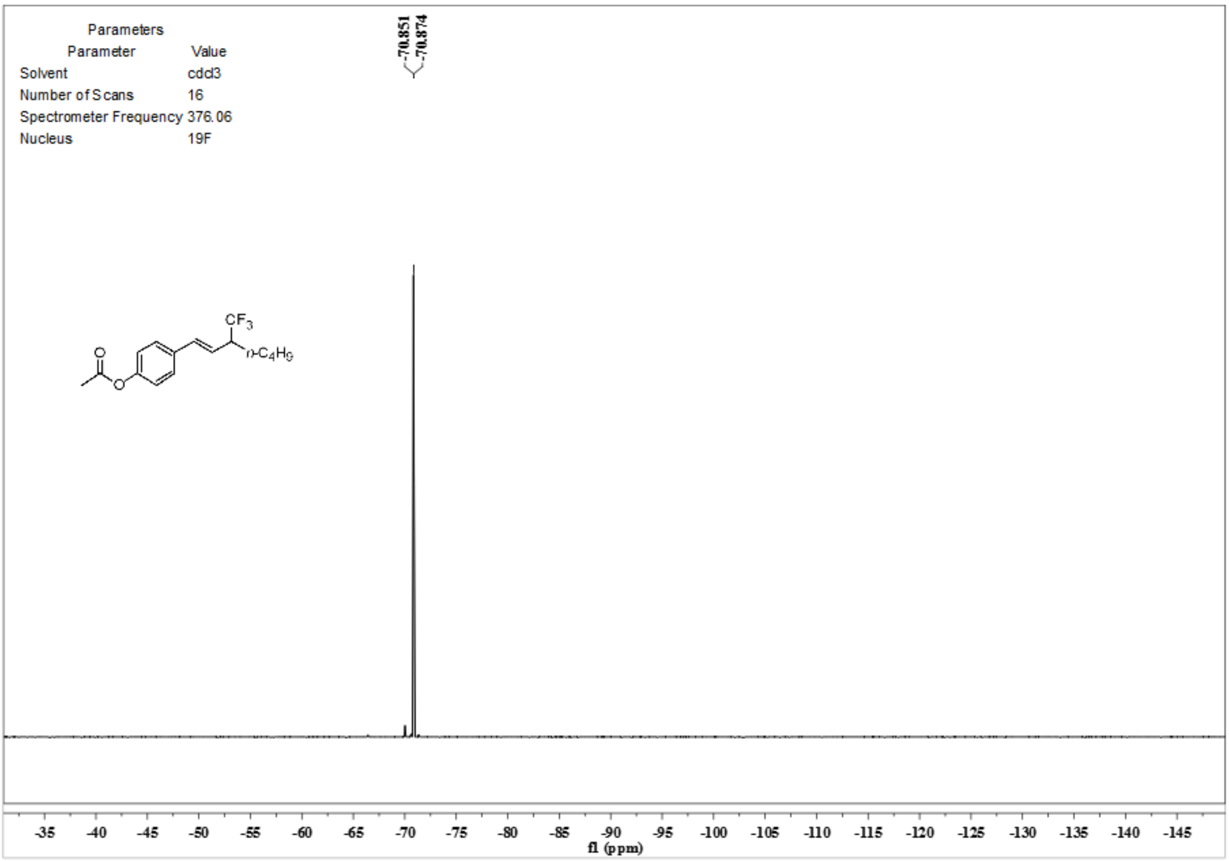

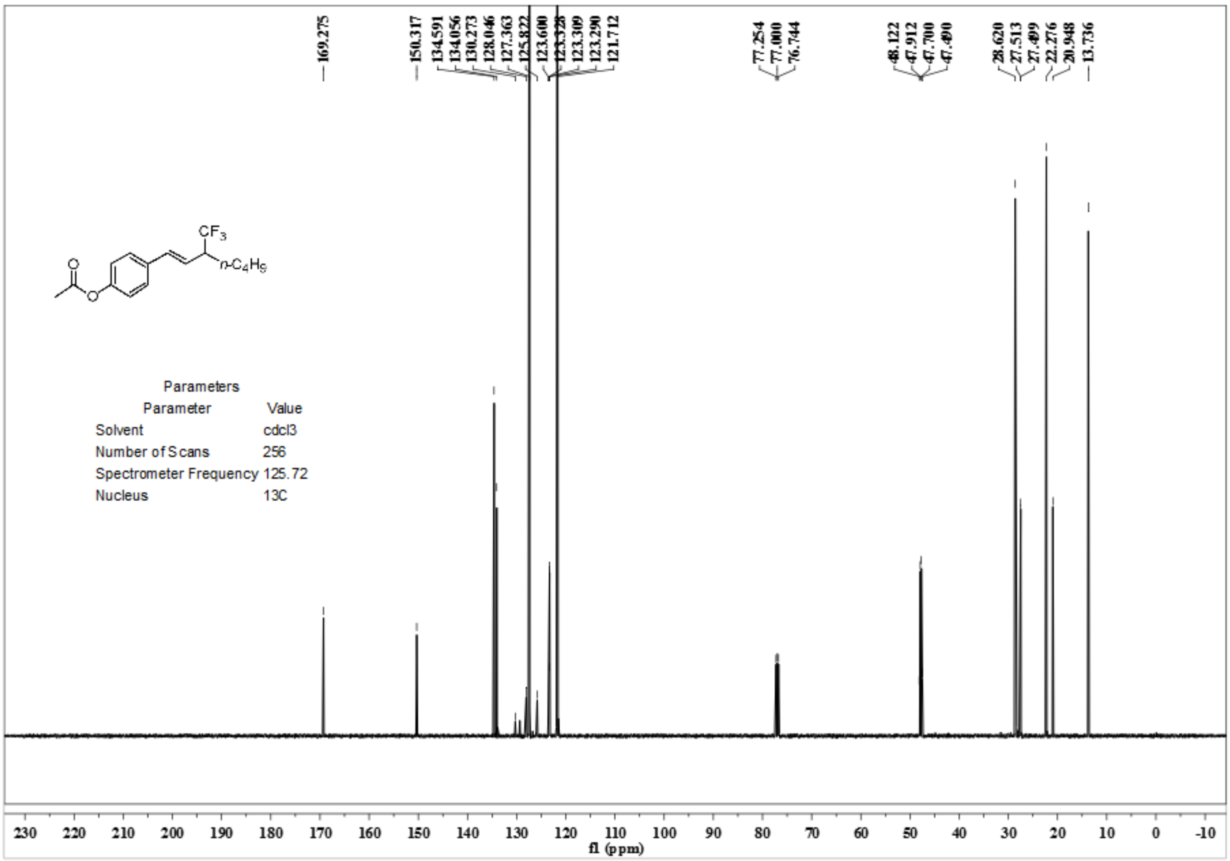

Methyl (*E*)-4-(3-(trifluoromethyl)hept-1-en-1-yl)benzoate (3g).

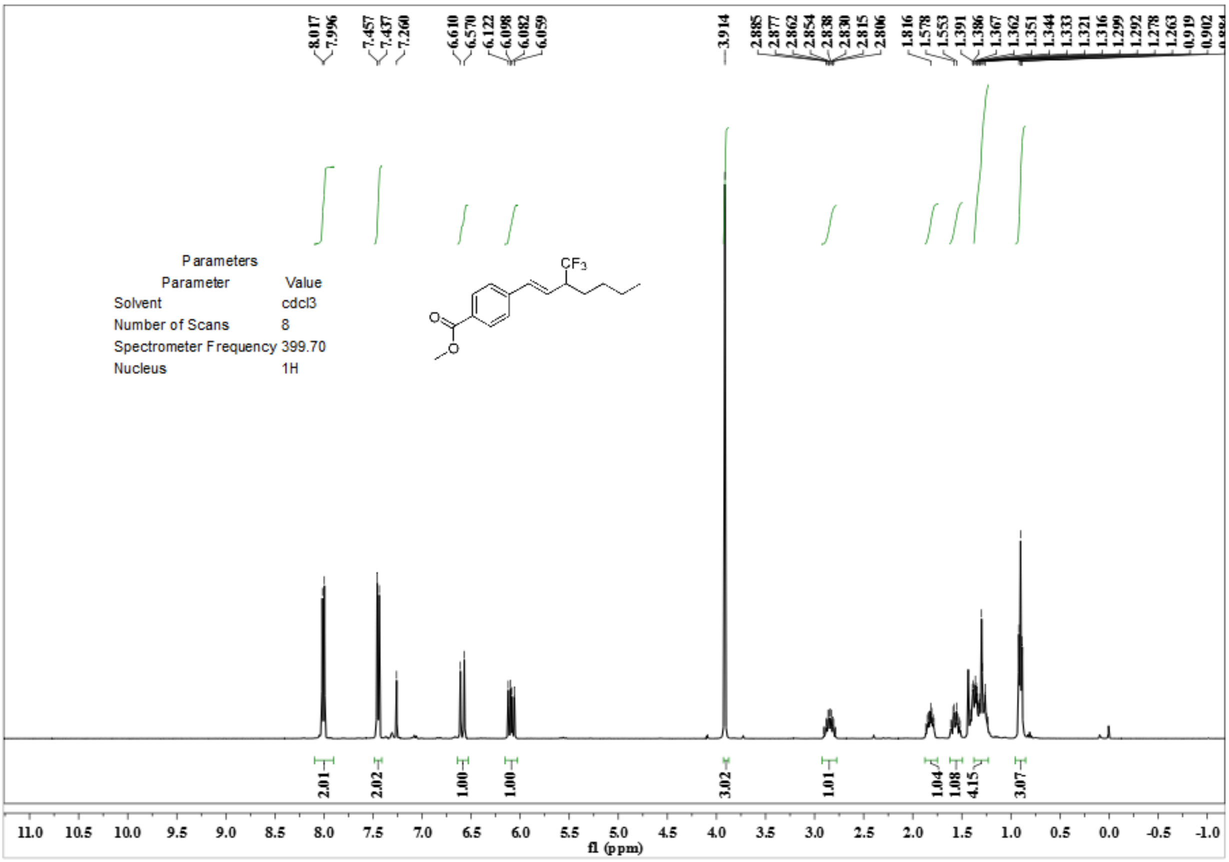

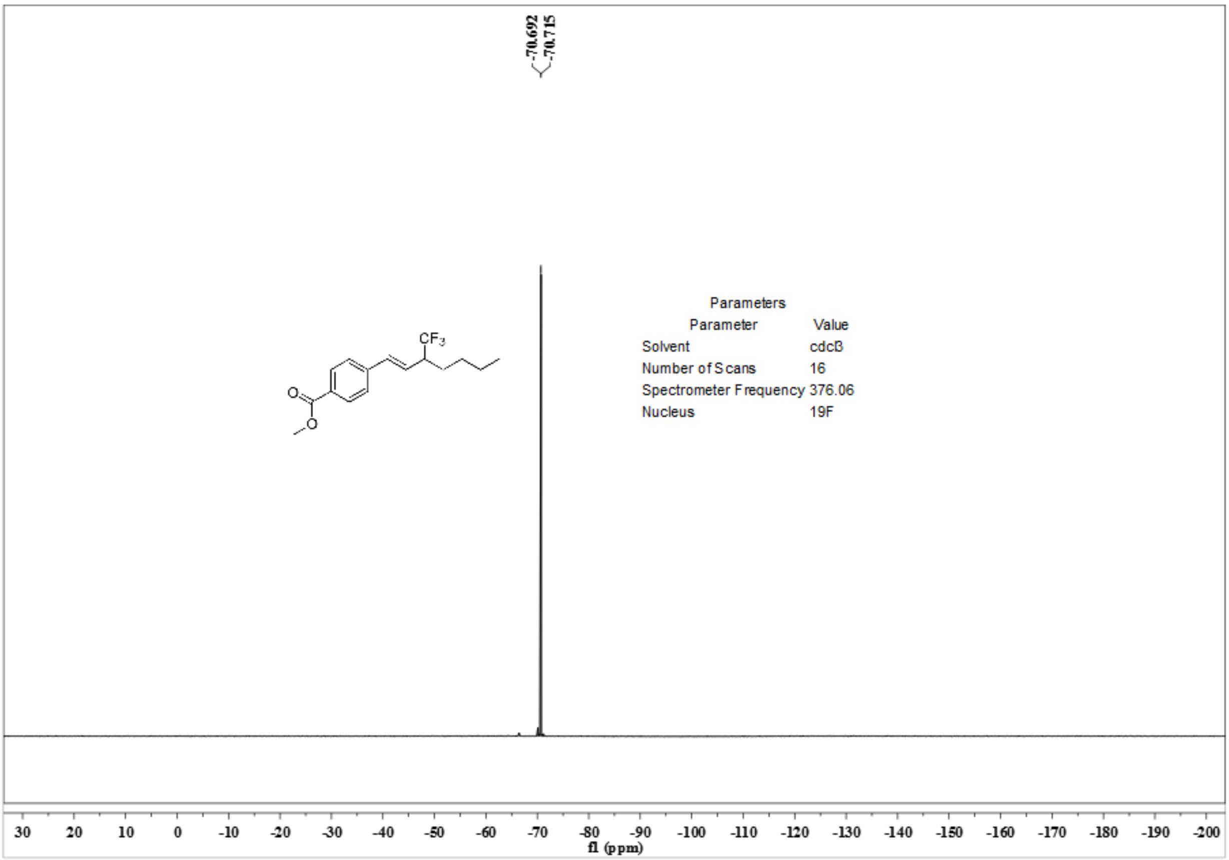
-70.692
-70.715
Parameters
Parameter Value
Solvent cdcl3
Number of Scans 16
Spectrometer Frequency 376.06
Nucleus 19F
CF3
O
O
fl (ppm)

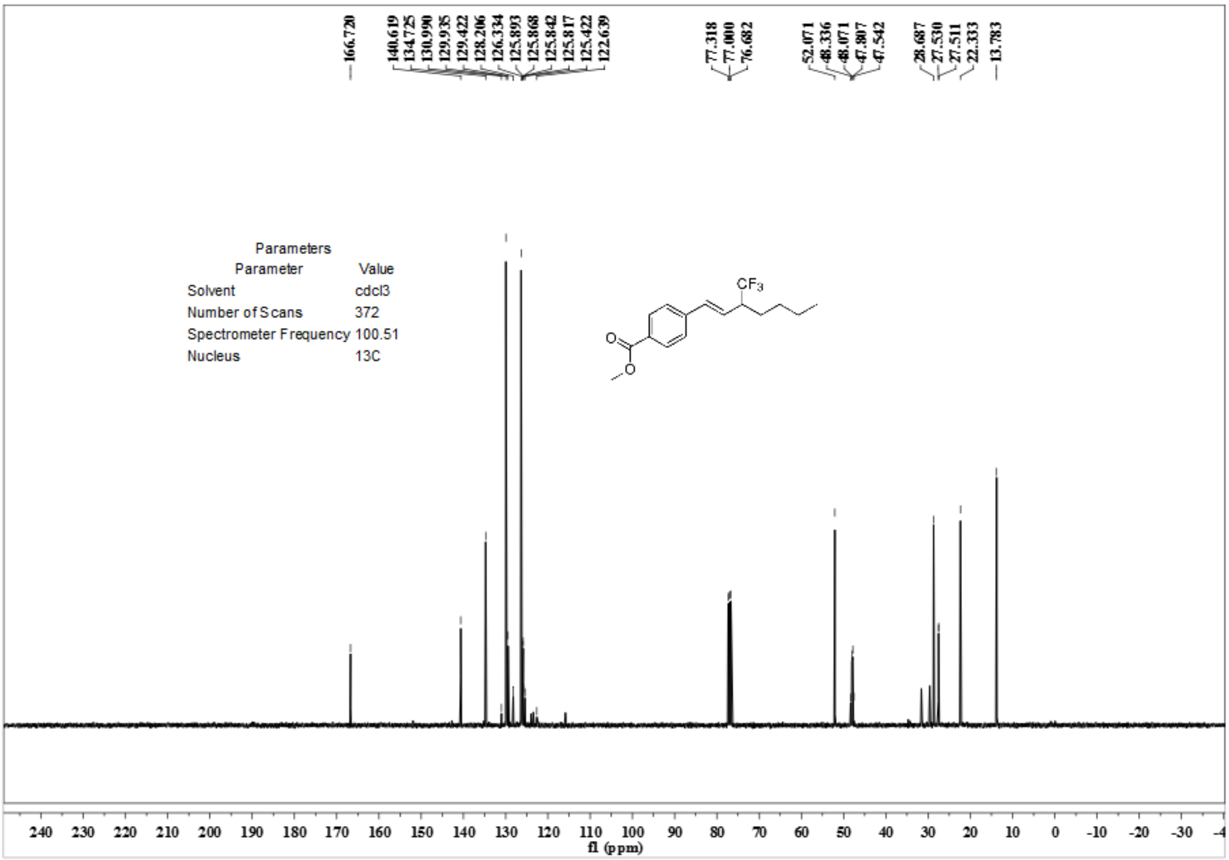
166.720
140.619
134.725
130.990
129.935
129.422
128.206
126.334
125.893
125.868
125.842
125.817
125.422
122.639
77.318
77.000
76.682
52.071
48.336
48.071
47.807
47.542
28.687
27.530
27.511
22.333
13.783
Parameters
Parameter Value
Solvent cdcl3
Number of Scans 372
Spectrometer Frequency 100.51
Nucleus 13C
CF3
O
O
fl (ppm)

(*E*)-4-(3-(Trifluoromethyl)hept-1-en-1-yl)benzonitrile (3h).

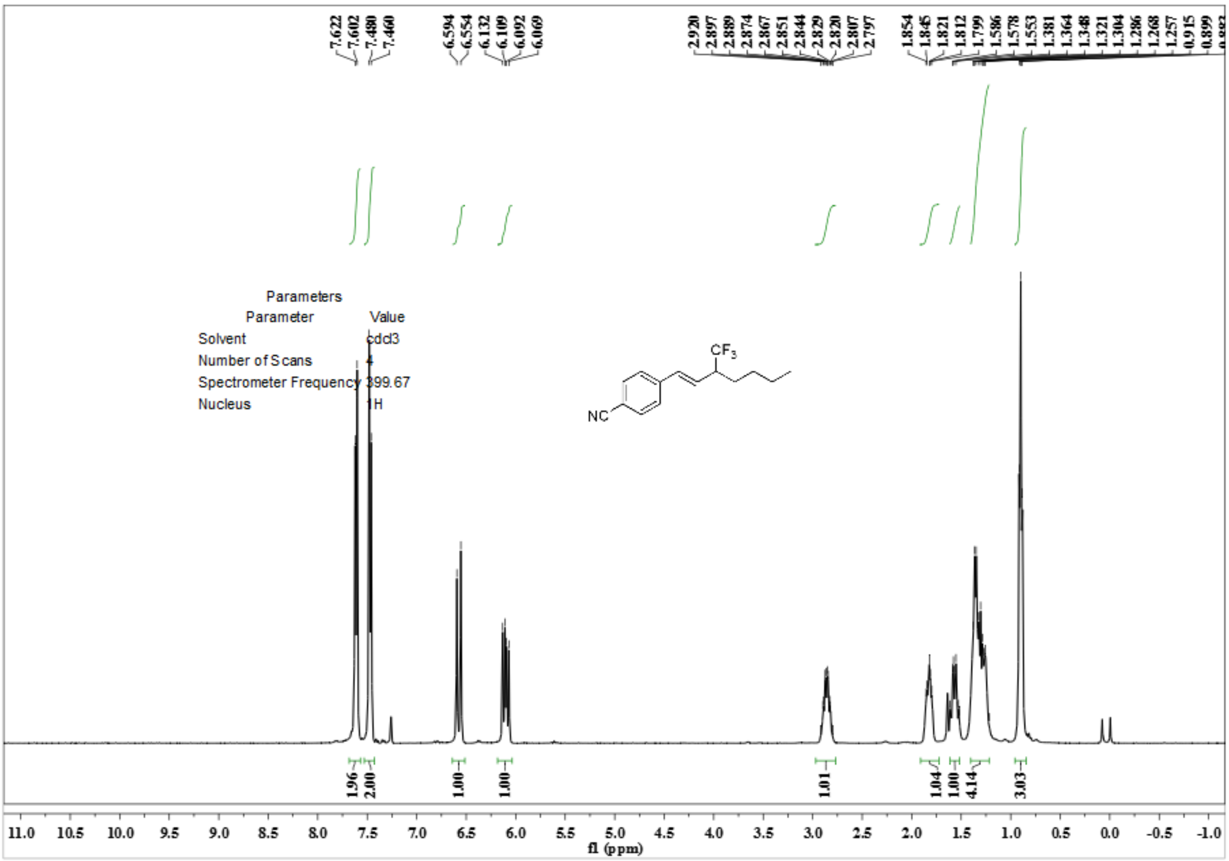

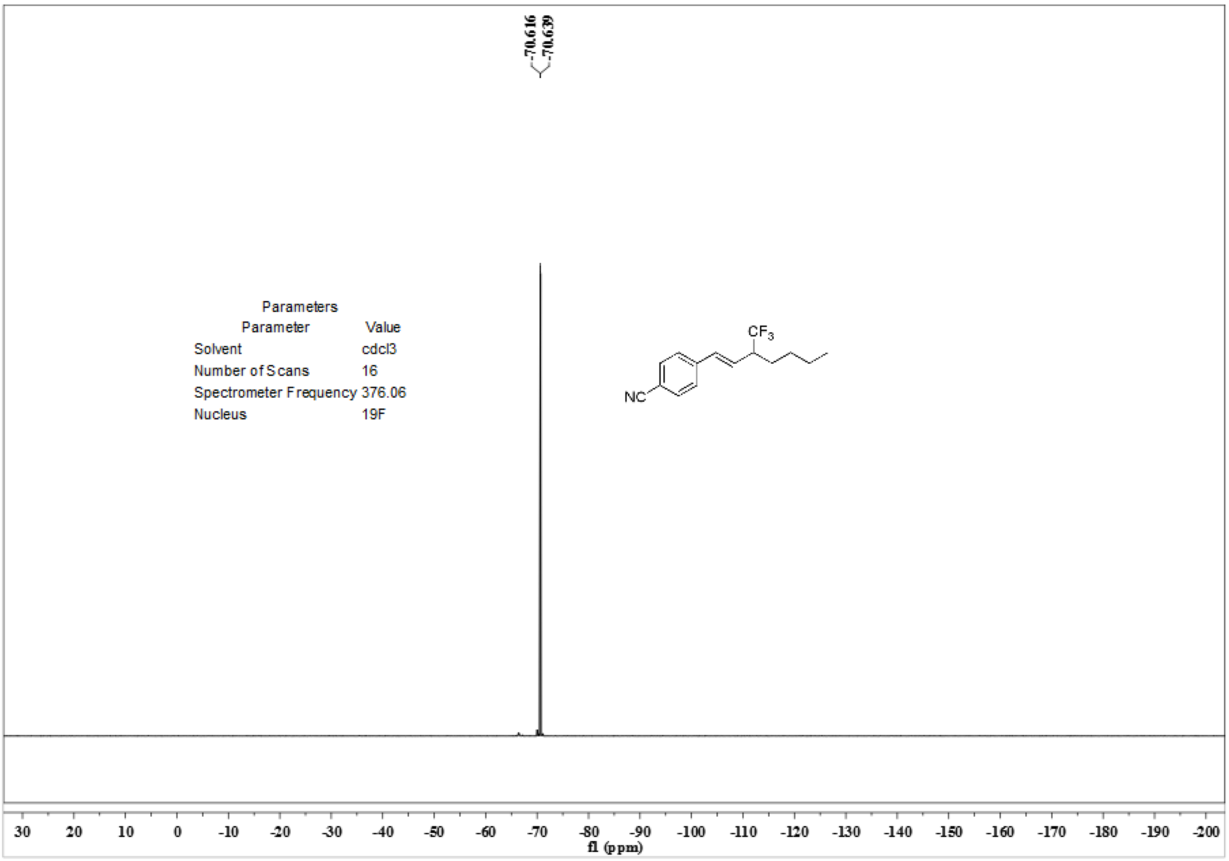

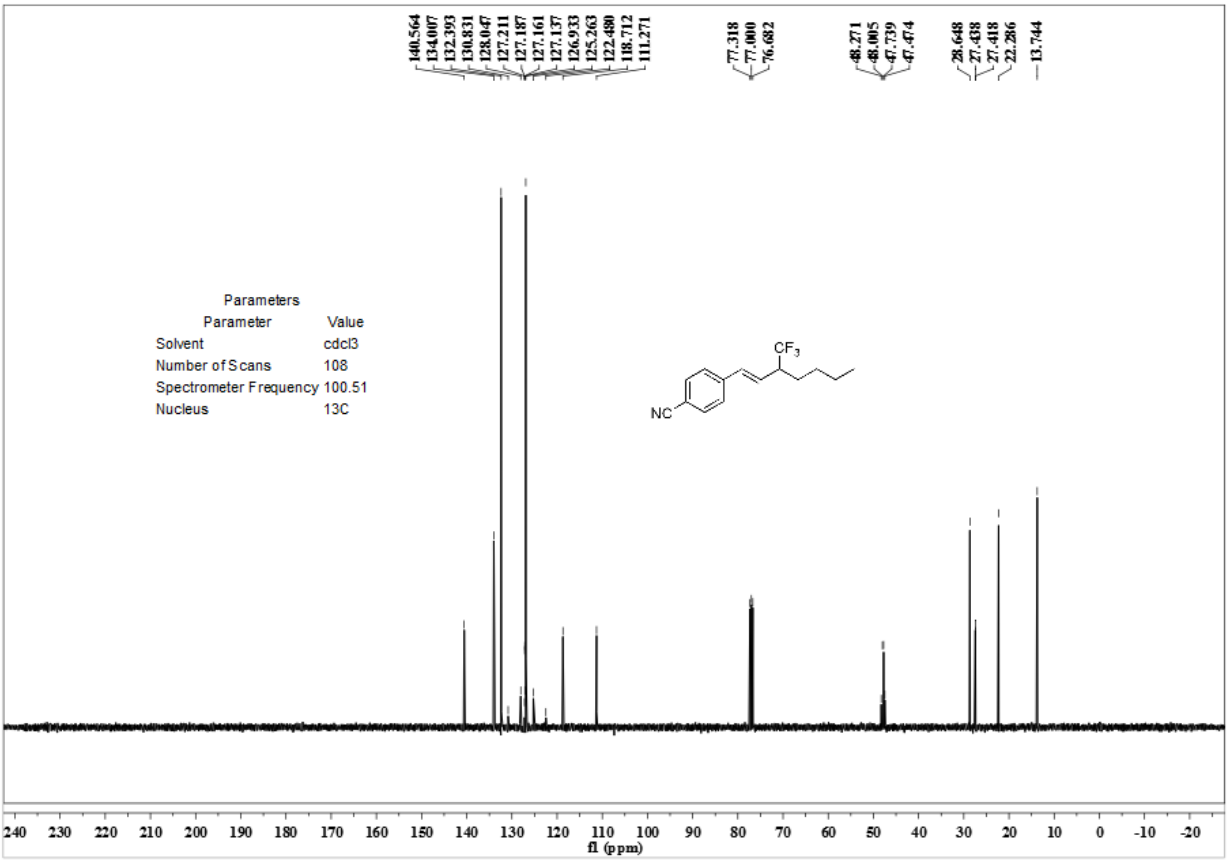

(*E*)-1-Fluoro-4-(3-(trifluoromethyl)hept-1-en-1-yl)benzene (3i).

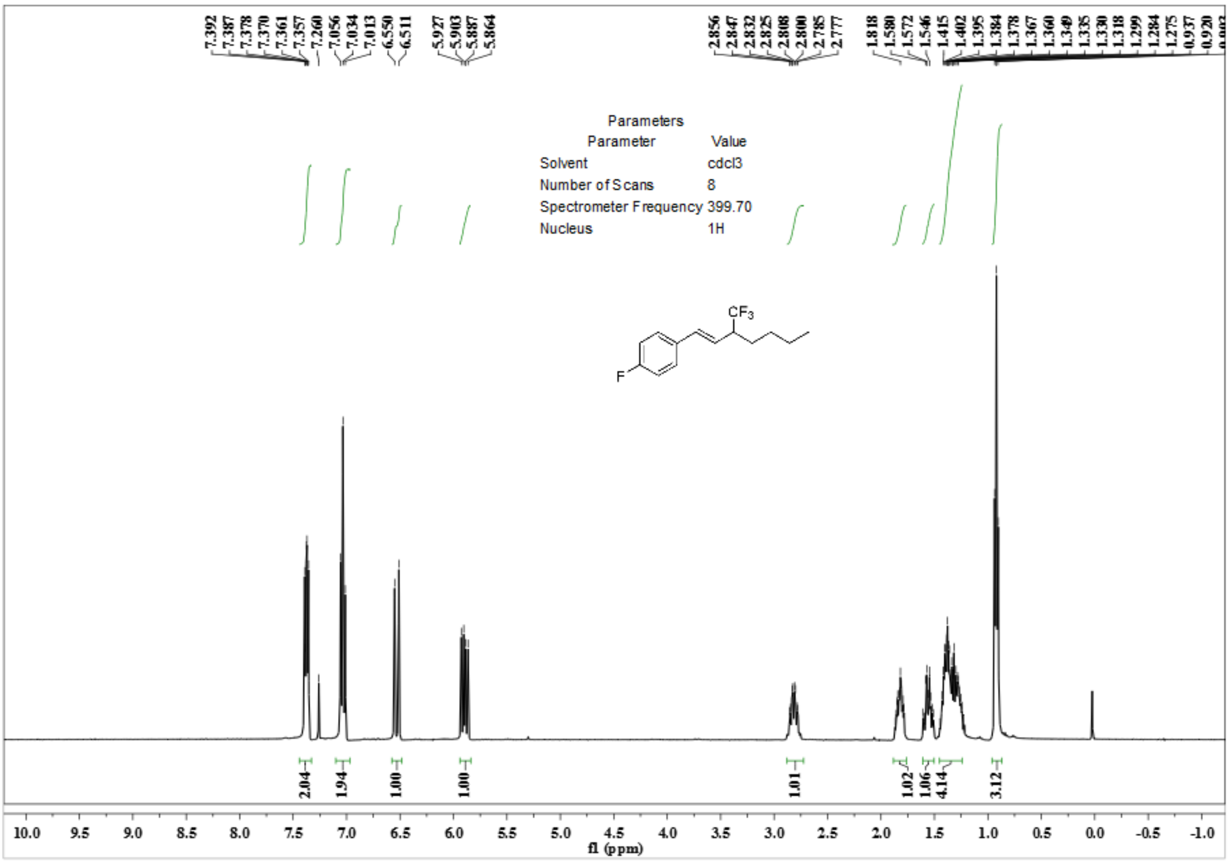

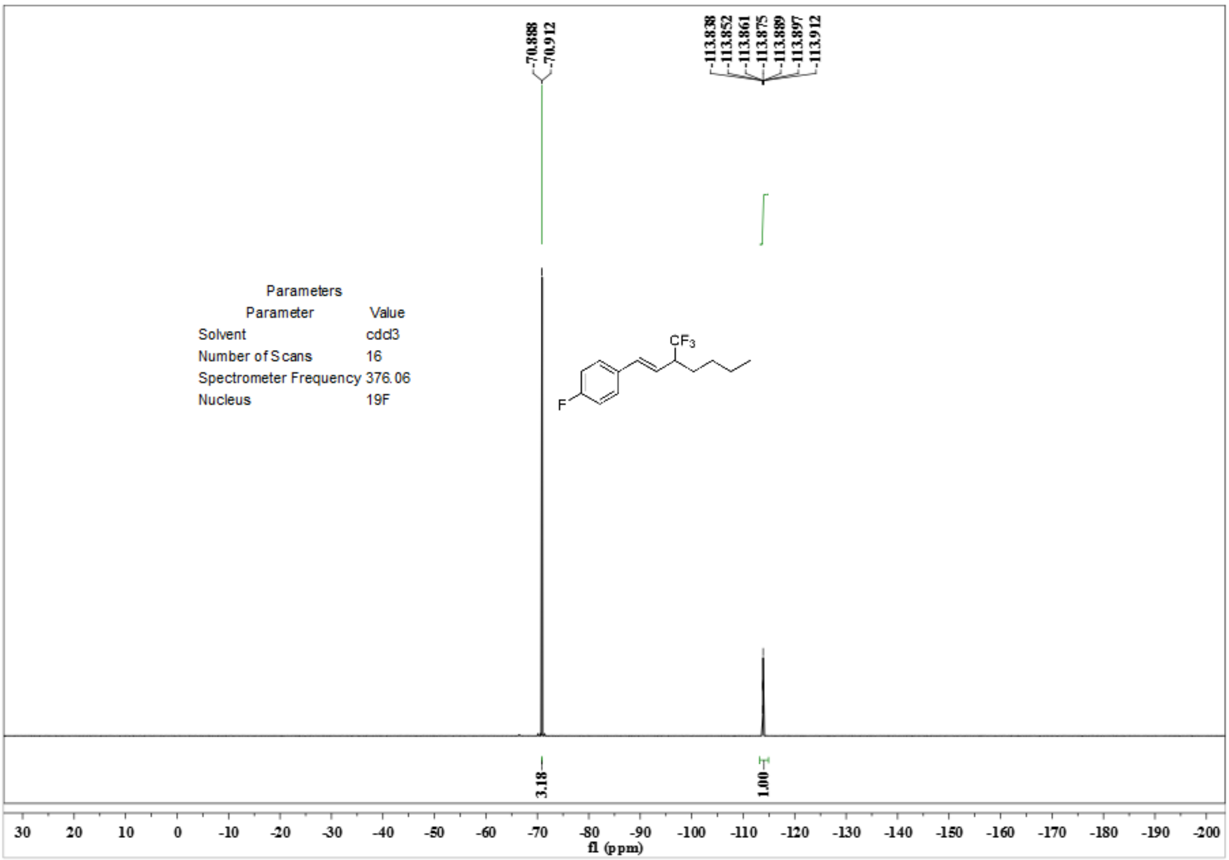

-70.888
-70.912
-113.838
-113.852
-113.861
-113.875
-113.889
-113.897
-113.912
Parameters
Parameter Value
Solvent cdcl3
Number of Scans 16
Spectrometer Frequency 376.06
Nucleus 19F
CF3
F
3.18
1.00
30 20 10 0 -10 -20 -30 -40 -50 -60 -70 -80 -90 -100 -110 -120 -130 -140 -150 -160 -170 -180 -190 -200
f1 (ppm)

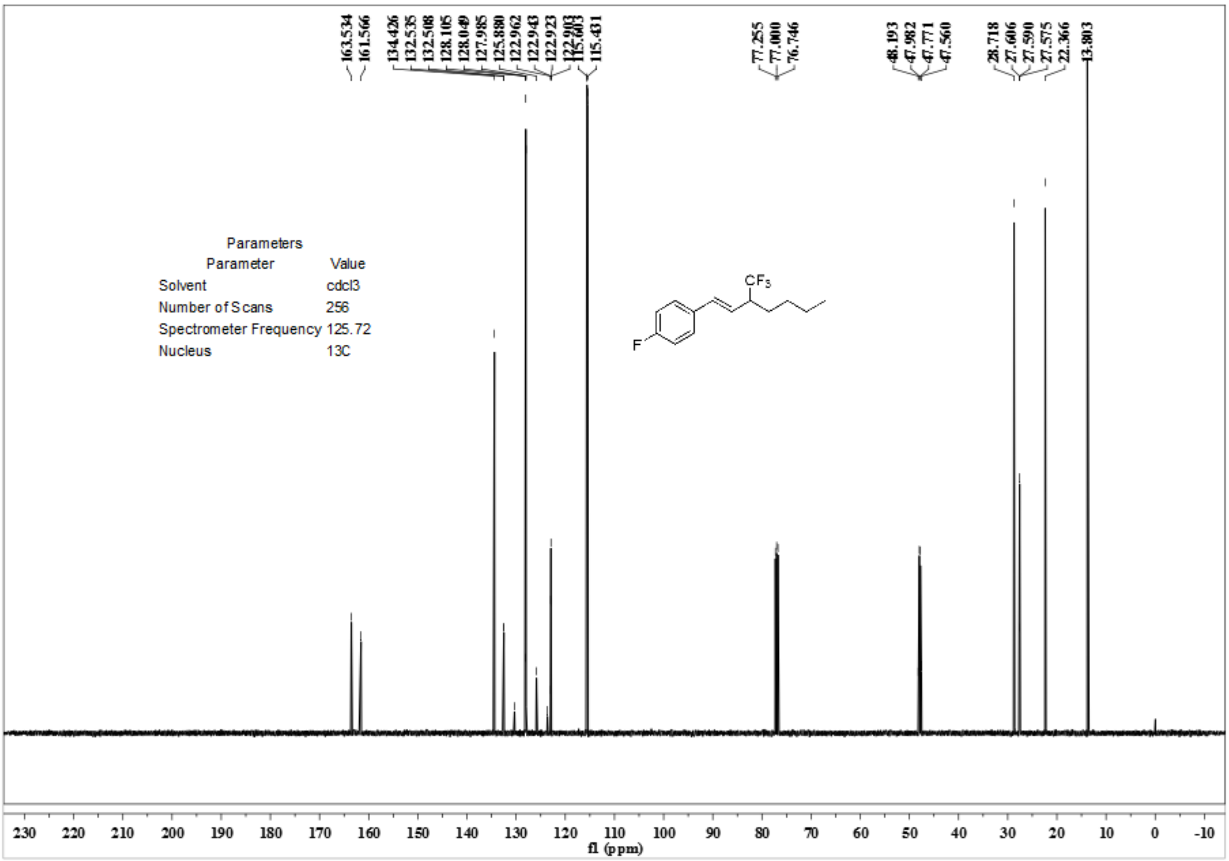

163.534
161.566
134.426
132.535
132.508
128.105
128.049
127.985
125.880
122.962
122.943
122.923
122.903
115.603
115.431
77.255
77.000
76.746
48.193
47.982
47.771
47.560
28.718
27.606
27.590
27.575
22.366
13.803
Parameters
Parameter Value
Solvent cdcl3
Number of Scans 256
Spectrometer Frequency 125.72
Nucleus 13C
CF3
F
230 220 210 200 190 180 170 160 150 140 130 120 110 100 90 80 70 60 50 40 30 20 10 0 -10
f1 (ppm)

(*E*)-1-Chloro-4-(3-(trifluoromethyl)hept-1-en-1-yl)benzene (3j).

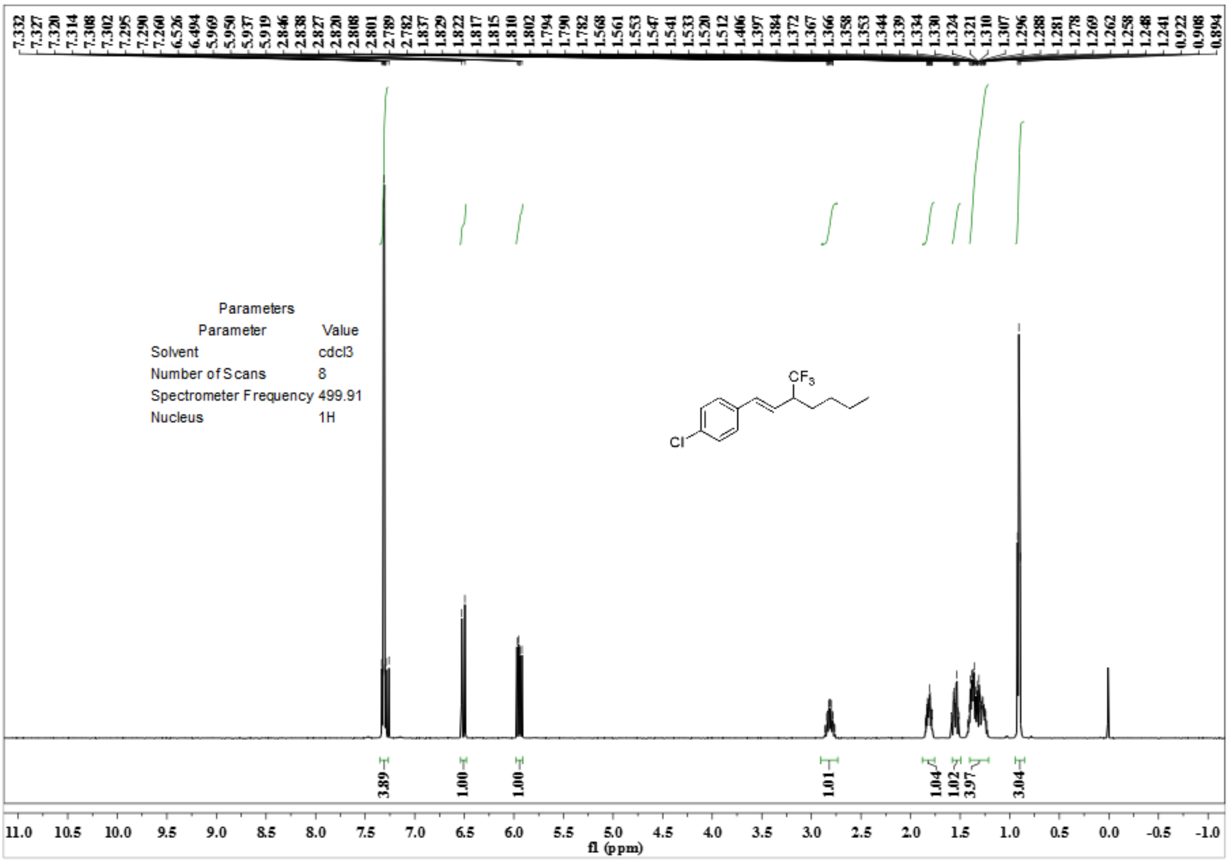

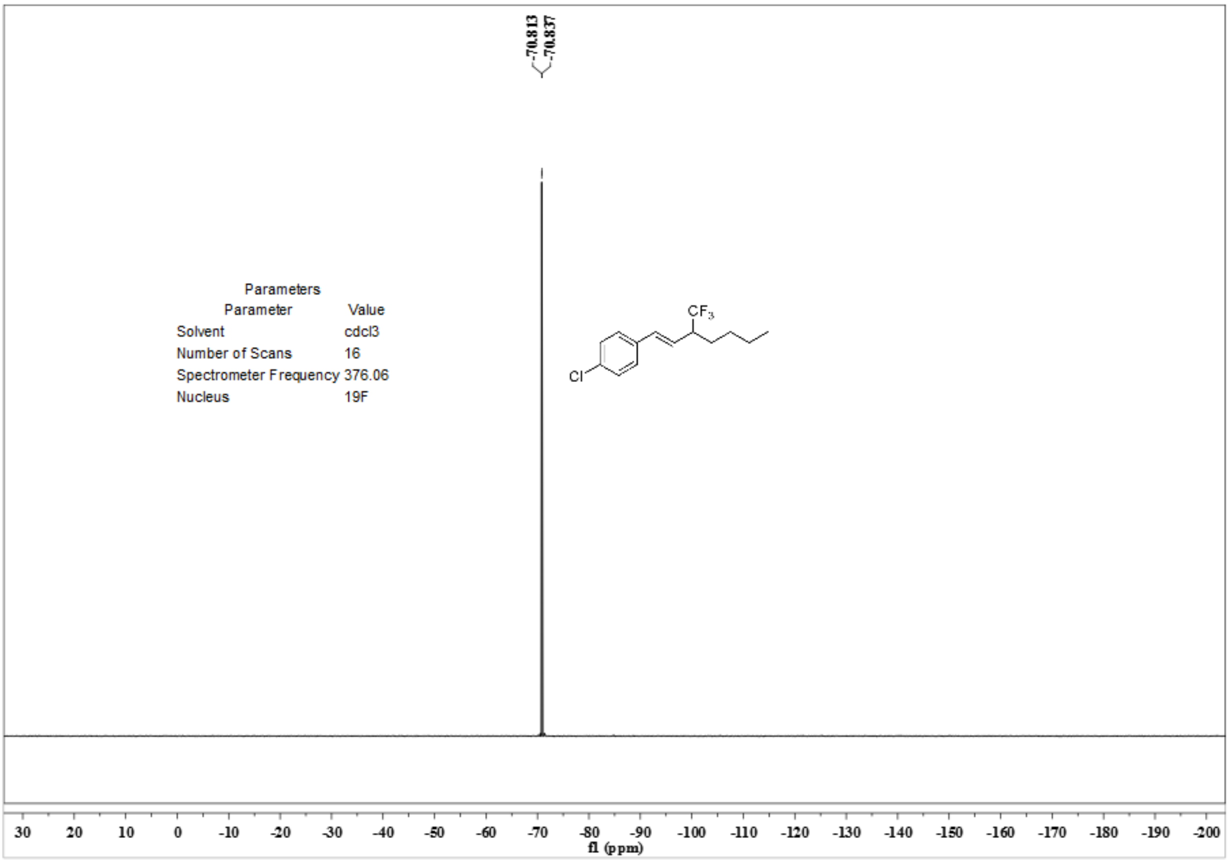

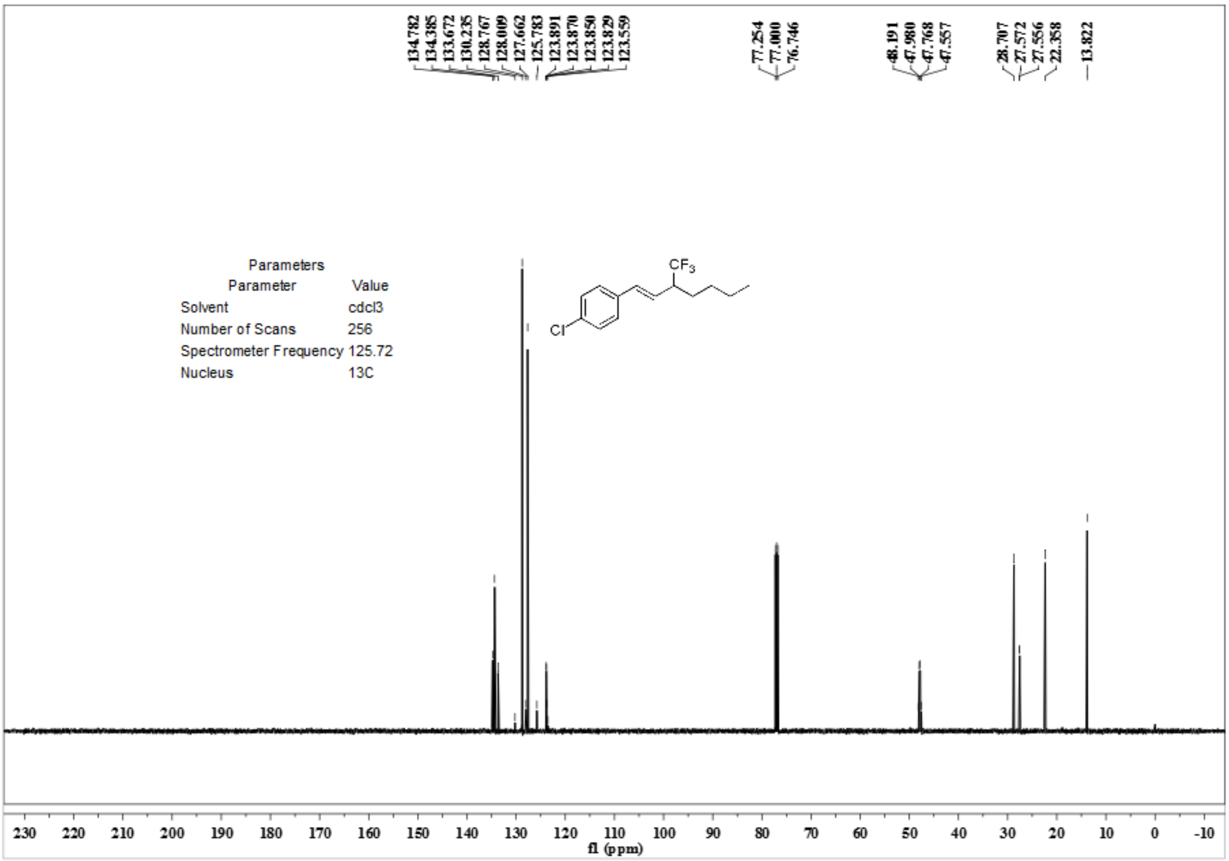

(*E*)-2-(3-(Trifluoromethyl)hept-1-en-1-yl)naphthalene (3k).

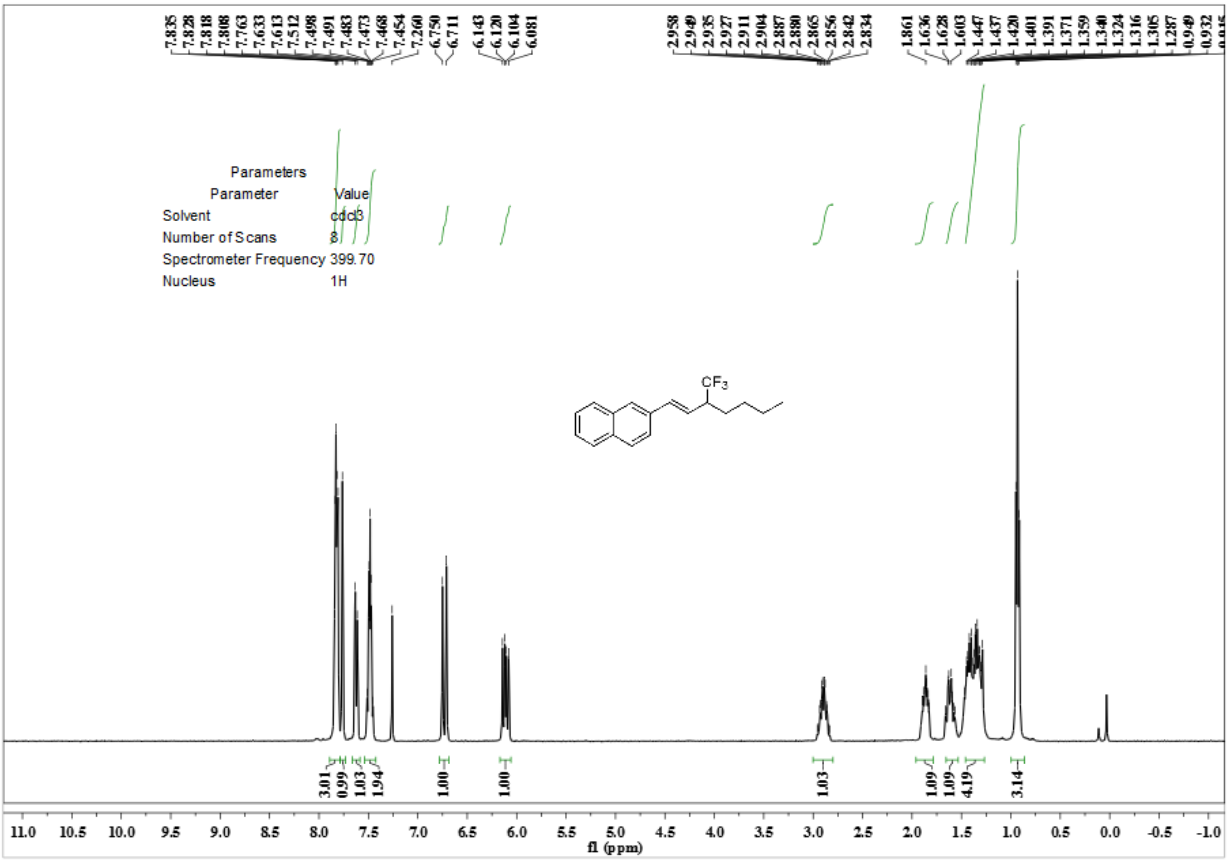

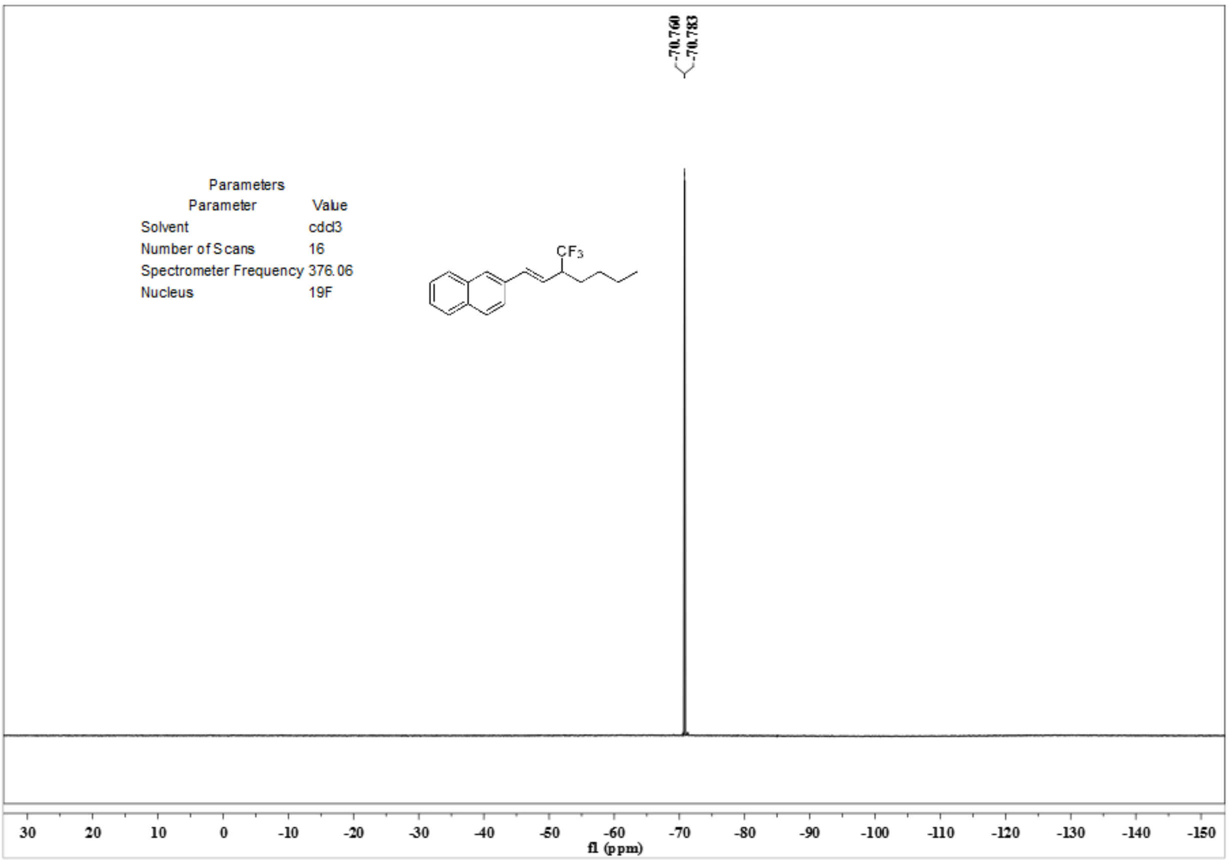

-70.760
-70.783
Parameters
Parameter Value
Solvent cdcl3
Number of Scans 16
Spectrometer Frequency 376.06
Nucleus 19F
CF3
fl (ppm)

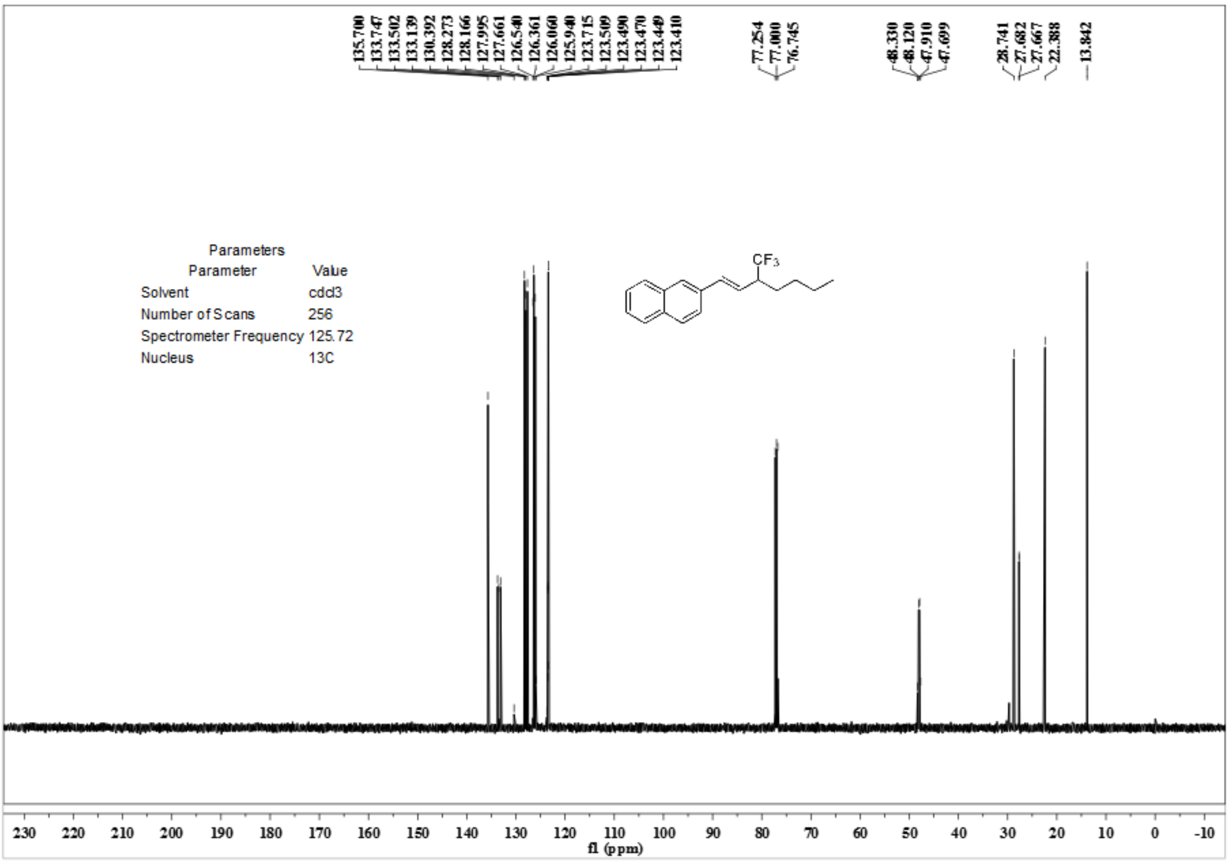

135.700
133.747
133.502
133.139
130.392
128.273
128.166
127.995
127.661
126.540
126.361
126.060
125.940
123.715
123.509
123.490
123.470
123.449
123.410
77.254
77.000
76.745
48.330
48.120
47.910
47.699
28.741
27.682
27.667
22.388
13.842
Parameters
Parameter Value
Solvent cdcl3
Number of Scans 256
Spectrometer Frequency 125.72
Nucleus 13C
CF3
fl (ppm)

((1*E*,3*E*)-5-(Trifluoromethyl)nona-1,3-dien-1-yl)benzene (3l).

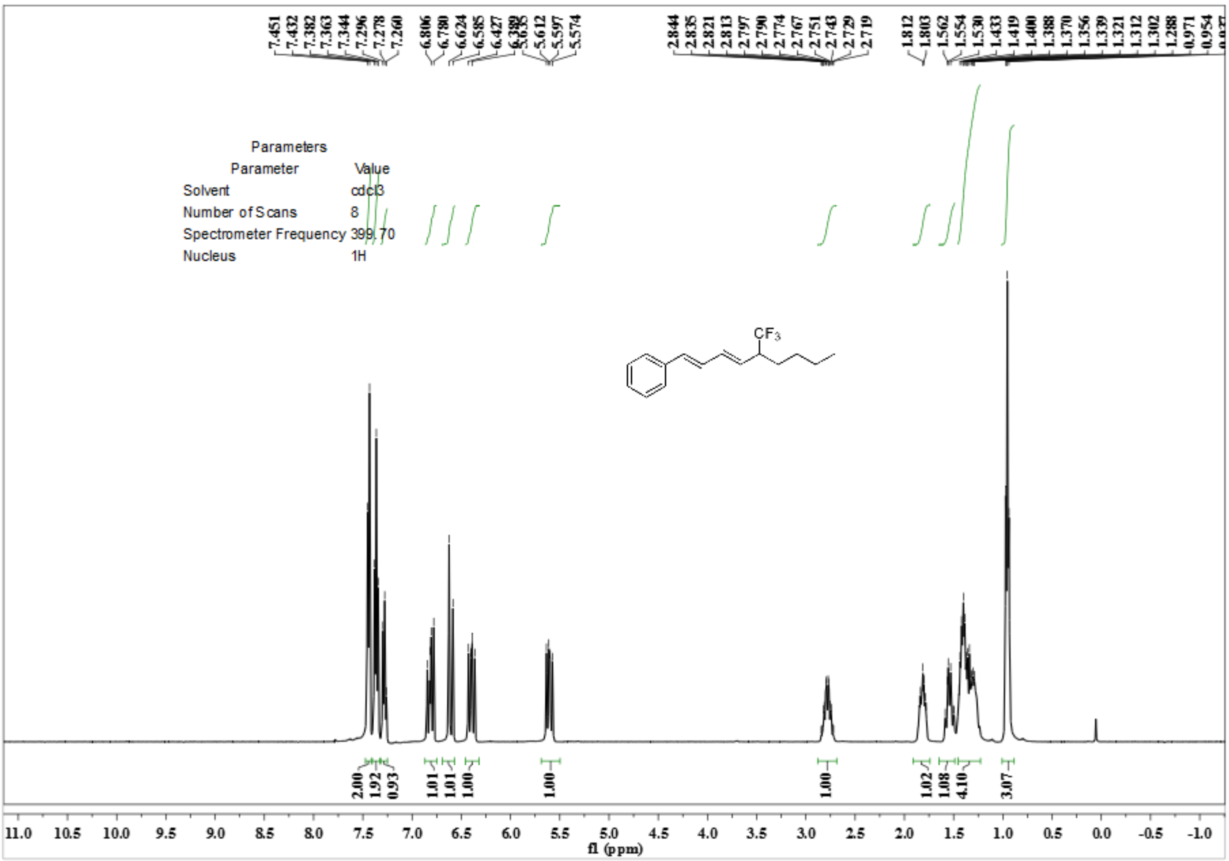

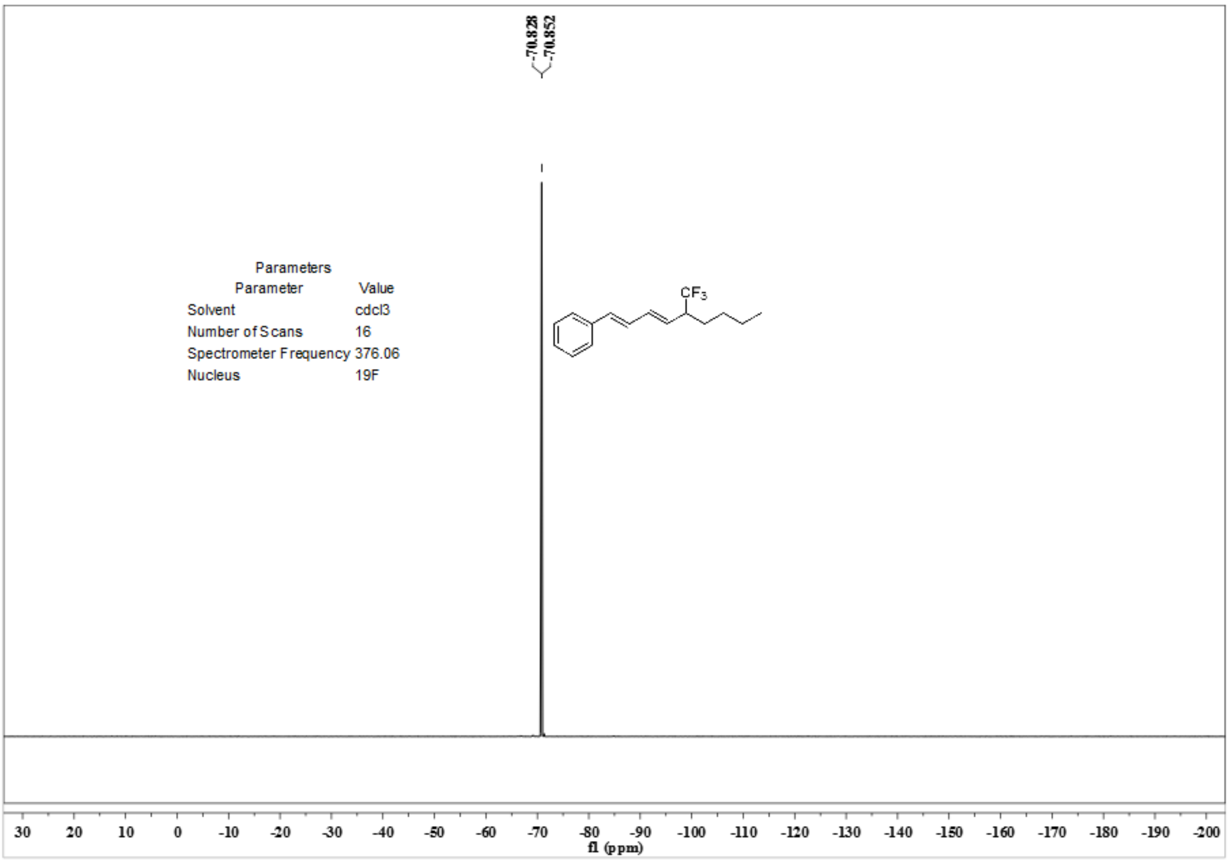

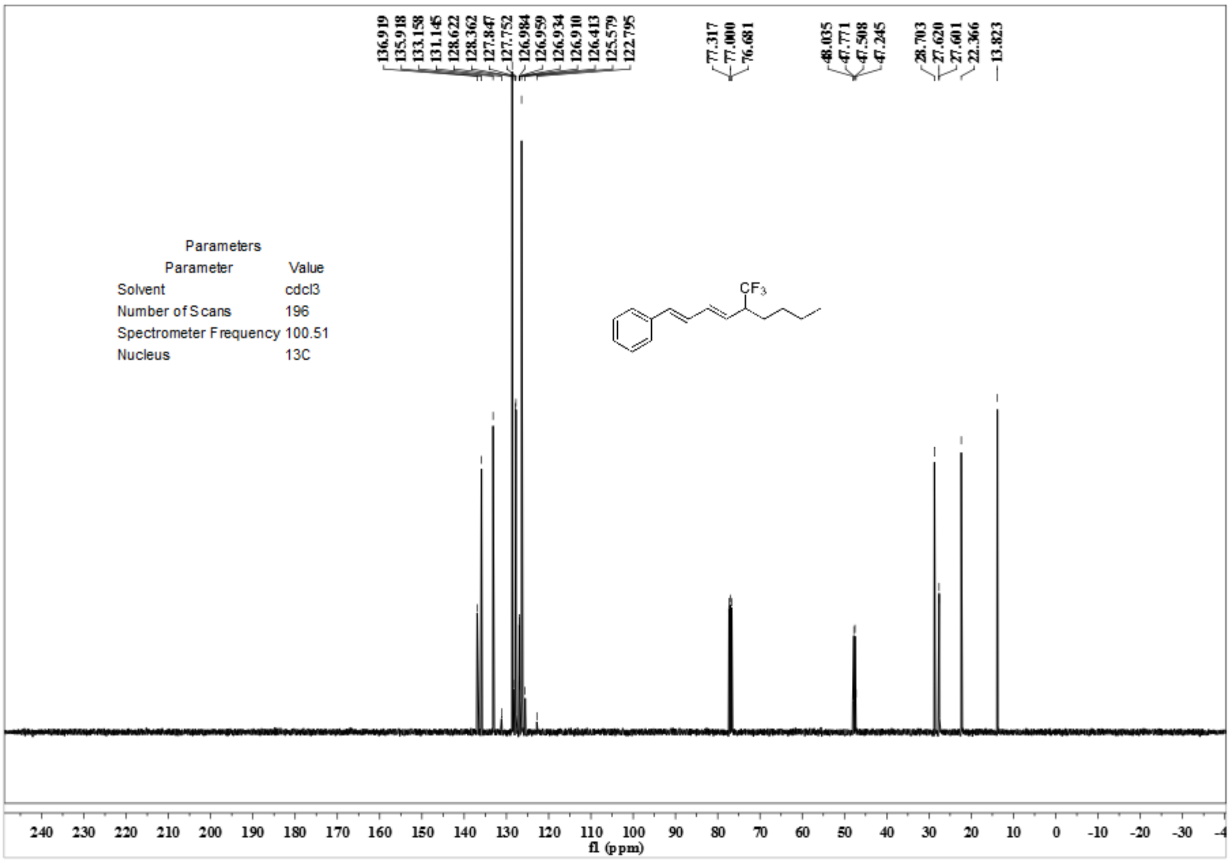

(*E*)-1-(3-(Trifluoromethyl)hept-1-en-1-yl)pyrrolidin-2-one (3m).

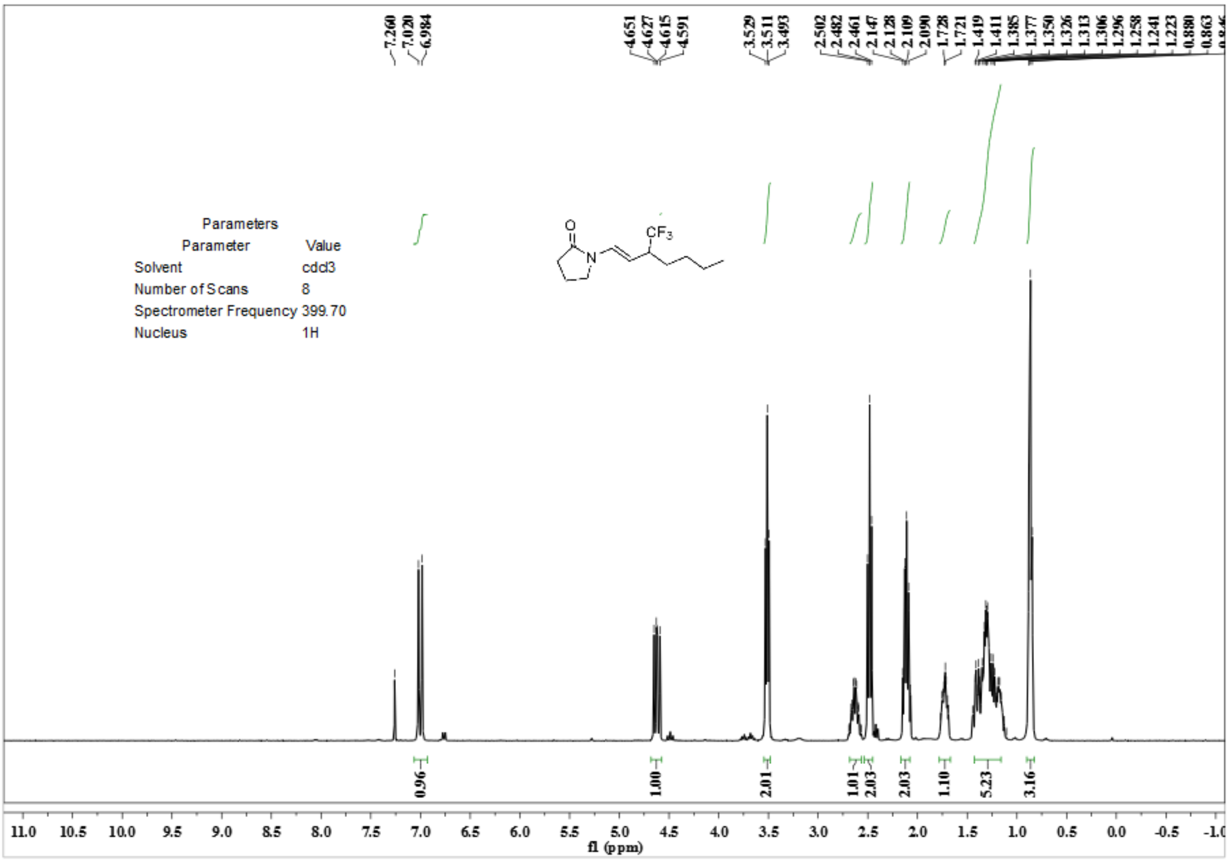

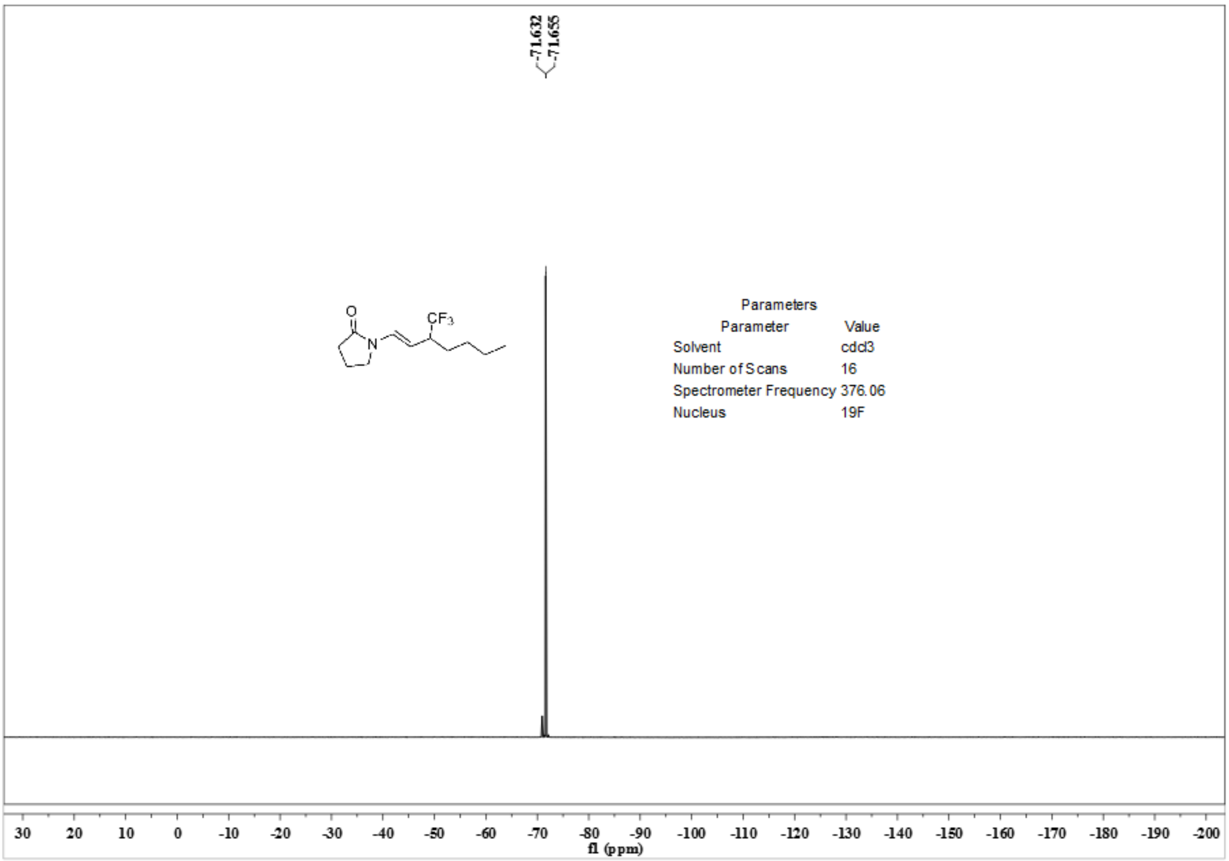
-71.632
-71.655
O
N
CF3
Parameters
Parameter Value
Solvent cdcl3
Number of Scans 16
Spectrometer Frequency 376.06
Nucleus 19F
fl (ppm)

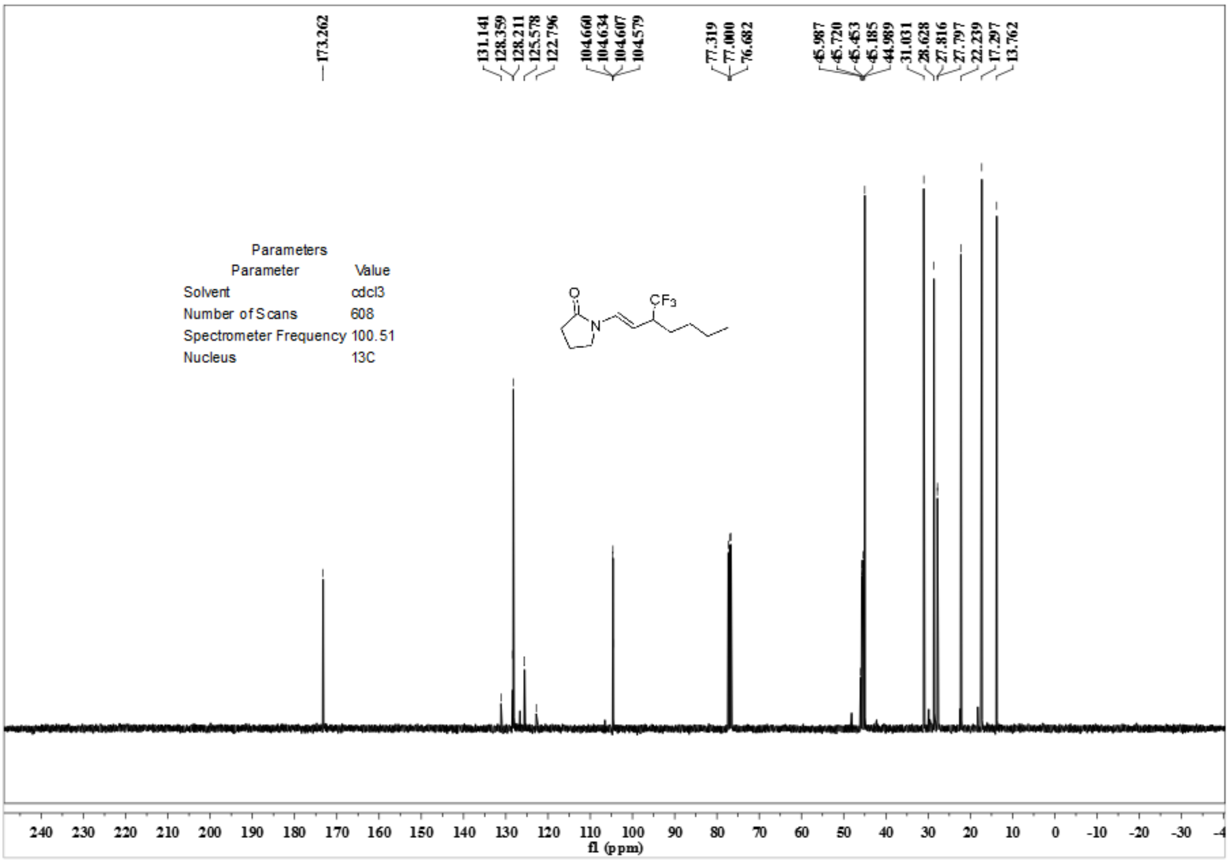
173.262
131.141
128.359
128.211
125.578
122.796
104.660
104.634
104.607
104.579
77.319
77.000
76.682
45.987
45.720
45.453
45.185
44.989
31.031
28.628
27.816
27.797
22.239
17.297
13.762
Parameters
Parameter Value
Solvent cdcl3
Number of Scans 608
Spectrometer Frequency 100.51
Nucleus 13C
O
N
CF3
fl (ppm)

(*E*)-4-(5-phenyl-3-(trifluoromethyl)pent-1-en-1-yl)phenyl acetate (4a).

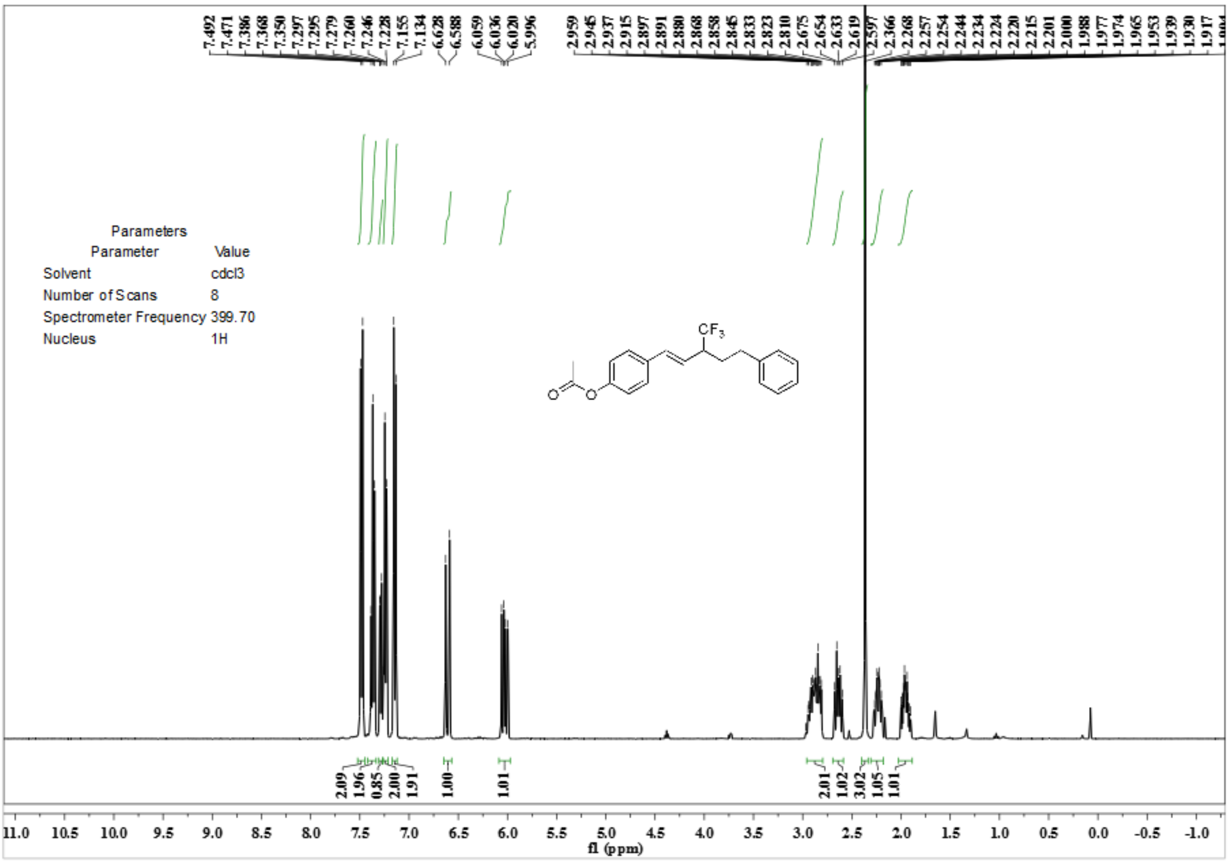

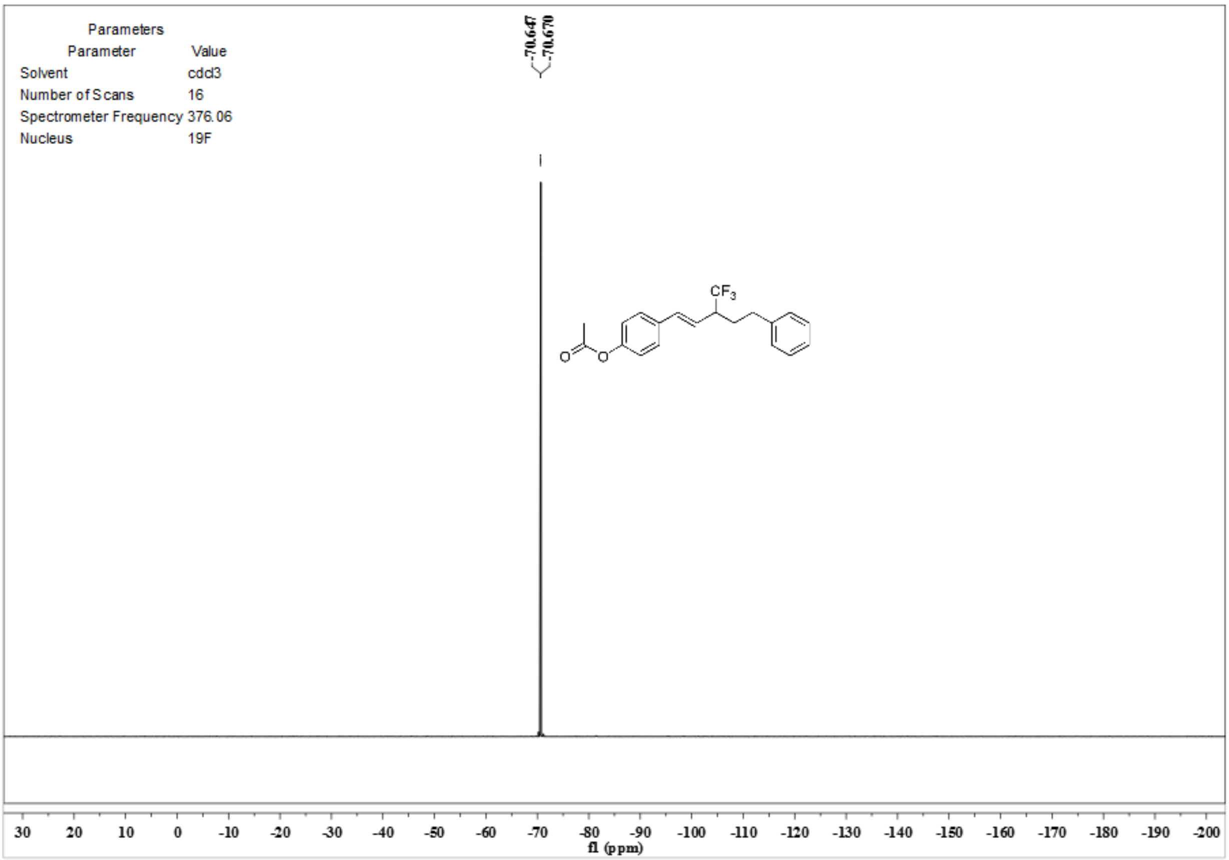

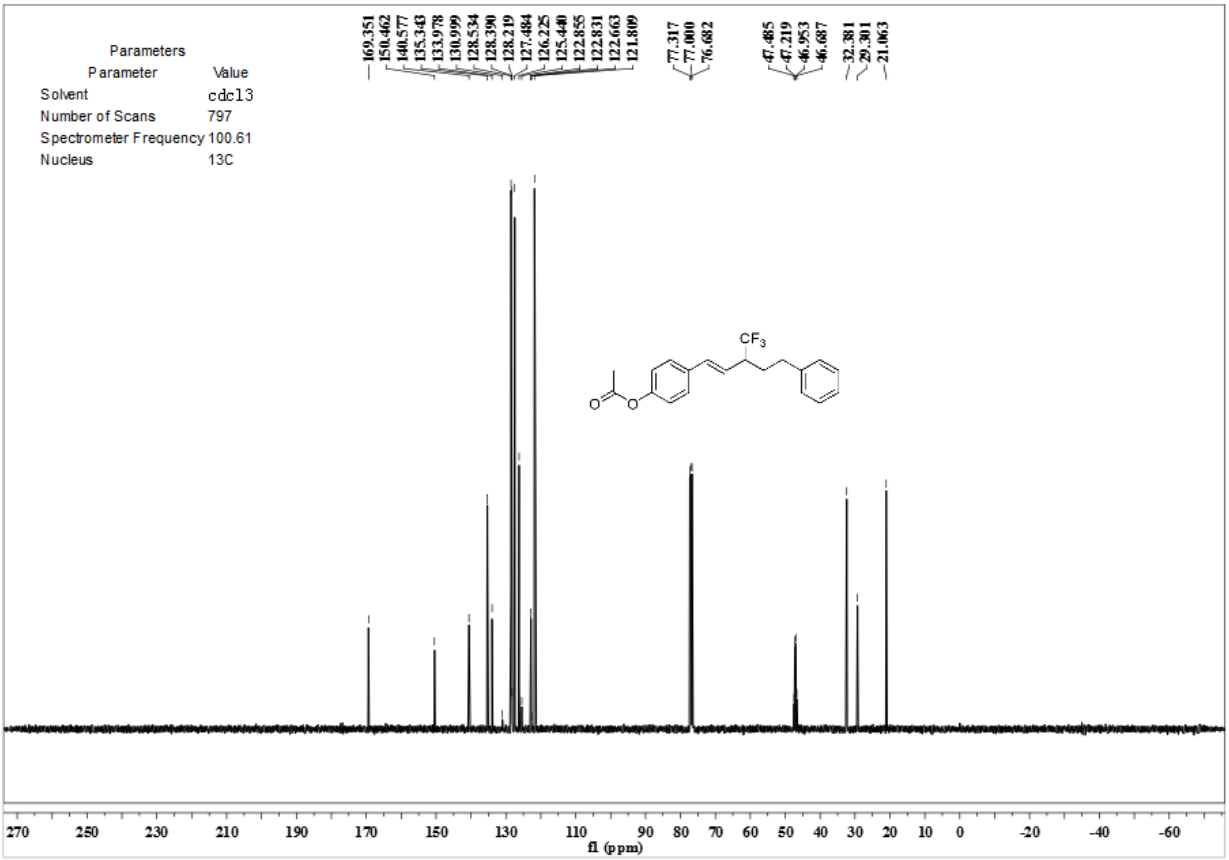

(*E*)-1-Chloro-4-(5-phenyl-3-(trifluoromethyl)pent-1-en-1-yl)benzene (4b).

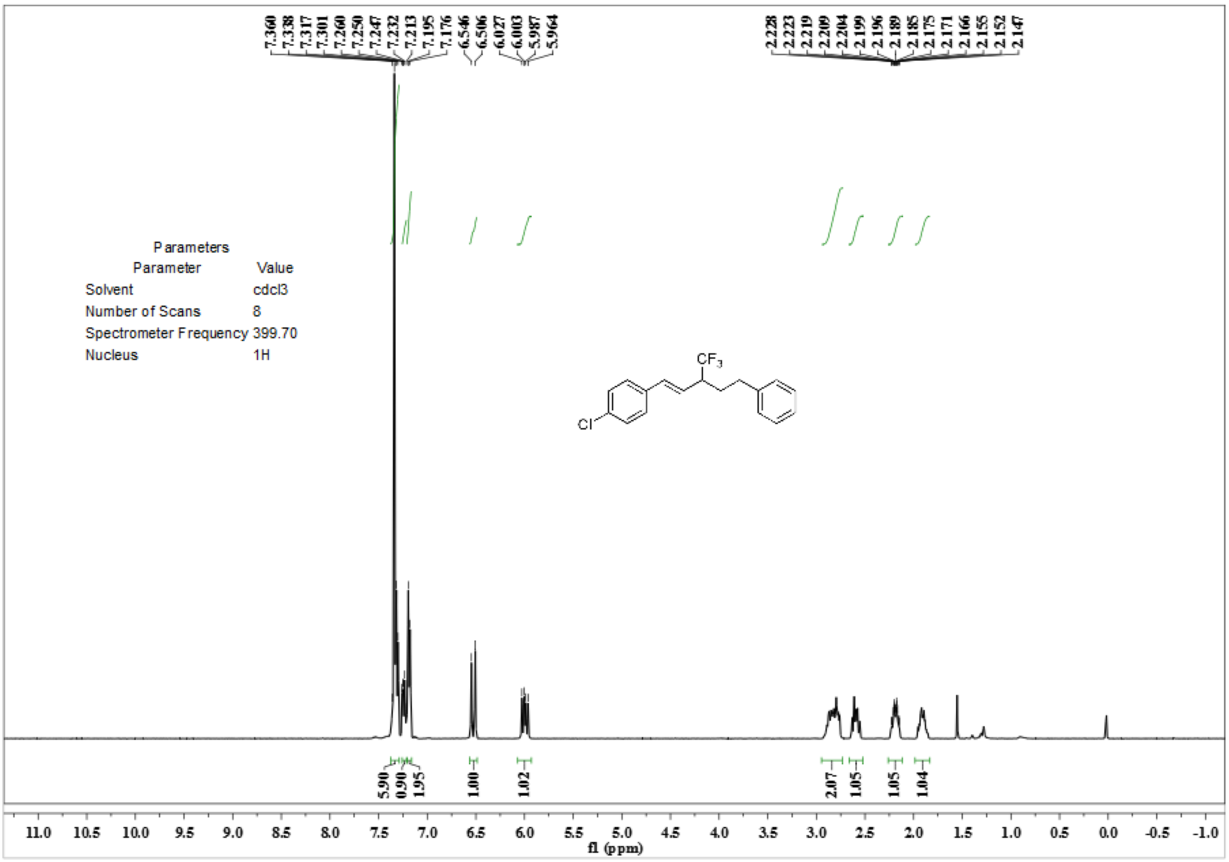

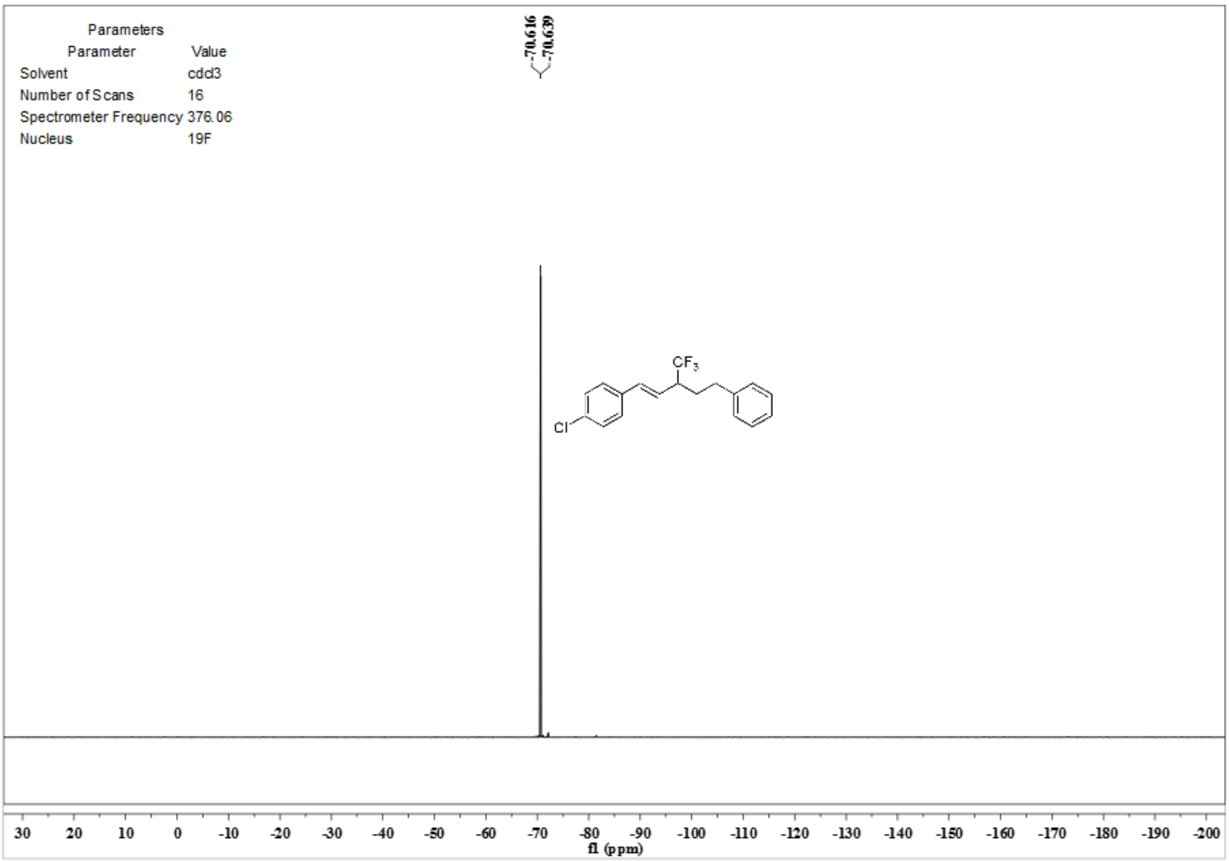
Parameters
Parameter Value
Solvent cdcl3
Number of Scans 16
Spectrometer Frequency 376.06
Nucleus 19F
-70.616
-70.639
CF3
Cl
fl (ppm)

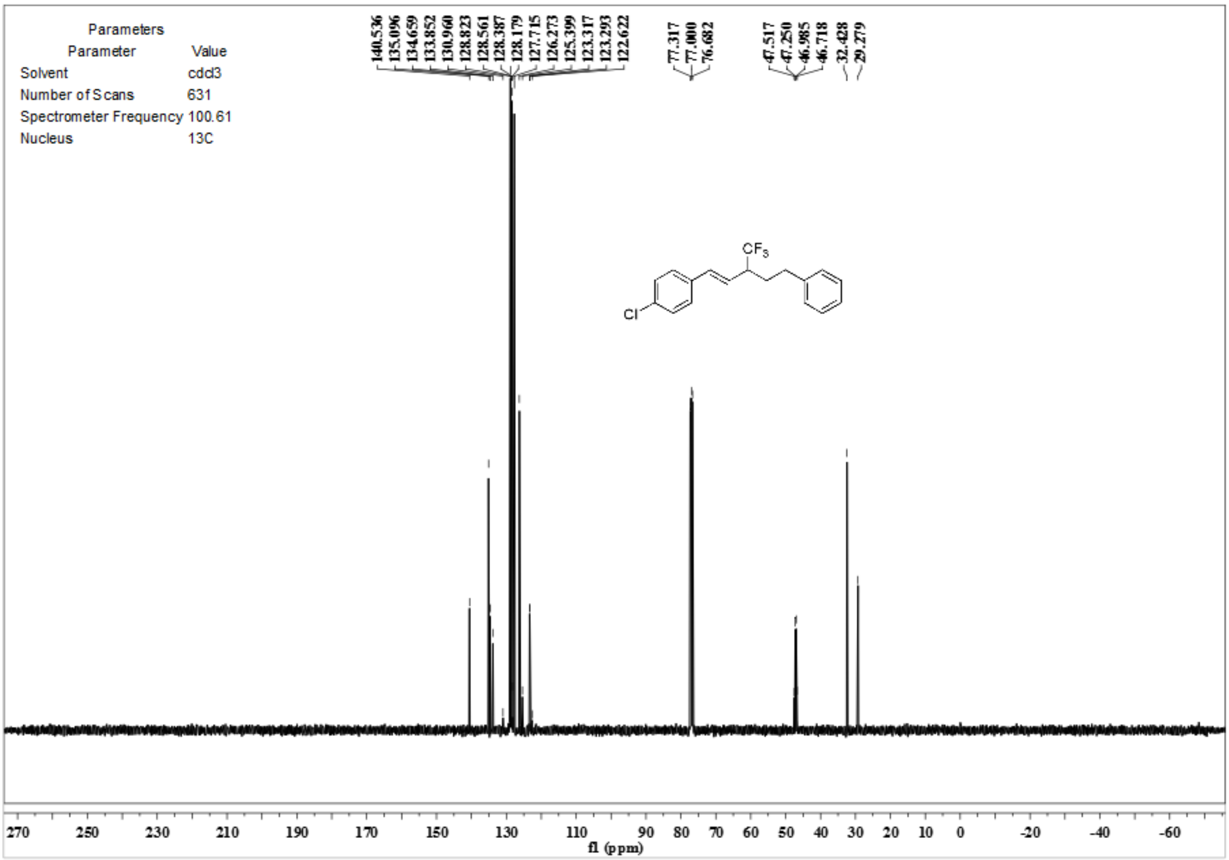
Parameters
Parameter Value
Solvent cdcl3
Number of Scans 631
Spectrometer Frequency 100.61
Nucleus 13C
140.536
135.096
134.659
133.852
130.960
128.823
128.561
128.387
128.179
127.715
126.273
125.399
123.317
123.293
122.622
77.317
77.000
76.682
47.517
47.250
46.985
46.718
32.428
29.279
CF3
Cl
fl (ppm)

(*E*)-2-(5-Phenyl-3-(trifluoromethyl)pent-1-en-1-yl)naphthalene (4c).

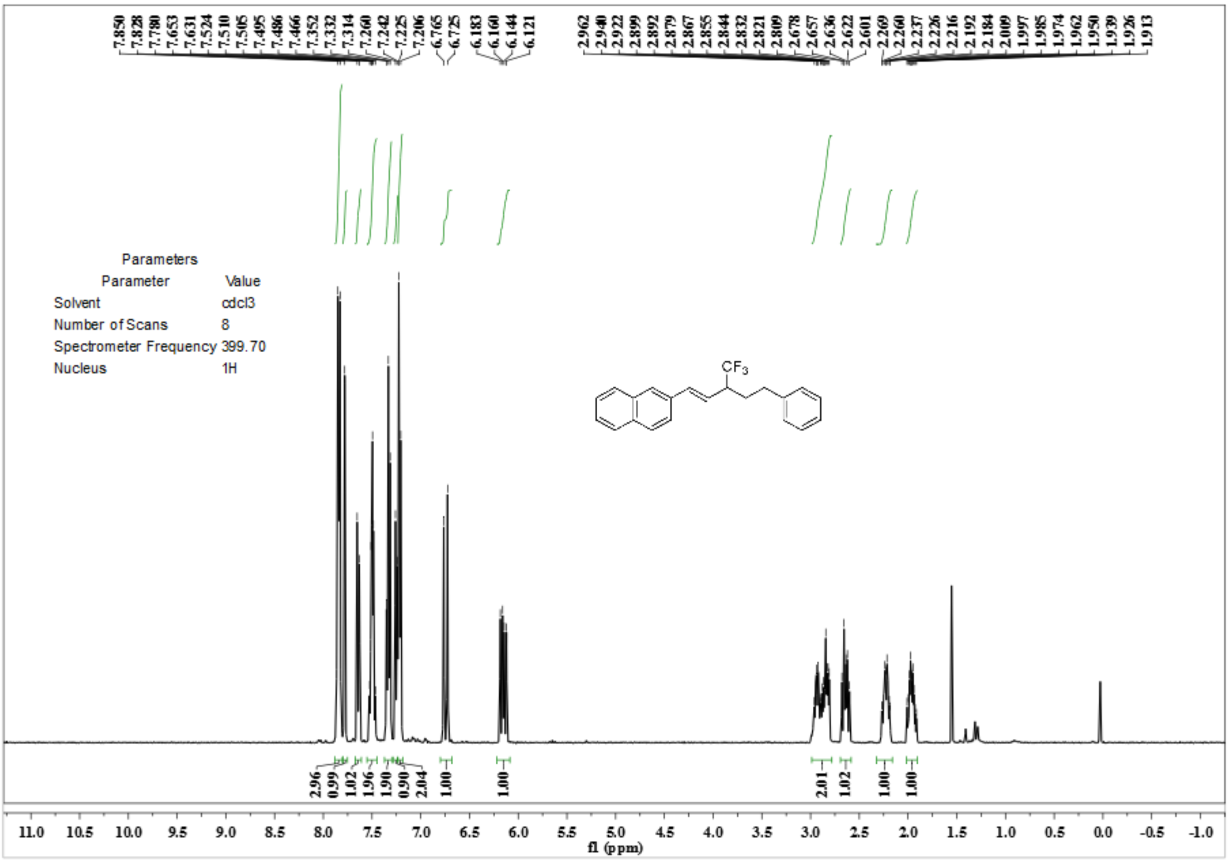

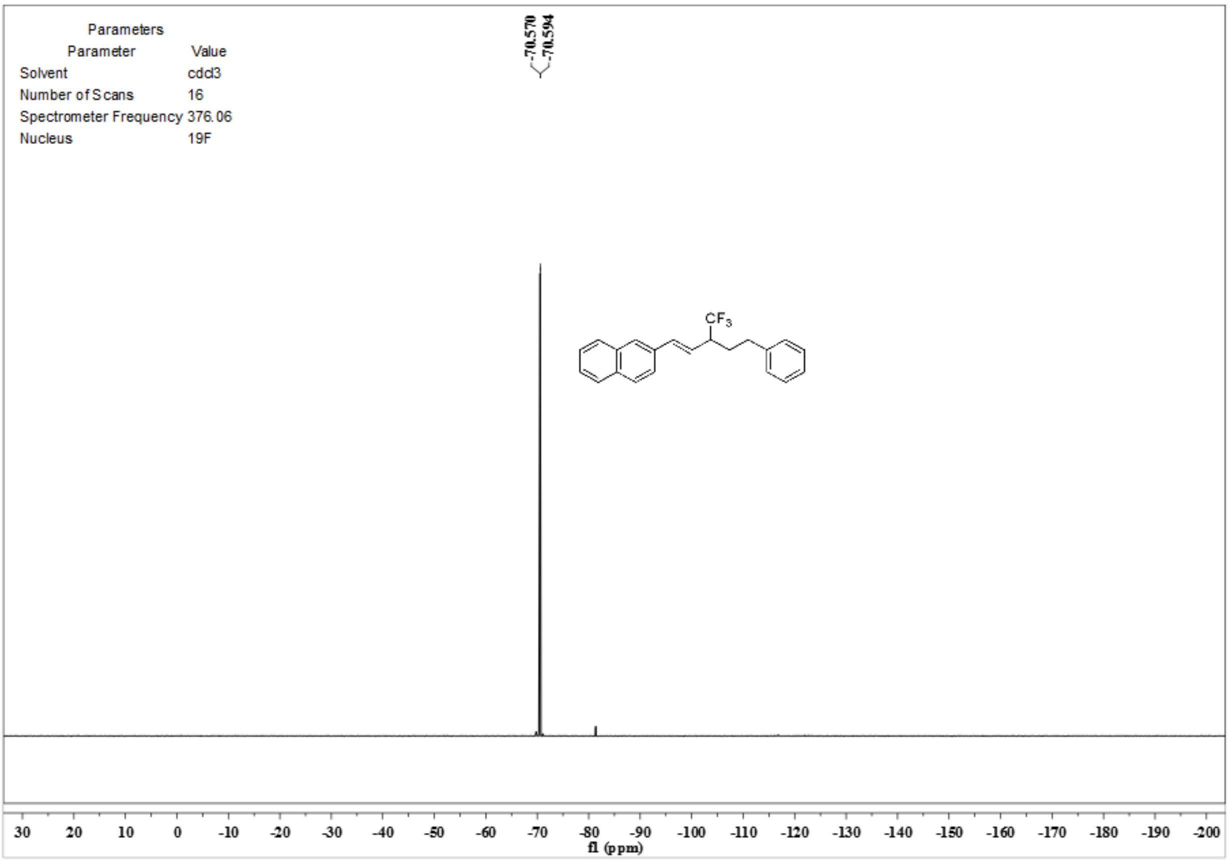

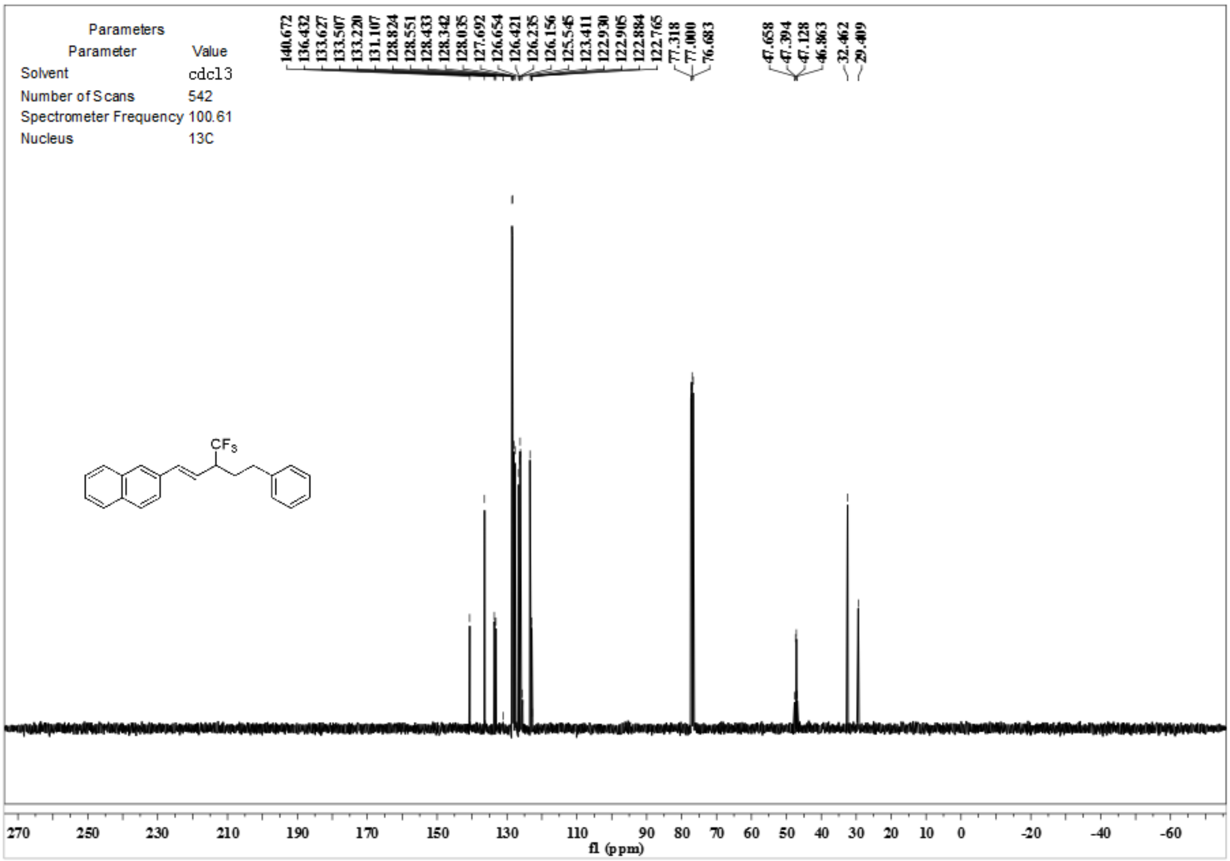

((1*E*,3*E*)-5-(trifluoromethyl)hepta-1,3-diene-1,7-diyl)dibenzene (4d).

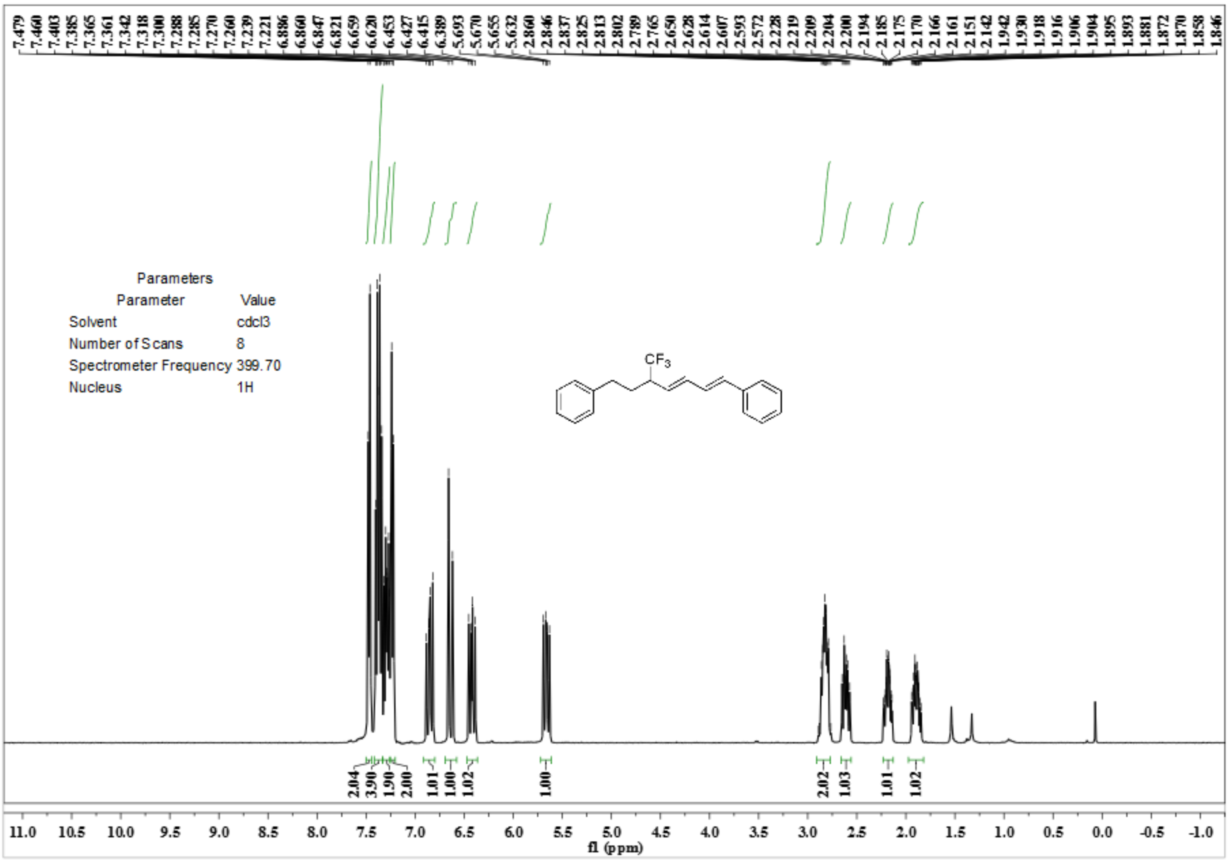

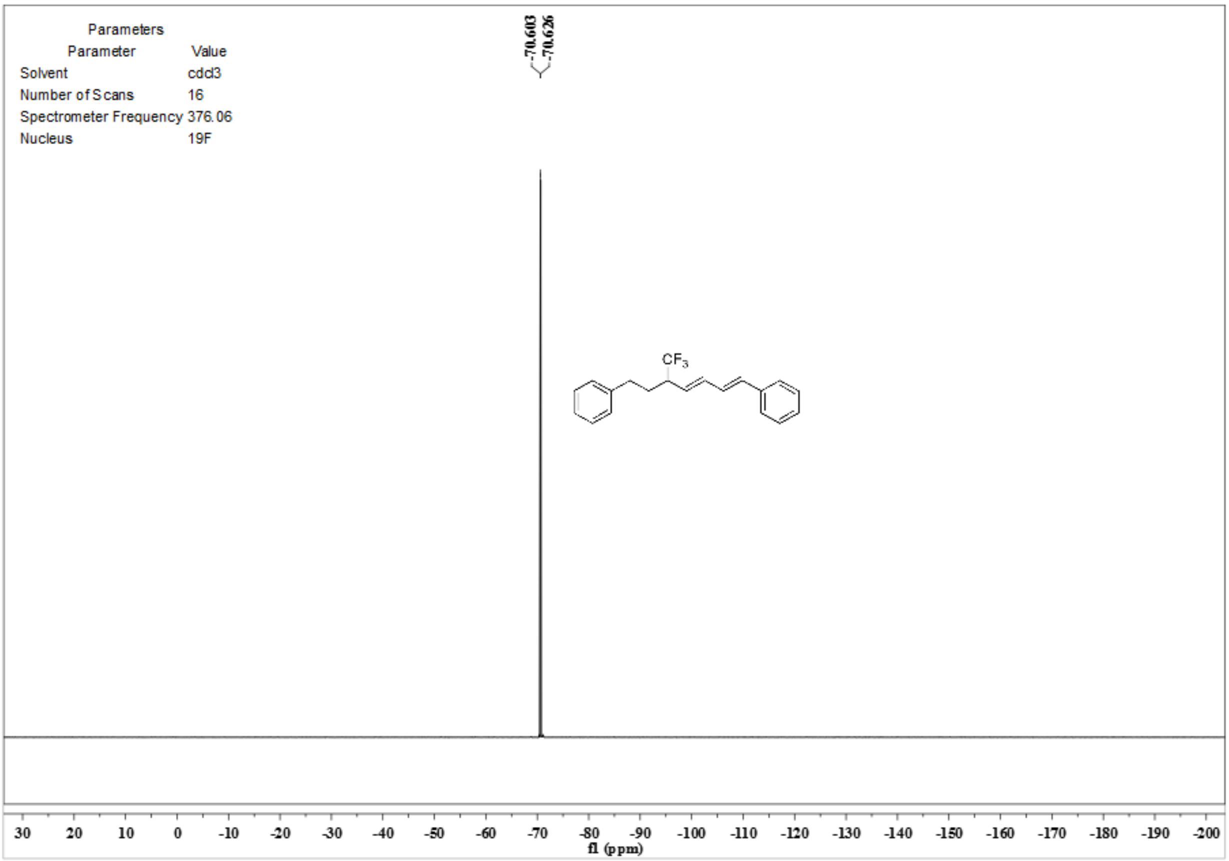
Parameters
Parameter Value
Solvent cdcl3
Number of Scans 16
Spectrometer Frequency 376.06
Nucleus 19F
-70.603
-70.626
CF3
30 20 10 0 -10 -20 -30 -40 -50 -60 -70 -80 -90 -100 -110 -120 -130 -140 -150 -160 -170 -180 -190 -200
f1 (ppm)

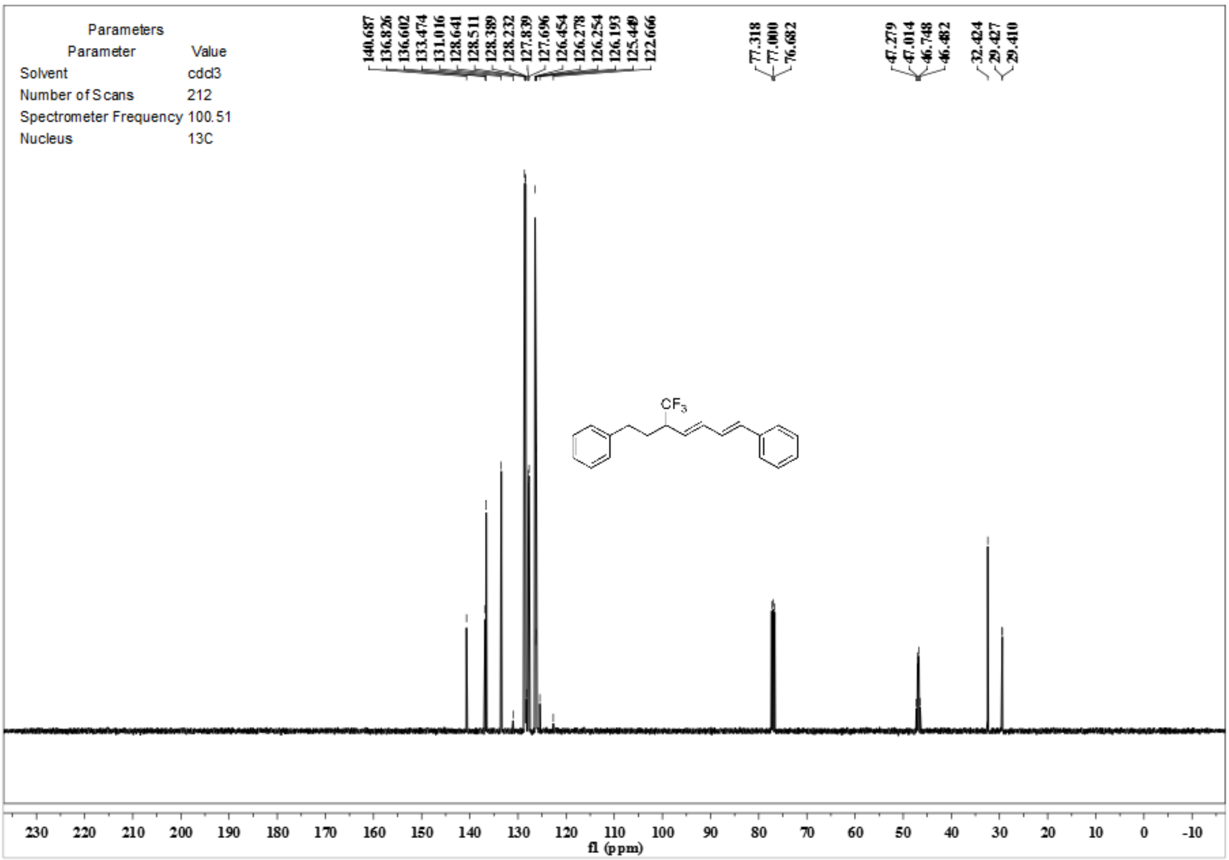
Parameters
Parameter Value
Solvent cdcl3
Number of Scans 212
Spectrometer Frequency 100.51
Nucleus 13C
140.687
136.826
136.602
133.474
131.016
128.641
128.511
128.389
128.232
127.839
127.696
126.454
126.278
126.254
126.193
125.449
122.666
77.318
77.000
76.682
47.279
47.014
46.748
46.482
32.424
29.427
29.410
CF3
230 220 210 200 190 180 170 160 150 140 130 120 110 100 90 80 70 60 50 40 30 20 10 0 -10
f1 (ppm)

(*E*)-4-(6-Hydroxy-3-(trifluoromethyl)hex-1-en-1-yl)phenyl acetate (4e).

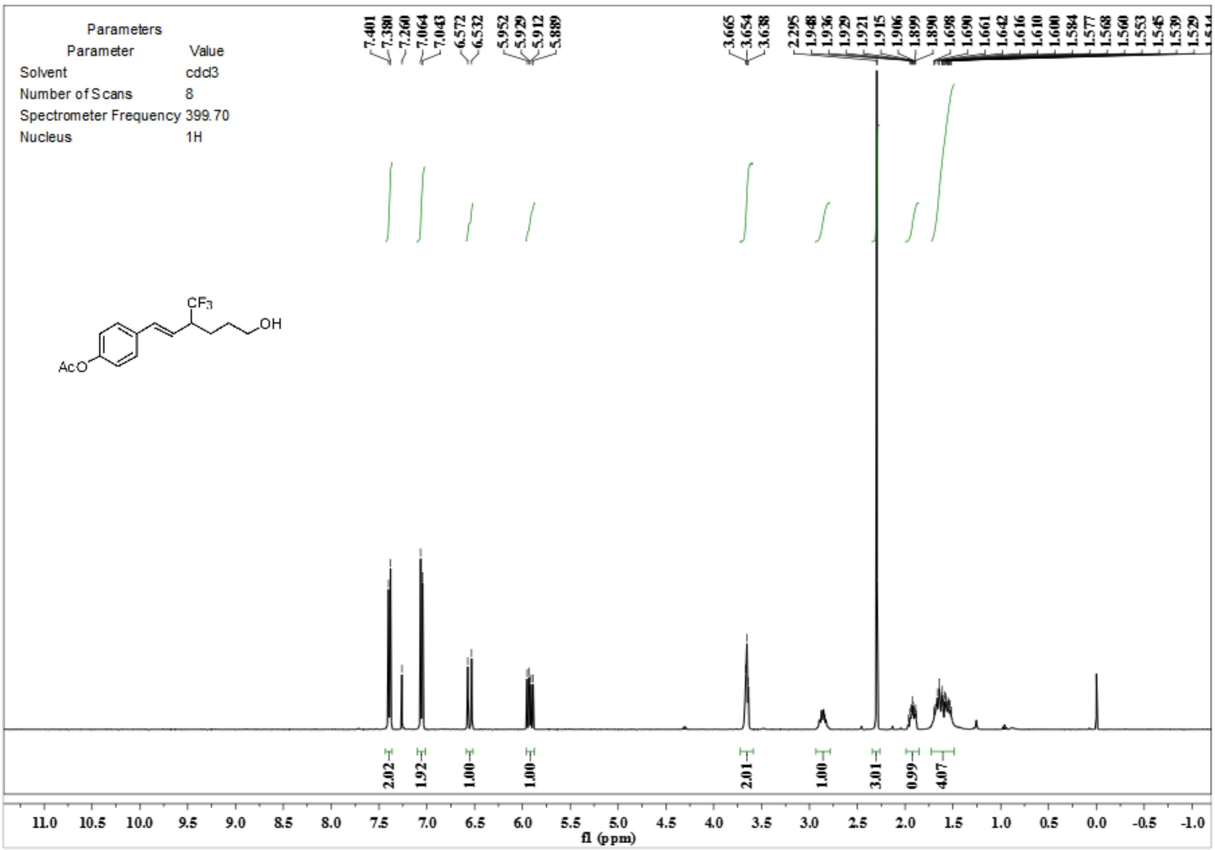

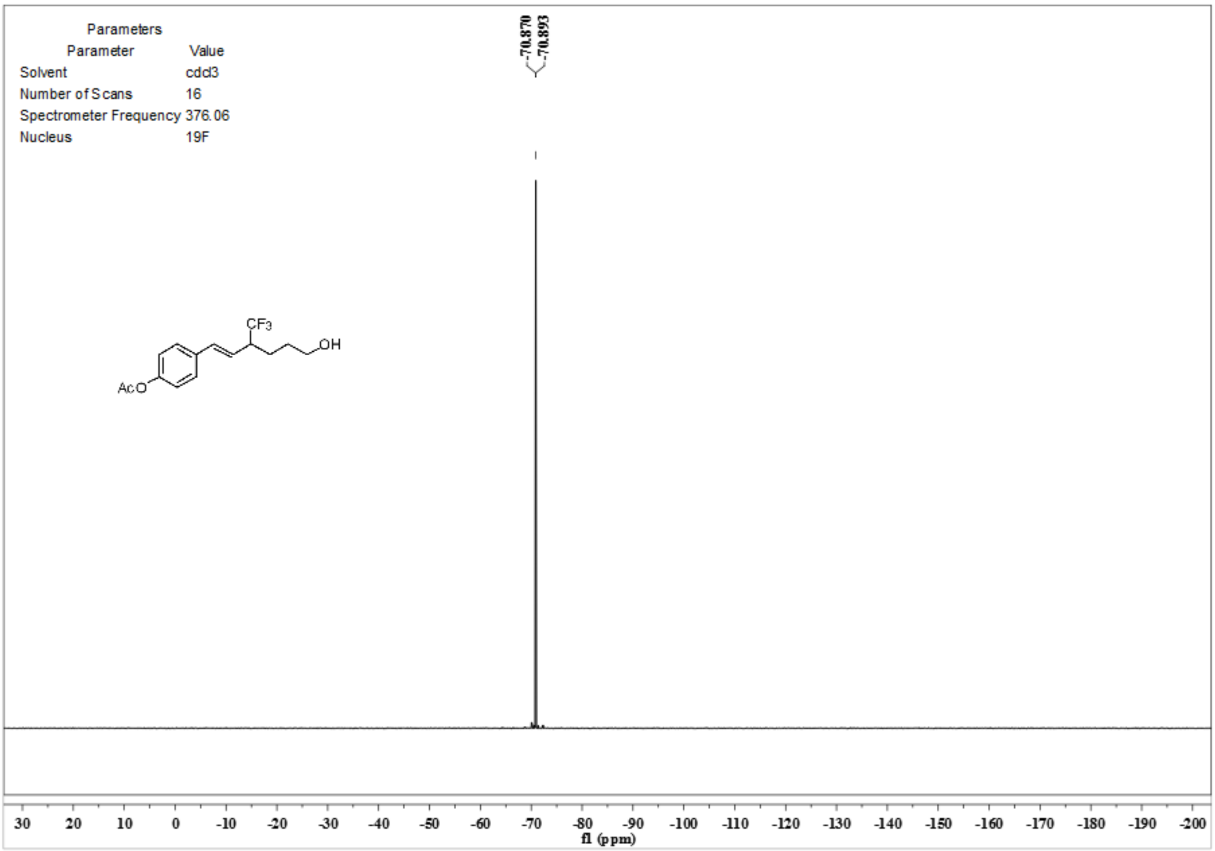

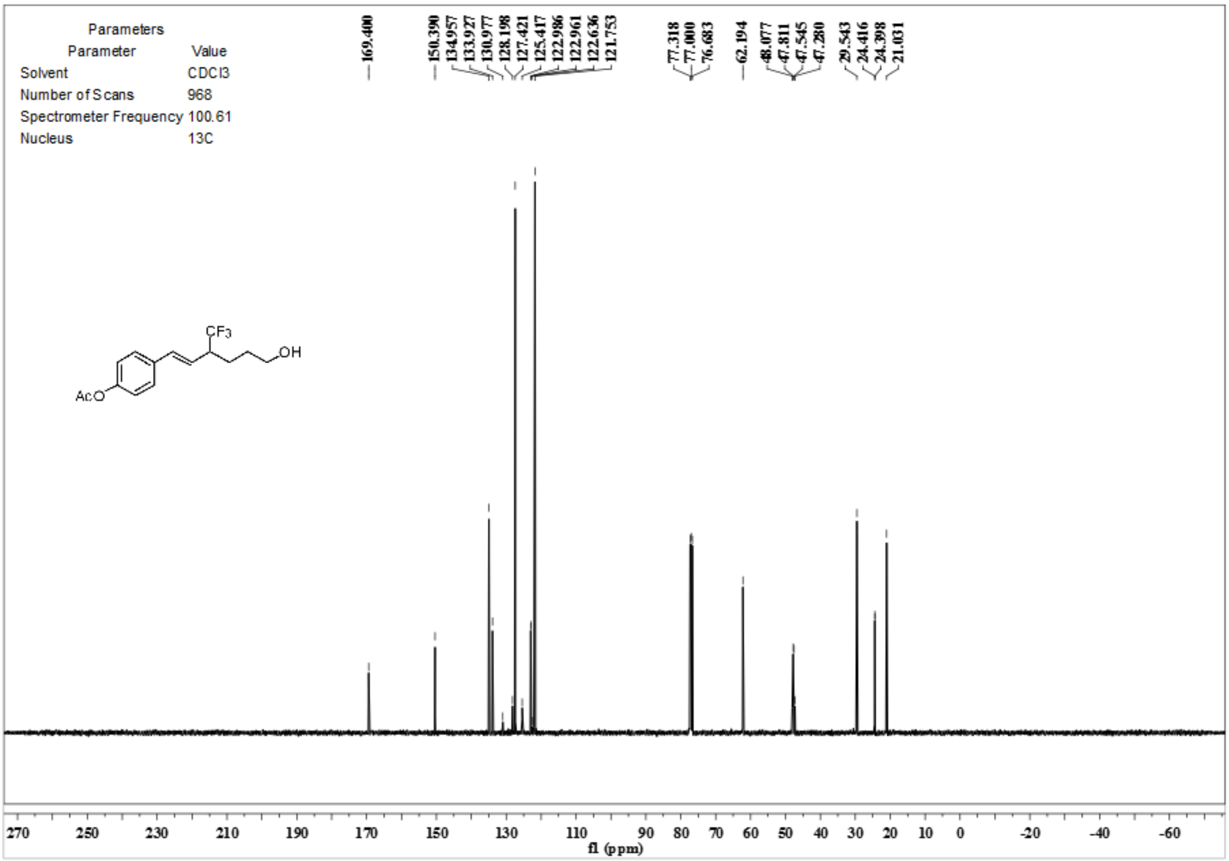

(*E*)-4-(6-hydroxy-3-(trifluoromethyl)hex-1-en-1-yl)benzonitrile (4f).

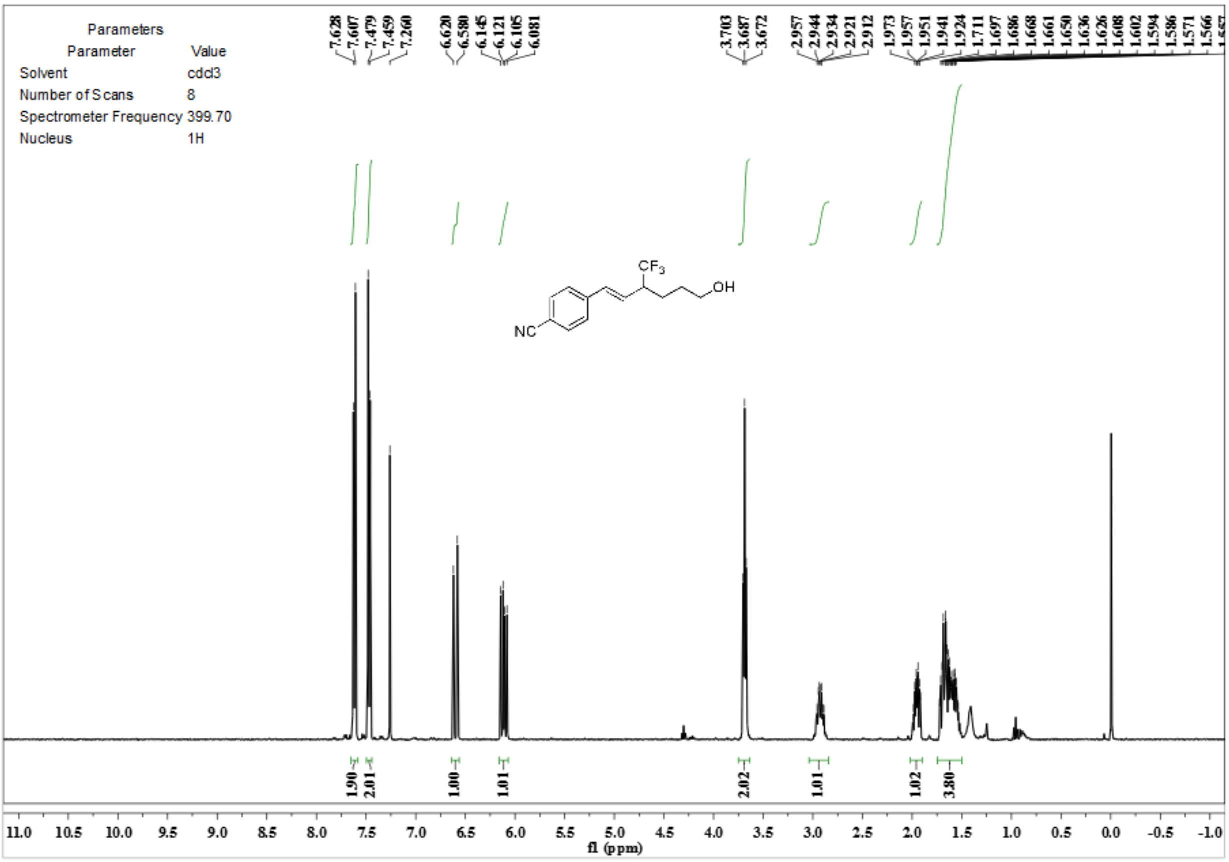

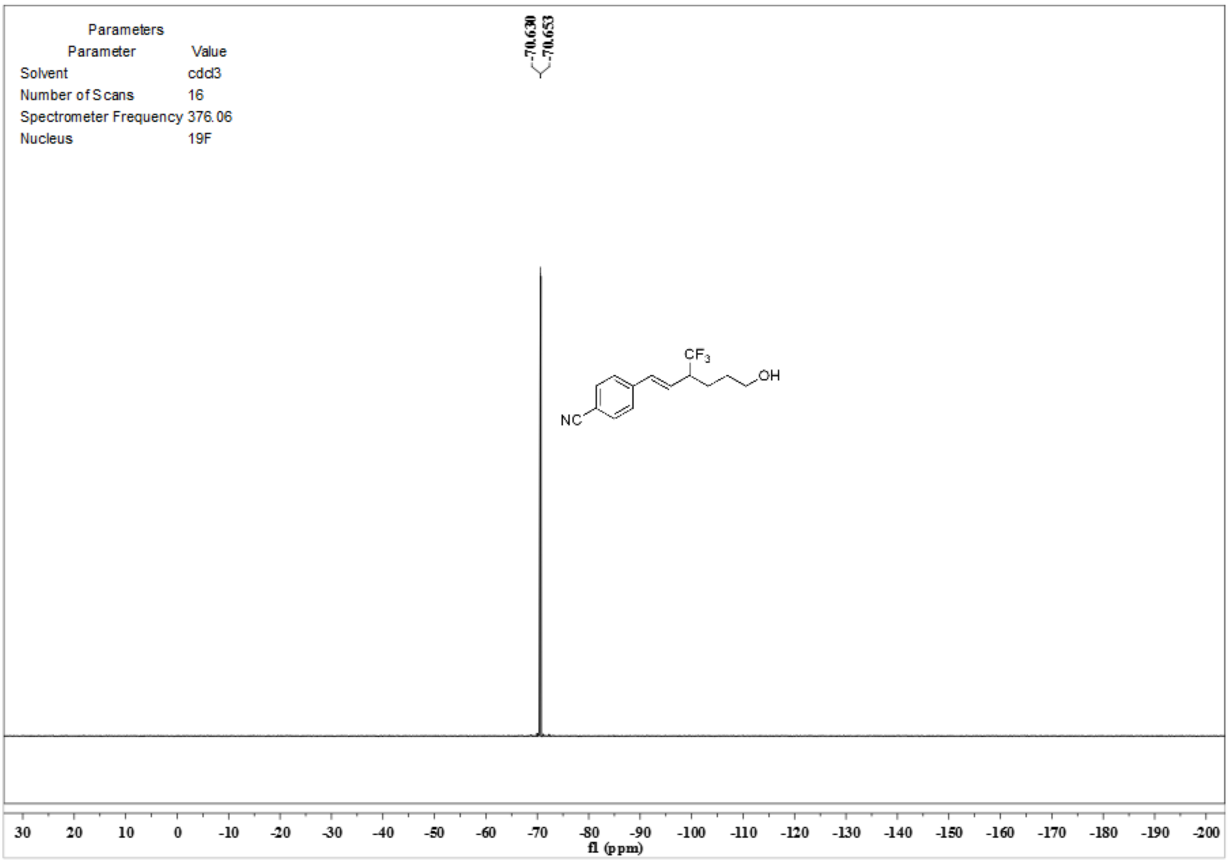
Parameters
Parameter Value
Solvent cdcl3
Number of Scans 16
Spectrometer Frequency 376.06
Nucleus 19F
-70.630
-70.653
CF3
OH
NC
f1 (ppm)

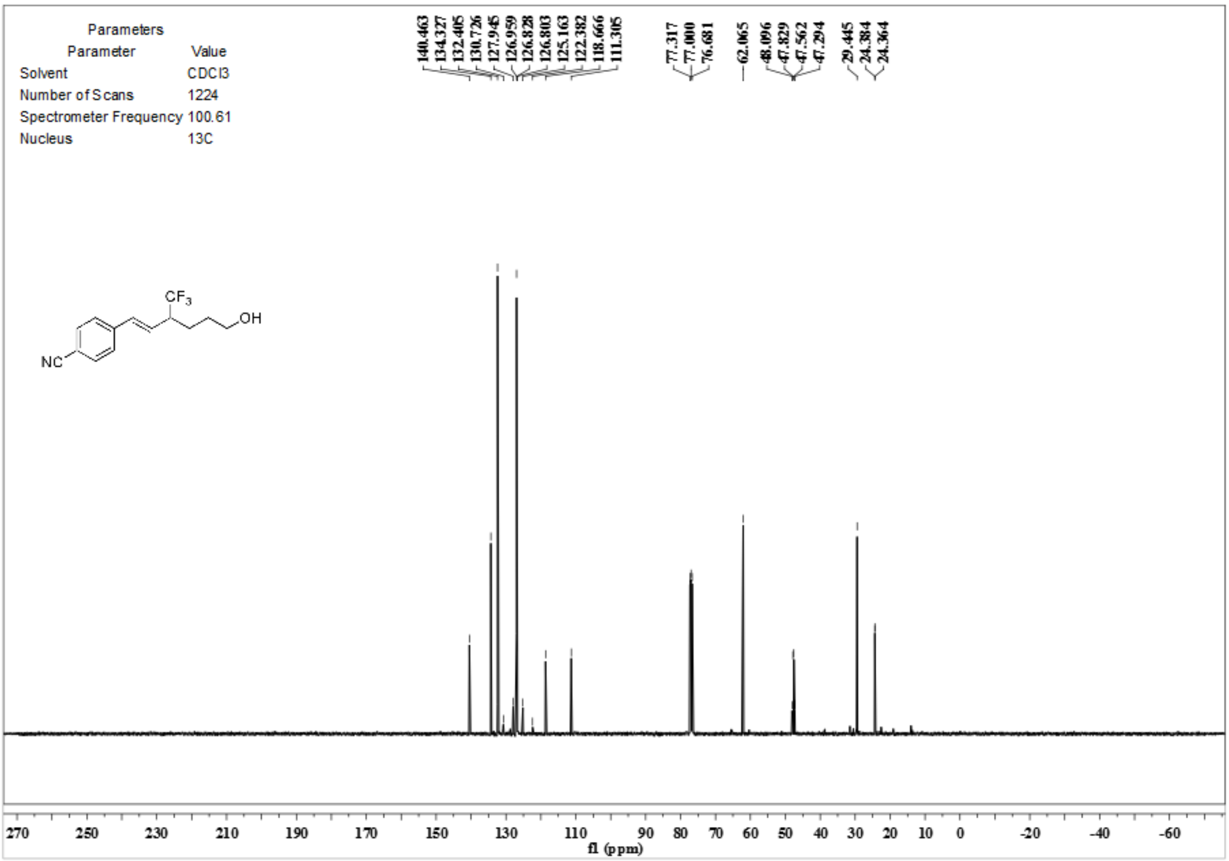
Parameters
Parameter Value
Solvent CDCl3
Number of Scans 1224
Spectrometer Frequency 100.61
Nucleus 13C
140.463
134.327
132.405
130.726
127.945
126.959
126.828
126.803
125.163
122.382
118.666
111.305
77.317
77.000
76.681
62.065
48.096
47.829
47.562
47.294
29.445
24.384
24.364
CF3
OH
NC
f1 (ppm)

(*E*)-6-(4-Methoxyphenyl)-4-(trifluoromethyl)hex-5-en-1-yl benzoate (4g).

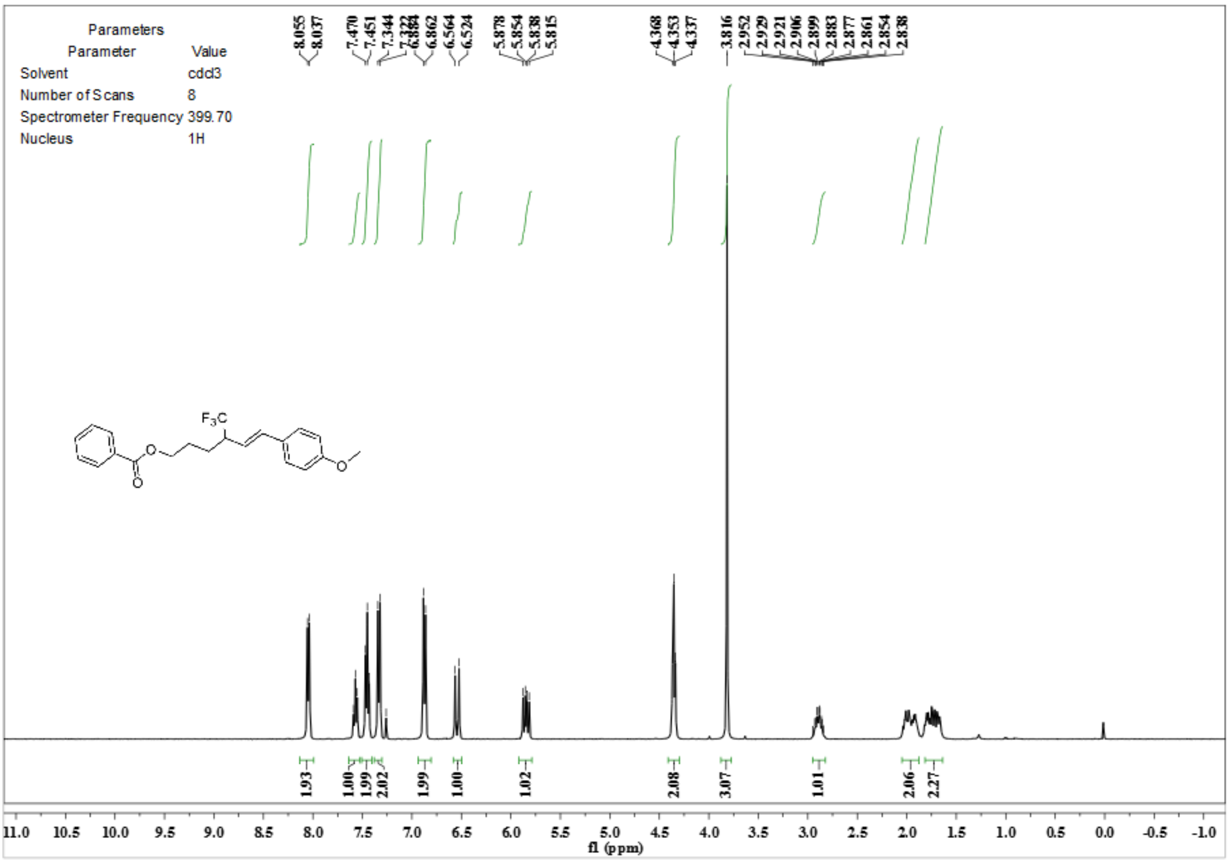

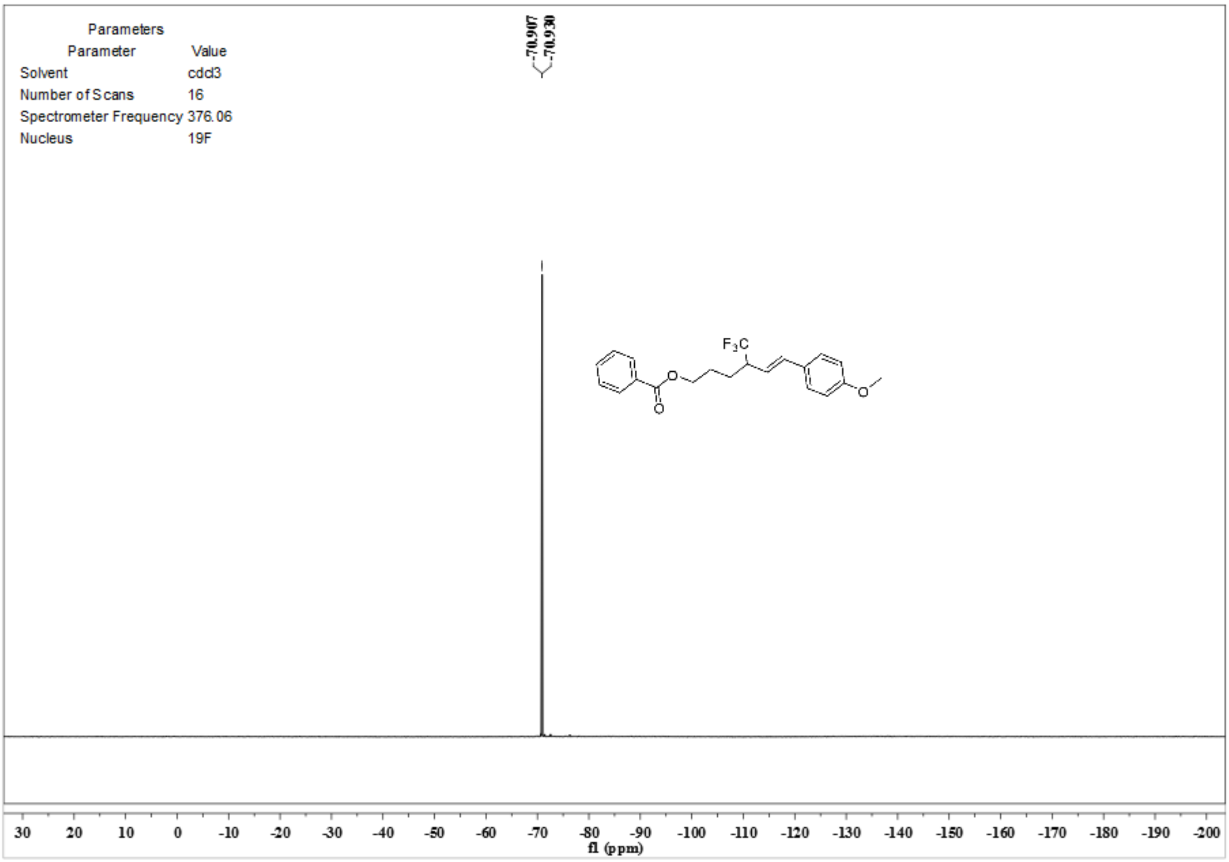

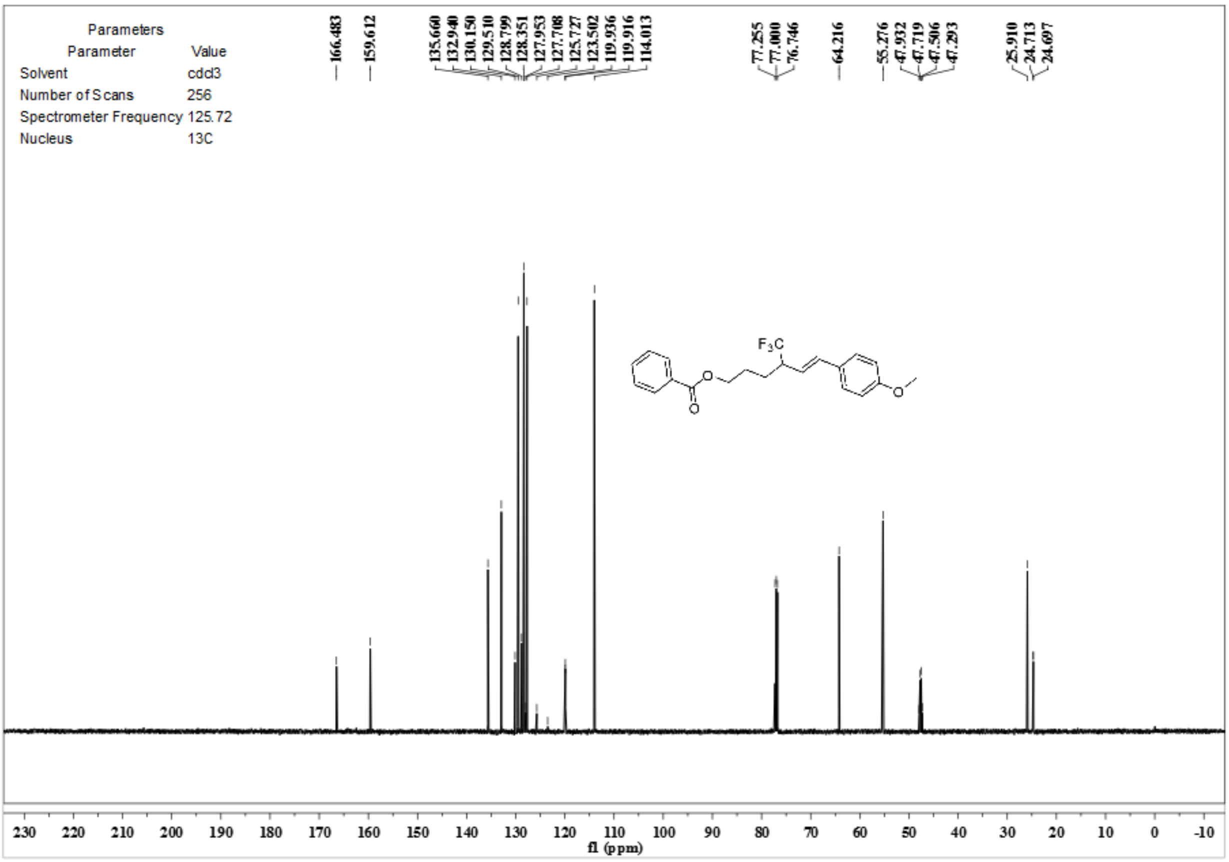

(5*E*,7*E*)-8-Phenyl-4-(trifluoromethyl)octa-5,7-dien-1-yl benzoate (4h).

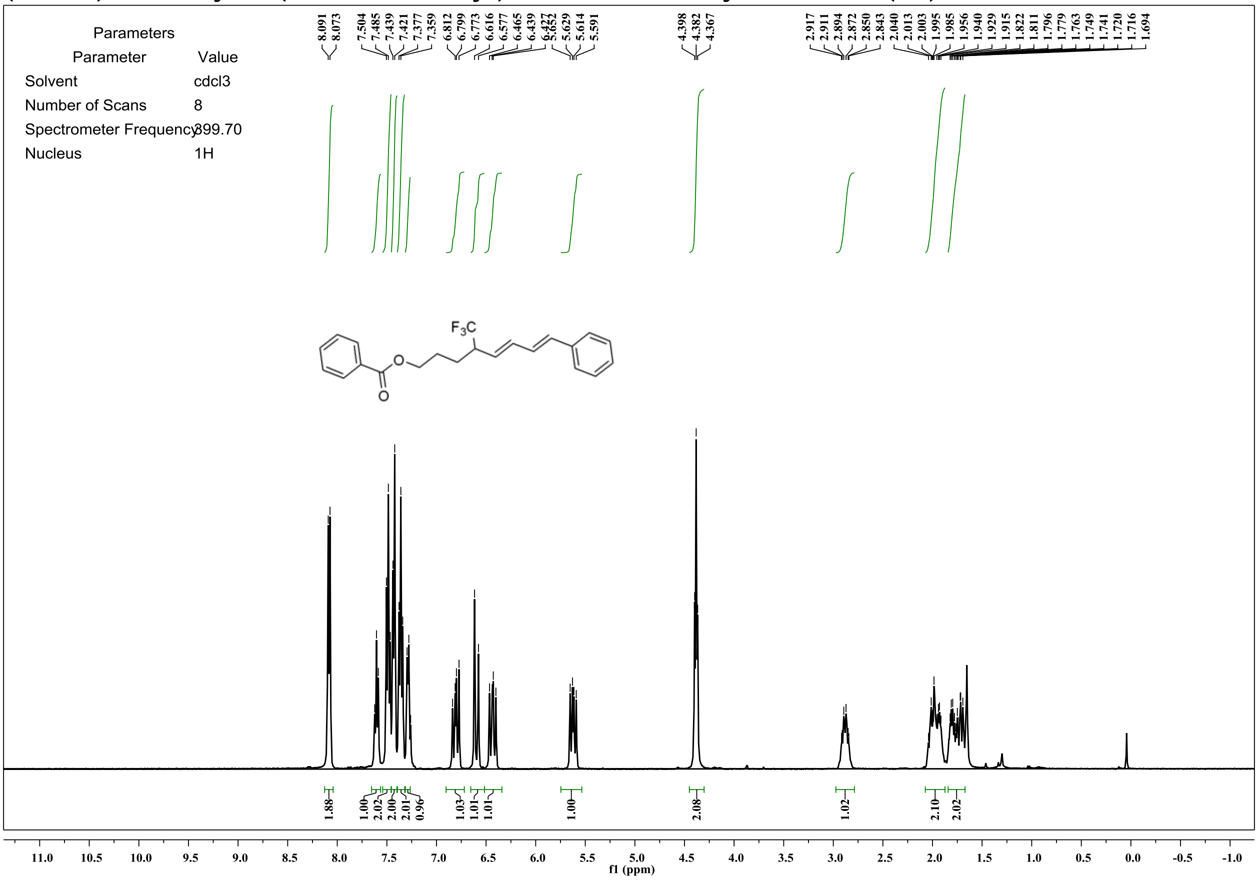

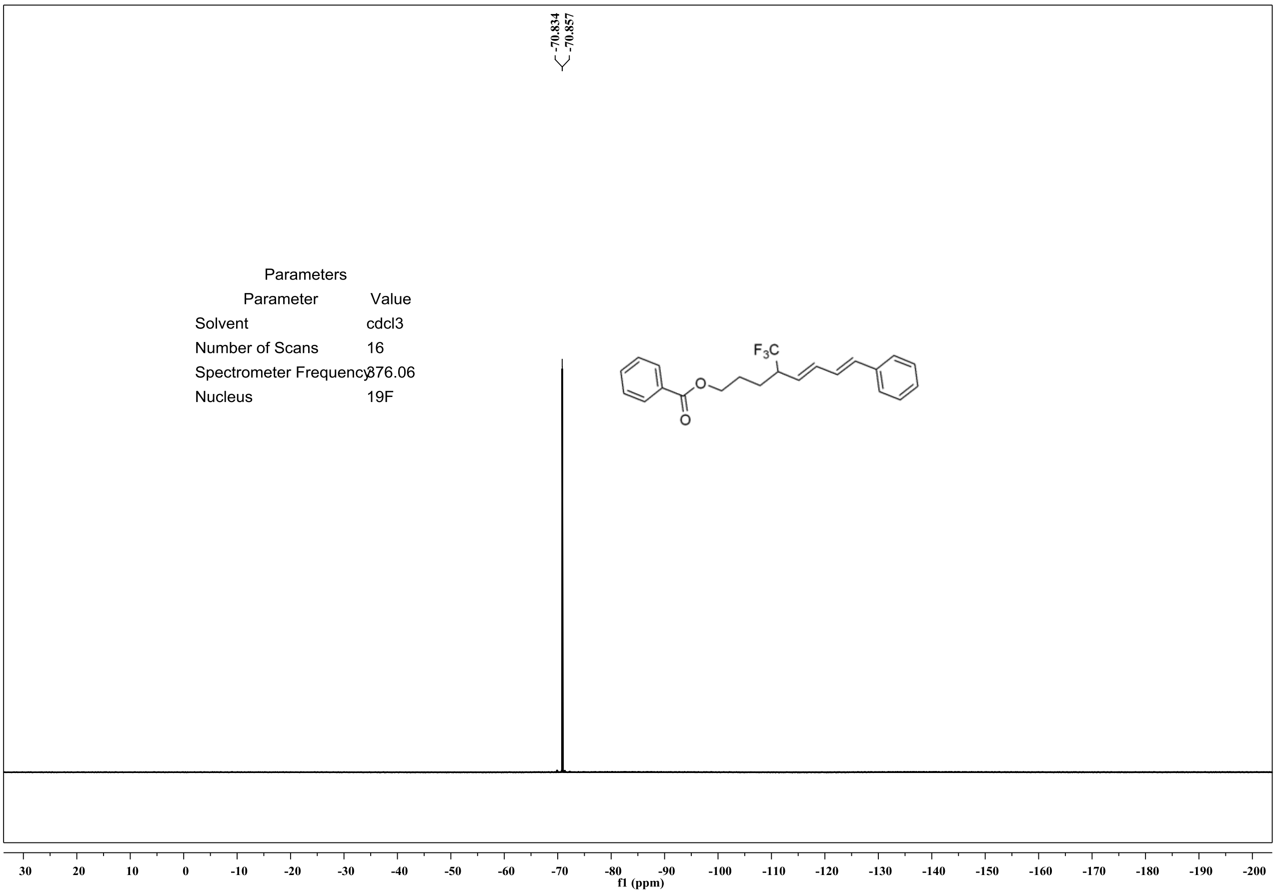

-70.834
-70.857
Parameters
Parameter Value
Solvent cdcl3
Number of Scans 16
Spectrometer Frequency 376.06
Nucleus 19F
F_3C
O
O
f1 (ppm)

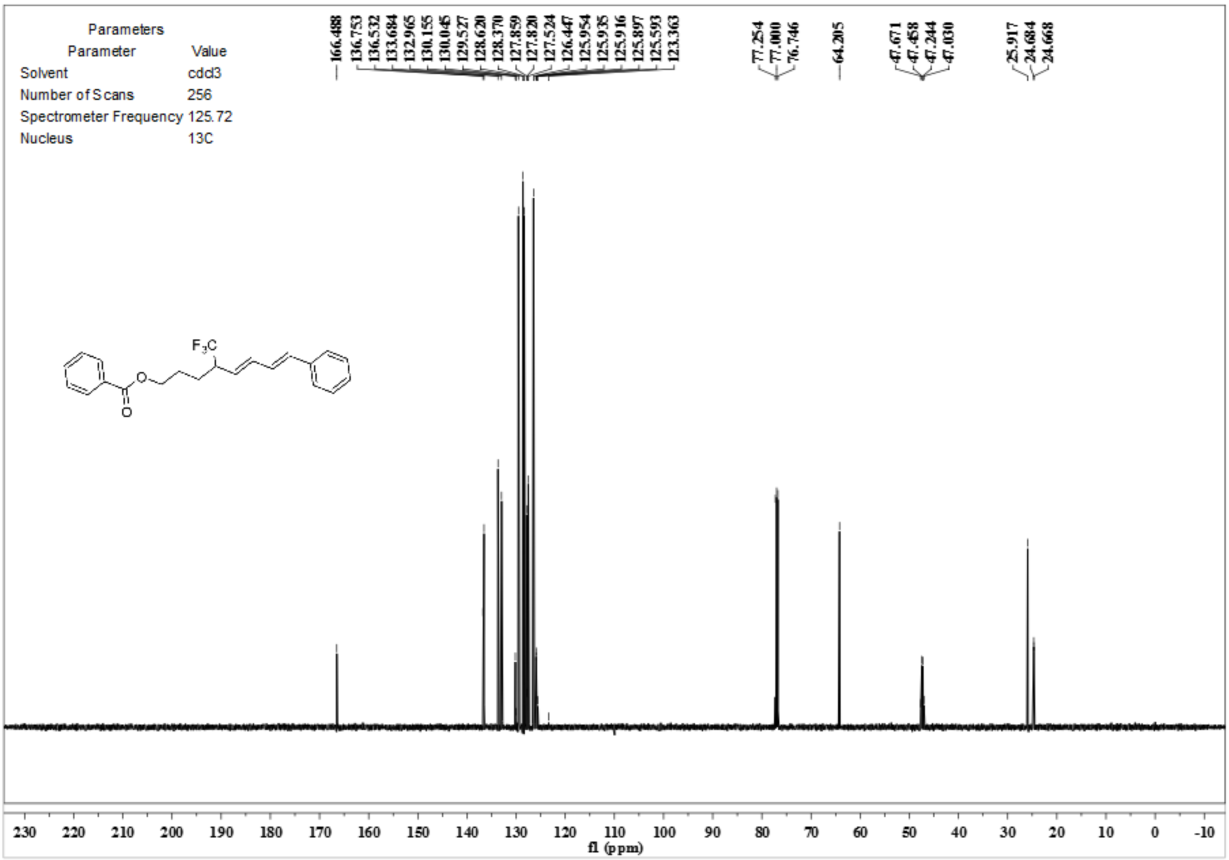

Parameters
Parameter Value
Solvent cdcl3
Number of Scans 256
Spectrometer Frequency 125.72
Nucleus 13C
166.488
136.753
136.532
133.684
132.965
130.155
130.045
129.527
128.620
128.370
127.859
127.820
127.524
126.447
125.954
125.935
125.916
125.897
125.593
123.363
77.254
77.000
76.746
64.205
47.671
47.458
47.244
47.030
25.917
24.684
24.668
F_3C
O
O
f1 (ppm)

(*E*)-6-(Naphthalen-2-yl)-4-(trifluoromethyl)hex-5-en-1-yl benzoate (4i).

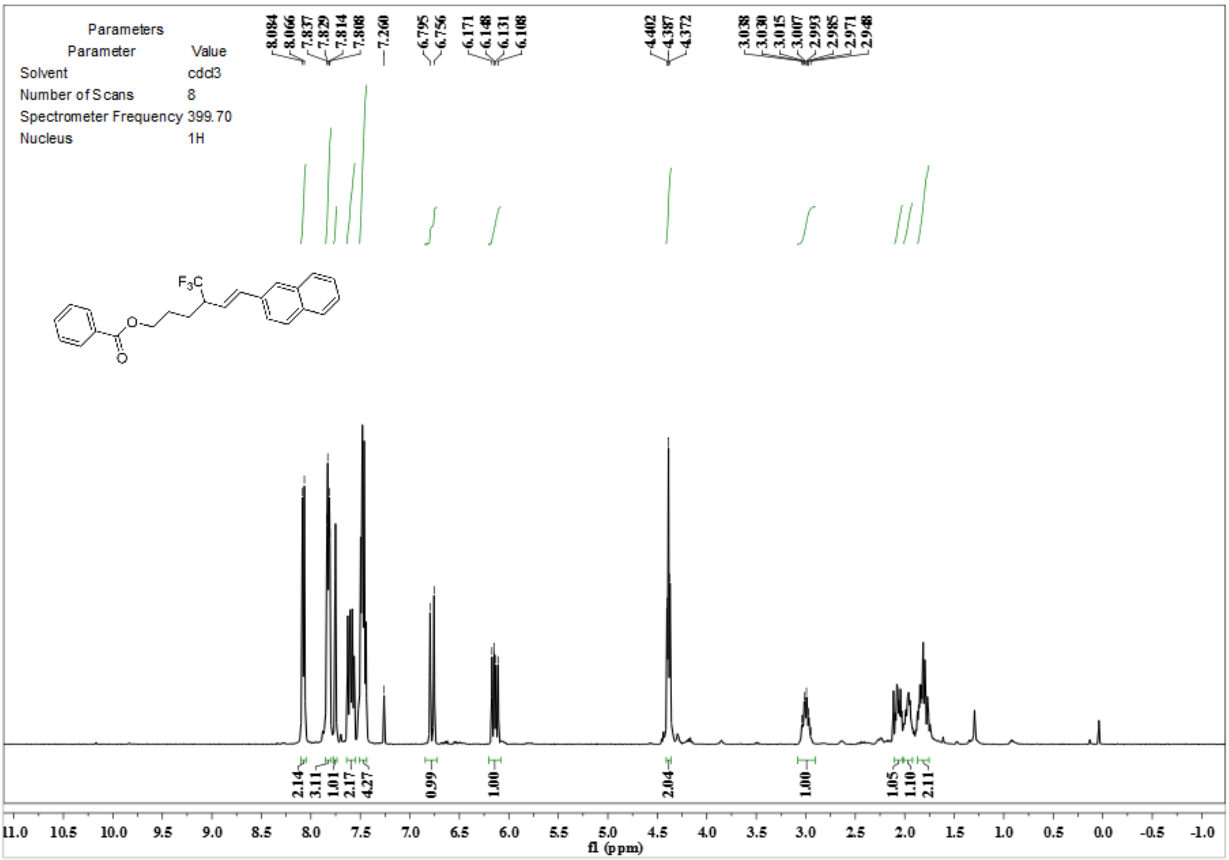

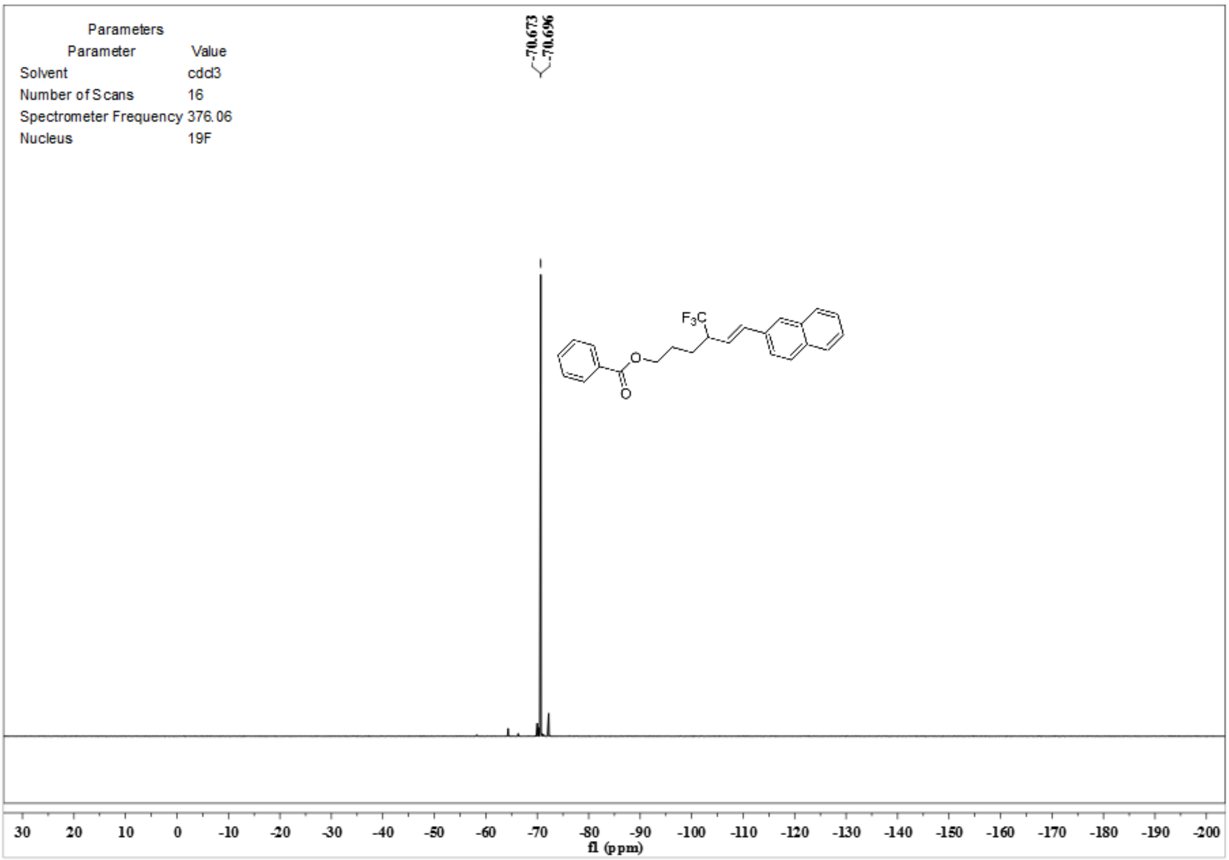

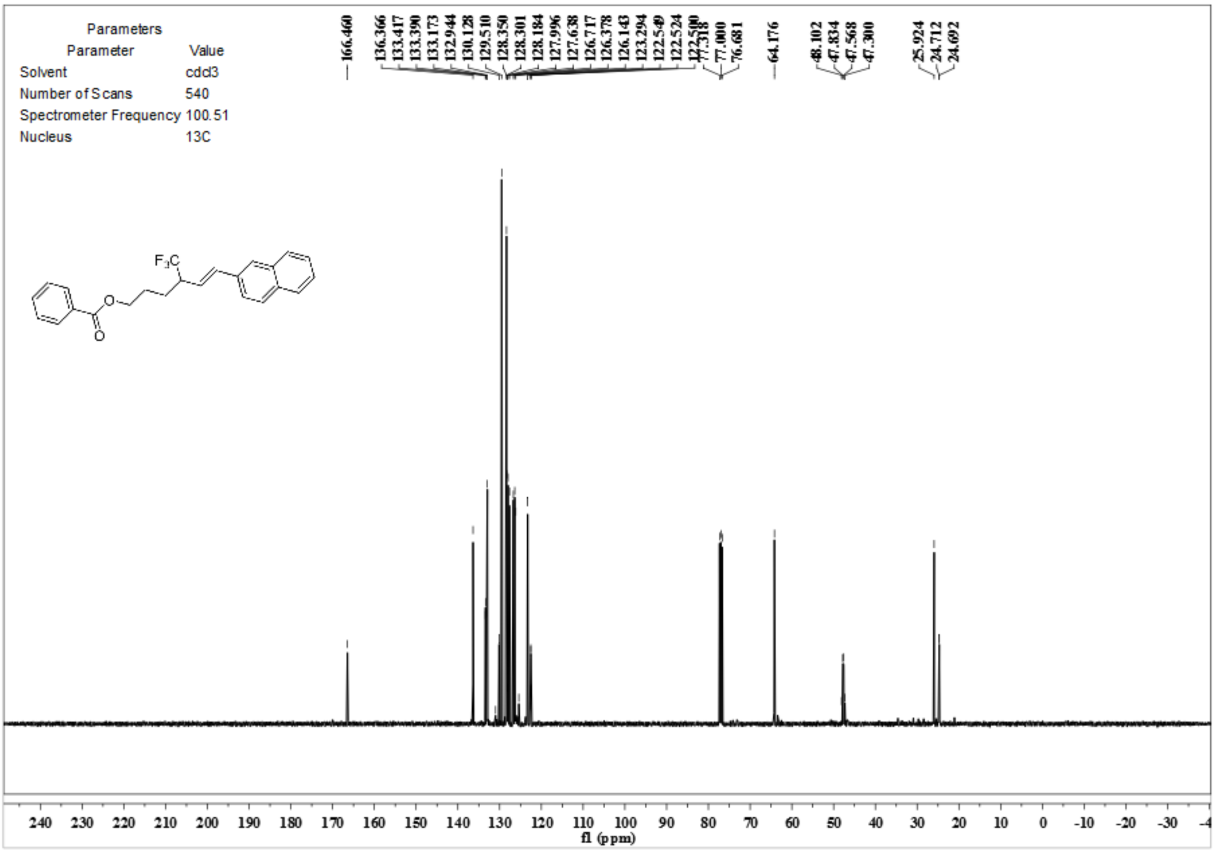

(*E*)-2-(6-(Naphthalen-2-yl)-4-(trifluoromethyl)hex-5-en-1-yl)isoindoline-1,3-dione (4j).

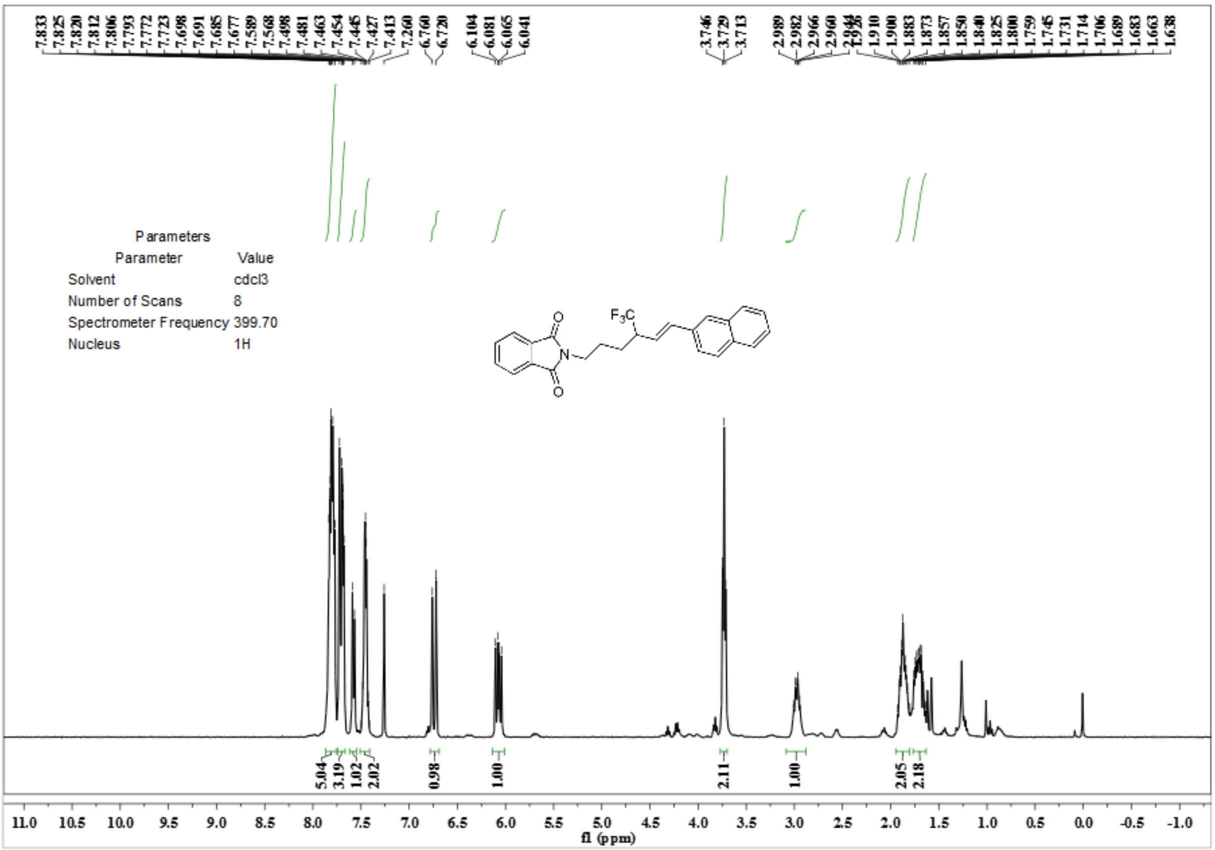

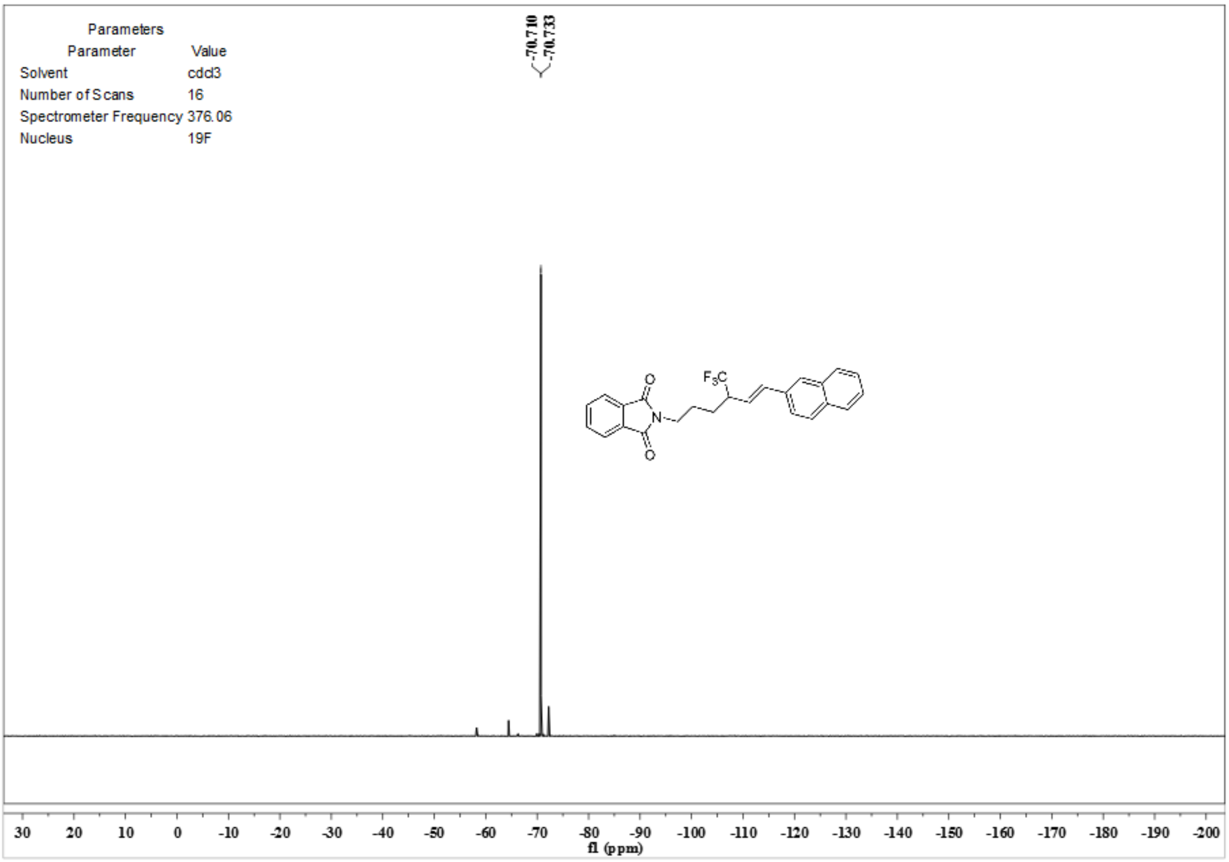
Parameters
Parameter Value
Solvent cdcl3
Number of Scans 16
Spectrometer Frequency 376.06
Nucleus 19F
-70.710
-70.733
f1 (ppm)

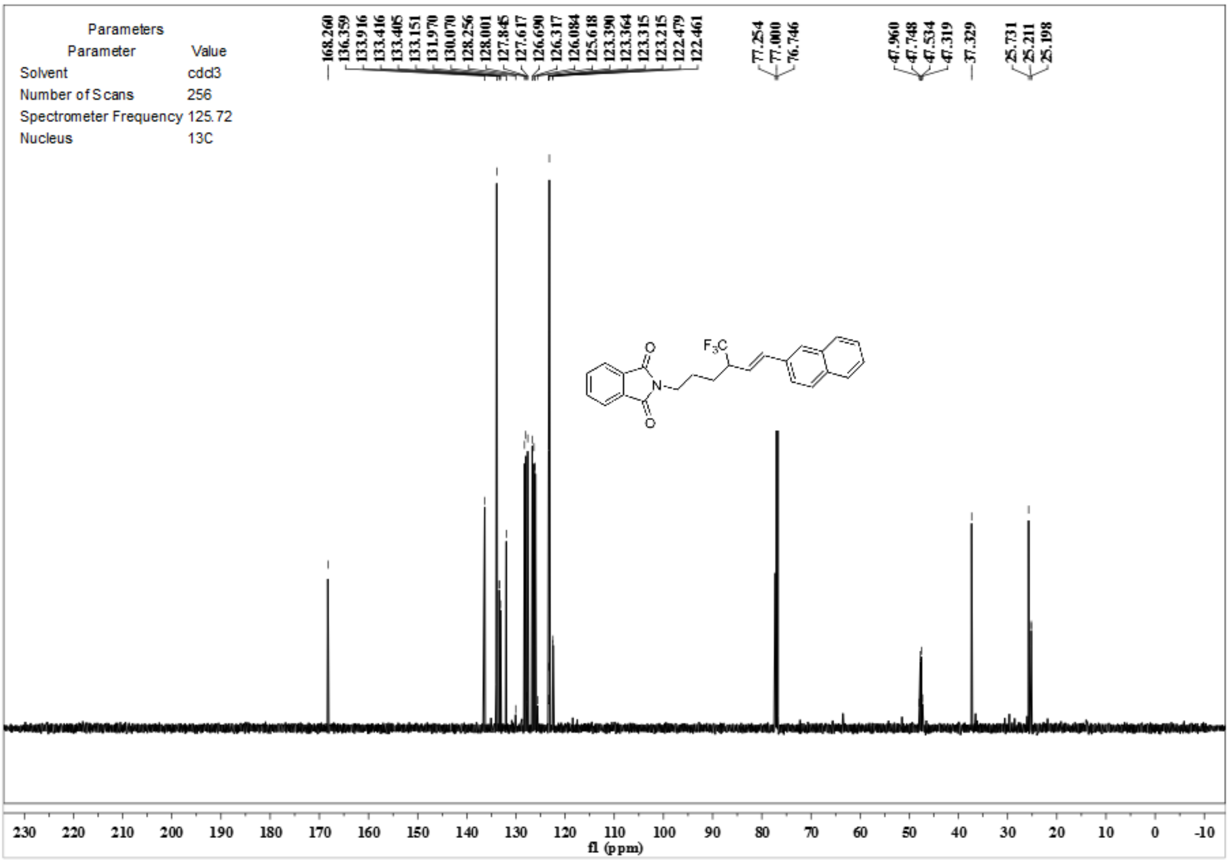
Parameters
Parameter Value
Solvent cdcl3
Number of Scans 256
Spectrometer Frequency 125.72
Nucleus 13C
168.260
136.359
133.916
133.416
133.405
133.151
131.970
130.070
128.256
128.001
127.845
127.617
126.690
126.317
126.084
125.618
123.390
123.364
123.315
123.215
122.479
122.461
77.254
77.000
76.746
47.960
47.748
47.534
47.319
37.329
25.731
25.211
25.198
f1 (ppm)

(*E*)-N-(6-(4-chlorophenyl)-4-(trifluoromethyl)hex-5-en-1-yl)-N-formylformamide (4k).

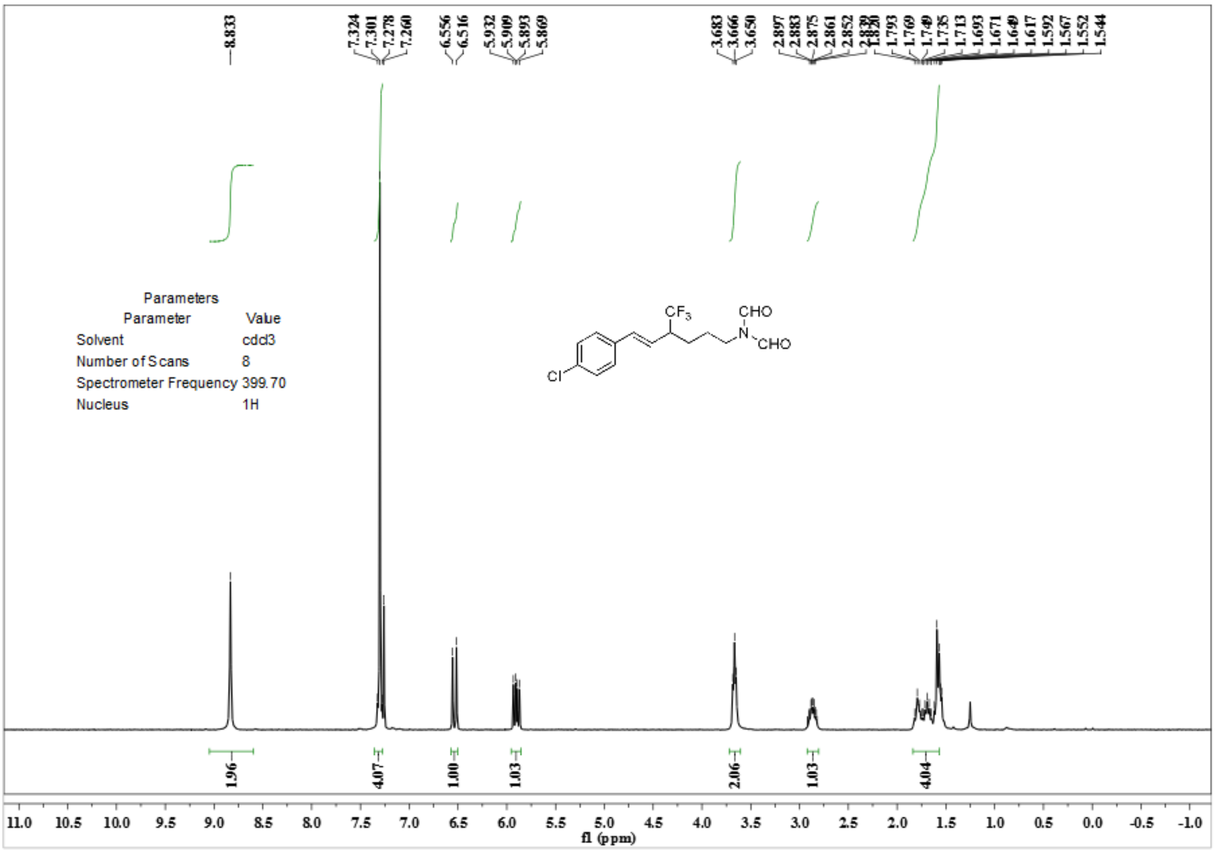

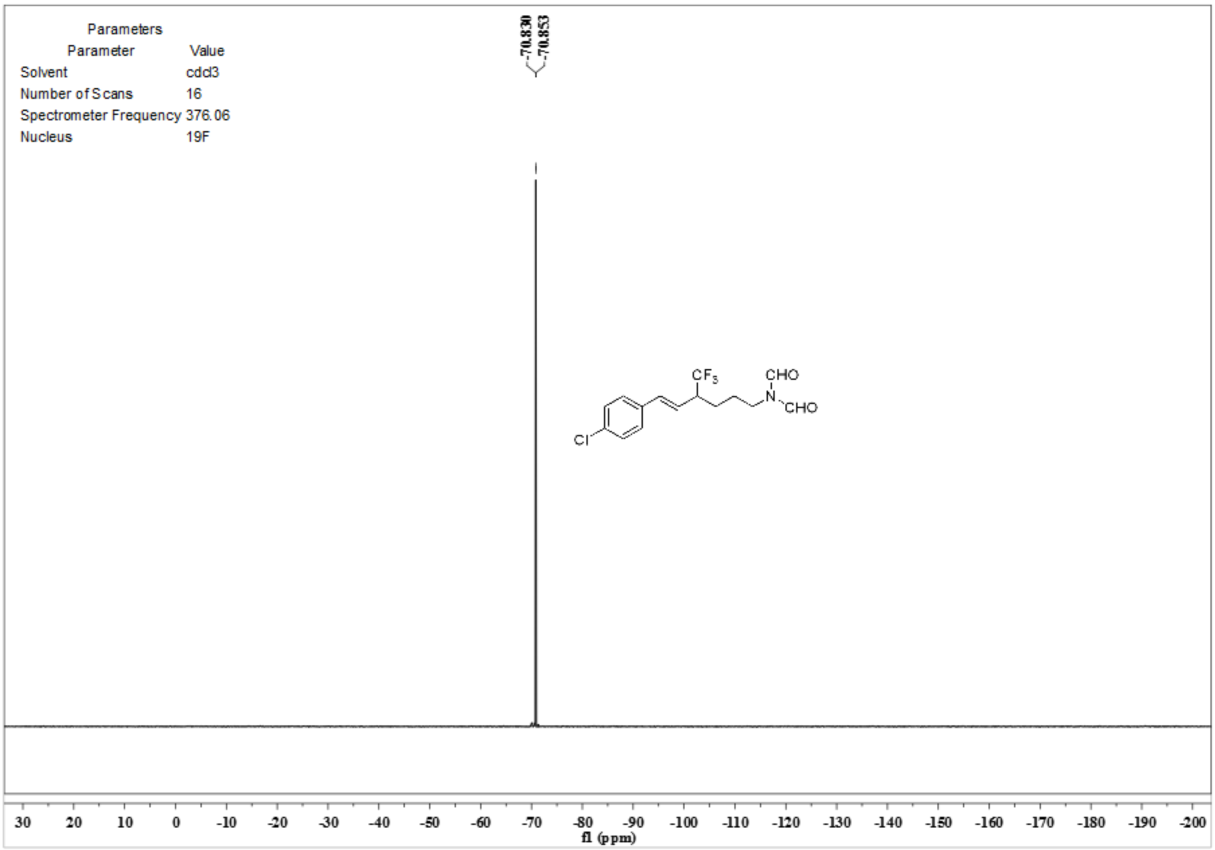

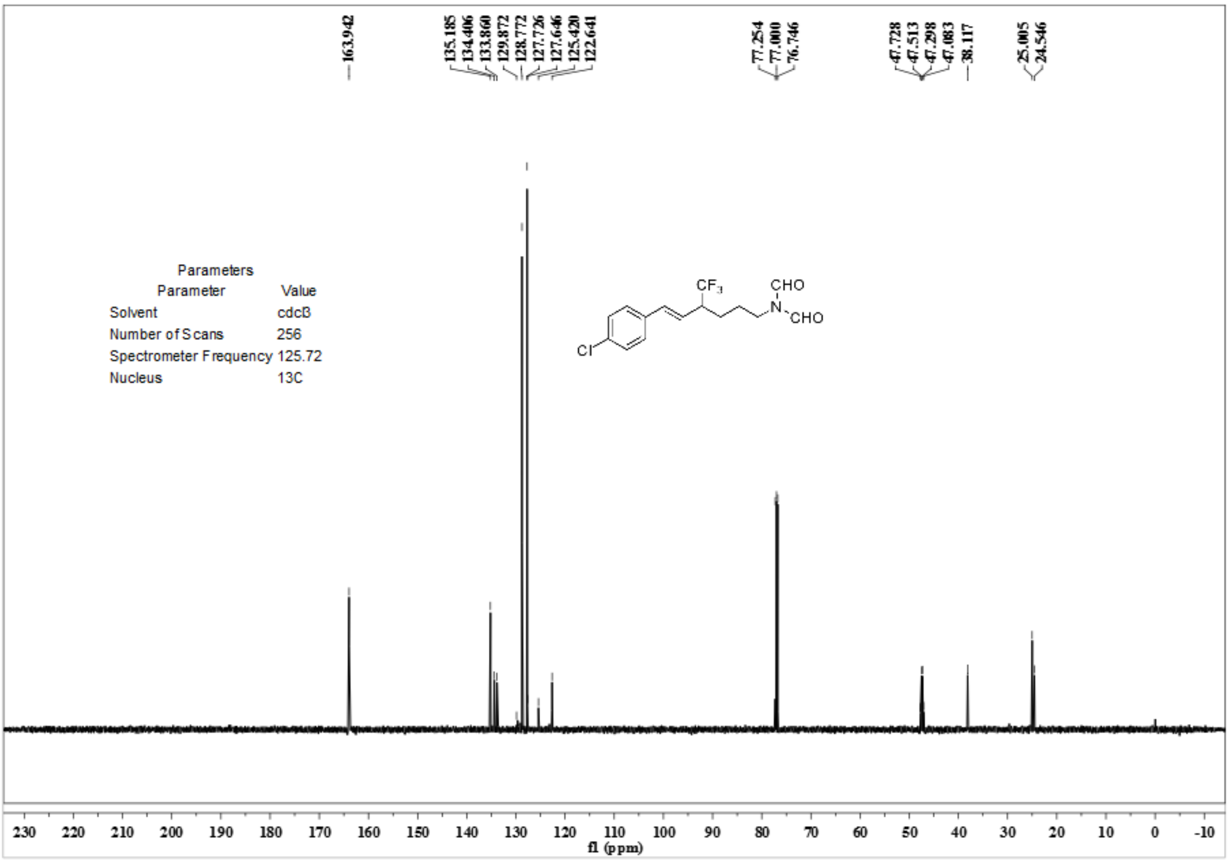

Ethyl (*E*)-2,2-difluoro-3-(4-methoxystyryl)heptanoate (4l).

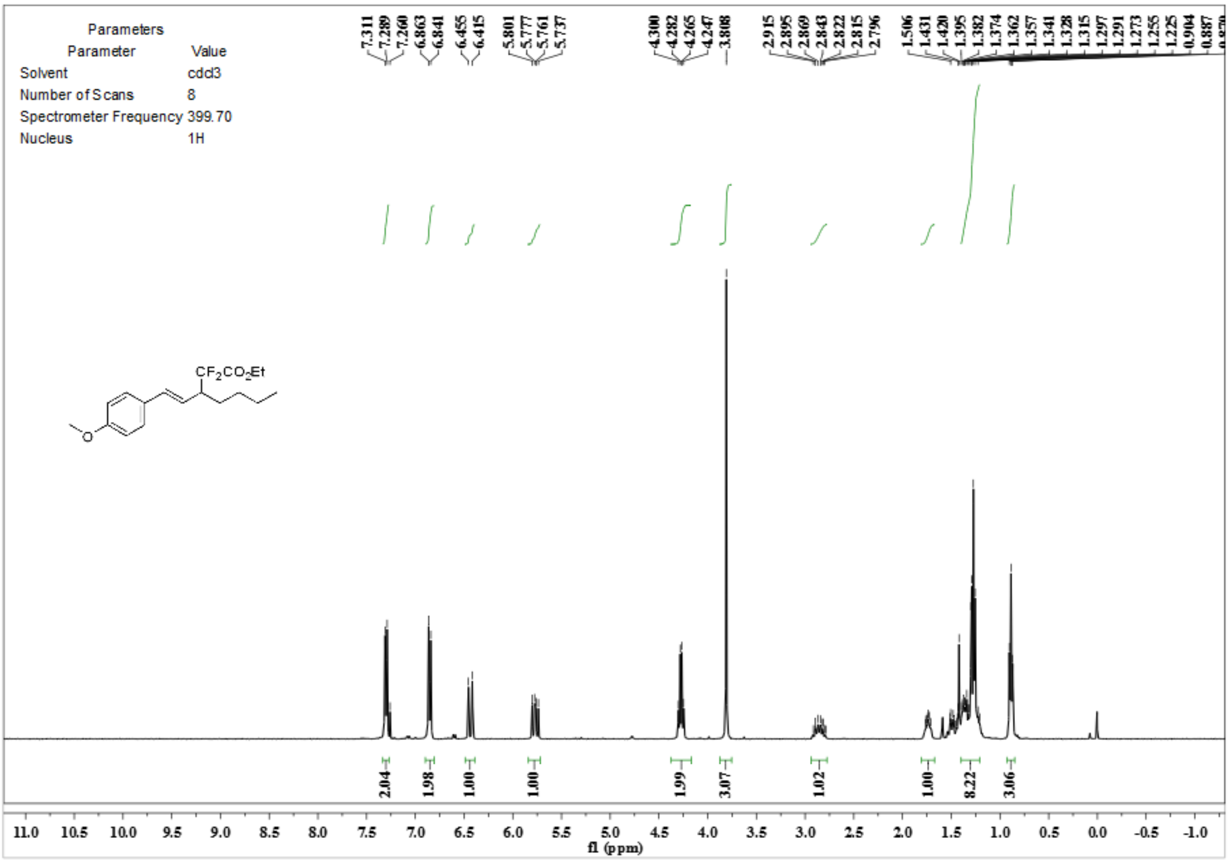

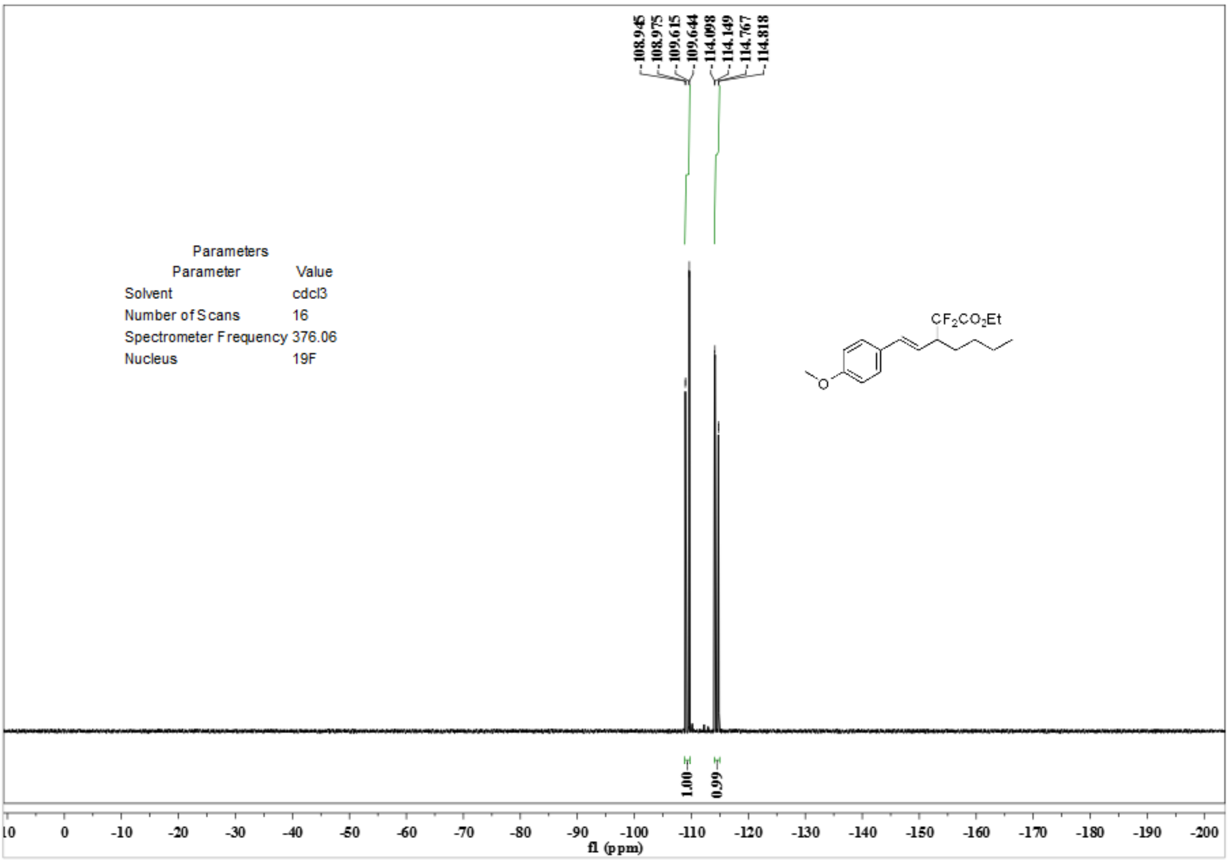

Parameters
Parameter Value
Solvent cdcl3
Number of Scans 16
Spectrometer Frequency 376.06
Nucleus 19F
CF_2CO_2Et
fl (ppm)

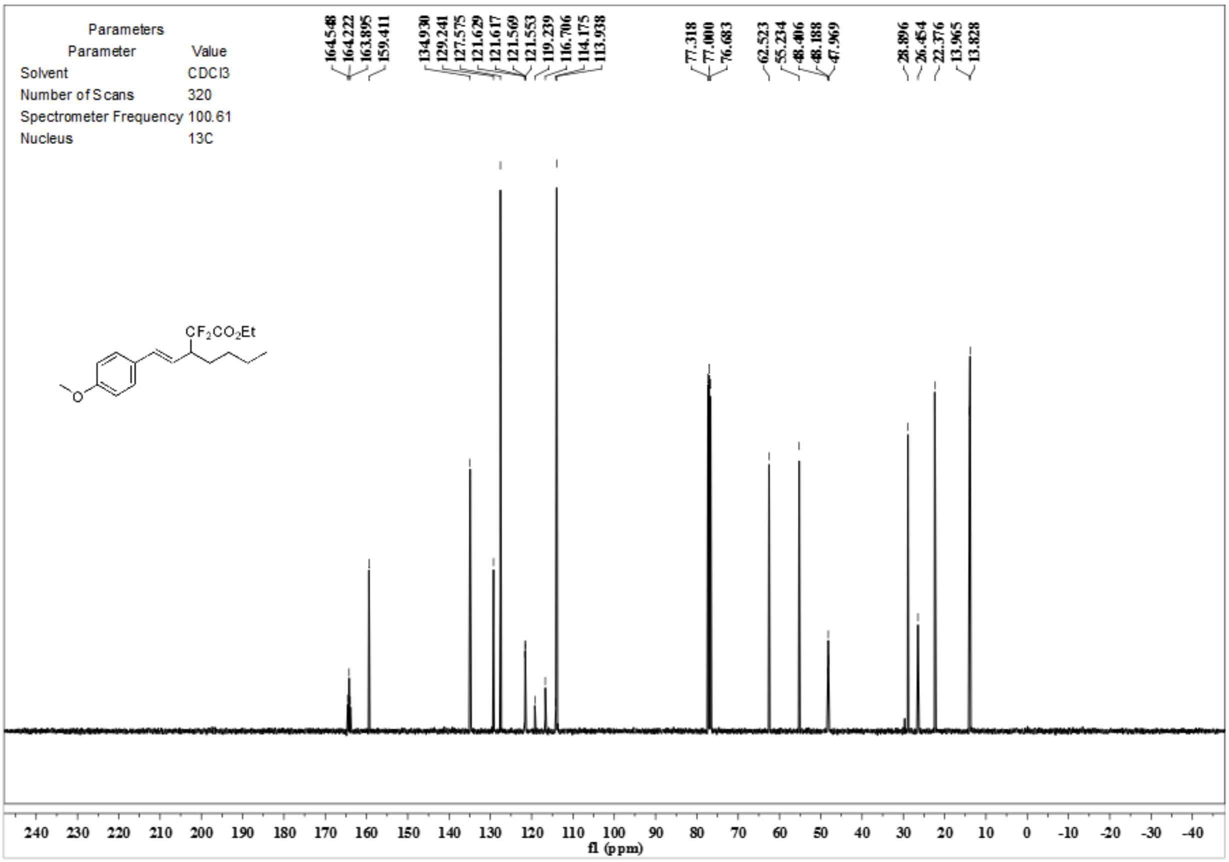

Parameters
Parameter Value
Solvent CDCl3
Number of Scans 320
Spectrometer Frequency 100.61
Nucleus 13C
CF_2CO_2Et
fl (ppm)

Ethyl (*E*)-2,2-difluoro-3-(4-fluorostyryl)heptanoate (4m).

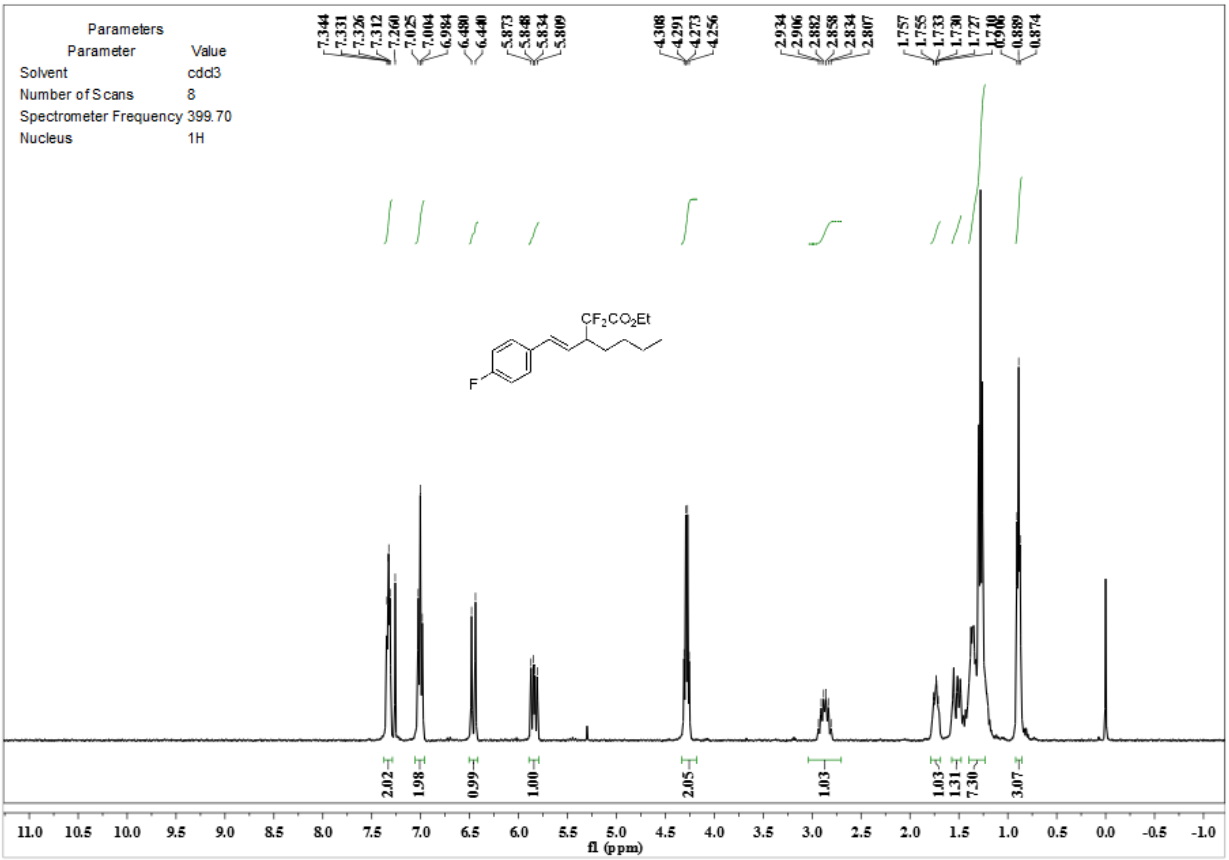

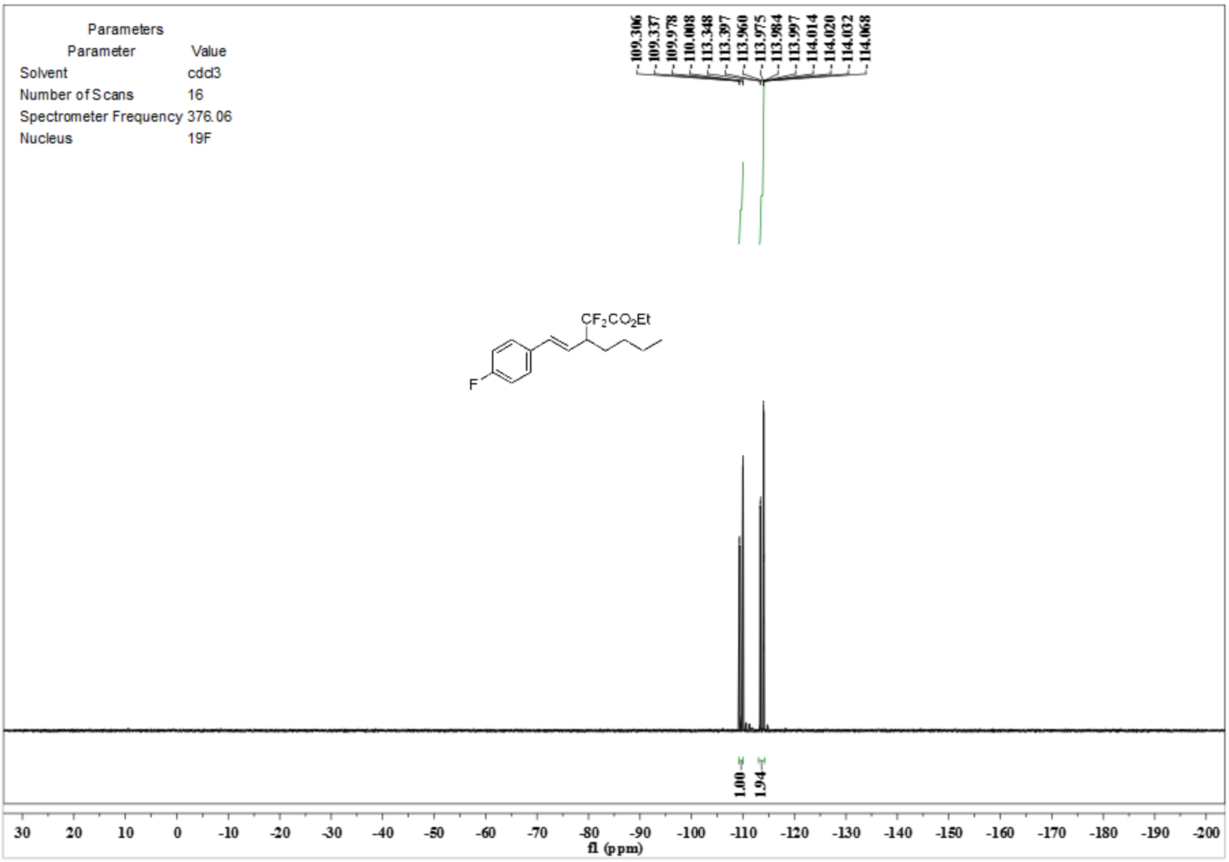

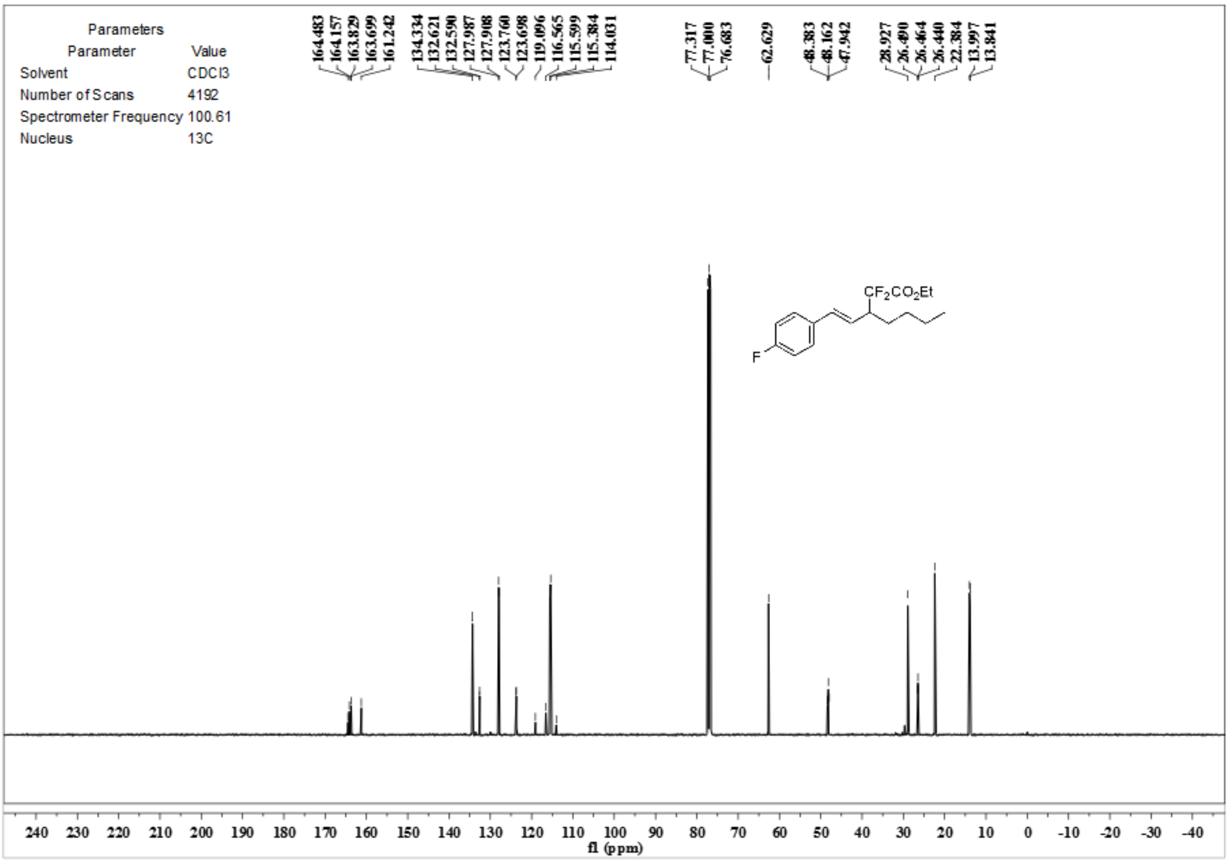

4-(2-(trifluoromethyl)hexyl)-1,2-dihydronaphthalene (6).

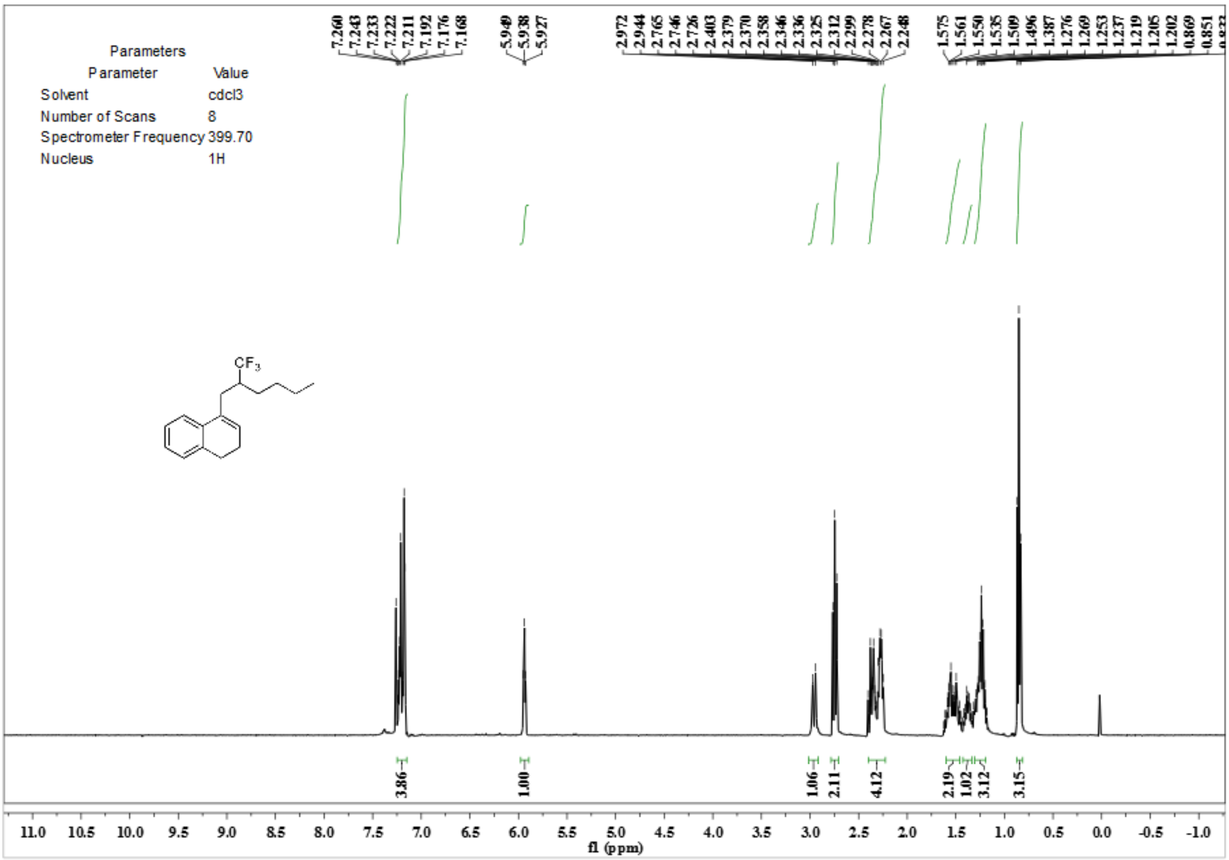

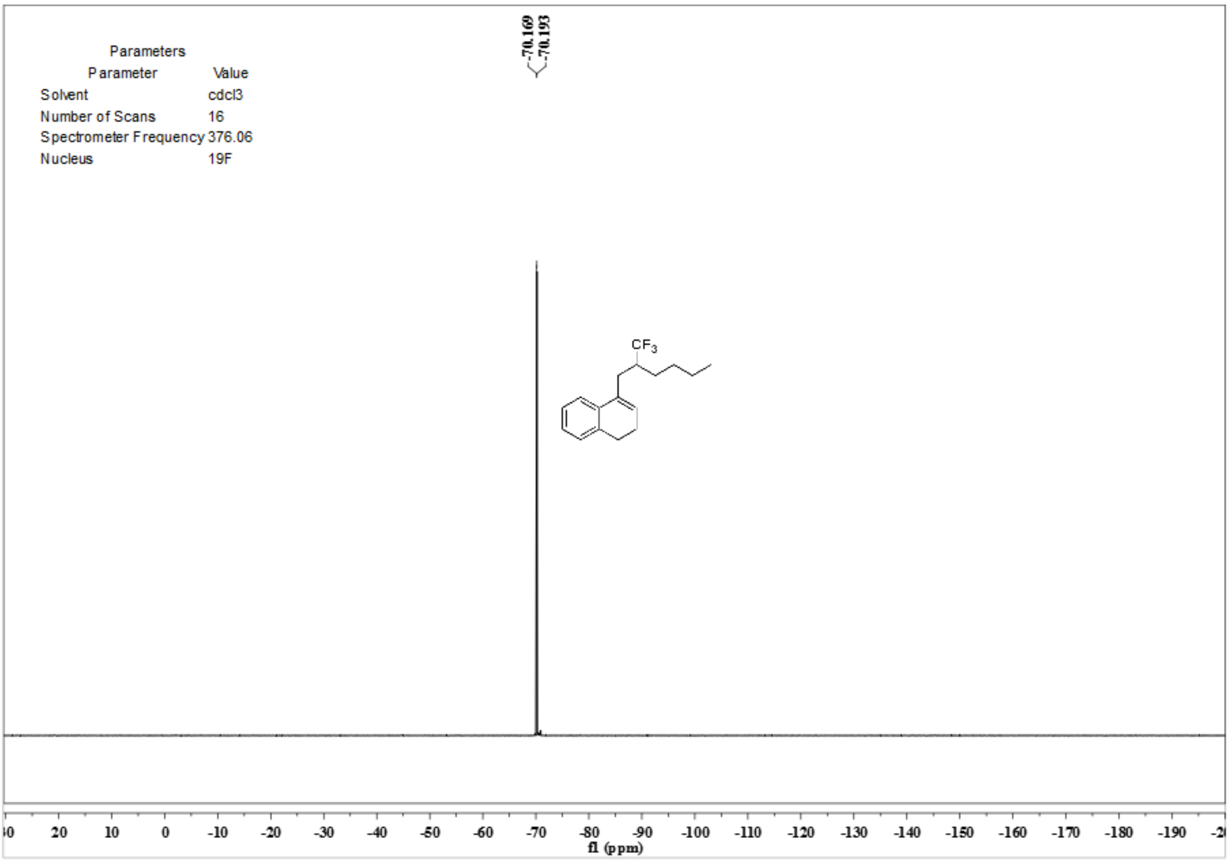

-70.169
-70.193
Parameters
Parameter Value
Solvent cdcl3
Number of Scans 16
Spectrometer Frequency 376.06
Nucleus 19F
CF3
f1 (ppm)

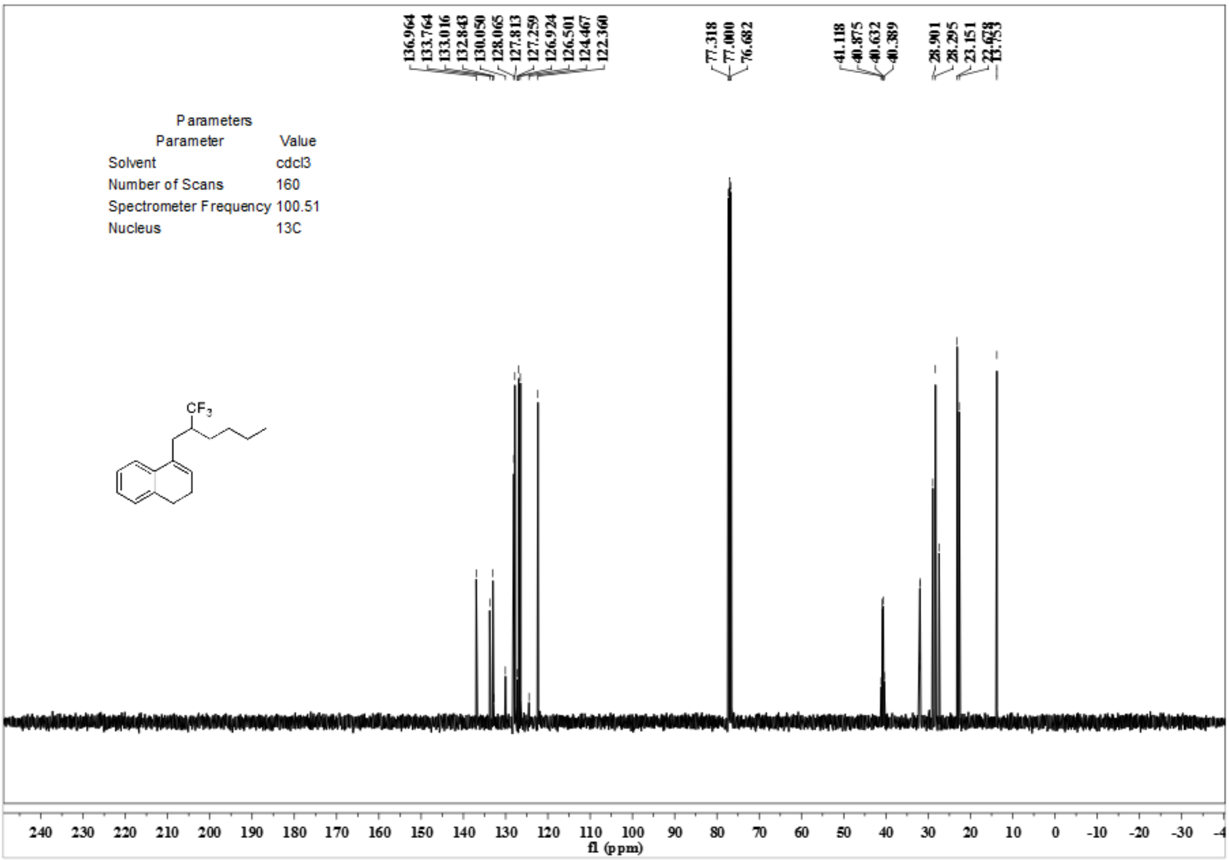

136.964
133.764
133.016
132.843
130.050
128.065
127.813
127.259
126.924
126.501
124.467
122.360
77.318
77.000
76.682
41.118
40.875
40.632
40.389
28.901
28.295
23.151
22.678
13.753
Parameters
Parameter Value
Solvent cdcl3
Number of Scans 160
Spectrometer Frequency 100.51
Nucleus 13C
CF3
f1 (ppm)
